# Association between immune cell subtypes and membranous nephropathy: A bidirectional Mendelian randomization study

**DOI:** 10.1097/MD.0000000000042774

**Published:** 2025-06-06

**Authors:** Yiyang Yu, Guangxu Liu, Rui Zhang, Baojiang Chen, Zhongqiu Luan

**Affiliations:** a Heilongjiang University of Chinese Medicine, Harbin, Heilongjiang, China; b Renshou County Chinese Medicine Hospital, Meishan, Sichuan, China; c First Affiliated Hospital of Heilongjiang University of Chinese Medicine, Harbin, Heilongjiang, China.

**Keywords:** causal association, immune cell subtypes, immunocyte, membranous nephropathy, Mendelian randomization

## Abstract

Prior studies have explored the contribution of immune cells in membranous nephropathy (MN). This study utilized Mendelian randomization (MR) to further explore 731 immune cell subtypes on MN, offering robust evidence for their causality. Genetic information for 731 subtypes of immune cells and MN was obtained from genome-wide association studies. Inverse variance weighting (IVW) was employed as the primary analytical method. To reinforce the findings, additional methods, MR-Egger, weighted median, and weighted mode, were employed, along with sensitivity analyses to ensure robustness and stability. Using the IVW method, MR analysis identified significant positive correlations between 17 immune cell subtypes and MN (*P* < .05, OR > 1) and negative correlations for 29 subtypes (*P* < .05, OR < 1). In the reverse MR analysis, MN exhibited positive associations with 2 subtypes (*P* < .05, OR > 1) and negatively correlated with another 4 subtypes (*P* < .05, OR < 1). None of these associations showed signs of horizontal pleiotropy (*P* > .05). This extensive bidirectional two-sample MR analysis provides insights into the complex causal links between certain immune subtypes and MN, underscoring the sophisticated interactions between components of the immune system and MN.

## 1. Introduction

Membranous nephropathy (MN) is a glomerular disease pathologically characterized by thickened glomerular capillary walls due to immune complex deposition on the external side of the basement membrane. It affects individuals across various age groups, geographic regions, and ethnicities. The average age at diagnosis ranges from 50 to 60 years, and males are about twice as likely to be diagnosed as females, although the underlying reasons for this disparity remain unclear.^[1]^ In North America, the occurrence of MN is estimated to be between 10 and 12 instances per million people yearly, while in Europe, it ranges from 2 to 17 cases per million individuals annually.^[2,3]^ MN constitutes 24.9% of primary glomerular diseases in China, with its occurrence showing a consistent upward trend in recent years.^[4]^ MN presents with a spectrum of clinical manifestations, ranging from mild symptoms to severe nephrotic syndrome characterized by hypoalbuminemia and generalized edema, which may progress to end-stage renal disease. Considerable progress has been achieved in elucidating the pathophysiological mechanisms of membranous nephropathy. A key finding has been the discovery of the M-type phospholipase A2 receptor which is an antigen located on podocytes that is targeted by specific antibodies. These antibodies can be detected in approximately 70% of adult cases, affirming its classification as an autoimmune disease.^[5]^ In recent years, researchers have identified several additional antigens that may contribute to the diverse causes of MN, including thrombospondin type-1 domain-containing 7A, exostosin 1 and exostosin 2, as well as the serine protease HtrA1.^[6–8]^ These newly discovered antigens are believed to play potential roles in the various etiologies of the disease. Each antigen-associated subtype of MN is characterized by specific immunological features, including IgG subclass profiles, distinct patterns of associated comorbidities, and clinical outcomes.

Despite significant advances, the precise etiology of MN remains elusive, and it is widely hypothesized to involve autoimmune mechanisms. Immune cells are pivotal in MN pathogenesis, primarily through antigen presentation and autoantibody production, critical for immune complex formation.^[7,9]^ Studies have highlighted that a disruption in the balance between regulatory T cells and B cells is critical in the development of MN, given their involvement in generating immune complexes associated with MN.^[10–12]^ Moreover, treatments aimed at B cells, including rituximab, have demonstrated potential in addressing MN.^[13,14]^ Additionally, elevated levels of circulating Th17 cells have been positively linked to an increased risk of MN, while regulatory T cells seem to offer protective effects against the condition.^[15]^ These findings emphasize the promise of immunomodulatory approaches in MN management. Nevertheless, particular immune cell subtypes in the development of MN and their direct association with MN progression remain unclear. To fill the gap, we investigated the intricate causal and reverse causal relationships between 731 immune cell subtypes and MN utilizing Mendelian randomization (MR) analysis. By integrating comprehensive data on immune cell subtypes, this study provides nuanced insights into the genetic determinants driving MN pathogenesis and progression. This approach lays the groundwork for identifying novel therapeutic targets and refining treatment strategies. To our knowledge, this study is the first to systematically evaluate such a broad range of immune cell subtypes for causal links with MN, highlighting its novel contribution to understanding MN pathogenesis.

MR is a powerful analytical method that leverages genetic data to investigate causality in observational studies.^[16]^ In MR analysis, genetic variants strongly associated with the exposure of interest serve as instrumental variables to assess the causality between exposures and outcomes.^[17,18]^ As these instruments are genetically based, they are unaffected by confounding factors. A progressive variation of conventional techniques, Bidirectional MR has proven essential in deciphering the complex, two-way interactions found in biological systems.^[19]^ This approach is particularly valuable for understanding the reciprocal relationship between exposures and outcomes. The goal of this study is to apply MR analysis to unravel the complex causal relationships between 731 immune cell subtypes and MN.

## 2. Methods and materials

**Figure 1.**
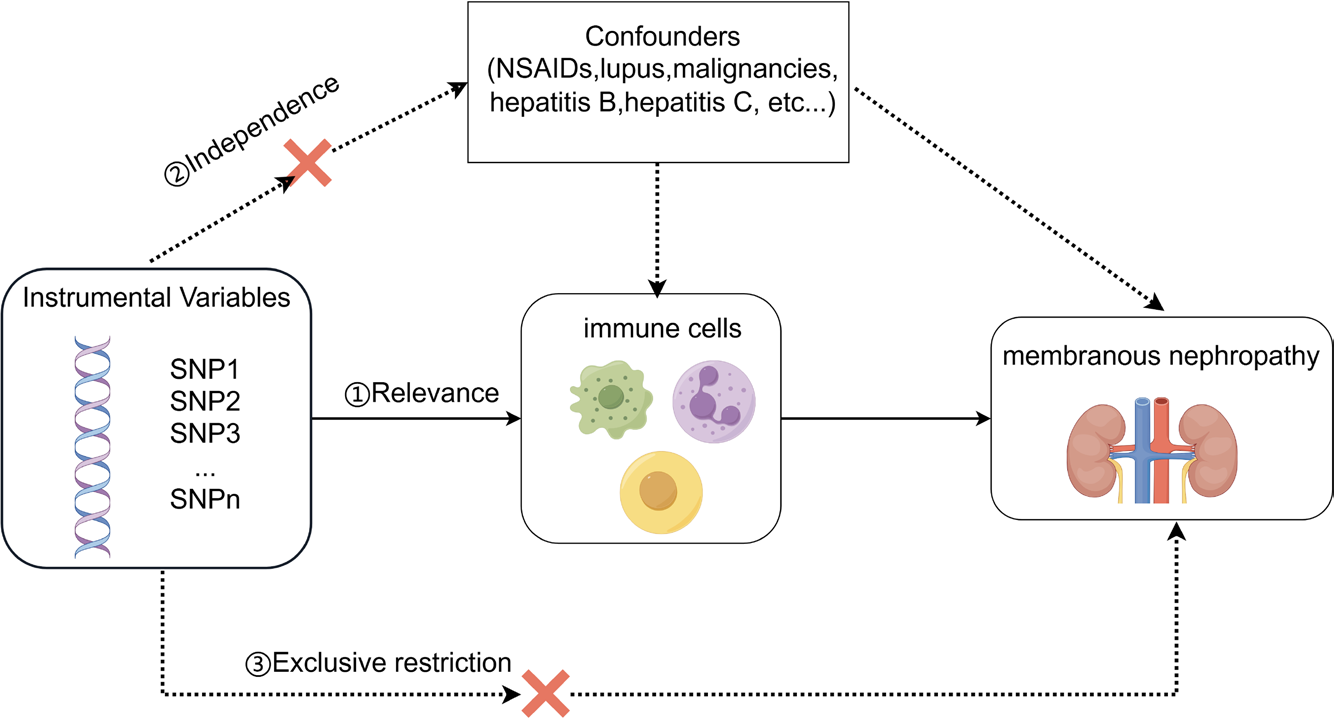
Visual overview of the methodology used in the Mendelian randomization study. (By Figdraw). Dashed lines indicate potential direct causal relationships that may violate the assumptions. By leveraging genetically predicted immune cell traits, this study aims to investigate their association with membranous nephropathy, providing insights into its potential underlying etiology.

The bidirectional two-sample MR approach was employed to explore the causal relationship between 731 immune cell subtypes and MN, utilizing summary statistics of genome-wide association studies (GWAS). Three essential assumptions must be satisfied in each MR study: (1) the genetic variant is strongly associated with the exposure of interest; (2) the genetic variant is independent of confounders; (3) the genetic variant affects the outcome solely through its effect on the exposure. The study’s overall design is depicted in Figure 1. Data for this analysis were sourced from publicly available GWAS databases, with all data desensitized to protect personal privacy and prevent the inclusion of identifiable information. No institutional data or informed consent was required for this study, and thus, ethical review was not necessary. Clinical trial number: not applicable.

### 2.1. Exposure and outcome data sources

From GWAS summary statistics reported by Orru et al, genetic tools for 731 immune cell subtypes were identified at suggestive genome-wide significance thresholds (*P* < 1 × 10⁻⁵).^[20]^ That study analyzed data from 3757 European participants utilizing flow cytometry techniques. We accessed these summary statistics via the GWAS Catalog (GCST0001391–GCST0002121). For the MN cohort, we selected a GWAS dataset (ebi-a-GCST010005)^[21]^ of 7979 European individuals (2150 cases and 5829 controls) with 5,327,688 SNPs. We considered key factors such as matching ancestry, sample size, and SNP density to ensure a suitable comparison. Notably, this dataset included only primary MN cases and utilized an up-to-date genome-wide reference panel to ensure high-quality genotyping data.

### 2.2. Selection of instrumental variable

For the forward MR analysis, with immune cell traits as exposures, we selected SNPs strongly associated with each trait at a significance threshold of *P* < 1 × 10⁻⁵. To ensure the independence of these SNPs, we applied stringent linkage disequilibrium pruning with an *r*² < 0.001 and a distance of 10,000 kb.

For the reverse MR analysis, using MN as the exposure, we used a more relaxed linkage disequilibrium pruning threshold of *r*² < 0.01 and a 1000 kb distance. This adjustment was necessary because disease has fewer genome-wide significant SNPs, requiring a less stringent threshold to secure sufficient independent instrumental variables. Additionally, the smaller window size of 1000 kb helped prevent the over-concentration of instrumental variables on specific chromosomal regions, reducing potential bias.

We checked all candidate SNPs in the PhenoScanner V2 database to screen for potential confounding associations, thereby upholding the MR independence assumption. We then calculated the F-statistic for each SNP, (F = β²/SE², β represents the effect size and SE is the standard error),^[22]^ and removed those with F < 10 to avoid weak instrument bias. Next, we harmonized allele orientations by aligning effect alleles for each SNP between the exposure and outcome datasets, ensuring consistent effect directions. Finally, SNPs that were palindromic with intermediate allele frequencies or had incompatible alleles between datasets were excluded to improve the robustness of the analysis.

### 2.3. Statistical analysis

We primarily used the inverse variance weighted (IVW) method to estimate the causal effect of each exposure under standard MR assumptions.^[23]^ If there was evidence of heterogeneity, we employed a random-effects IVW model instead of a fixed-effects model.^[24]^ We also applied the weighted median method, giving reliable estimates even if some variants are invalid.^[25]^ We further performed sensitivity analyses using the weighted mode and MR-Egger methods to test the consistency of our findings.^[26]^ We tested for heterogeneity among the genetic variants using Cochran Q test, with *P* < .05 indicates significant heterogeneity.^[27]^ To detect and correct horizontal pleiotropy, we used the MR-PRESSO test (Mendelian Randomization Pleiotropy Residual Sum and Outlier).^[28]^ To assess the robustness and symmetry of the results, we conducted leave-one-out sensitivity analyses and visualized the data with funnel and scatter plots.^[29]^ All results were considered statistically significant at a threshold of *P* < .05. Analyses were performed using R software (version 4.4.1) and the TwoSampleMR package (version 0.6.8).

## 3. Results

### 3.1. Selection of forward instrumental variables

In this study, we analyzed GWAS data for 731 immune cell subtypes to identify genetic variants with significant associations that could be used as instrumental variables. And all selected variants had F-statistics exceeding 10, ensuring the strength of the instruments. Detailed information about the instrumental variables is presented in Table 1.

**Table 1:** SNPs screened for all positive results.

ID	nSNP	*P*val (IVW)	Traits
GCST90001428	9	.048292	IgD- CD38dim %B cell
GCST90001469	10	.037519	CD62L- myeloid DC %DC
GCST90001471	9	.036144	CD62L- plasmacytoid DC %DC
GCST90001477	11	.041537	HLA DR++ monocyte AC
GCST90001484	10	.0127	CD39 + resting Treg %resting Treg
GCST90001491	8	.017605	CD39 + activated Treg %CD4 Treg
GCST90001495	10	.035975	CD39 + secreting Treg AC
GCST90001595	4	.033462	DP (CD4 + CD8+) %T cell
GCST90001607	8	.026516	CD8br %leukocyte
GCST90001621	20	.045995	NKT AC
GCST90001678	7	.046802	CD28- CD25++ CD8br AC
GCST90001696	9	.045998	CD45RA- CD28- CD8br %CD8br
GCST90001699	8	.044135	CD45RA + CD28- CD8br %CD8br
GCST90001707	5	.032278	BAFF-R on IgD + CD38- unsw mem
GCST90001709	10	.042714	BAFF-R on IgD + CD38dim
GCST90001728	7	.027543	CD19 on IgD + CD38- unsw mem
GCST90001779	7	.04542	CD25 on IgD + CD24-
GCST90001797	10	.034477	CD27 on CD20- CD38-
GCST90001856	14	.030975	CD3 on CD39 + secreting Treg
GCST90001862	7	.036929	CD3 on CD28 + DN (CD4-CD8-)
GCST90001863	8	.007906	CD3 on CD28 + CD45RA- CD8br
GCST90001869	13	.036552	CD3 on resting Treg
GCST90001870	9	.039869	CD34 on HSC
GCST90001877	9	.019444	HVEM on naive CD4+
GCST90001886	4	.013021	CD28 on CD39 + activated Treg
GCST90001887	10	.044383	CD28 on secreting Treg
GCST90001889	9	.009295	CD28 on activated & secreting Treg
GCST90001892	5	.003807	CD28 on CD39 + CD4+
GCST90001900	4	.006812	CD28 on resting Treg
GCST90001902	10	.046405	CD28 on activated Treg
GCST90001925	9	.034571	CD127 on CD28 + DN (CD4-CD8-)
GCST90001929	5	.021377	CD127 on CD28 + CD45RA + CD8br
GCST90001988	8	.034621	HLA DR on CD14 + CD16- monocyte
GCST90001991	8	.034786	HLA DR on CD14 + monocyte
GCST90002000	9	.001377	PDL-1 on CD14- CD16-
GCST90002001	9	.021637	CD64 on CD14- CD16-
GCST90002009	11	.003074	HLA DR on CD14- CD16-
GCST90002023	10	.026754	CD4 on CM CD4 +
GCST90002028	9	.038373	CD19 on B cell
GCST90002057	7	.040646	CD8 on TD CD8br
GCST90002069	6	.015638	CD4 on CD39 + secreting Treg
GCST90002091	13	.031726	CD11b on CD14 + monocyte
GCST90002095	10	.039721	CD11b on CD33dim HLA DR-
GCST90002096	9	.034082	CD11b on basophil
GCST90002112	8	.001024	HLA DR on CD33- HLA DR+
GCST90002116	9	.035236	HLA DR on B cell

IVW = inverse variance weighting, nSNP = number of single-nucleotide polymorphism.

### 3.2. Causal link between immune cells and MN

**Figure 2.**
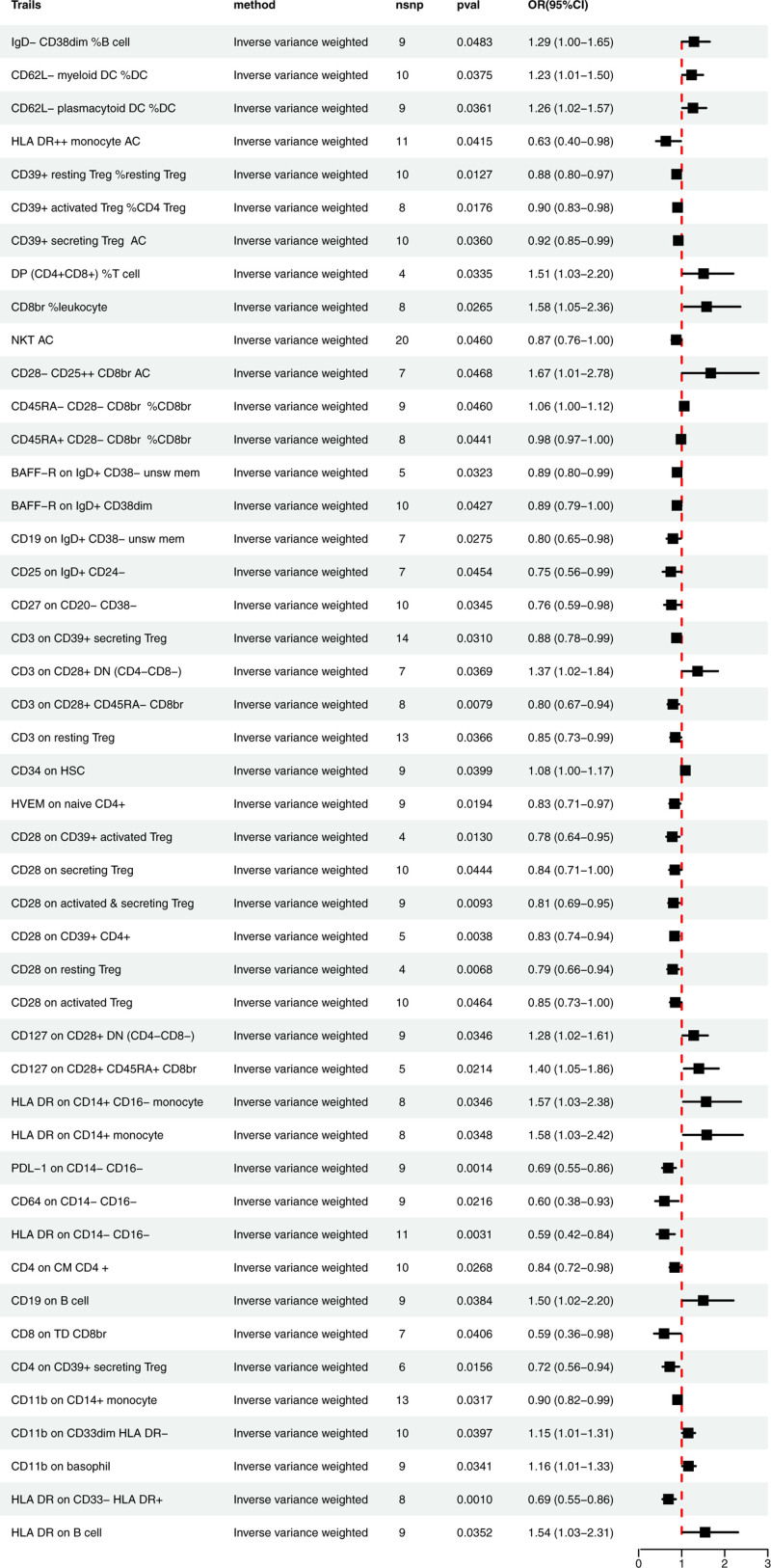
Results of the forward Mendelian randomization analysis (IVW). IVW = inverse variance weighting.

The findings from the Genetic Prediction IVW method assessing the relationship between immune cell traits and MN are illustrated in Figure 2, which includes 46 immune cell traits in total. Of these, 17 traits were significantly positively associated with the occurrence of MN (OR > 1, *P* < .05). A total of 6 cell panels of these traits were identified. B cell panel: IgD- CD38dim %B cell; monocyte panel: HLA DR on CD14 + monocyte, HLA DR on CD14 + CD16- monocyte; myeloid cell panel: CD11b on basophil, CD11b on CD33dim HLA DR-, CD34 on HSC; TBNK panel: HLA DR on B cell, DP (CD4 + CD8+) %T cell, CD8br %leukocyte, CD19 on B cell; Treg panel: CD28- CD25++ CD8br AC, CD127 on CD28 + CD45RA + CD8br, CD127 on CD28 + DN (CD4-CD8-), CD45RA- CD28- CD8br %CD8br, CD3 on CD28 + DN (CD4-CD8-);cDC panel: CD62L- myeloid DC %DC,CD62L- plasmacytoid DC %DC.

In contrast, 29 immune cell traits were found to be inversely associated with MN incidence, indicating a protective role in the development of the disease. There are also 6 cell panels, although with some variations. B cell panel: CD25 on IgD + CD24-, CD27 on CD20- CD38-, CD19 on IgD + CD38- unsw mem, BAFF-R on IgD + CD38- unsw mem, BAFF-R on IgD + CD38dim; maturation stages of T cell: CD8 on TD CD8br, HVEM on naive CD4+, CD4 on CM CD4 +; monocyte panel: HLA DR on CD14- CD16-, CD64 on CD14- CD16-, PDL-1 on CD14- CD16-; myeloid cell panel: HLA DR On CD33- HLA DR+, CD11b on CD14 + monocyte; TBNK panel: HLA DR++ monocyte AC, NKT AC; Treg panel: CD3 on CD39 + secreting Treg, CD39 + resting Treg %resting Treg, CD39 + activated Treg %CD4 Treg, CD4 on CD39 + secreting Treg, CD28 on CD39 + activated Treg, CD28 on CD39 + CD4+, CD39 + secreting Treg AC, CD45RA + CD28- CD8br %CD8br, CD3 on CD28 + CD45RA- CD8br, CD3 on resting Treg, CD28 on secreting Treg, CD28 on activated & secreting Treg, CD28 on resting Treg, CD28 on activated Treg.

### 3.3. Forward sensitivity analyses

Sensitivity analyses revealed that none of the 46 immune cell subtypes included in the MR study of MN showed evidence of horizontal pleiotropy (MR-Egger intercept *P* > .05). These findings support the credibility of the causally robust results obtained. Additionally, both the leave-one-out analysis and funnel plots confirmed the stability of the data, indicating that the findings were free from substantial bias. Detailed information on the sensitivity analyses is provided in Table S1, Supplemental Digital Content, https://links.lww.com/MD/P133 and File S1, Supplemental Digital Content, https://links.lww.com/MD/P132.

### 3.4. Selection of reverse instrumental variables

In this study, GWAS data on MN were used to identify instrumental variables, all of which exhibited F-values >10, confirming the absence of weak instrument bias. Detailed information regarding the selected instrumental variables can be found in Table 2.

**Table 2:** SNPs for all positive results in reverse analyses.

ID	nSNP	*P* val (IVW)	Traits
GCST90001595	21	.00832	DP (CD4 + CD8+) %T cell
GCST90001728	31	.002504	CD19 on IgD + CD38- unsw mem
GCST90001779	31	.025985	CD25 on IgD + CD24-
GCST90001886	31	.03861	CD28 on CD39 + activated Treg
GCST90002009	31	.009977	HLA DR on CD14- CD16-
GCST90002112	31	.020061	HLA DR on CD33- HLA DR+

IVW = inverse variance weighting, nSNP = number of single-nucleotide polymorphism.

### 3.5. Causal link between MN and immune cells

**Figure 3.**
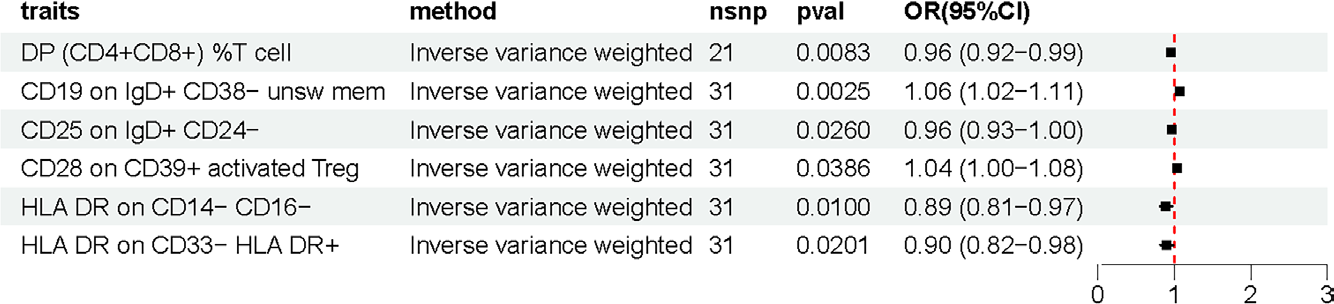
Reverse Mendelian randomization analysis results.

The outcomes of the Genetic Prediction IVW method for immune cells in MN are shown in Figure 3, which presents 6 immune cell traits achieving statistical significance (*P* < .05). Among these, 2 immune cell traits demonstrated a positive association with the development of MN, as evidenced by OR >1. Treg panel: CD28 on CD39 + activated Treg, B cell panel: CD19 on IgD + CD38- unsw mem. The remaining 4 traits were negatively correlated with the incidence of MN. Monocyte panel: HLA DR on CD14- CD16-; Myeloid cell panel: HLA DR on CD33- HLA DR+; B cell panel: CD25 on IgD + CD24-; TBNK panel: DP (CD4 + CD8+) %T cell.

### 3.6. Reverse sensitivity analyses

Rigorous sensitivity analyses can effectively exclude bias arising from genetic pleiotropy, thereby enhancing the reliability of the results. The Cochran Q test and MR-Egger intercept method were employed, avoiding presence of heterogeneity and horizontal pleiotropy among the instrumental variables for MN. Furthermore, sensitivity analyses using the leave-one-out method, along with funnel plots, demonstrated that the data were stable and free from substantial bias. Detailed information on the sensitivity analyses is provided in Table S2, Supplemental Digital Content, https://links.lww.com/MD/P134 and File S2, Supplemental Digital Content, https://links.lww.com/MD/P135.

## 4. Discussion

This study represented a pioneering large-scale MR analysis designed to elucidate the genetic causal relationships between immune cell subtypes and MN. Our findings supported previous exploratory cohort studies, underscoring a causal association between specific immune cell subtypes and MN. Forward MR analysis identified 46 immunocyte subtypes, spanning B cells, Maturation stages of T cells, monocytes, myeloid cells, TBNK, Treg, and cDC panels, as potential contributors or inhibitors to MN. It was notable that 6 immunocyte subtypes contained in forward analysis served bidirectional causal effects, in reverse analysis.

This study identified 17 immune cell subtypes that showed a positive association with increased MN risk. Advances in the immunopathogenesis of MN, particularly the discovery of disease-associated autoantigens and autoantibodies, have transitioned therapeutic strategies from nonspecific immunosuppression to targeted B-cell therapies.^[30]^ Consistent with this shift, prior studies have highlighted several of the immune subtypes we identified. For instance, IgD- CD38dim %B cells have been proposed as cellular biomarkers for MN, given their potential role in the pathogenesis and in monitoring of anti-PLA2R1-associated MN.^[31]^ CD34, a stem cell marker, has been implicated in MN pathogenesis by facilitating the mobilization and migration of hematopoietic progenitors.^[32–34]^ Clinical control studies by Deng et al identified a positive association between CD19 expression on B cells and MN, suggesting a pathogenic role via the promotion of pro-inflammatory cytokines.^[35]^ Zhao Cui et al investigated the genetic profiles of 261 idiopathic MN patients and 599 matched controls, revealing an association between HLA class II genes, particularly HLA DR, and MN developmen.^[36]^ Apart from the 4 aforementioned immune cell subtypes, the remaining 13 have not been directly investigated in MN, which may represent potential biomarkers or therapeutic targets for MN.

Notably, these novel signals encompass both innate and adaptive immunity. For instance, we identified associations with monocyte and myeloid cell populations, including elevated HLA DR expression on classical monocytes, alongside CD11b + basophils and CD33dim HLA DR- myeloid-derived cells. Our analysis also highlighted dendritic cell subsets lacking the lymph node–homing receptor CD62L. On the adaptive immunity front, our findings point to unconventional T cell subsets, such as circulating CD4 + CD8 + double-positive T cells and highly differentiated CD8 + T cells marked by the loss of CD28 and CD45RA expression. Additionally, we noted associations with distinct regulatory T cell populations defined by markers like CD25, CD28, CD127, and CD39. Strikingly, none of these specific cell subsets have been previously tied to MN, hinting at previously unrecognized immunopathological pathways. These discoveries not only propose new candidates for MN biomarkers or therapeutic targets but also emphasize the need for deeper mechanistic studies to clarify their roles in disease progression.

Our MR analysis identified negative associations between 29 immunocyte subtypes and MN, offering novel insights into potential therapeutic strategies. BAFF is crucial for B cell maturation, supporting peripheral B cell activation, survival, and differentiation. Its interaction with BAFF-R is essential for B-cell homeostasis.^[37]^Chee et al^[38]^ suggested BAFF inhibition as a therapeutic approach in IgA nephropathy, highlighting BAFF-R as a risk factor, though its role in MN remains unexplored. Consistent with our findings, depletion of CD19 + B cells is an effective MN treatment. For instance, rituximab, an anti-CD20 monoclonal antibody targeting CD19 + B cells, induces remission in many MN patients.^[31]^ The study by Zhao et al demonstrated that CD25 and CD27 were involved in the secretion of IgG4 by Br1 cells, which is associated with the pathogenesis of MN.^[39]^ Studies have shown that the HVEM signaling pathway was essential in controlling T cell-mediated B cell differentiation, thus inhibiting excessive immune activation.^[40,41]^ Accordingly, HVEM on effector memory CD4 + T cells appear protective in MN.^[15]^ Our study further identified protective roles for other subtypes, such as CD14 + monocytes expressing CD11b, though conflicting reports suggest elevated CD11b in treated MN patients, warranting further investigation.^[42]^ Although direct evidence linking many of these subtypes to MN is limited, our results align with MN pathogenesis that autoantibodies (against phospholipase A2 receptor) drive CD4 + T cell activation and B-cell differentiation into plasma cells, producing pathogenic IgG4.^[43,44]^ CD4 + T cells orchestrate this autoimmune response by fostering an inflammatory milieu that amplifies autoreactive B-cell activity.^[10]^ Conversely, regulatory mechanisms, such as CD39 + regulatory T cells producing immunosuppressive adenosine or CD14-CD16- monocytes expressing PDL-1^,[45,46]^, counteract Th1/Th17-driven inflammation.^[47]^ This delicate balance between effector and regulatory immune responses shapes MN’s clinical course. These findings highlight previously unrecognized immunopathological pathways, underscoring the innovation of this study in identifying novel therapeutic targets for MN.

In addition to the forward MR findings, we observed 6 subtypes that exhibited significant reverse causal associations. Three subtypes, CD25 on IgD + CD24- B cells, HLA DR on CD14- CD16- monocytes, HLA DR on CD33- HLA DR + cells, exhibited consistent bidirectional causality, suggesting a robust reciprocal relationship with MN. For instance, CD25 on IgD + CD24- B cells may modulate B-cell activation, positioning it as a potential diagnostic biomarker, while HLA DR-expressing monocytes could serve as targets for anti-inflammatory therapies in MN management. These overlapping subtypes hold promise as biomarkers or therapeutic targets. In contrast, the other 3 subtypes, CD19 on IgD + CD38- unswitched memory B cells, CD4 + CD8 + double-positive T cells, and CD28 on CD39 + activated regulatory T cells, showed asymmetrical causal relationships, indicating complex feedback mechanisms. For example, CD19 on unswitched memory B cells may reflect a compensatory immune response in MN, warranting further mechanistic studies. These intricate interactions highlight the dynamic interplay between immune cells and MN, paving the way for precision immunotherapy and deeper investigations into their clinical applications.

Our conclusions were drawn from genetic evidence using comprehensive MR analyses, which strengthen the causal inference and validation. Consequently, the results are not likely influenced by heterogeneity, horizontal pleiotropy, or confounding variables. However, several limitations should be acknowledged. First, we selected instrumental variables using a relaxed threshold of *P* < 1 × 10^−5^, which, while enabling a broader assessment of the associations between immune cell subtypes and MN, may result in weaker instrument strength. Second, a key limitation is that our dataset is exclusively derived from European populations, which restricts the generalizability of the findings. Genetic associations can vary significantly across ethnic groups due to differences in allele frequencies, linkage disequilibrium patterns, and environmental exposures. As a result, the causal relationships identified here may not hold in non-European populations, and caution is warranted when extrapolating these findings beyond European cohorts. To overcome this limitation, future studies should replicate these analyses in diverse populations to confirm the broader applicability of our results. Third, although no evidence of horizontal pleiotropy was found for all instrumental variables, we permitted a small number of instrumental variables to exhibit heterogeneity to ensure the completeness of the results. This approach may introduce variability in the causal estimates, which should be carefully considered when interpreting the findings.

Furthermore, the two-sample MR method faces inherent limitations in analyzing multiple exposures, such as its inability to account for correlations between exposures, potentially affecting the reliability of the results. There is a need for further development of suitable analytical approaches. To translate these findings into clinical practice, comprehensive clinical trials are essential to validate the results. To further elucidate the associations between immune cell subtypes and MN, as well as the underlying mechanisms, future studies require more extensive GWAS datasets and advanced analytical methods or experimental validations.

## 5. Conclusion

In conclusion, this comprehensive bidirectional two-sample MR analysis establishes causal relationships between specific immune cell subtypes and MN, underscoring the immune system’s complex involvement in MN pathogenesis. These findings not only advance our immunological understanding of MN but also carry direct translational implications. Identifying immune cell subtypes with causal roles may inform clinical decision-making, which could facilitate earlier diagnosis through immune-based biomarkers, and highlight novel targets for MN treatment. Ultimately, this work provides a foundation for translating these insights into improved prevention and management strategies for MN.

## Acknowledgments

We would like to express our gratitude to the participants and researchers involved in the GWAS datasets utilized in this study.

## Author contributions

**Conceptualization:** Yiyang Yu, Zhongqiu Luan.

**Data curation:** Yiyang Yu, Guangxu Liu, Rui Zhang, Baojiang Chen.

**Formal analysis:** Yiyang Yu, Guangxu Liu, Rui Zhang, Baojiang Chen, Zhongqiu Luan.

**Funding acquisition:** Zhongqiu Luan.

**Investigation:** Yiyang Yu, Guangxu Liu, Baojiang Chen.

**Methodology:** Yiyang Yu, Guangxu Liu, Baojiang Chen.

**Software:** Yiyang Yu, Guangxu Liu.

**Supervision:** Yiyang Yu, Guangxu Liu, Rui Zhang, Baojiang Chen, Zhongqiu Luan.

**Validation:** Guangxu Liu, Rui Zhang, Zhongqiu Luan.

**Visualization:** Rui Zhang.

**Writing – original draft:** Yiyang Yu, Rui Zhang, Baojiang Chen.

**Writing – review & editing:** Yiyang Yu, Baojiang Chen, Zhongqiu Luan.

## Supplementary Material


