# Supplementary material for: Association between immune cell subtypes and membranous nephropathy: A bidirectional Mendelian randomization study
Source: Medicine (Baltimore). 2025 Jun 6;104(23):e42774. doi: 10.1097/MD.0000000000042774 (PMC12151028; doi:10.1097/MD.0000000000042774)
Supplement: Supplementary file 1 [file medi-104-e42774-s001.pdf]

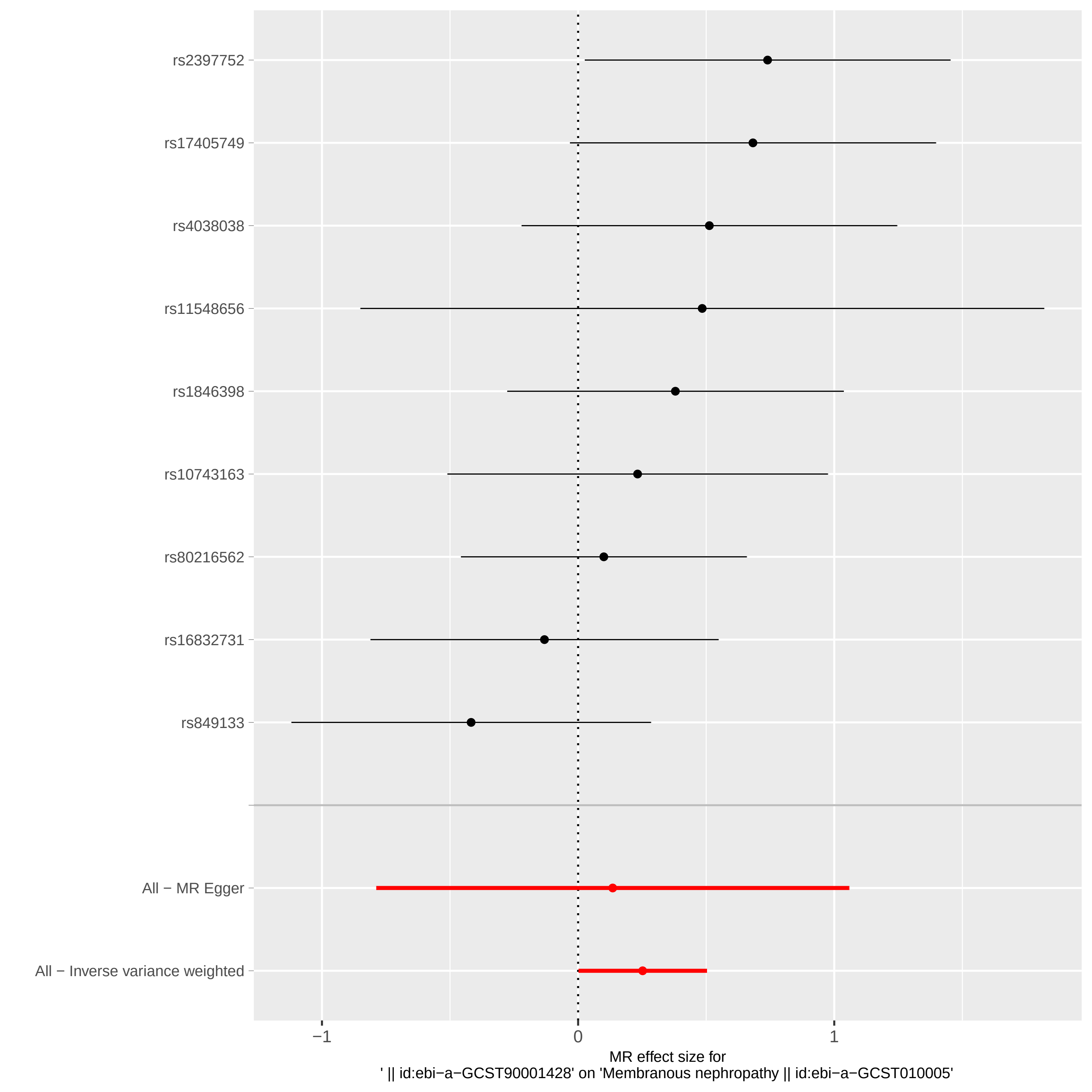

rs2397752
rs17405749
rs4038038
rs11548656
rs1846398
rs10743163
rs80216562
rs16832731
rs849133
All − MR Egger
All − Inverse variance weighted
−1
0
1
MR effect size for
' || id:ebi−a−GCST90001428' on 'Membranous nephropathy || id:ebi−a−GCST010005'

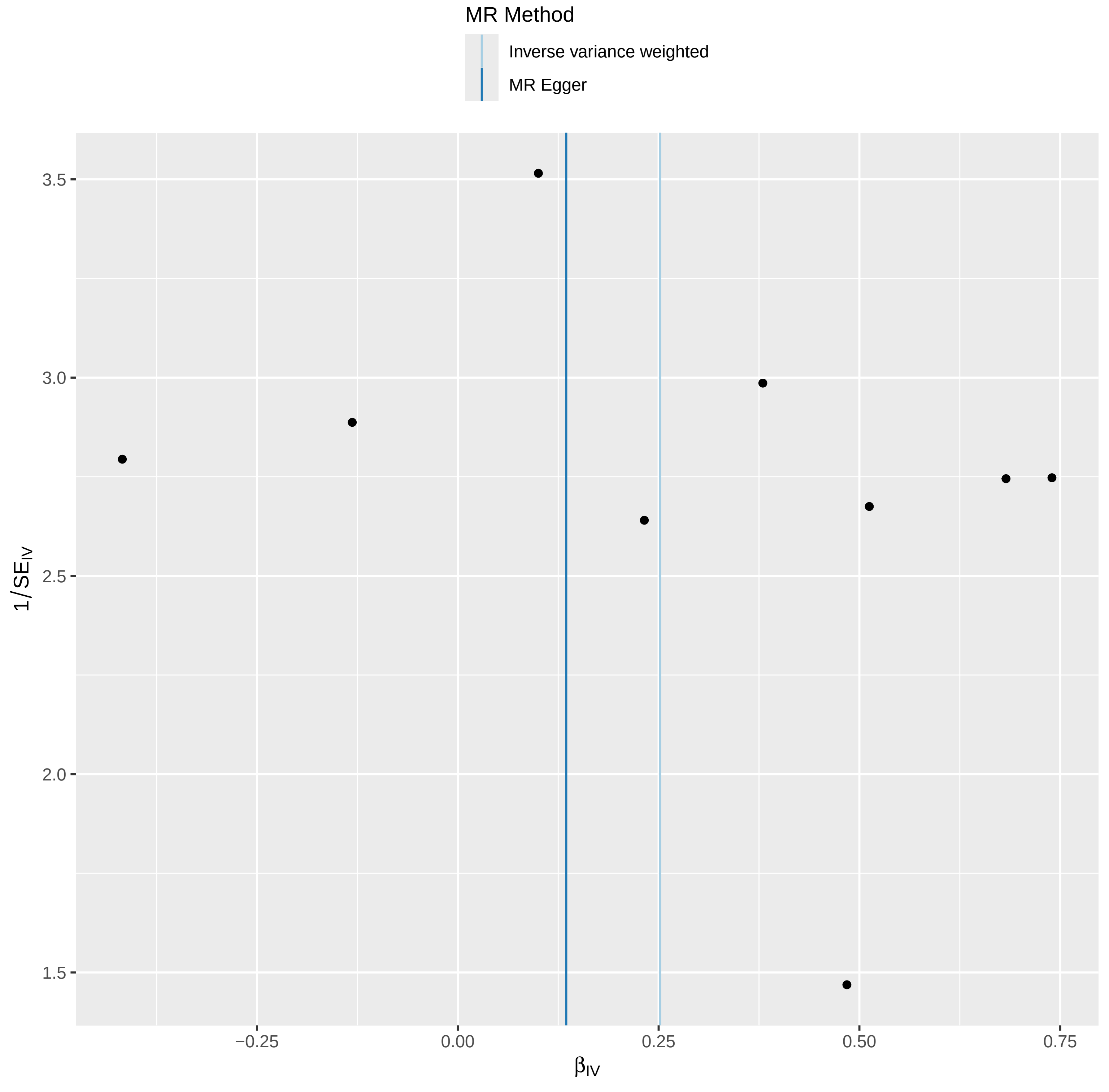

MR Method
Inverse variance weighted
MR Egger
3.5
3.0
2.5
2.0
1.5
1/SE$_{IV}$
−0.25
0.00
0.25
0.50
0.75
β$_{IV}$

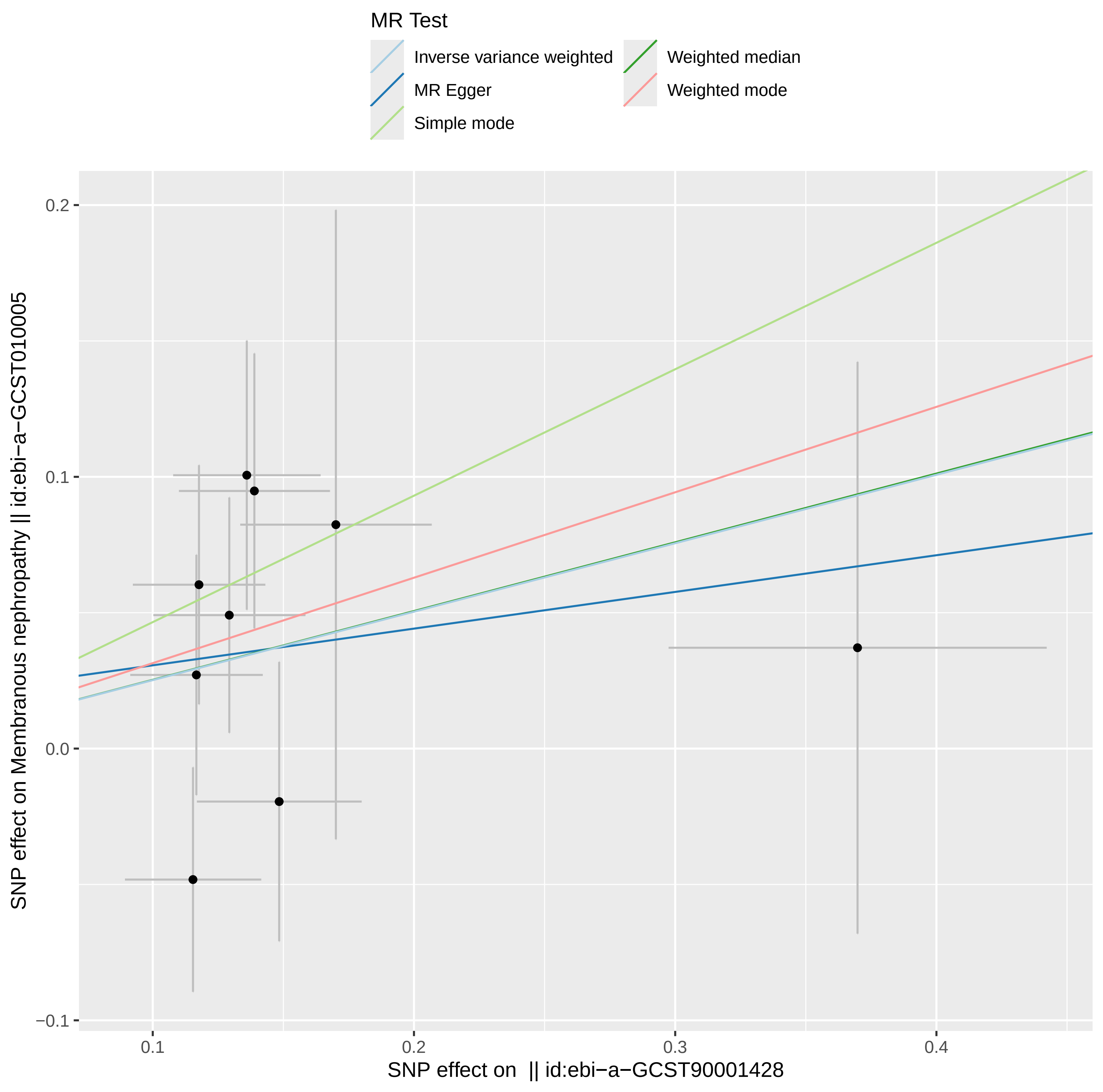
MR Test
Inverse variance weighted
MR Egger
Simple mode
Weighted median
Weighted mode
0.2
0.1
0.0
−0.1
0.1
0.2
0.3
0.4
SNP effect on Membranous nephropathy || id:ebi−a−GCST010005
SNP effect on || id:ebi−a−GCST90001428

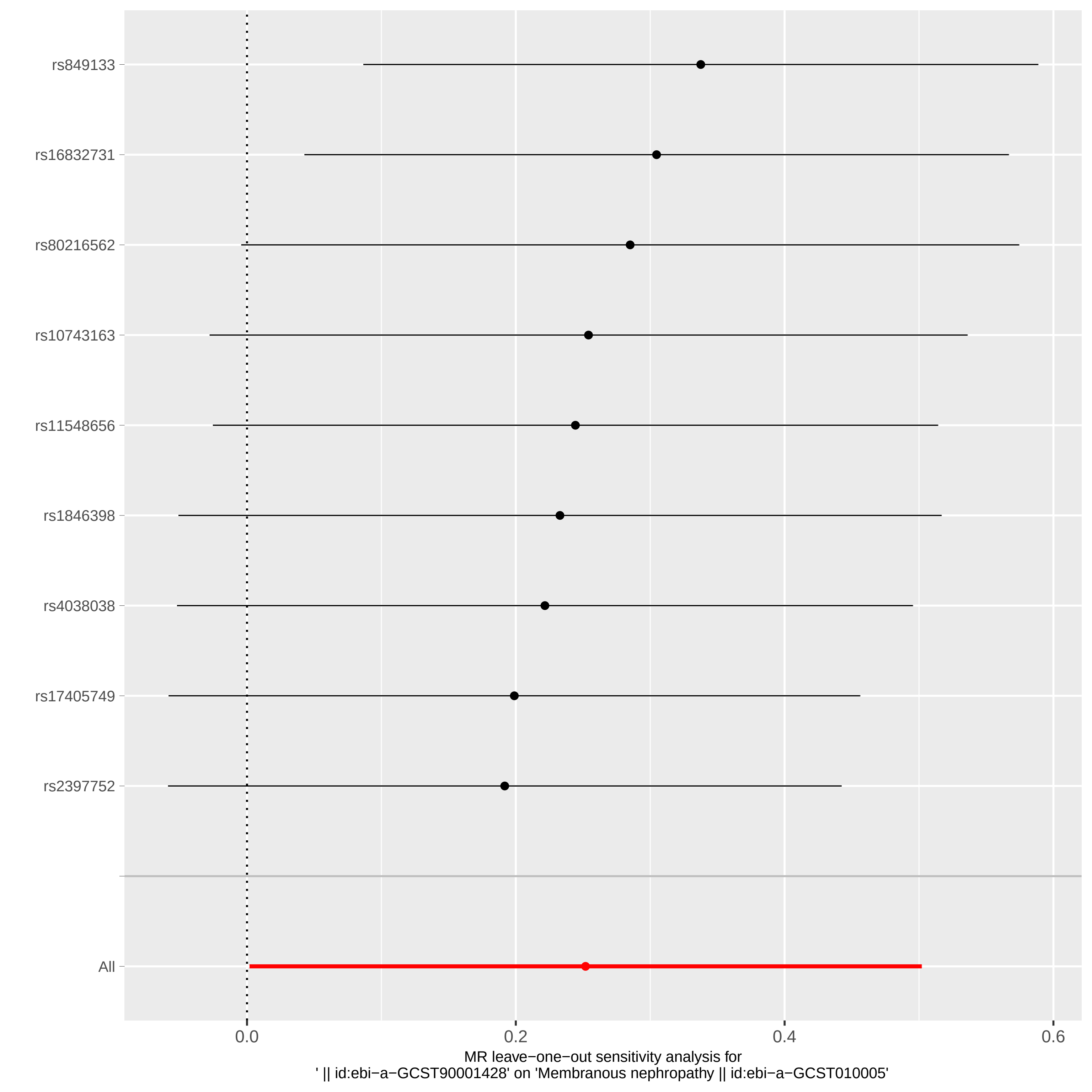
rs849133
rs16832731
rs80216562
rs10743163
rs11548656
rs1846398
rs4038038
rs17405749
rs2397752
All
0.0
0.2
0.4
0.6
MR leave−one−out sensitivity analysis for
' || id:ebi−a−GCST90001428' on 'Membranous nephropathy || id:ebi−a−GCST010005'

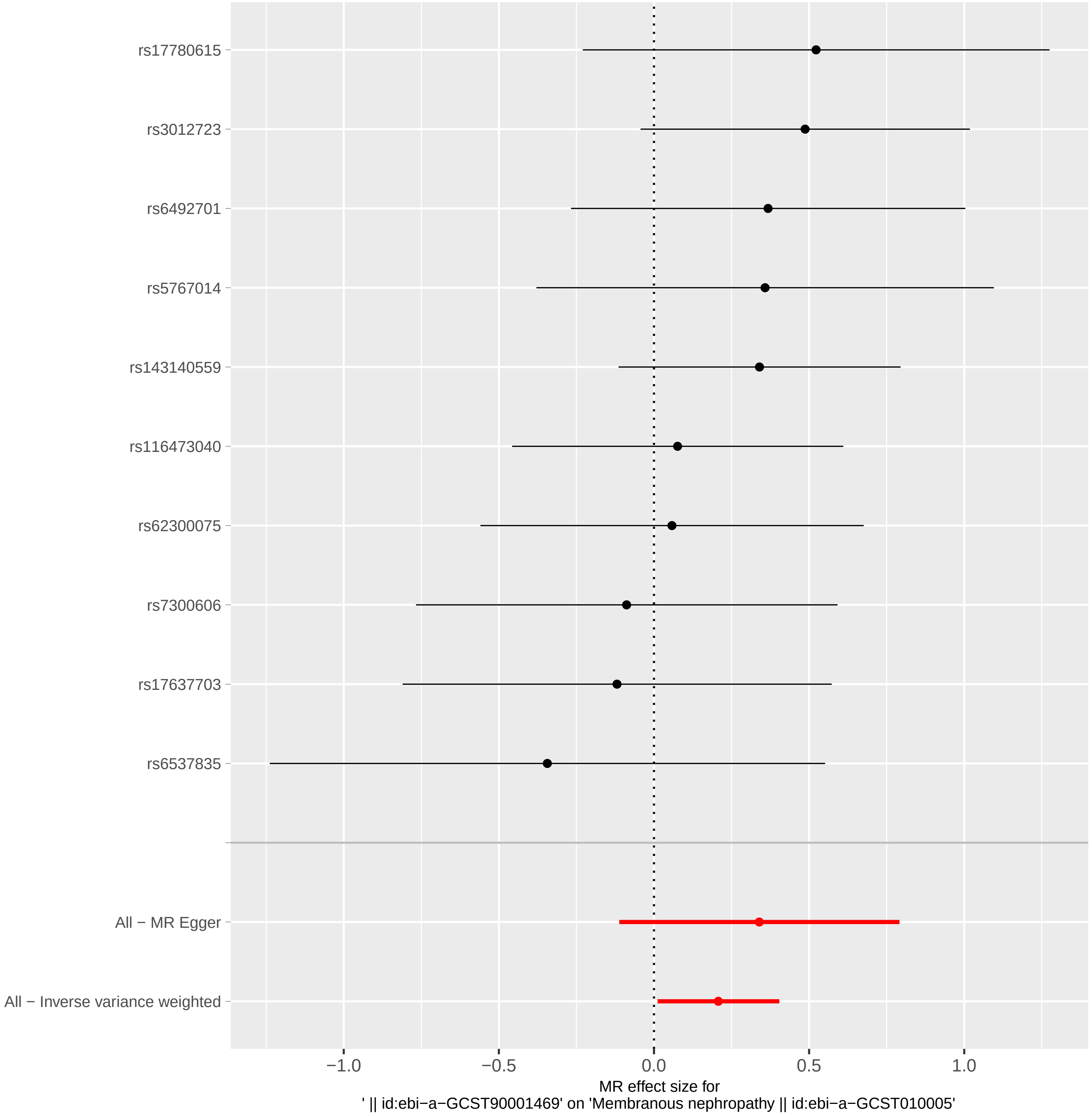
rs17780615
rs3012723
rs6492701
rs5767014
rs143140559
rs116473040
rs62300075
rs7300606
rs17637703
rs6537835
All − MR Egger
All − Inverse variance weighted
−1.0
−0.5
0.0
0.5
1.0
MR effect size for
' || id:ebi−a−GCST90001469' on 'Membranous nephropathy || id:ebi−a−GCST010005'

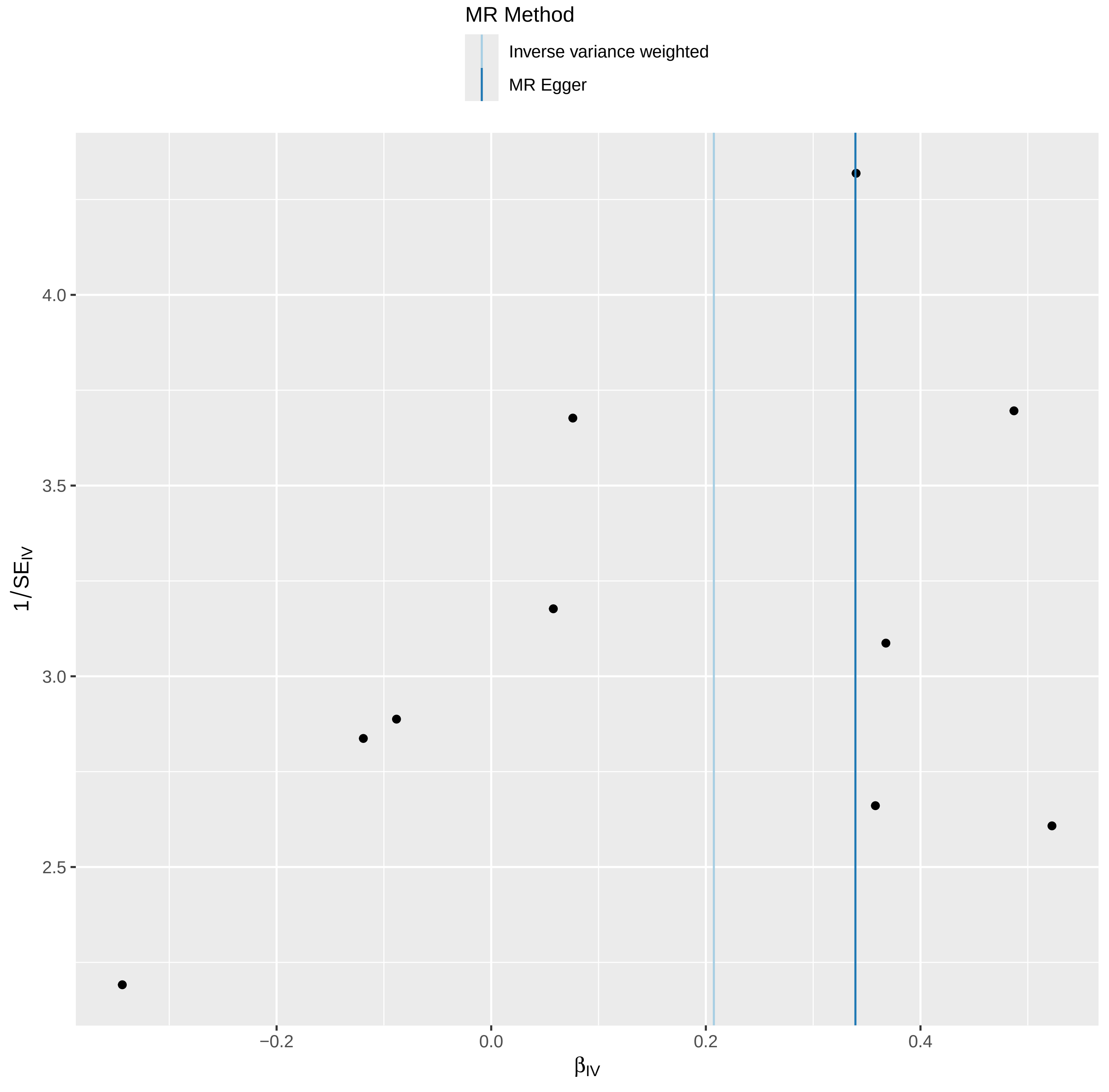

MR Method
Inverse variance weighted
MR Egger
4.0
3.5
3.0
2.5
1/SE_IV
−0.2
0.0
0.2
0.4
β_IV

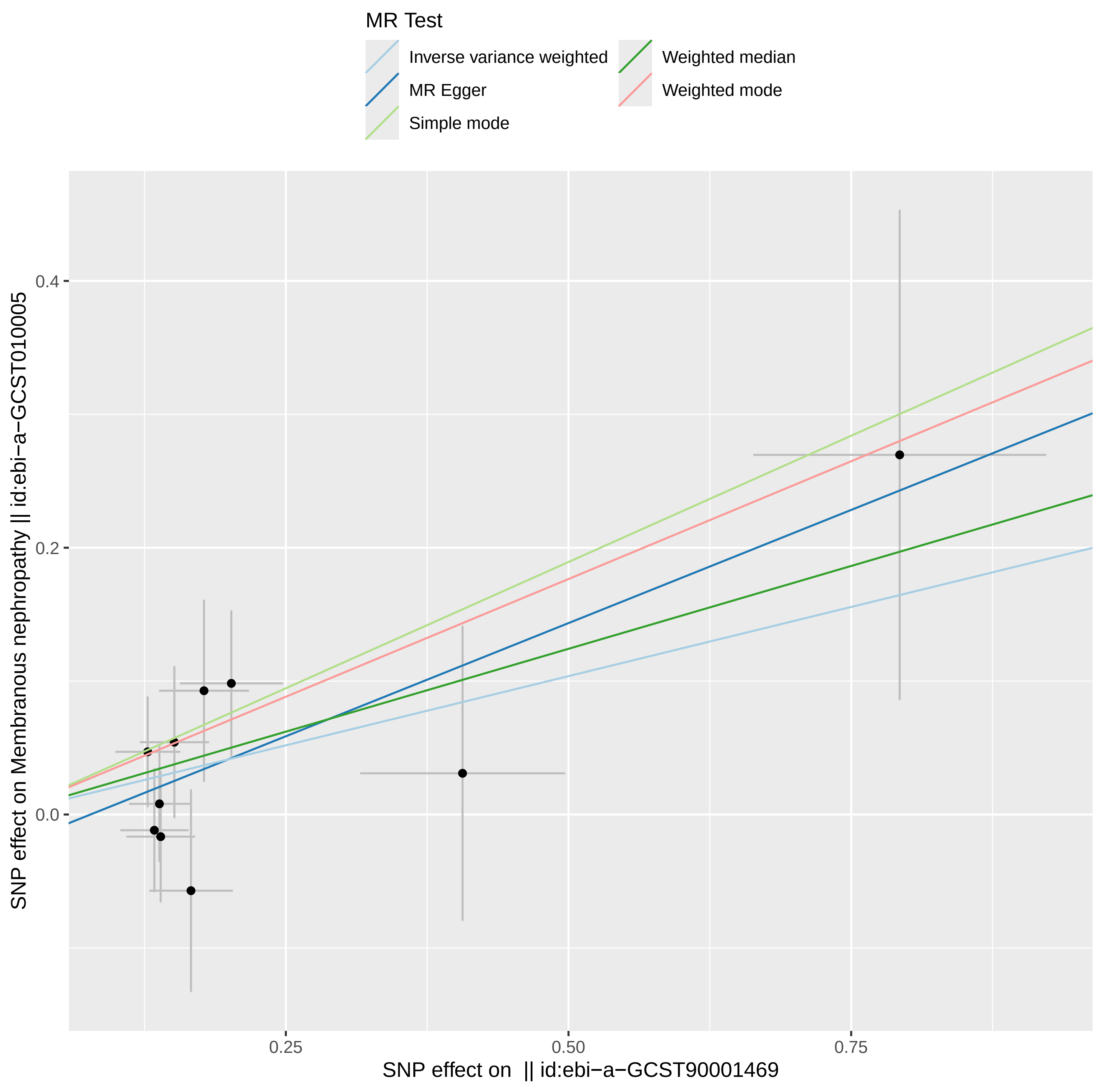
MR Test
Inverse variance weighted
MR Egger
Simple mode
Weighted median
Weighted mode
SNP effect on Membranous nephropathy || id:ebi−a−GCST010005
0.4
0.2
0.0
0.25
0.50
0.75
SNP effect on || id:ebi−a−GCST90001469

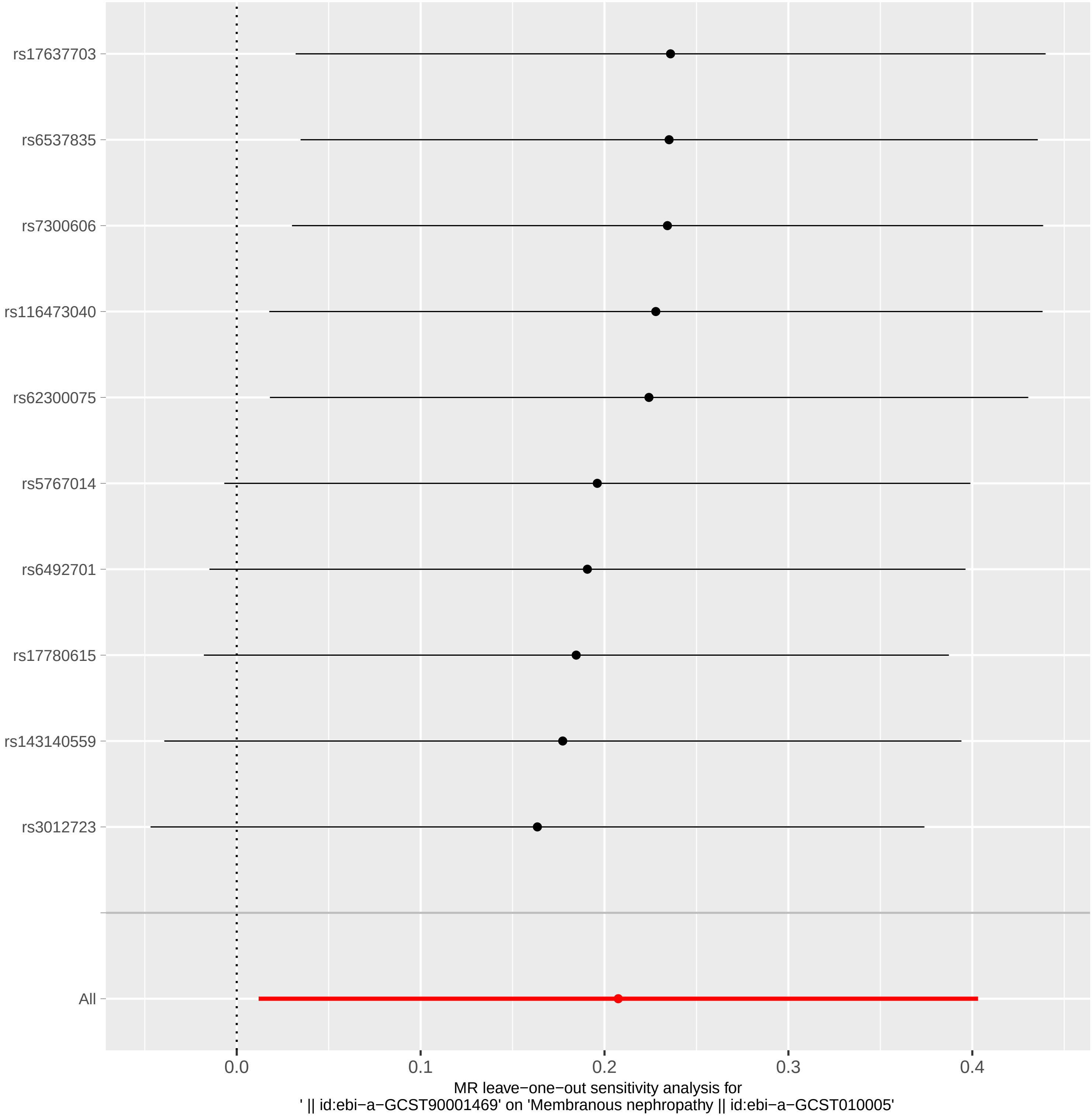

rs17637703
rs6537835
rs7300606
rs116473040
rs62300075
rs5767014
rs6492701
rs17780615
rs143140559
rs3012723
All
0.0
0.1
0.2
0.3
0.4
MR leave−one−out sensitivity analysis for
' || id:ebi−a−GCST90001469' on 'Membranous nephropathy || id:ebi−a−GCST010005'

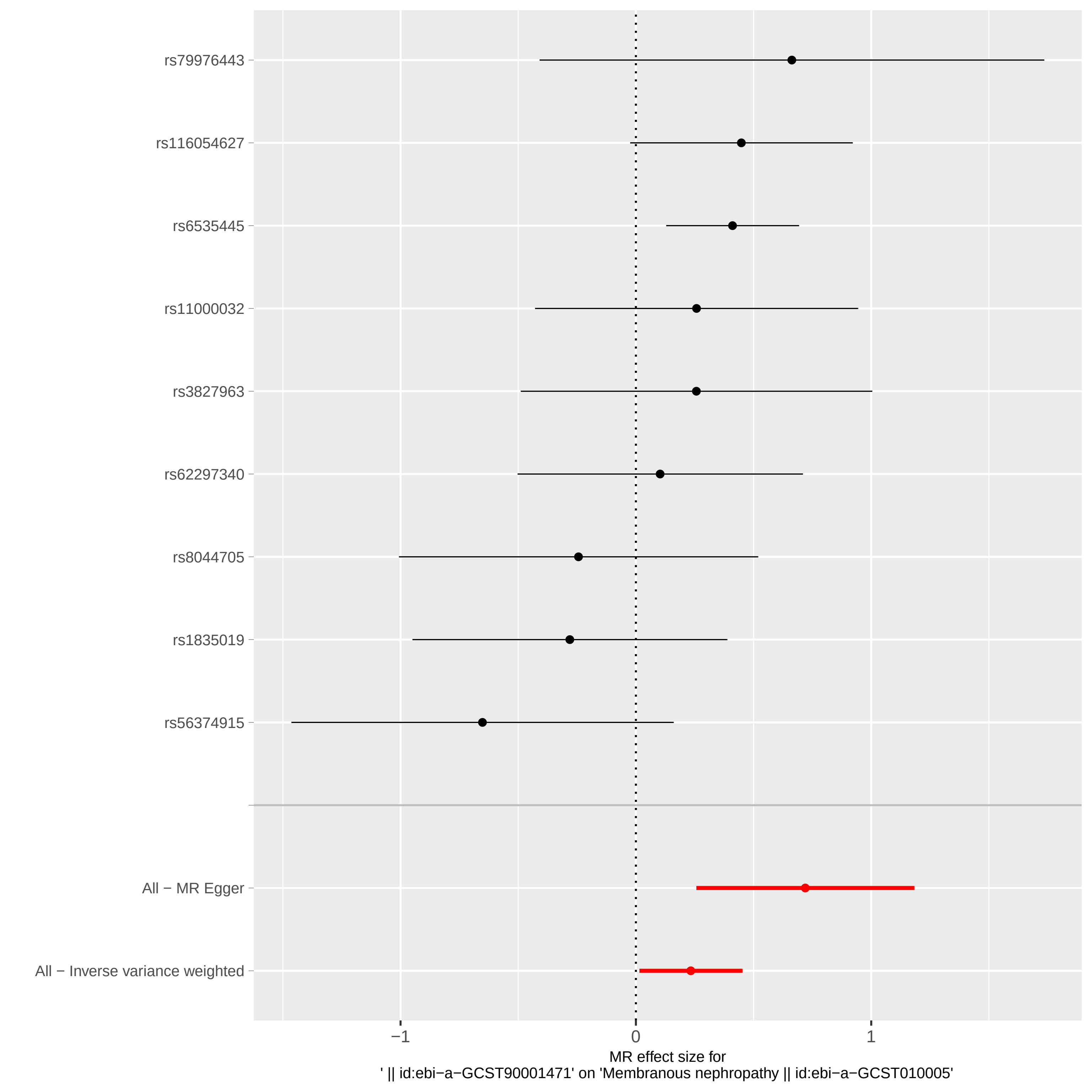

rs79976443
rs116054627
rs6535445
rs11000032
rs3827963
rs62297340
rs8044705
rs1835019
rs56374915
All − MR Egger
All − Inverse variance weighted
−1
0
1
MR effect size for
' || id:ebi−a−GCST90001471' on 'Membranous nephropathy || id:ebi−a−GCST010005'

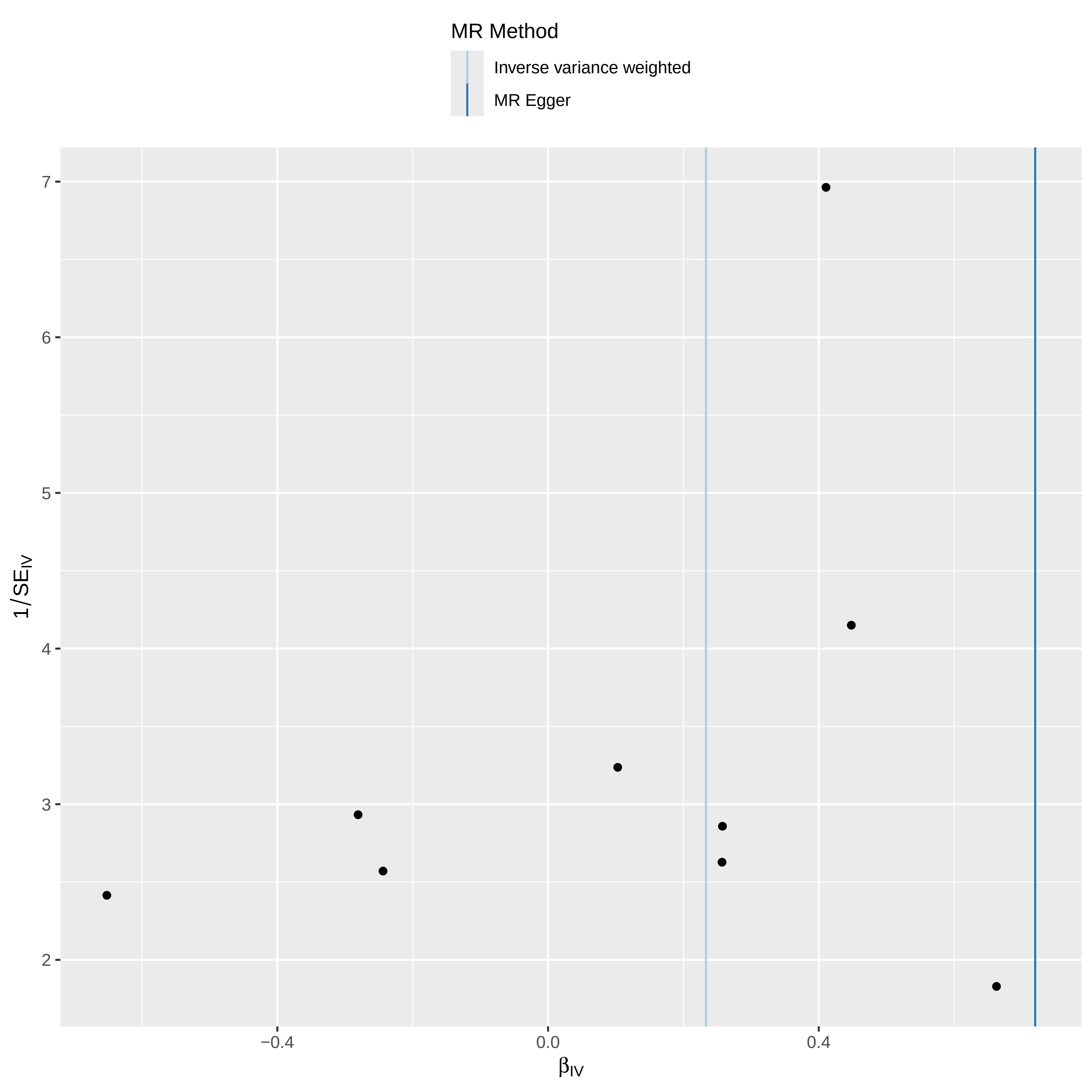

MR Method
Inverse variance weighted
MR Egger
7
6
5
4
3
2
1/SE_IV
−0.4
0.0
0.4
β_IV

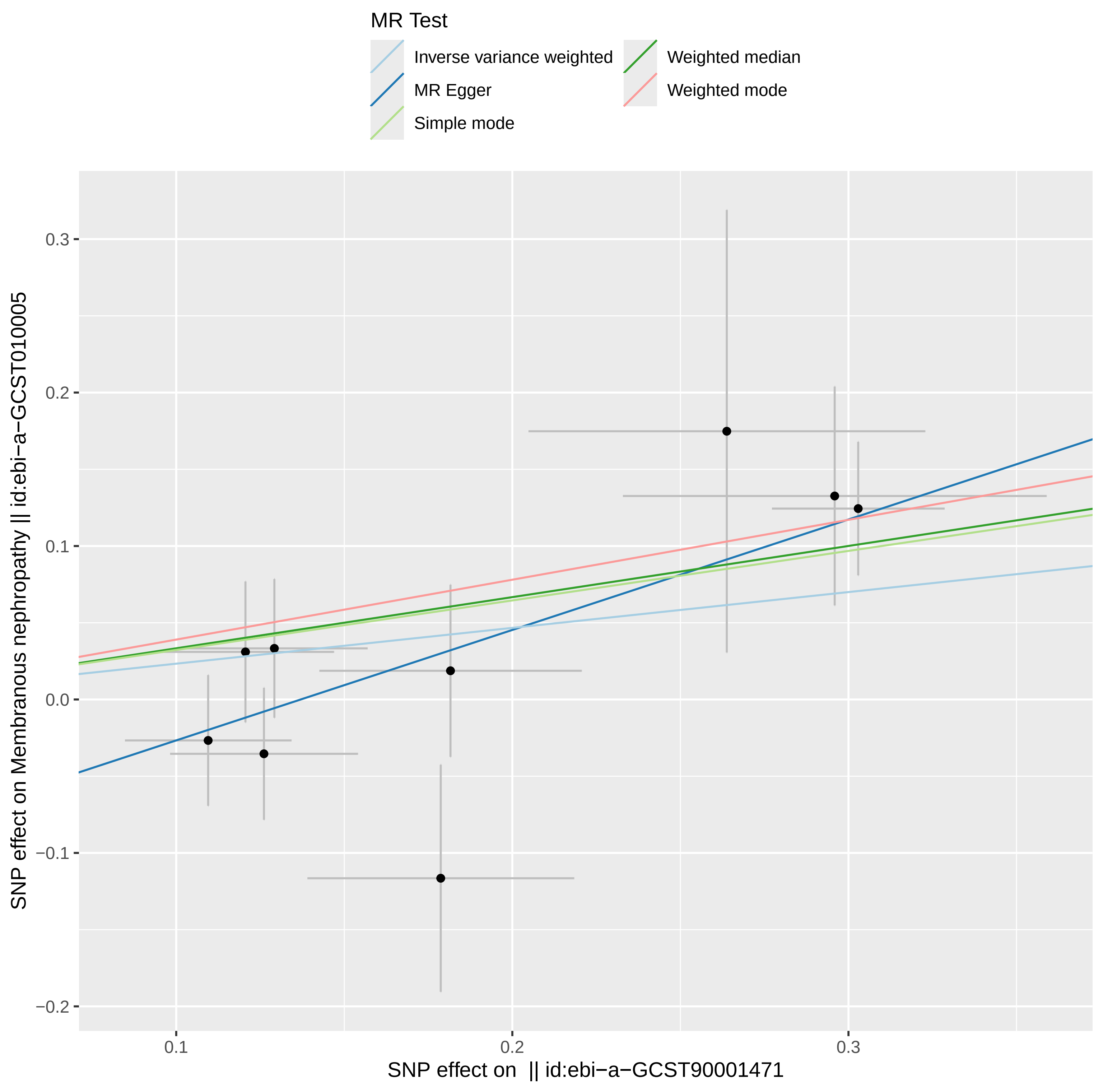
MR Test
Inverse variance weighted
MR Egger
Simple mode
Weighted median
Weighted mode
SNP effect on Membranous nephropathy || id:ebi−a−GCST010005
0.3
0.2
0.1
0.0
−0.1
−0.2
0.1
0.2
0.3
SNP effect on || id:ebi−a−GCST90001471

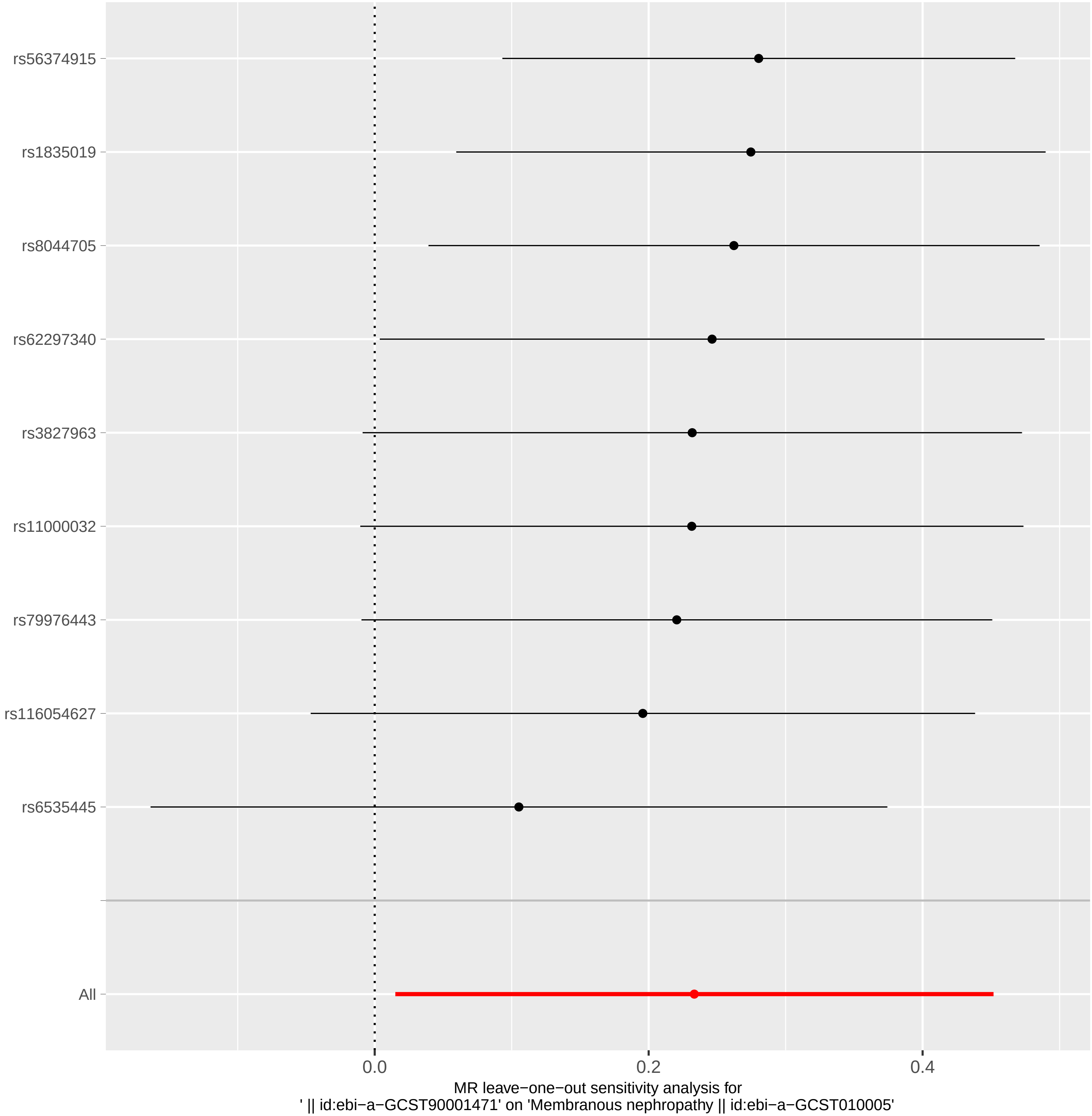
rs56374915
rs1835019
rs8044705
rs62297340
rs3827963
rs11000032
rs79976443
rs116054627
rs6535445
All
0.0
0.2
0.4
MR leave−one−out sensitivity analysis for
' || id:ebi−a−GCST90001471' on 'Membranous nephropathy || id:ebi−a−GCST010005'

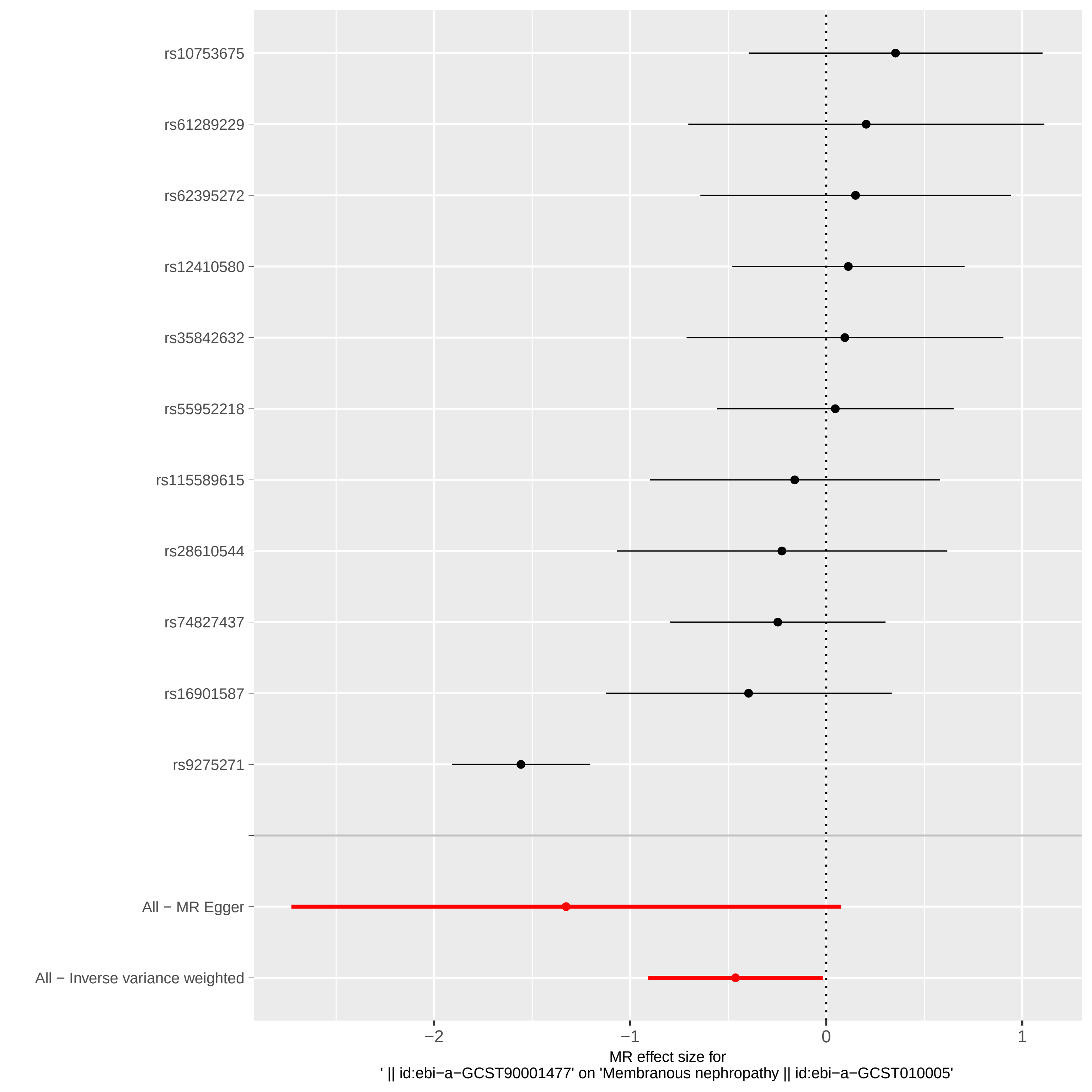
rs10753675
rs61289229
rs62395272
rs12410580
rs35842632
rs55952218
rs115589615
rs28610544
rs74827437
rs16901587
rs9275271
All − MR Egger
All − Inverse variance weighted
−2
−1
0
1
MR effect size for
' || id:ebi−a−GCST90001477' on 'Membranous nephropathy || id:ebi−a−GCST010005'

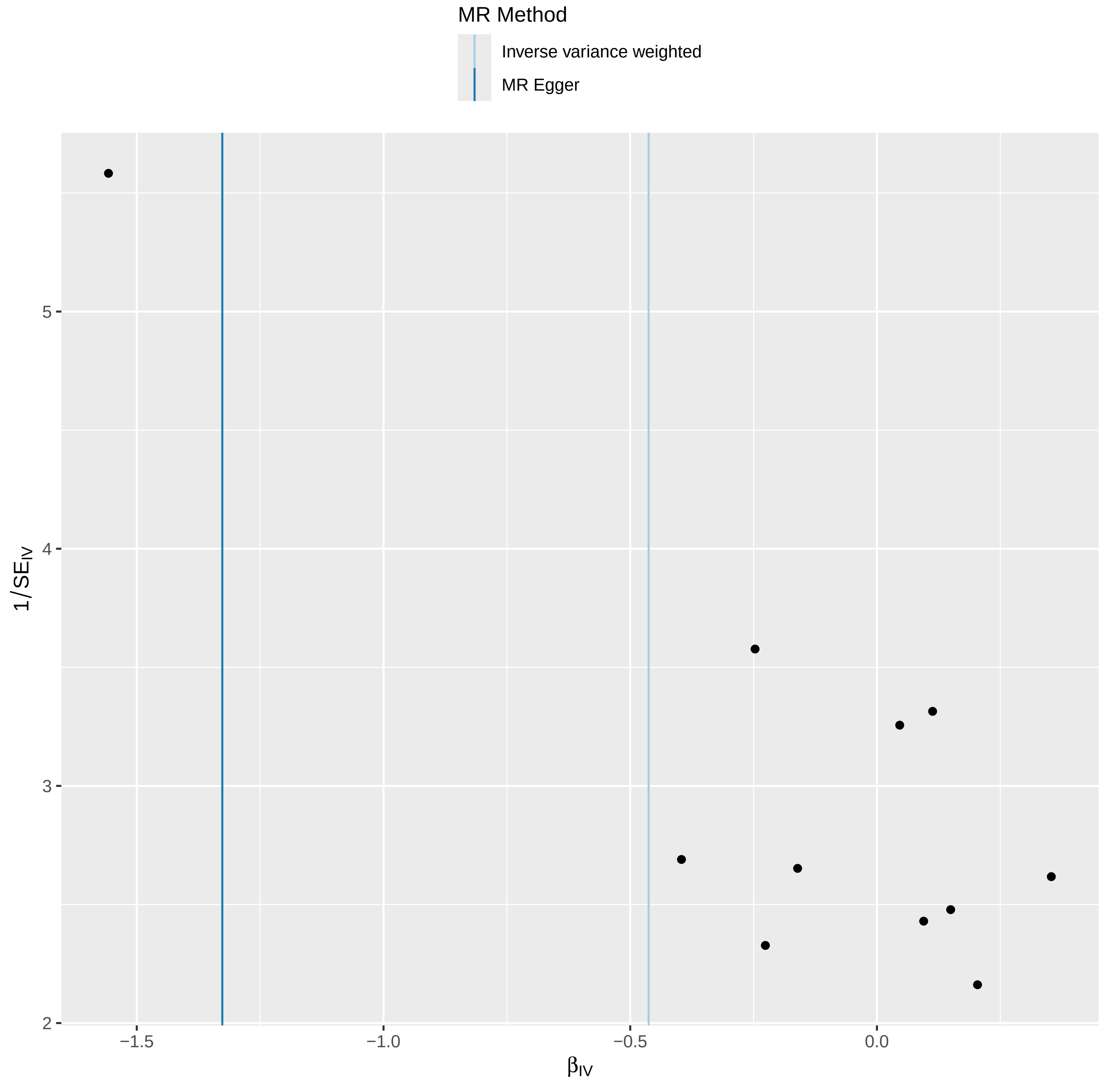
MR Method
Inverse variance weighted
MR Egger
1/SE$_{IV}$
5
4
3
2
−1.5
−1.0
−0.5
0.0
β$_{IV}$

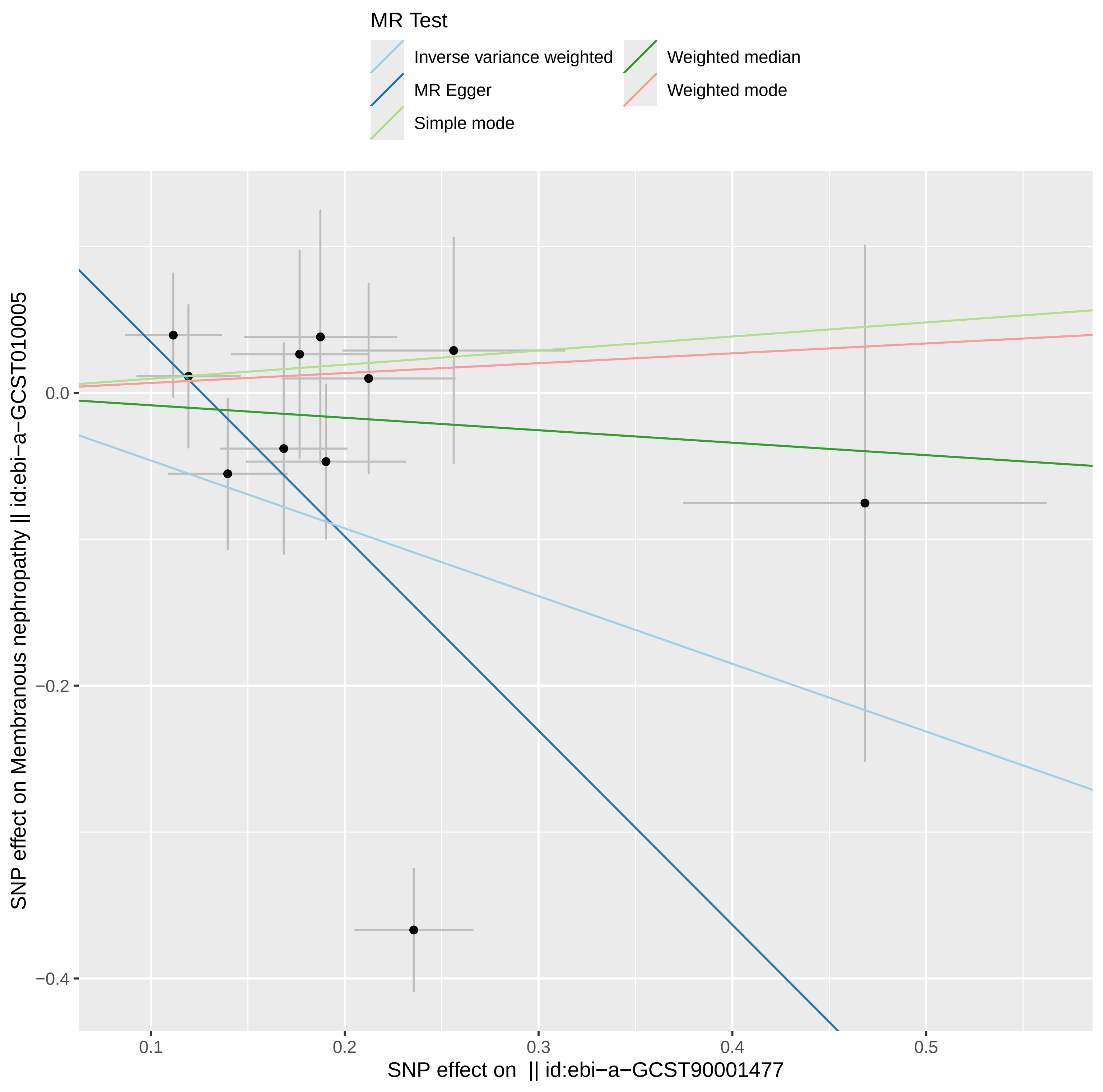

MR Test
Inverse variance weighted
MR Egger
Simple mode
Weighted median
Weighted mode
0.0
−0.2
−0.4
0.1
0.2
0.3
0.4
0.5
SNP effect on Membranous nephropathy || id:ebi−a−GCST010005
SNP effect on || id:ebi−a−GCST90001477

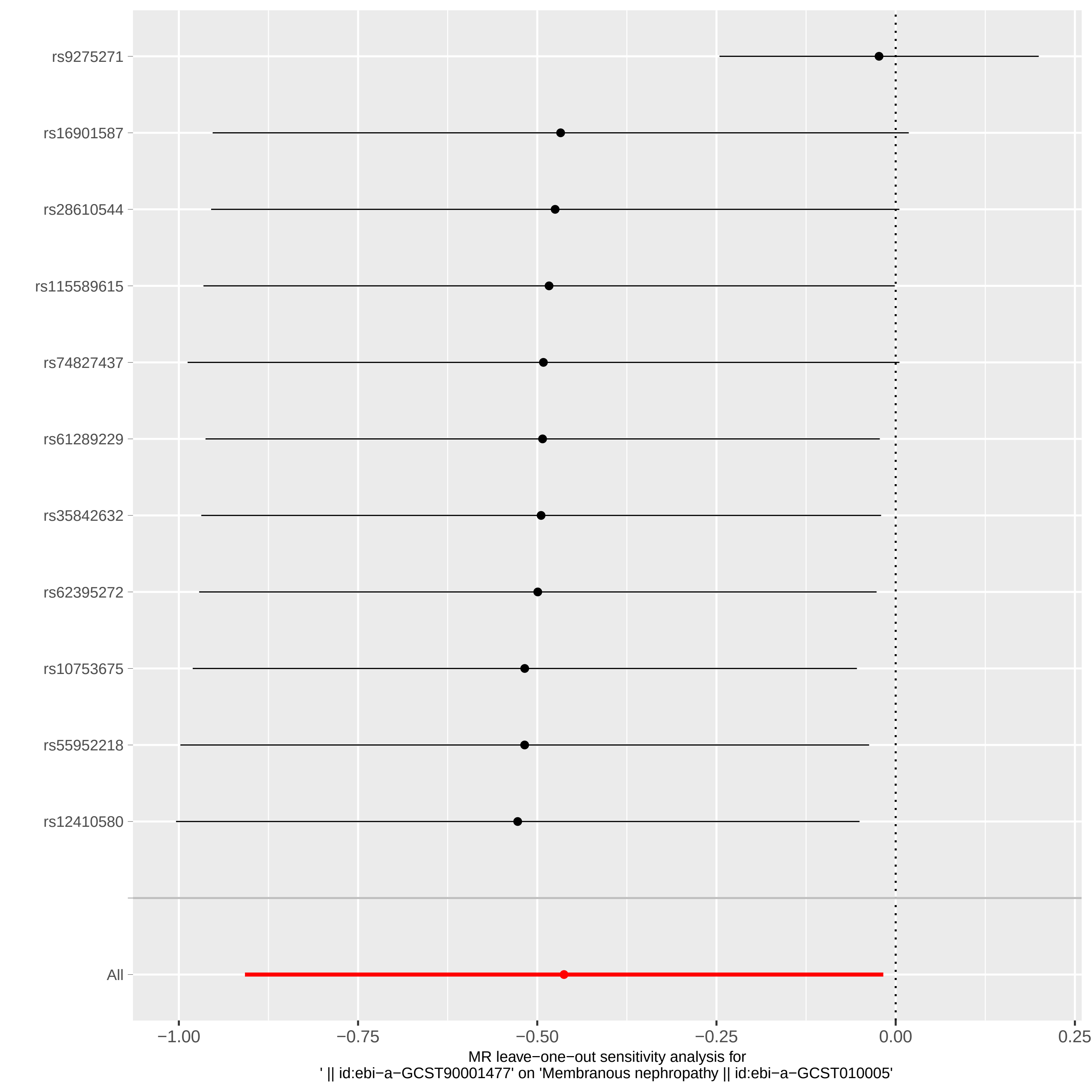

rs9275271
rs16901587
rs28610544
rs115589615
rs74827437
rs61289229
rs35842632
rs62395272
rs10753675
rs55952218
rs12410580
All
−1.00
−0.75
−0.50
−0.25
0.00
0.25
MR leave−one−out sensitivity analysis for
' || id:ebi−a−GCST90001477' on 'Membranous nephropathy || id:ebi−a−GCST010005'

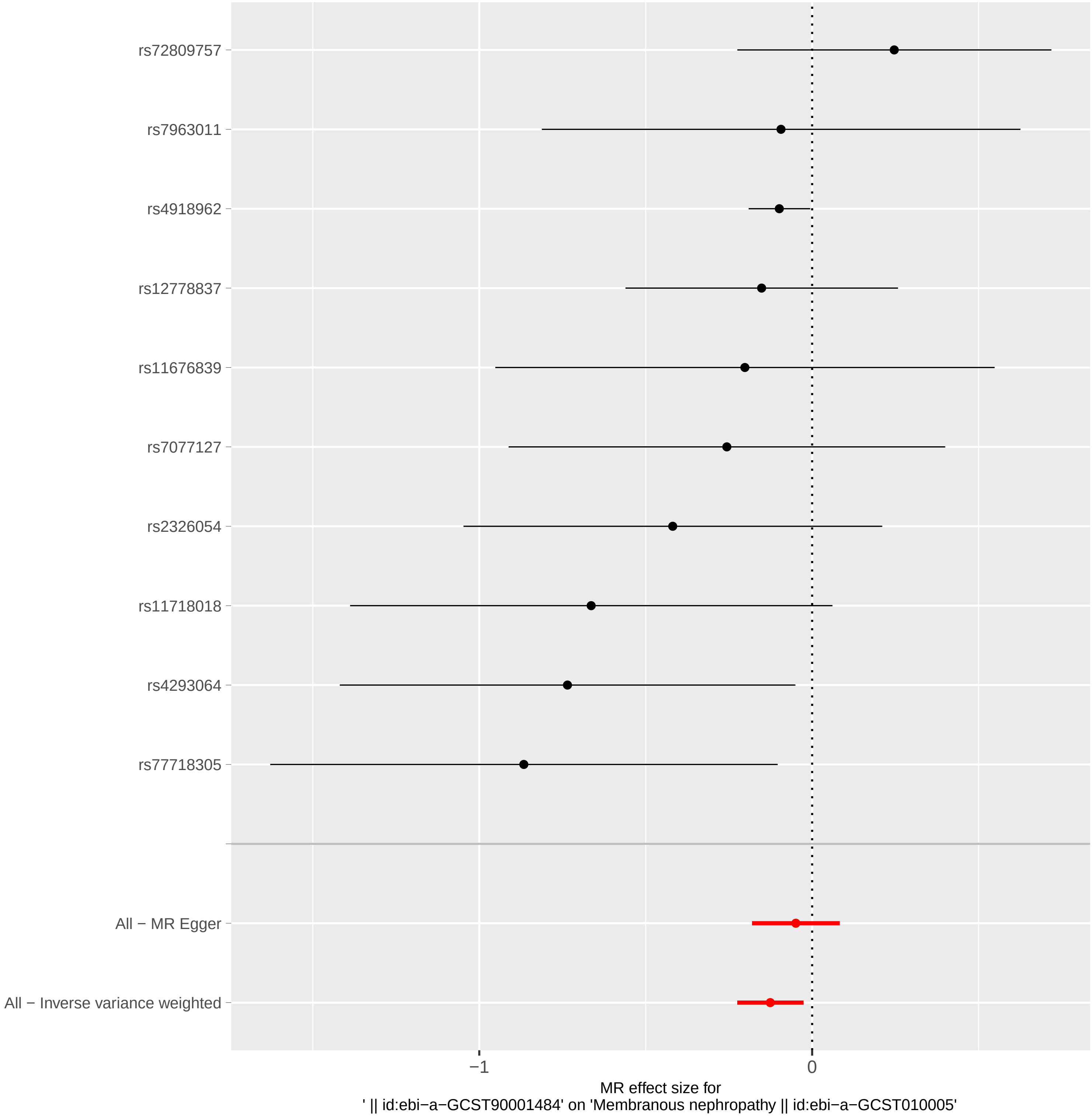

rs72809757
rs7963011
rs4918962
rs12778837
rs11676839
rs7077127
rs2326054
rs11718018
rs4293064
rs77718305
All − MR Egger
All − Inverse variance weighted
−1
0
MR effect size for
' || id:ebi−a−GCST90001484' on 'Membranous nephropathy || id:ebi−a−GCST010005'

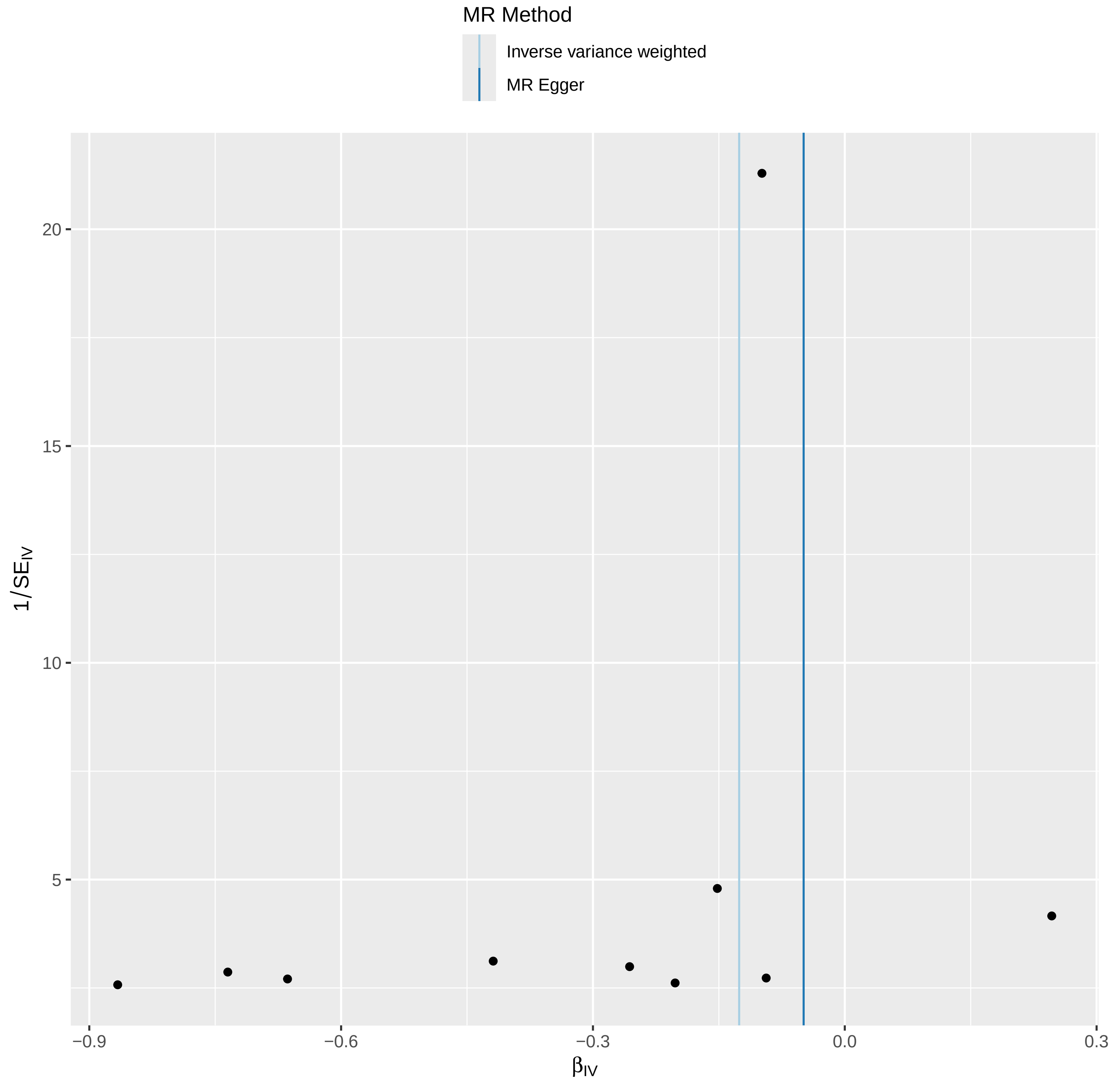
MR Method
Inverse variance weighted
MR Egger
20
15
10
5
1/SE$_{IV}$
−0.9
−0.6
−0.3
0.0
0.3
β_{IV}

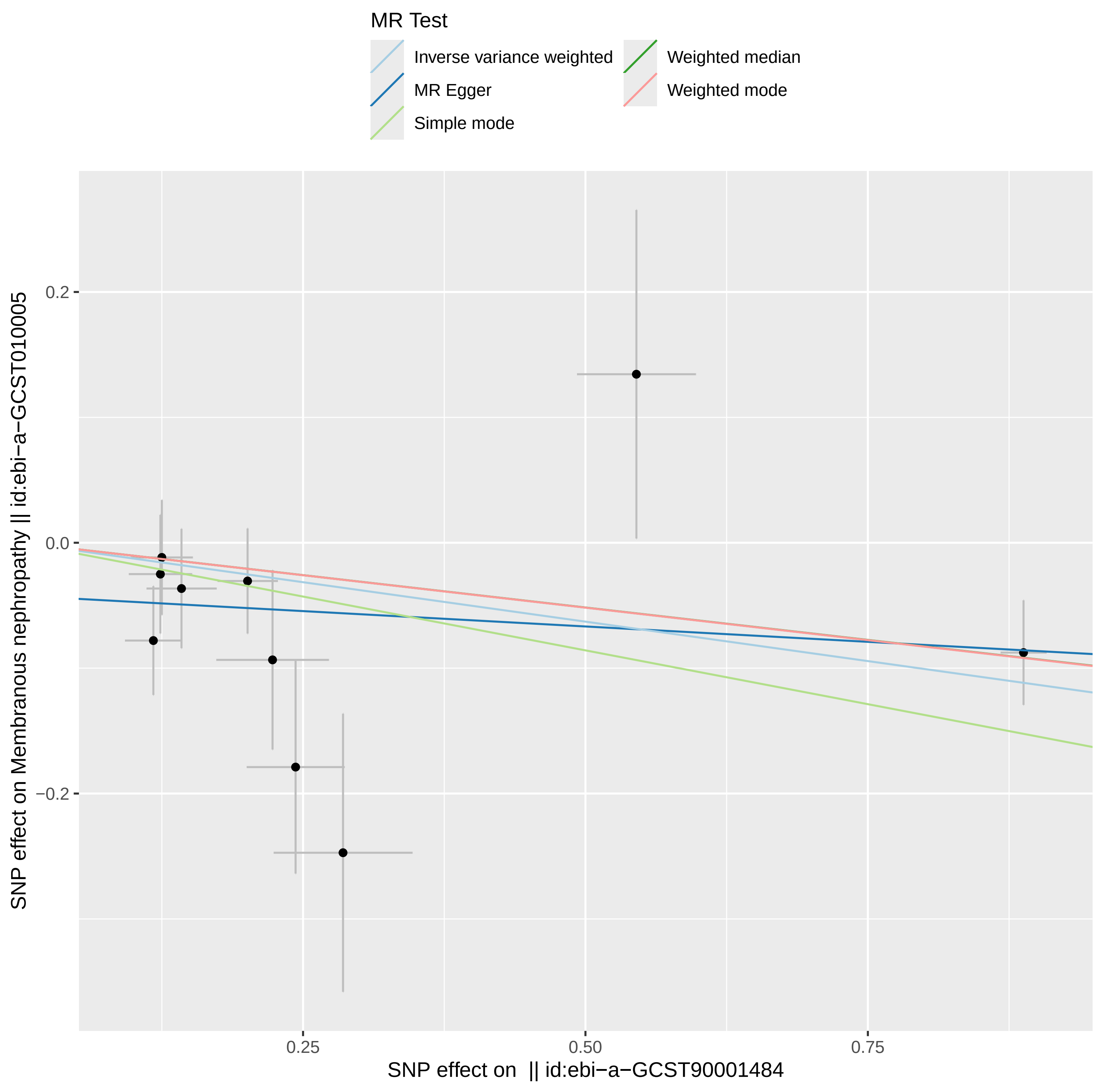
MR Test
Inverse variance weighted
MR Egger
Simple mode
Weighted median
Weighted mode
0.2
0.0
−0.2
0.25
0.50
0.75
SNP effect on Membranous nephropathy || id:ebi−a−GCST010005
SNP effect on || id:ebi−a−GCST90001484

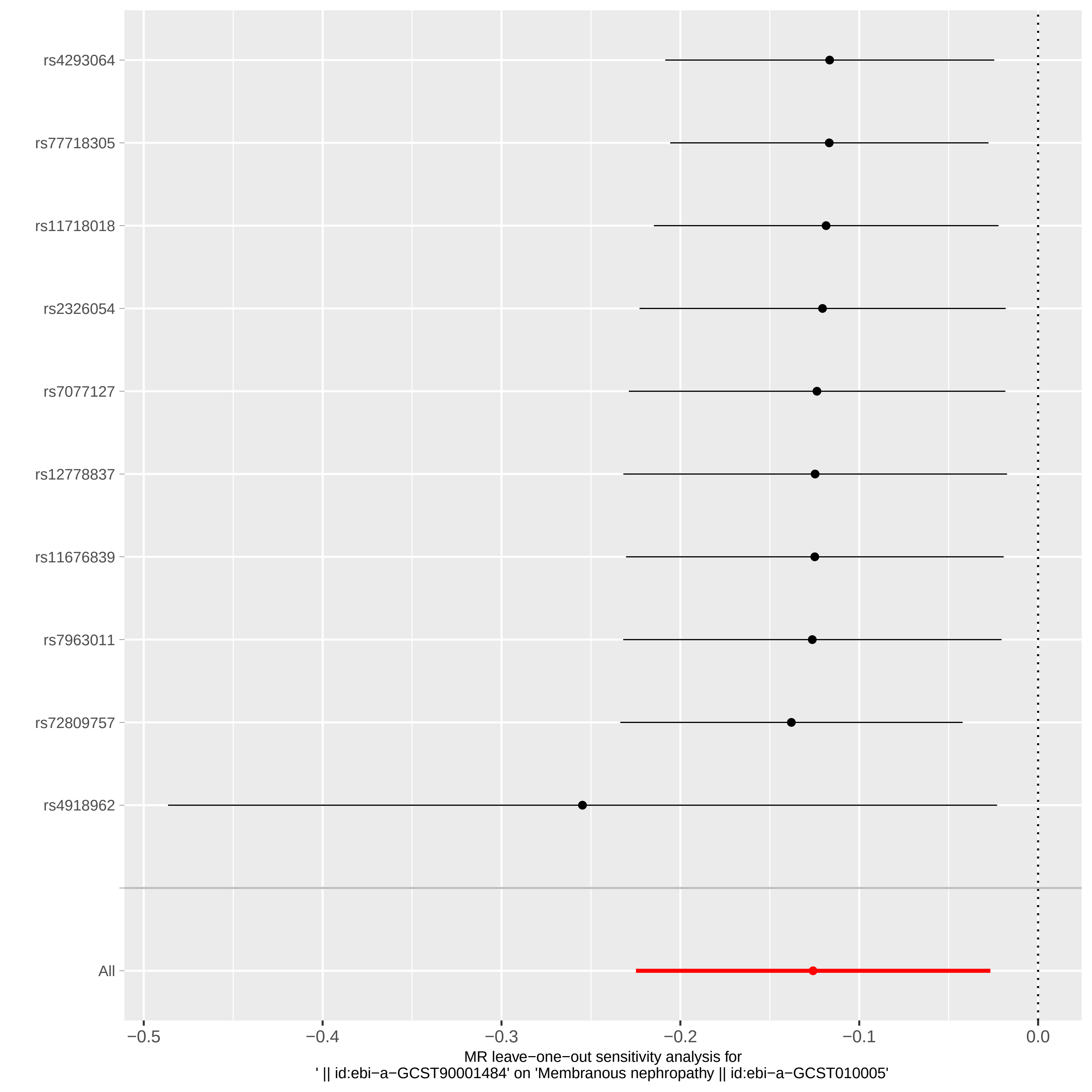

rs4293064
rs77718305
rs11718018
rs2326054
rs7077127
rs12778837
rs11676839
rs7963011
rs72809757
rs4918962
All
−0.5
−0.4
−0.3
−0.2
−0.1
0.0
MR leave−one−out sensitivity analysis for
' || id:ebi−a−GCST90001484' on 'Membranous nephropathy || id:ebi−a−GCST010005'

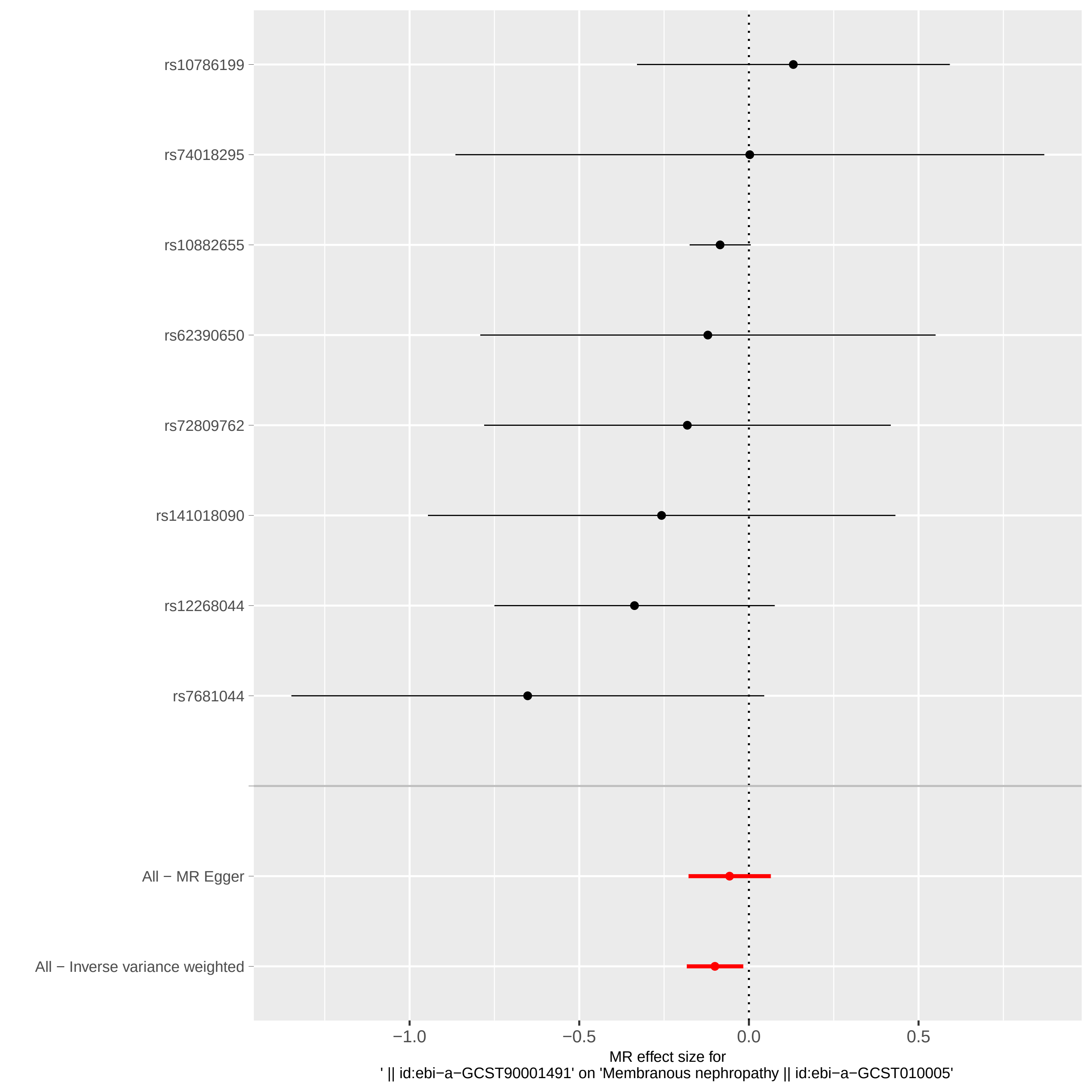

rs10786199
rs74018295
rs10882655
rs62390650
rs72809762
rs141018090
rs12268044
rs7681044
All − MR Egger
All − Inverse variance weighted
−1.0
−0.5
0.0
0.5
MR effect size for
' || id:ebi−a−GCST90001491' on 'Membranous nephropathy || id:ebi−a−GCST010005'

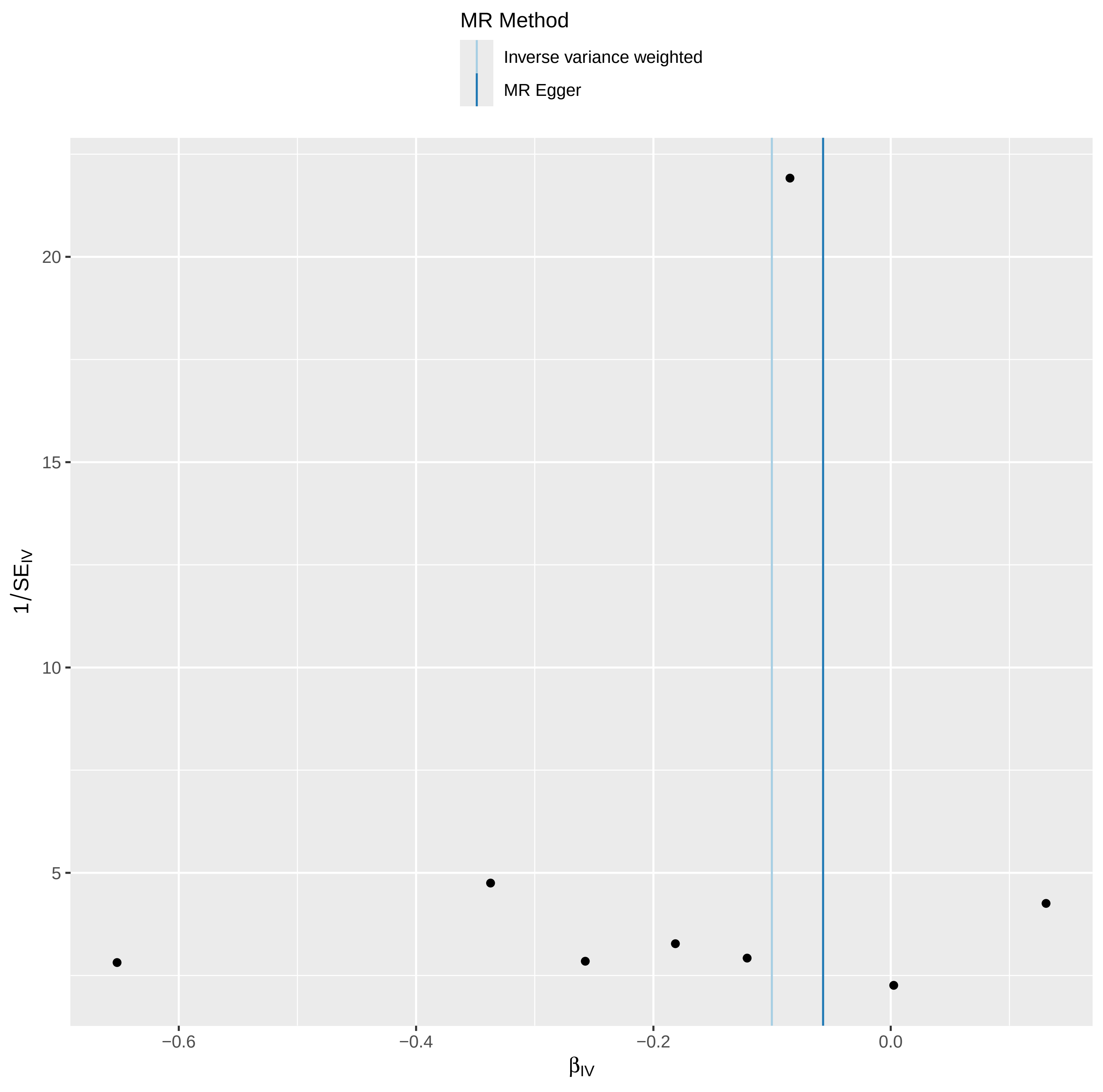
MR Method
Inverse variance weighted
MR Egger
20
15
10
5
1/SE_IV
−0.6
−0.4
−0.2
0.0
β_IV

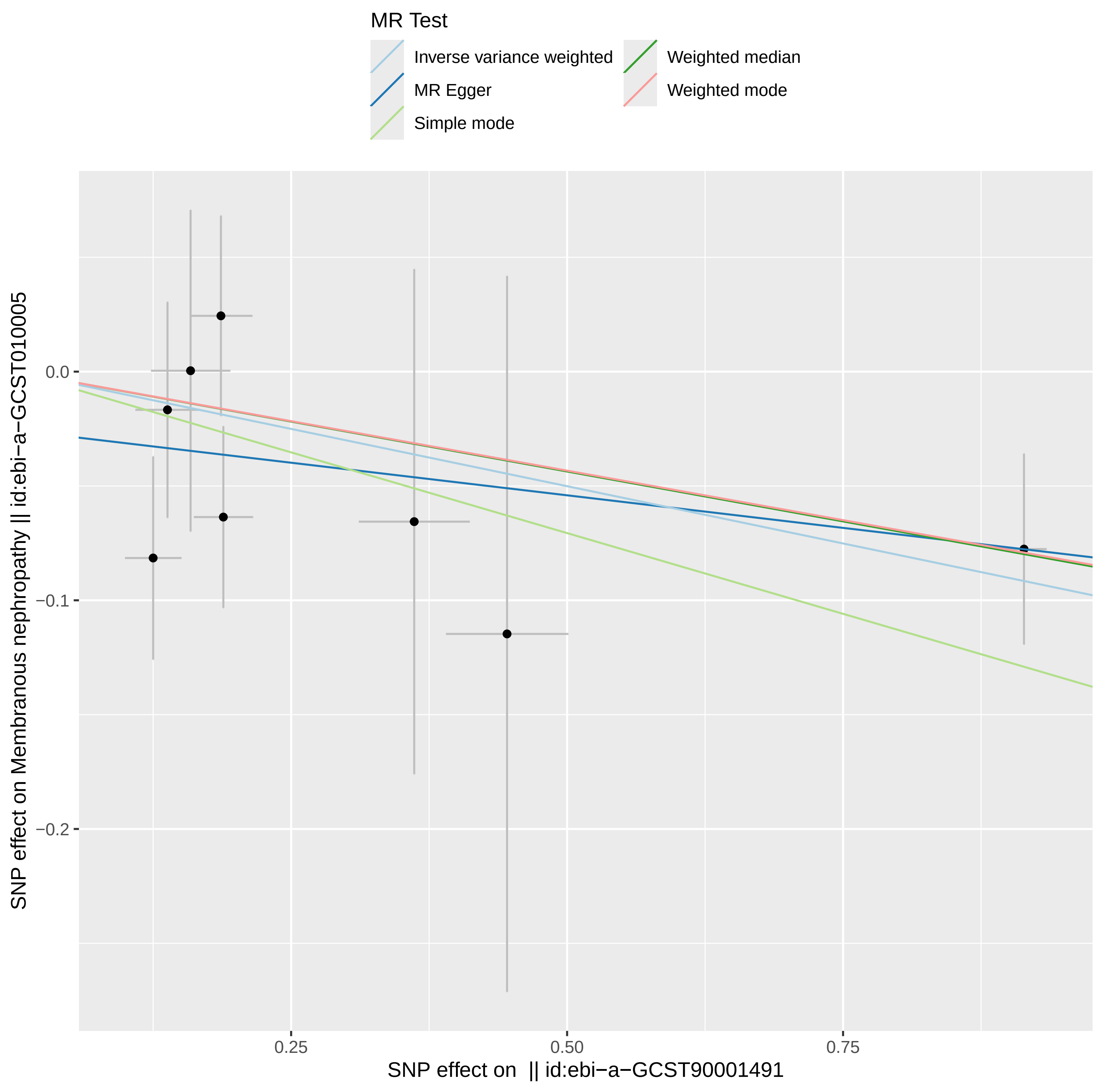

MR Test
Inverse variance weighted
MR Egger
Simple mode
Weighted median
Weighted mode
0.0
−0.1
−0.2
0.25
0.50
0.75
SNP effect on Membranous nephropathy || id:ebi−a−GCST010005
SNP effect on || id:ebi−a−GCST90001491

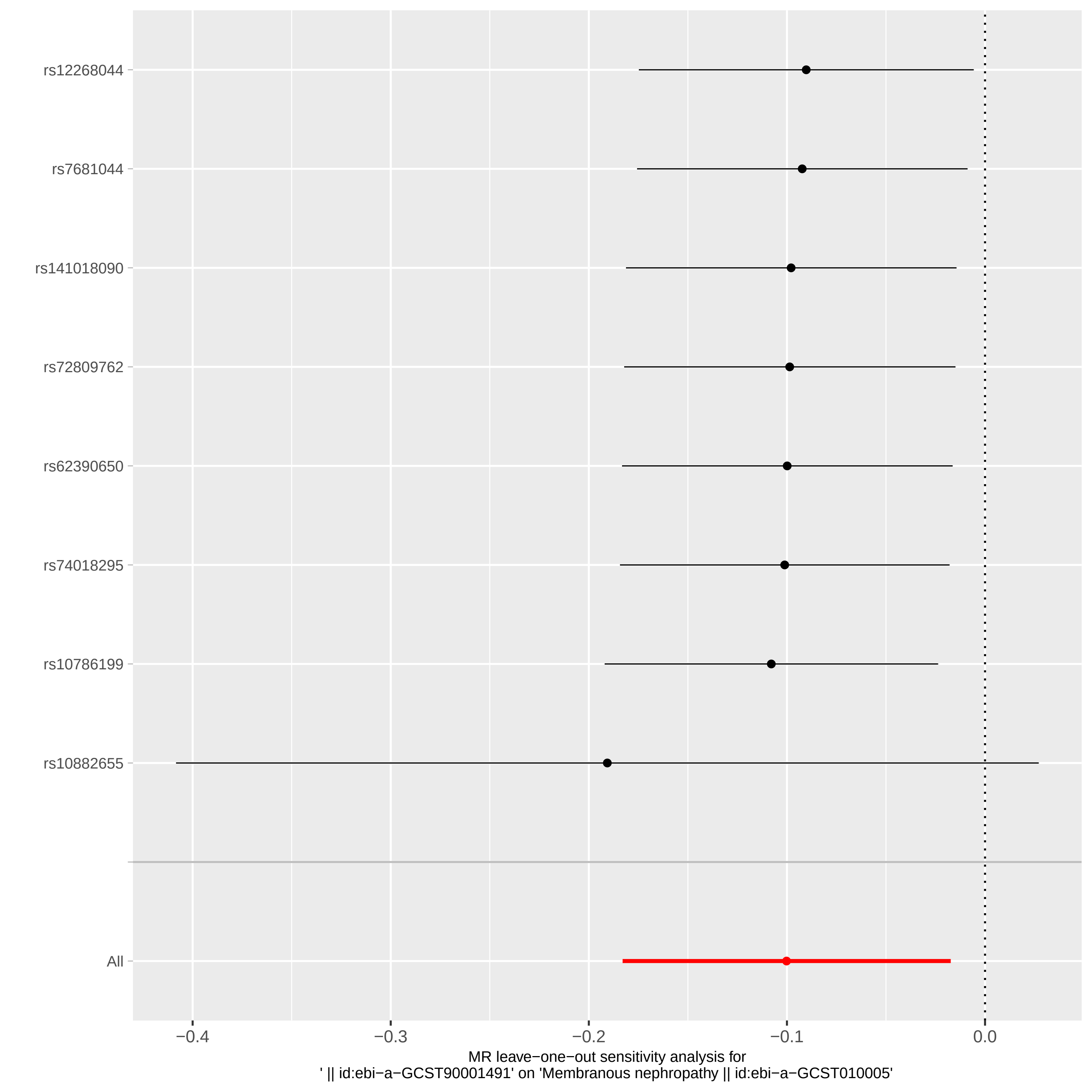

rs12268044
rs7681044
rs141018090
rs72809762
rs62390650
rs74018295
rs10786199
rs10882655
All
−0.4
−0.3
−0.2
−0.1
0.0
MR leave−one−out sensitivity analysis for
' || id:ebi−a−GCST90001491' on 'Membranous nephropathy || id:ebi−a−GCST010005'

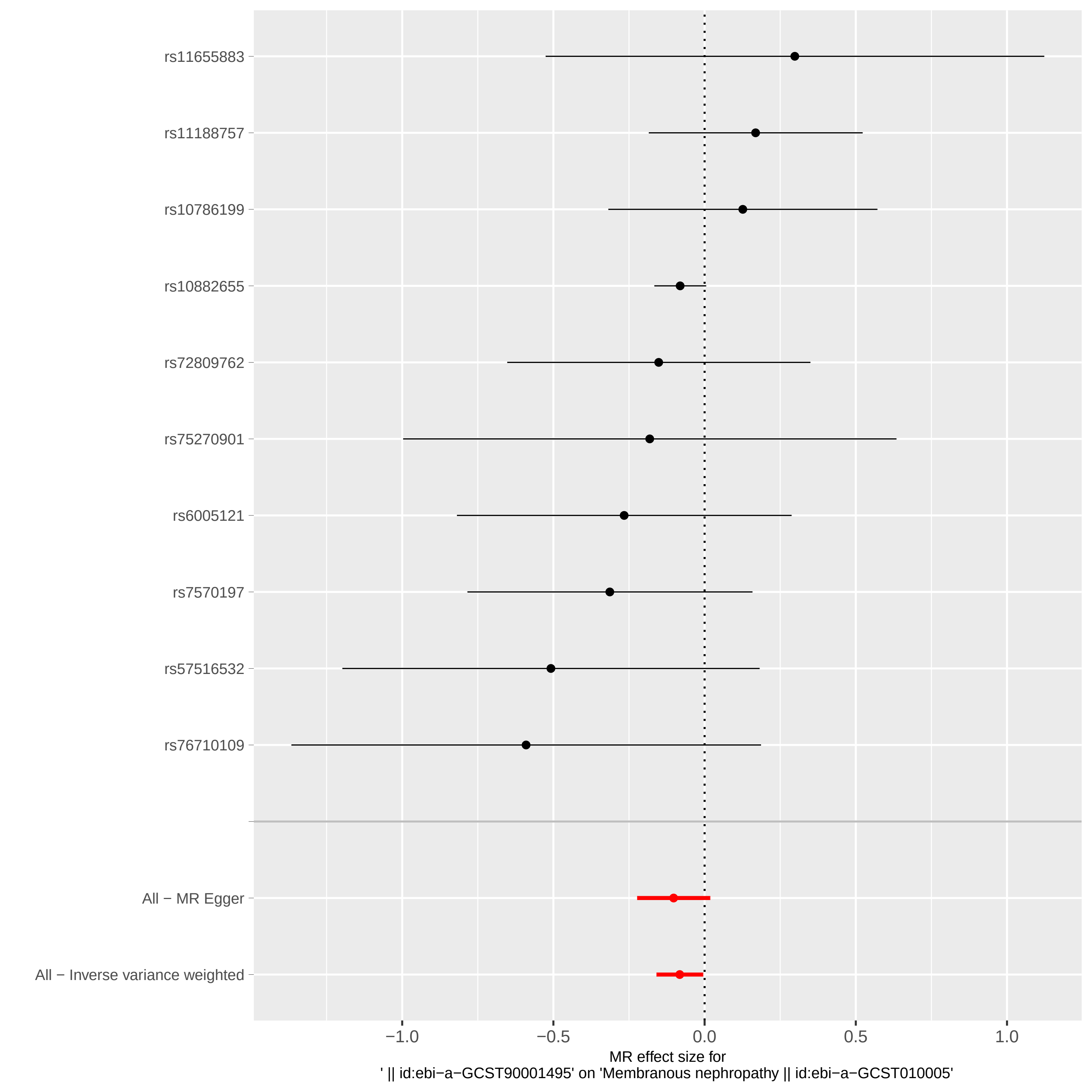
rs11655883
rs11188757
rs10786199
rs10882655
rs72809762
rs75270901
rs6005121
rs7570197
rs57516532
rs76710109
All − MR Egger
All − Inverse variance weighted
−1.0
−0.5
0.0
0.5
1.0
MR effect size for
' || id:ebi−a−GCST90001495' on 'Membranous nephropathy || id:ebi−a−GCST010005'

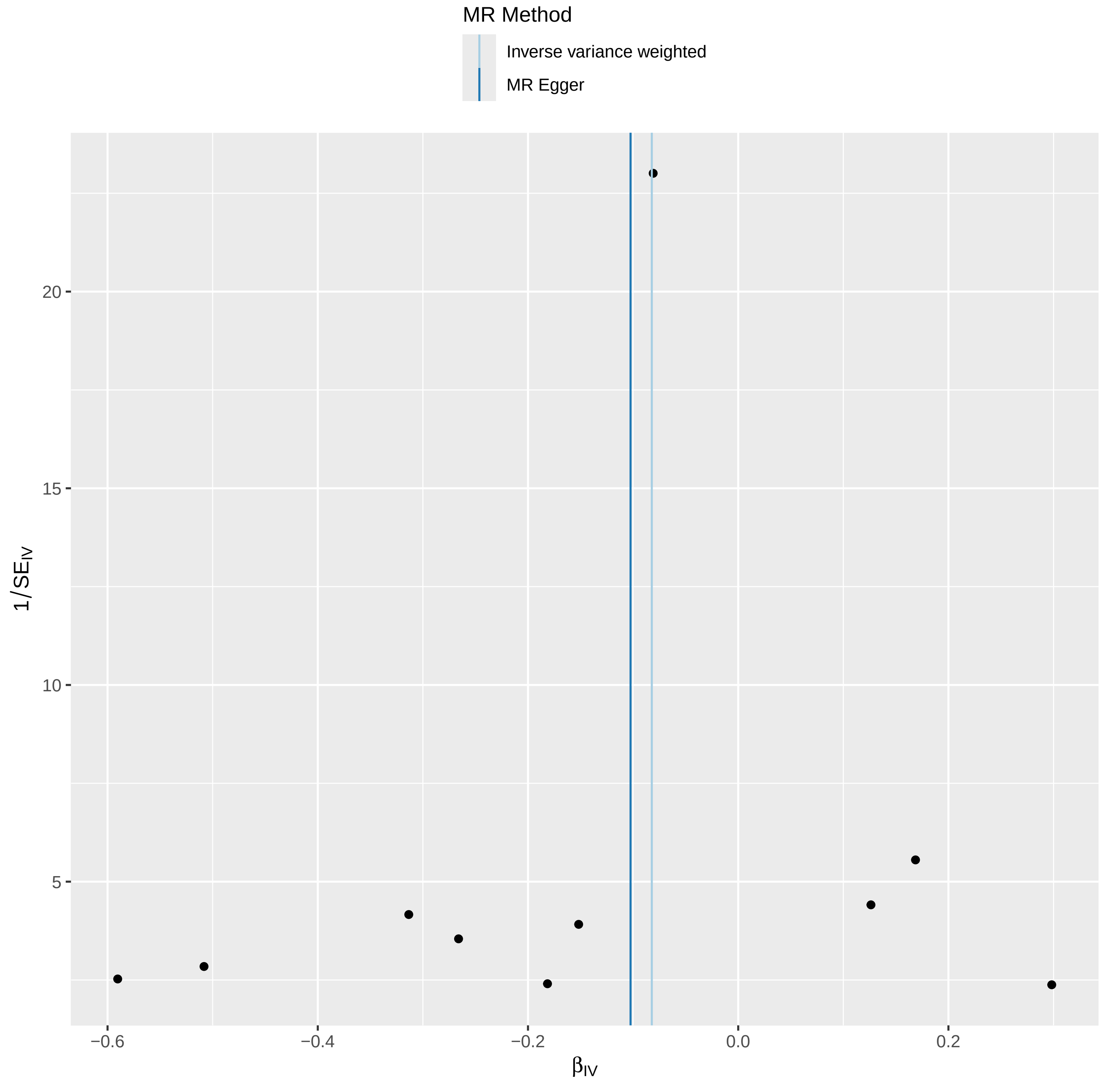

MR Method
Inverse variance weighted
MR Egger
20
15
10
5
1/SE$_{IV}$
−0.6
−0.4
−0.2
0.0
0.2
β$_{IV}$

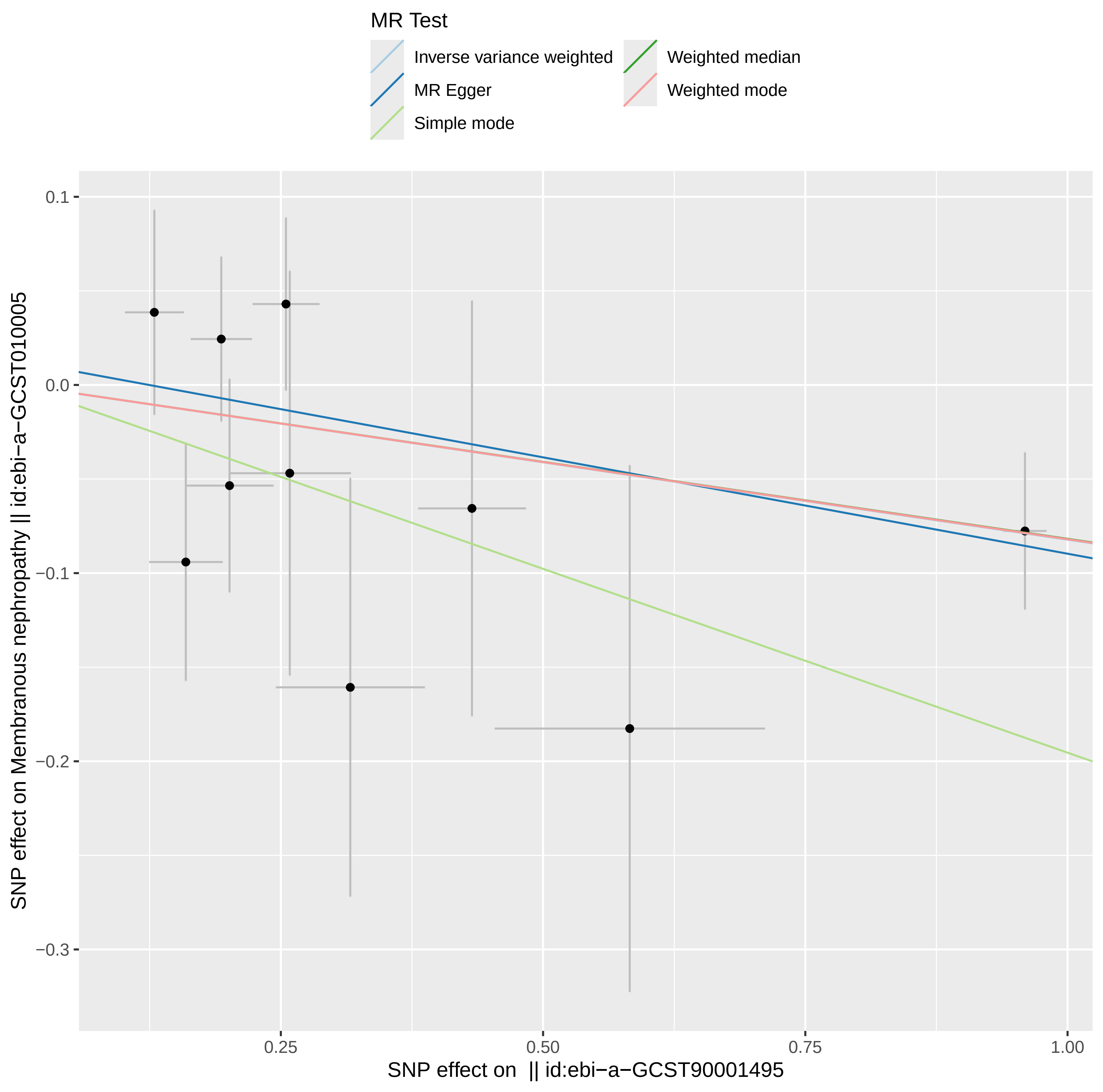

MR Test
Inverse variance weighted
MR Egger
Simple mode
Weighted median
Weighted mode
0.1
0.0
−0.1
−0.2
−0.3
0.25
0.50
0.75
1.00
SNP effect on Membranous nephropathy || id:ebi−a−GCST010005
SNP effect on || id:ebi−a−GCST90001495

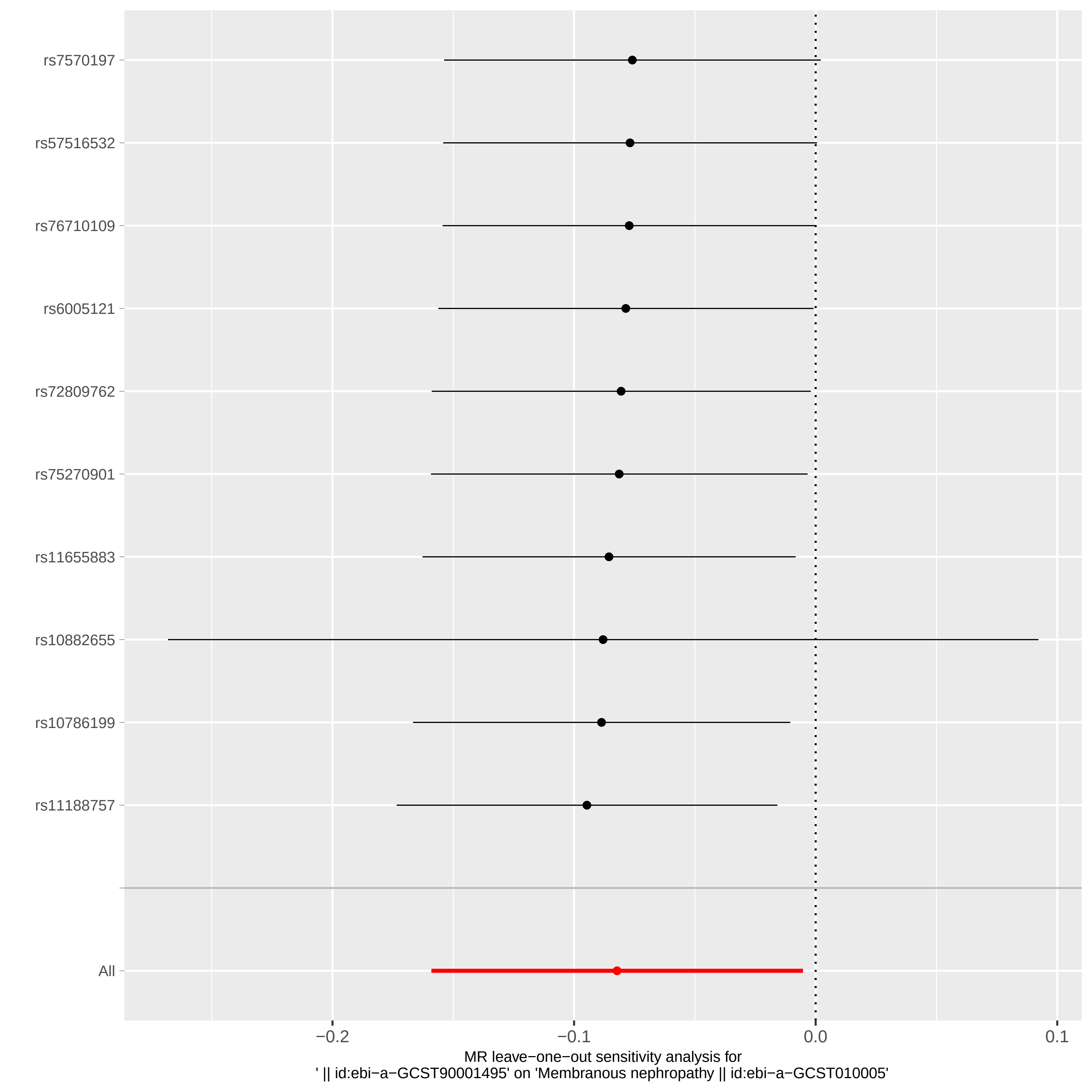
rs7570197
rs57516532
rs76710109
rs6005121
rs72809762
rs75270901
rs11655883
rs10882655
rs10786199
rs11188757
All
−0.2
−0.1
0.0
0.1
MR leave−one−out sensitivity analysis for
' || id:ebi−a−GCST90001495' on 'Membranous nephropathy || id:ebi−a−GCST010005'

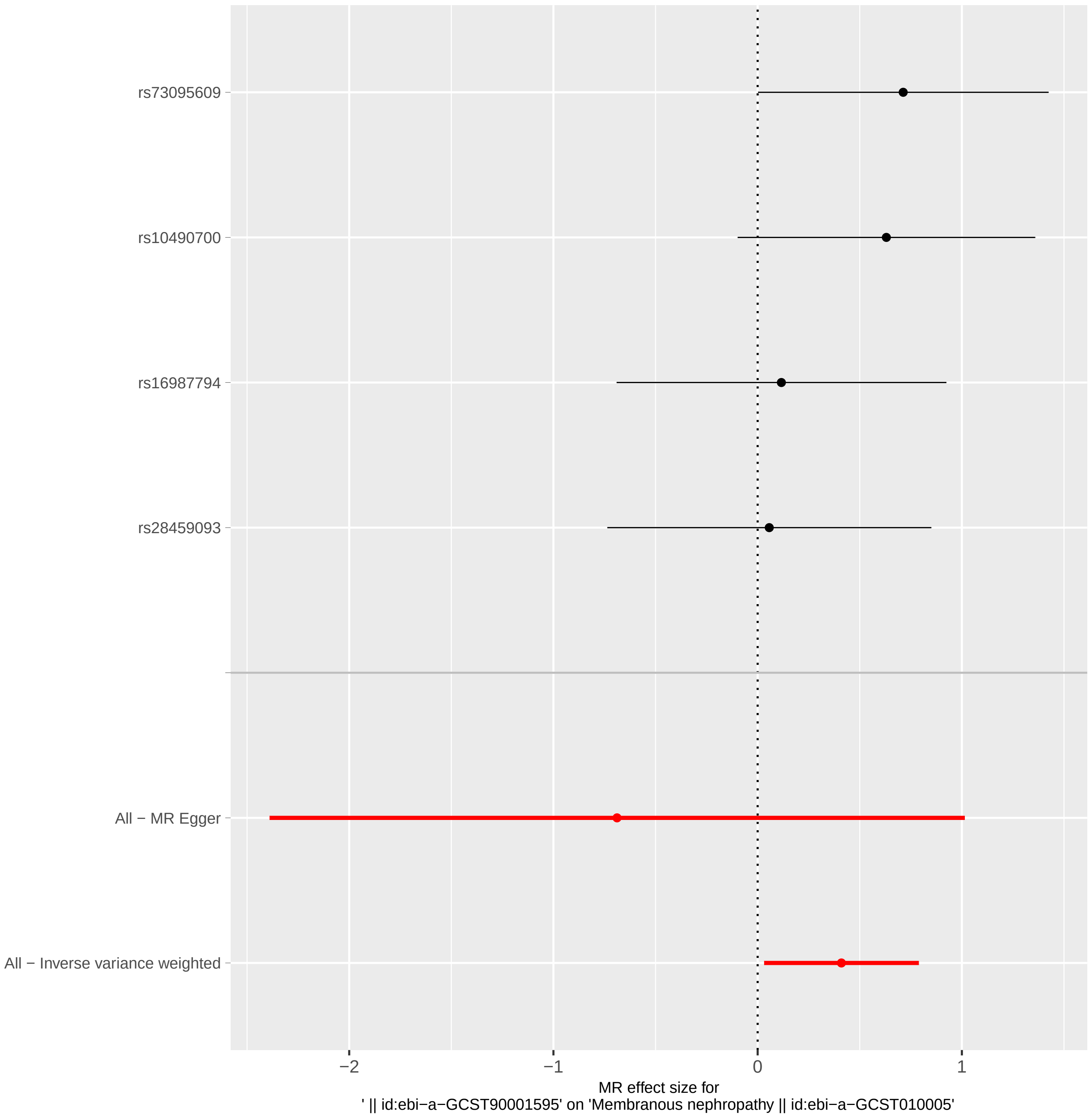

rs73095609
rs10490700
rs16987794
rs28459093
All − MR Egger
All − Inverse variance weighted
−2
−1
0
1
MR effect size for
' || id:ebi−a−GCST90001595' on 'Membranous nephropathy || id:ebi−a−GCST010005'

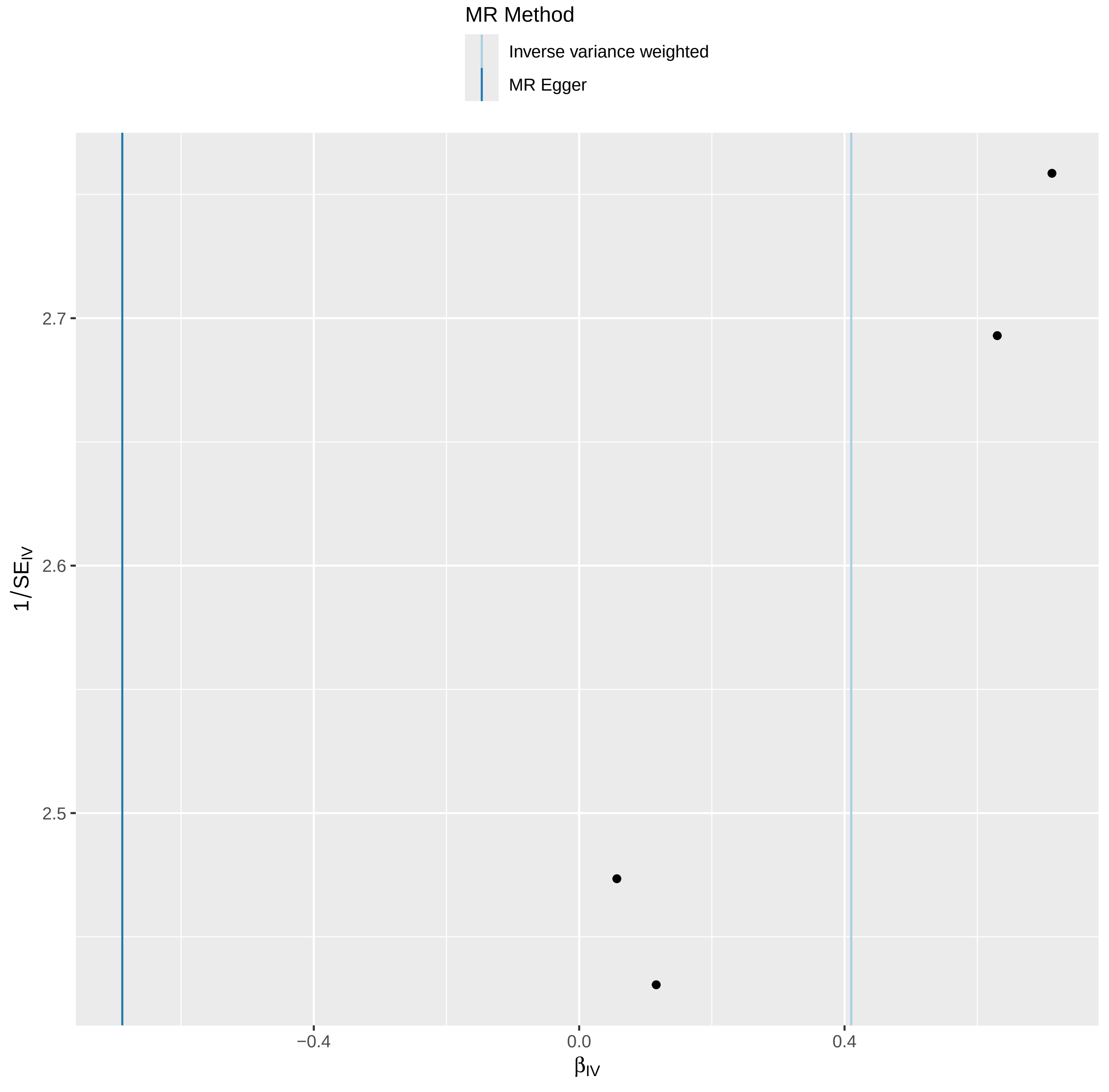
MR Method
Inverse variance weighted
MR Egger
2.7
2.6
2.5
1/SE_IV
−0.4
0.0
0.4
β_IV

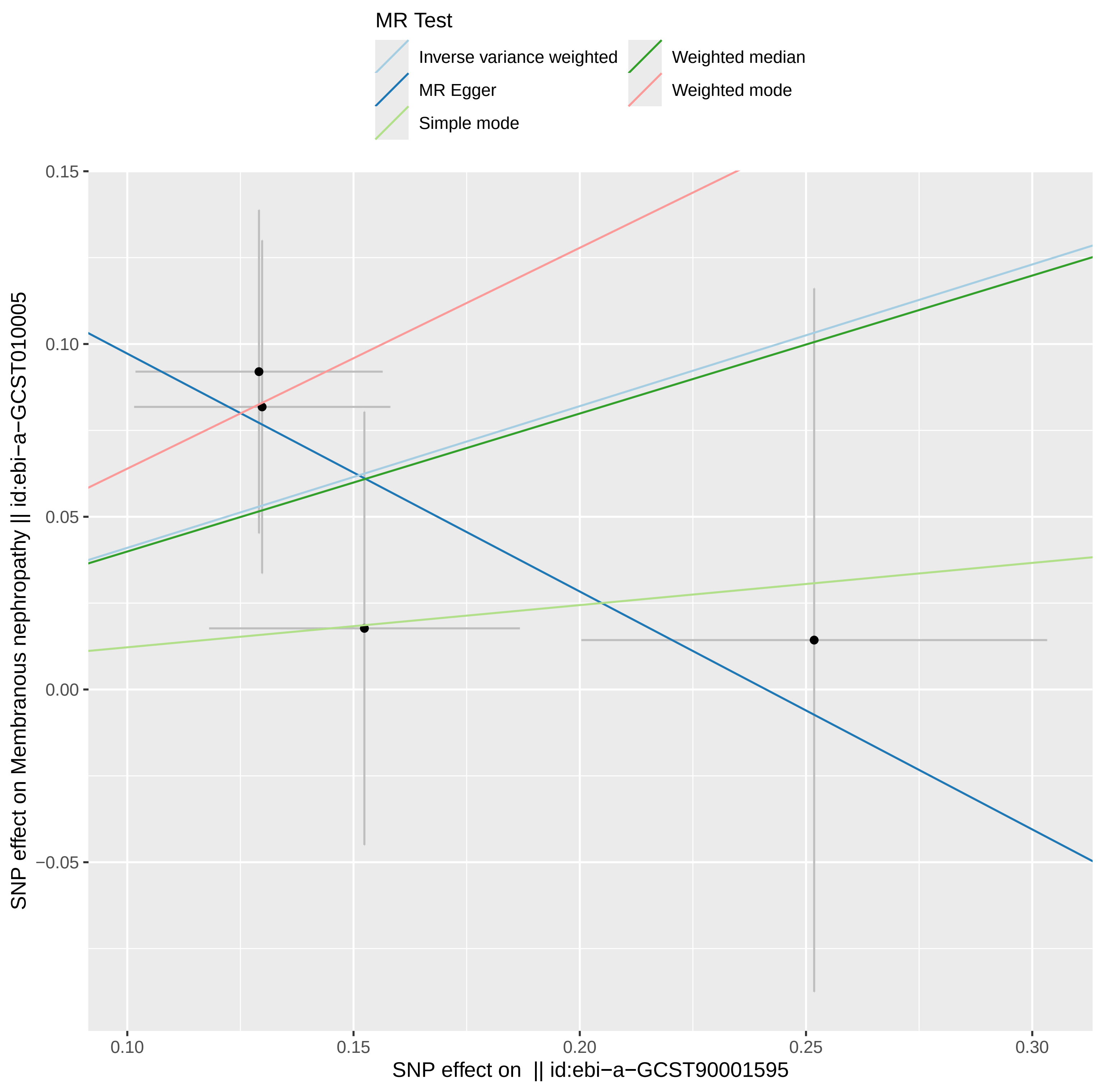
MR Test
Inverse variance weighted
MR Egger
Simple mode
Weighted median
Weighted mode
SNP effect on Membranous nephropathy || id:ebi−a−GCST010005
0.15
0.10
0.05
0.00
−0.05
0.10
0.15
0.20
0.25
0.30
SNP effect on || id:ebi−a−GCST90001595

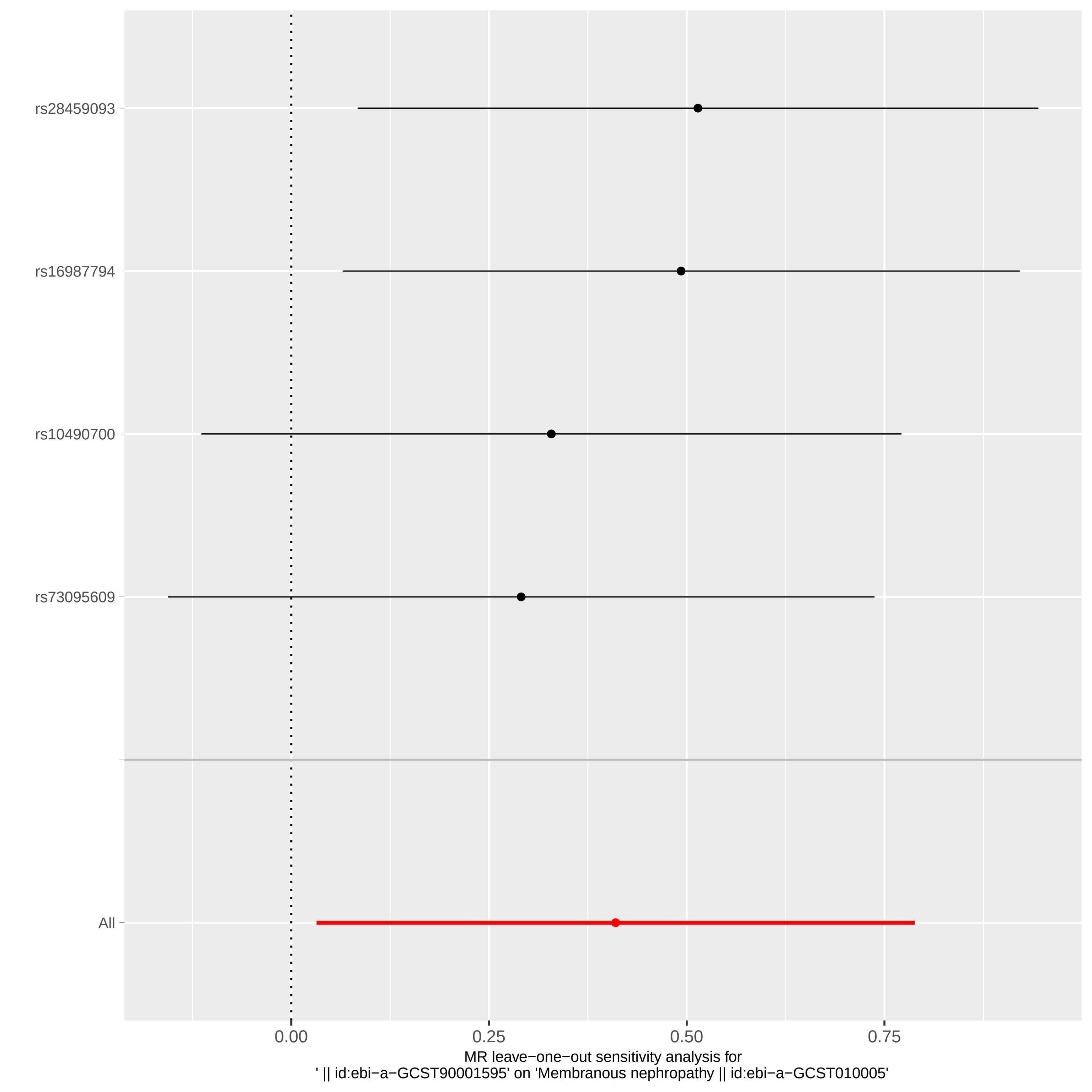

rs28459093
rs16987794
rs10490700
rs73095609
All
0.00
0.25
0.50
0.75
MR leave−one−out sensitivity analysis for
' || id:ebi−a−GCST90001595' on 'Membranous nephropathy || id:ebi−a−GCST010005'

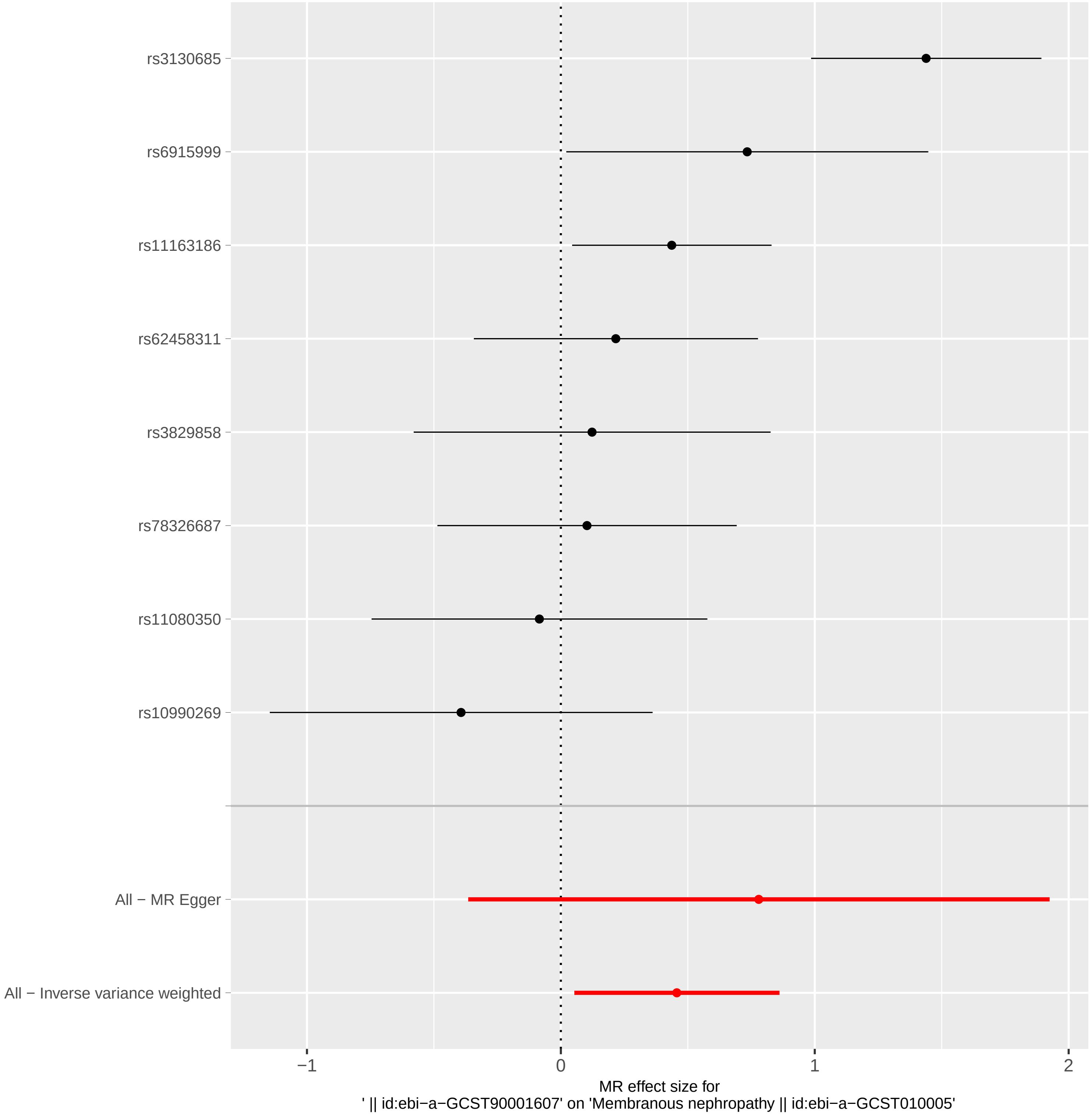
rs3130685
rs6915999
rs11163186
rs62458311
rs3829858
rs78326687
rs11080350
rs10990269
All − MR Egger
All − Inverse variance weighted
−1
0
1
2
MR effect size for
' || id:ebi−a−GCST90001607' on 'Membranous nephropathy || id:ebi−a−GCST010005'

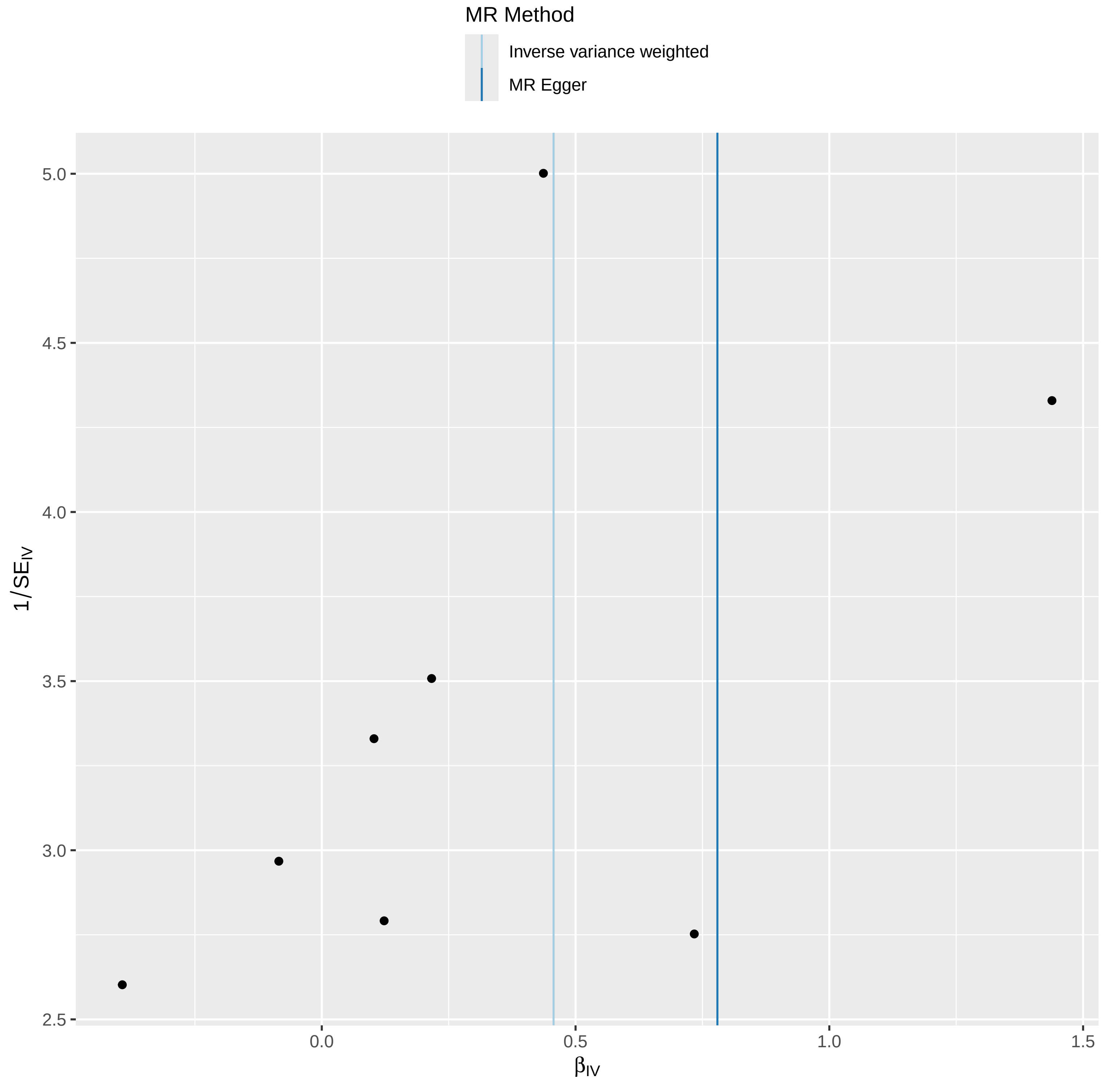
MR Method
Inverse variance weighted
MR Egger
5.0
4.5
4.0
3.5
3.0
2.5
1/SE_IV
0.0
0.5
1.0
1.5
β_IV

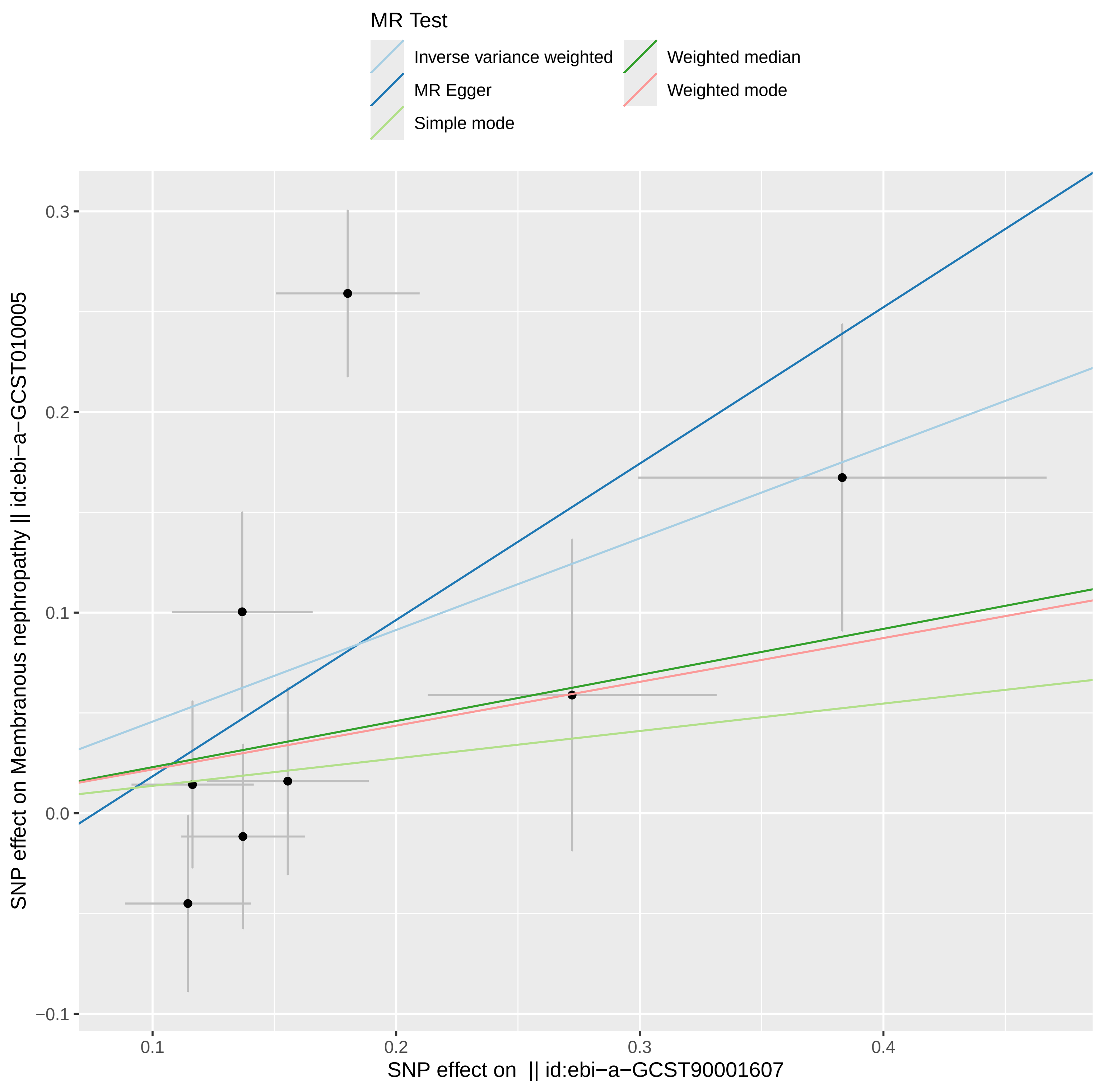

MR Test
Inverse variance weighted
MR Egger
Simple mode
Weighted median
Weighted mode
0.3
0.2
0.1
0.0
−0.1
0.1
0.2
0.3
0.4
SNP effect on Membranous nephropathy || id:ebi−a−GCST010005
SNP effect on || id:ebi−a−GCST90001607

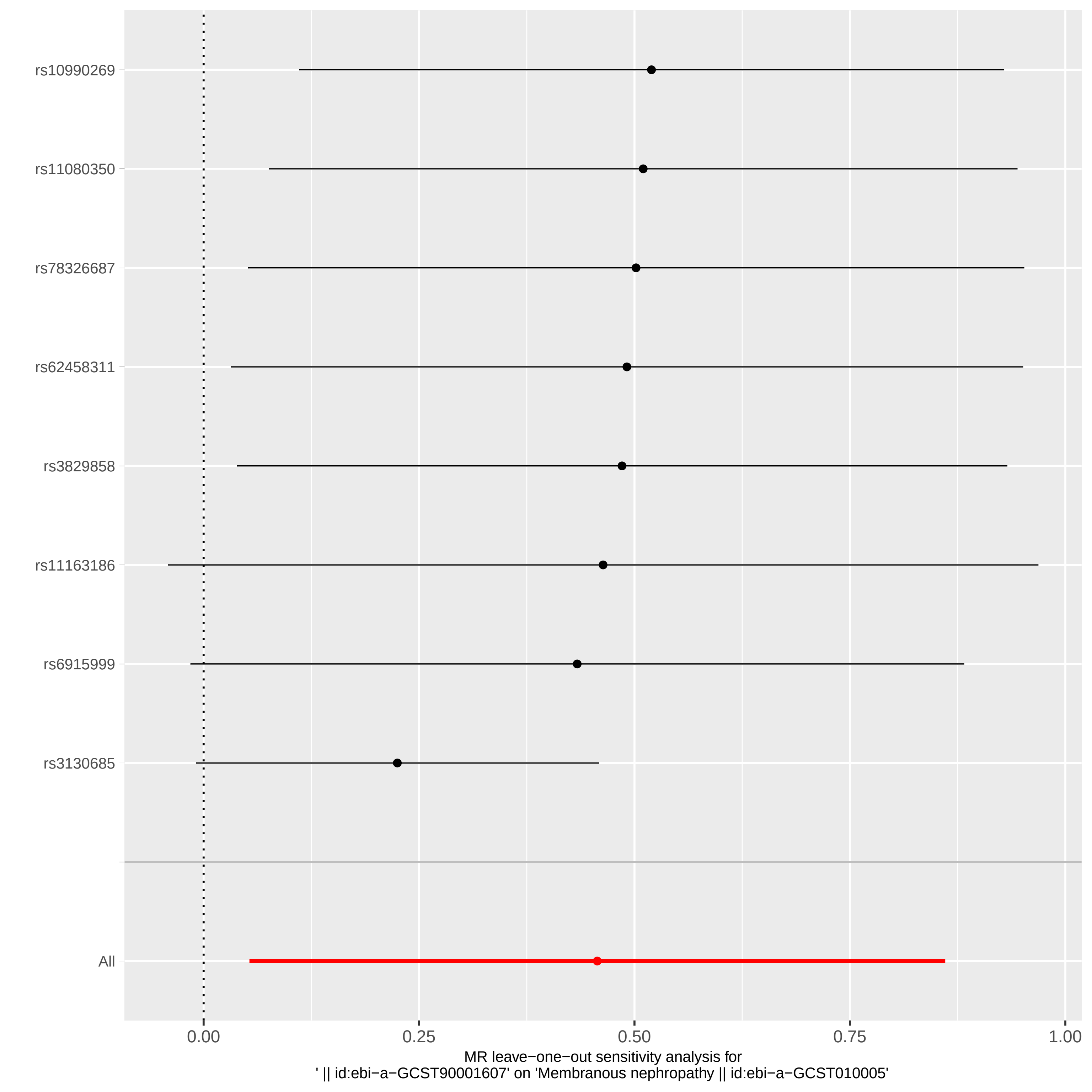
rs10990269
rs11080350
rs78326687
rs62458311
rs3829858
rs11163186
rs6915999
rs3130685
All
0.00
0.25
0.50
0.75
1.00
MR leave−one−out sensitivity analysis for
' || id:ebi−a−GCST90001607' on 'Membranous nephropathy || id:ebi−a−GCST010005'

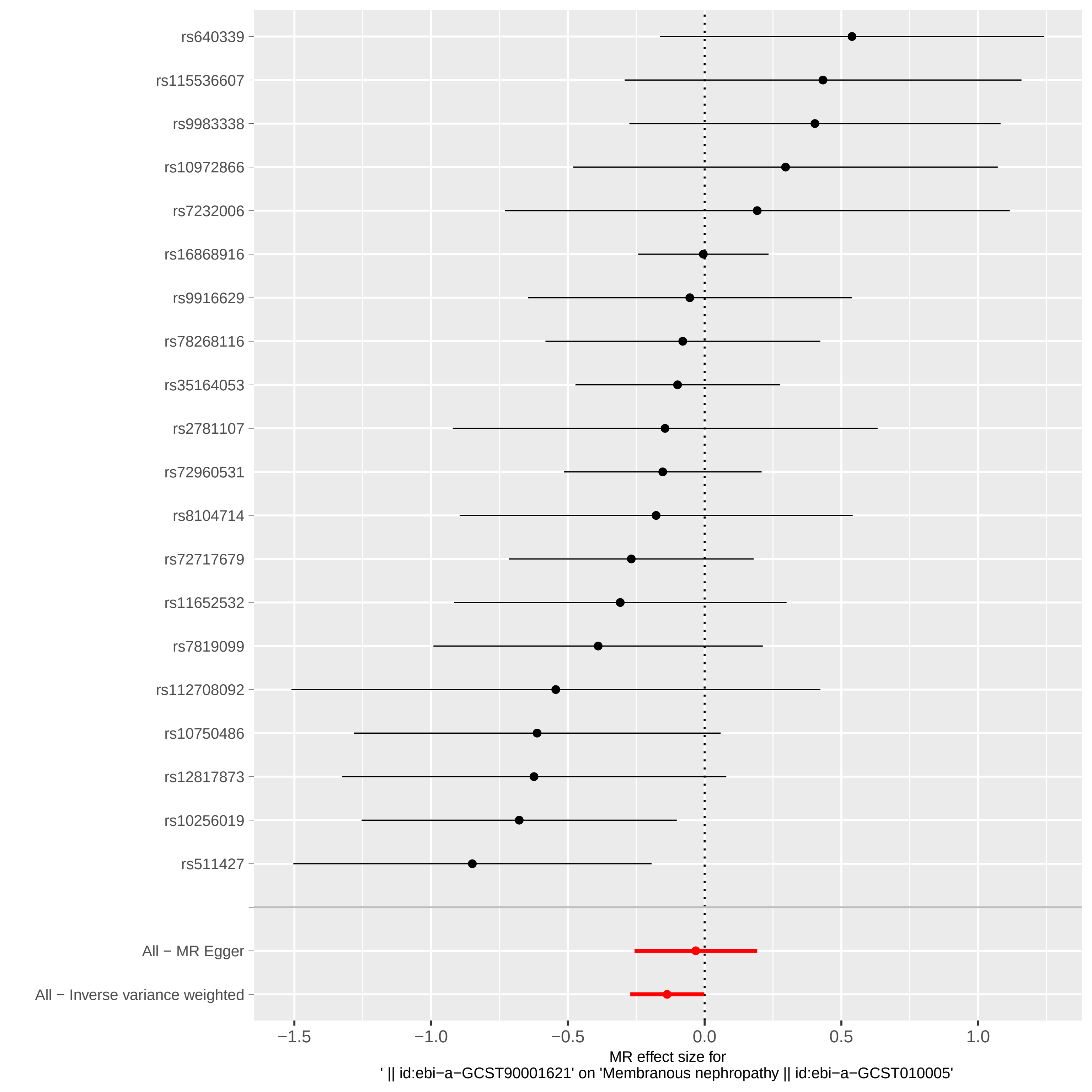
rs640339
rs115536607
rs9983338
rs10972866
rs7232006
rs16868916
rs9916629
rs78268116
rs35164053
rs2781107
rs72960531
rs8104714
rs72717679
rs11652532
rs7819099
rs112708092
rs10750486
rs12817873
rs10256019
rs511427
All − MR Egger
All − Inverse variance weighted
−1.5
−1.0
−0.5
0.0
0.5
1.0
MR effect size for
' || id:ebi−a−GCST90001621' on 'Membranous nephropathy || id:ebi−a−GCST010005'

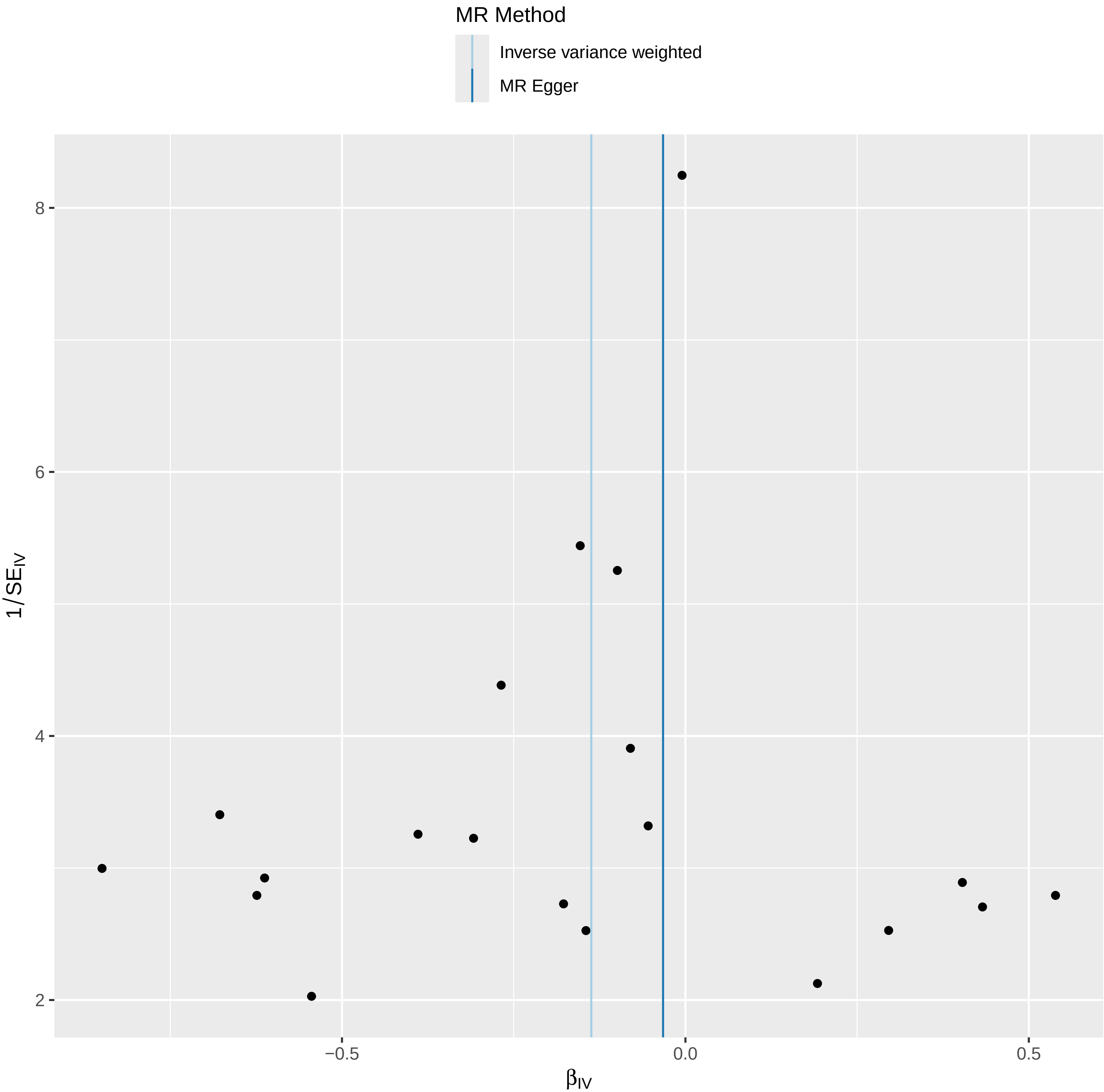

MR Method
Inverse variance weighted
MR Egger
8
6
4
2
$1/SE_{IV}$
−0.5
0.0
0.5
β_{IV}

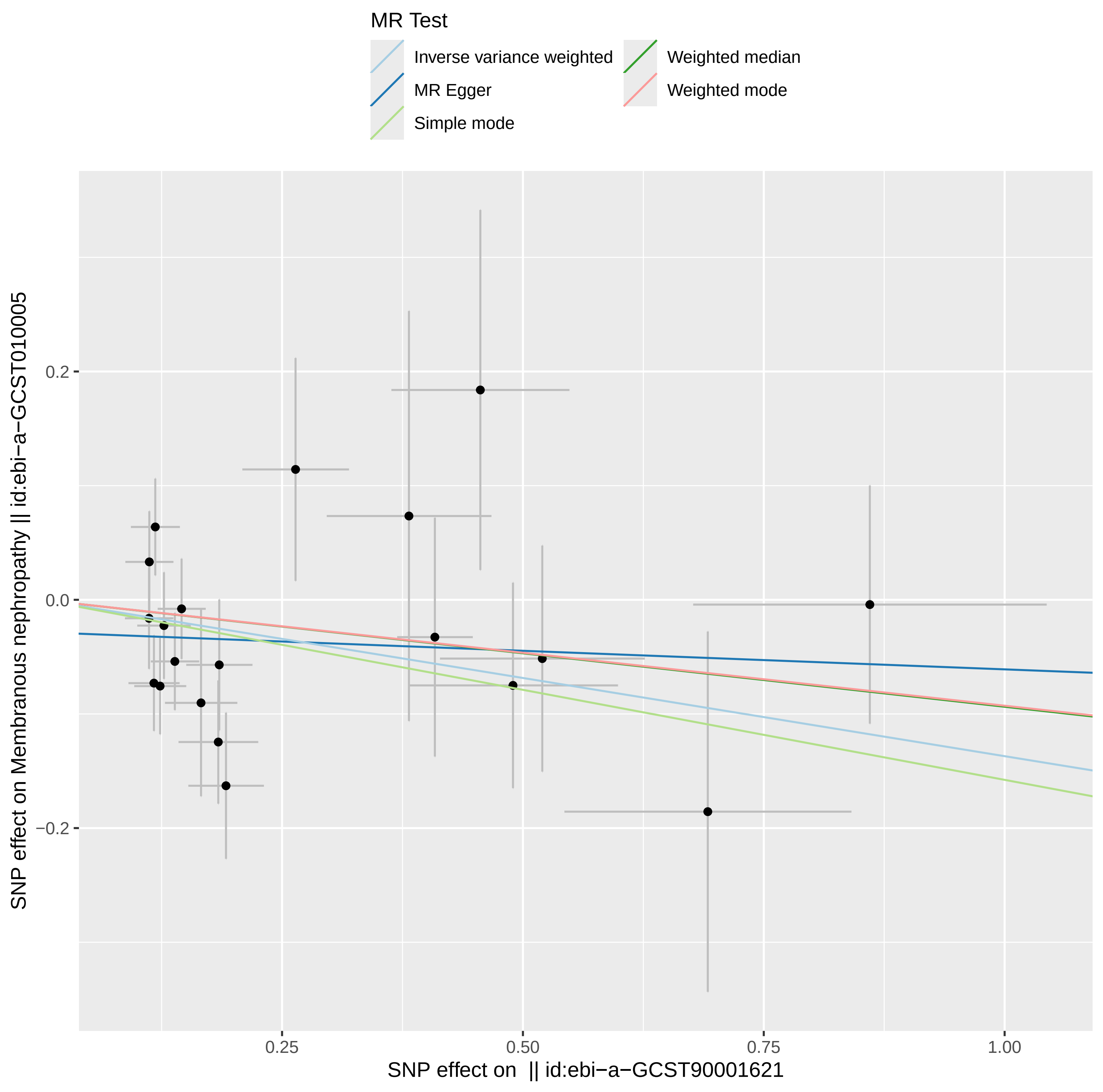

MR Test
Inverse variance weighted
MR Egger
Simple mode
Weighted median
Weighted mode
0.2
0.0
−0.2
0.25
0.50
0.75
1.00
SNP effect on Membranous nephropathy || id:ebi−a−GCST010005
SNP effect on || id:ebi−a−GCST90001621

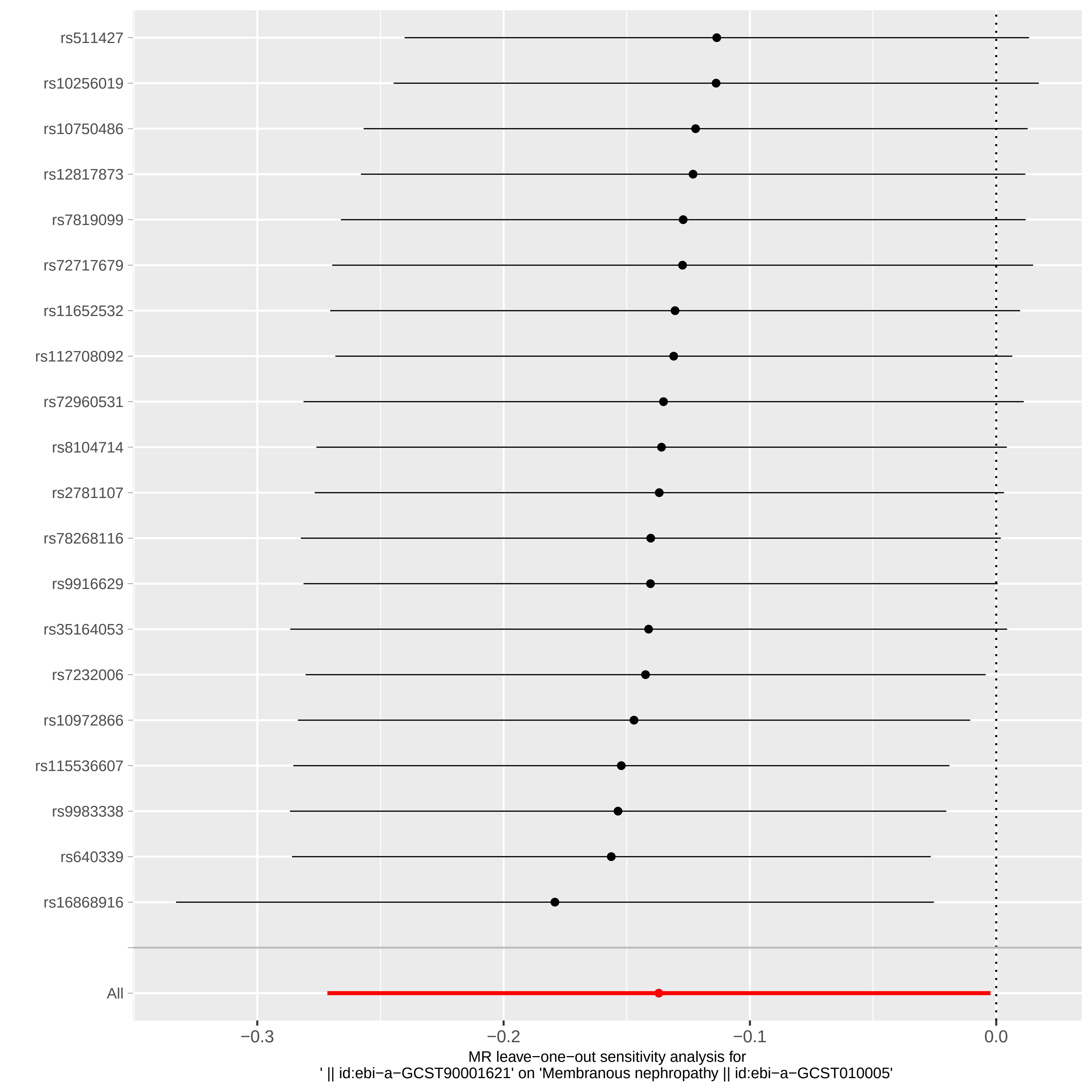
rs511427
rs10256019
rs10750486
rs12817873
rs7819099
rs72717679
rs11652532
rs112708092
rs72960531
rs8104714
rs2781107
rs78268116
rs9916629
rs35164053
rs7232006
rs10972866
rs115536607
rs9983338
rs640339
rs16868916
All
−0.3
−0.2
−0.1
0.0
MR leave−one−out sensitivity analysis for
' || id:ebi−a−GCST90001621' on 'Membranous nephropathy || id:ebi−a−GCST010005'

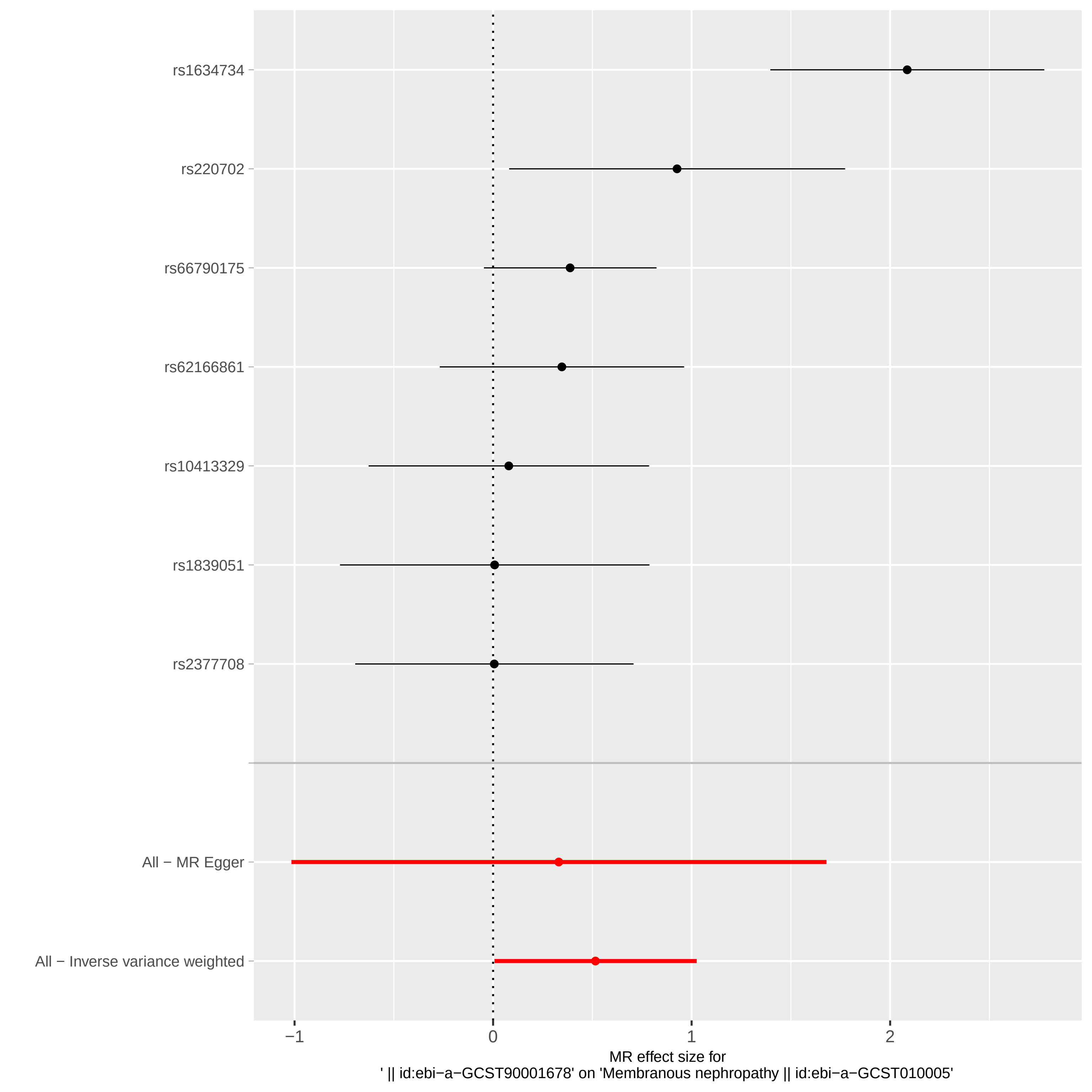
rs1634734
rs220702
rs66790175
rs62166861
rs10413329
rs1839051
rs2377708
All − MR Egger
All − Inverse variance weighted
−1
0
1
2
MR effect size for
' || id:ebi−a−GCST90001678' on 'Membranous nephropathy || id:ebi−a−GCST010005'

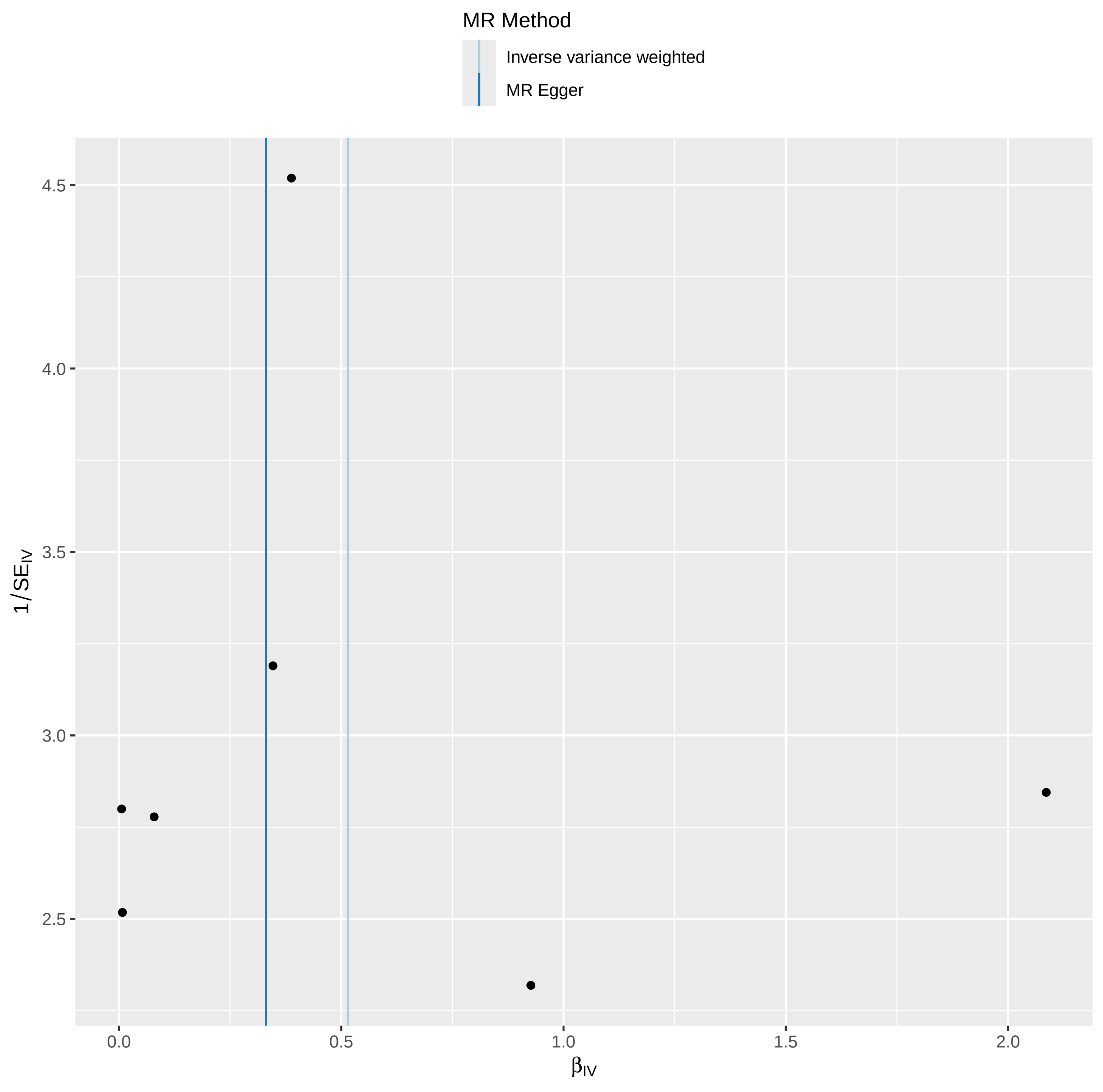

MR Method
Inverse variance weighted
MR Egger
4.5
4.0
3.5
3.0
2.5
1/SE_IV
0.0
0.5
1.0
1.5
2.0
β_IV

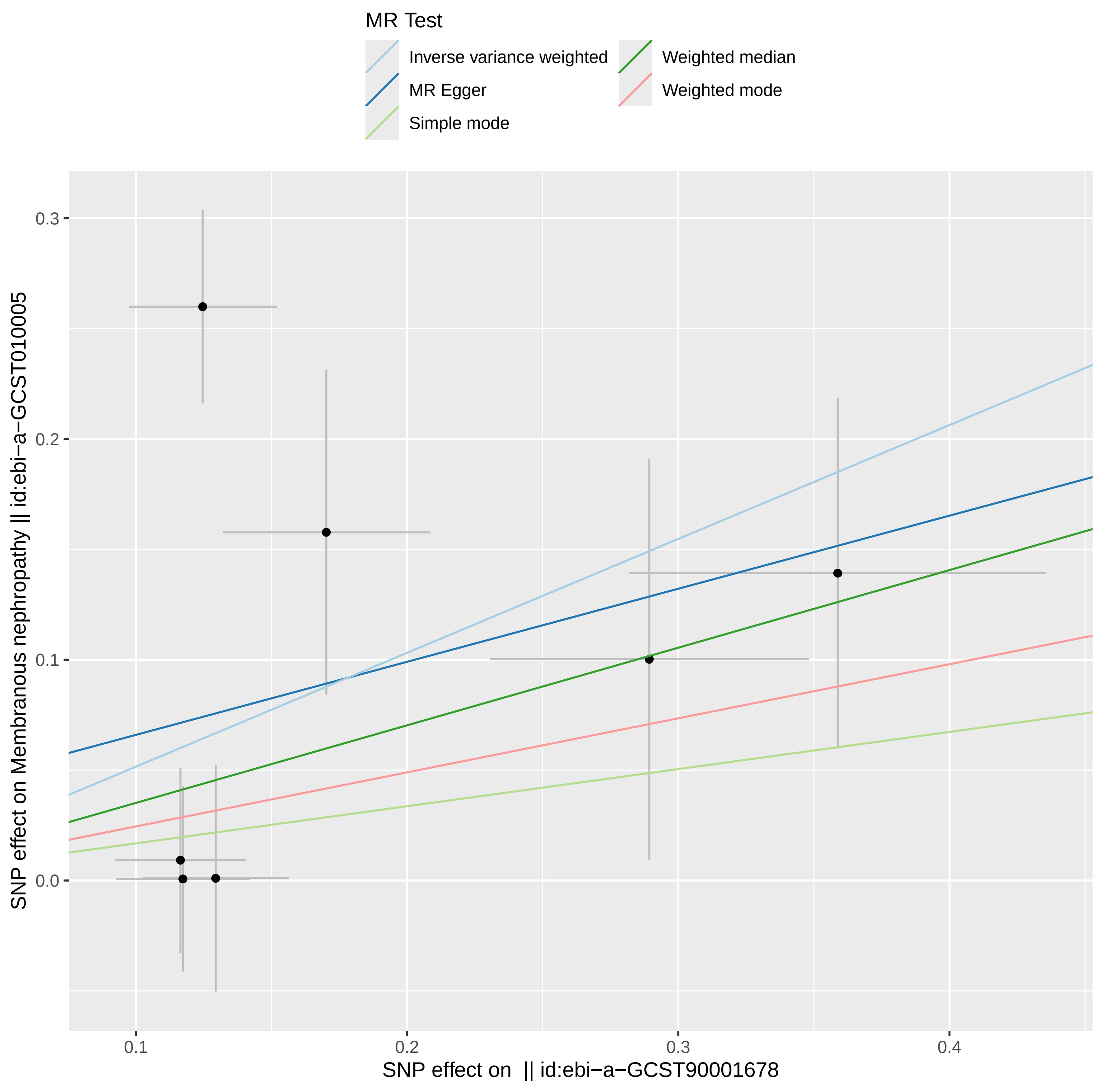

MR Test
Inverse variance weighted
MR Egger
Simple mode
Weighted median
Weighted mode
0.3
0.2
0.1
0.0
0.1
0.2
0.3
0.4
SNP effect on Membranous nephropathy || id:ebi−a−GCST010005
SNP effect on || id:ebi−a−GCST90001678

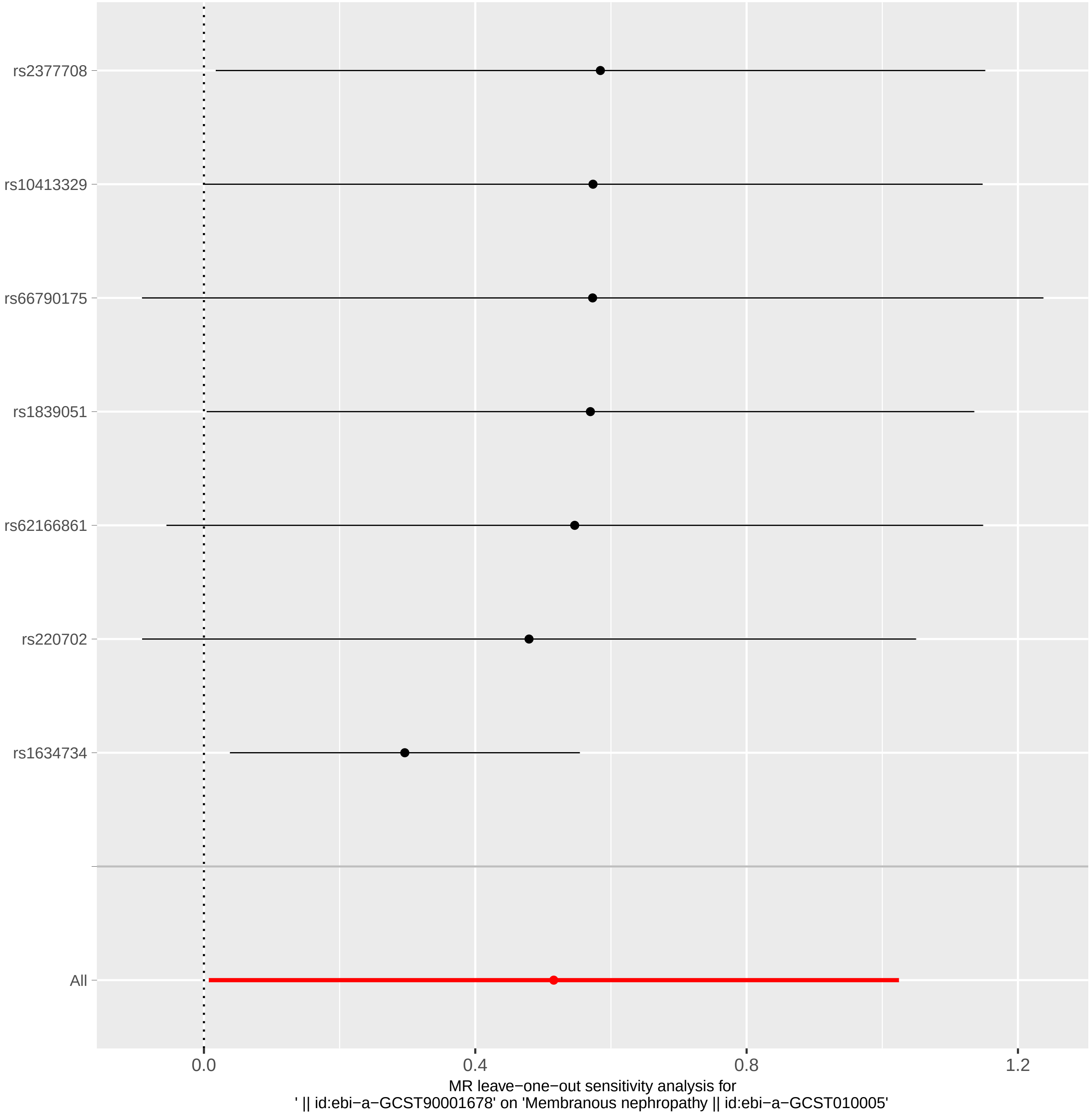

rs2377708
rs10413329
rs66790175
rs1839051
rs62166861
rs220702
rs1634734
All
0.0
0.4
0.8
1.2
MR leave−one−out sensitivity analysis for
' || id:ebi−a−GCST90001678' on 'Membranous nephropathy || id:ebi−a−GCST010005'

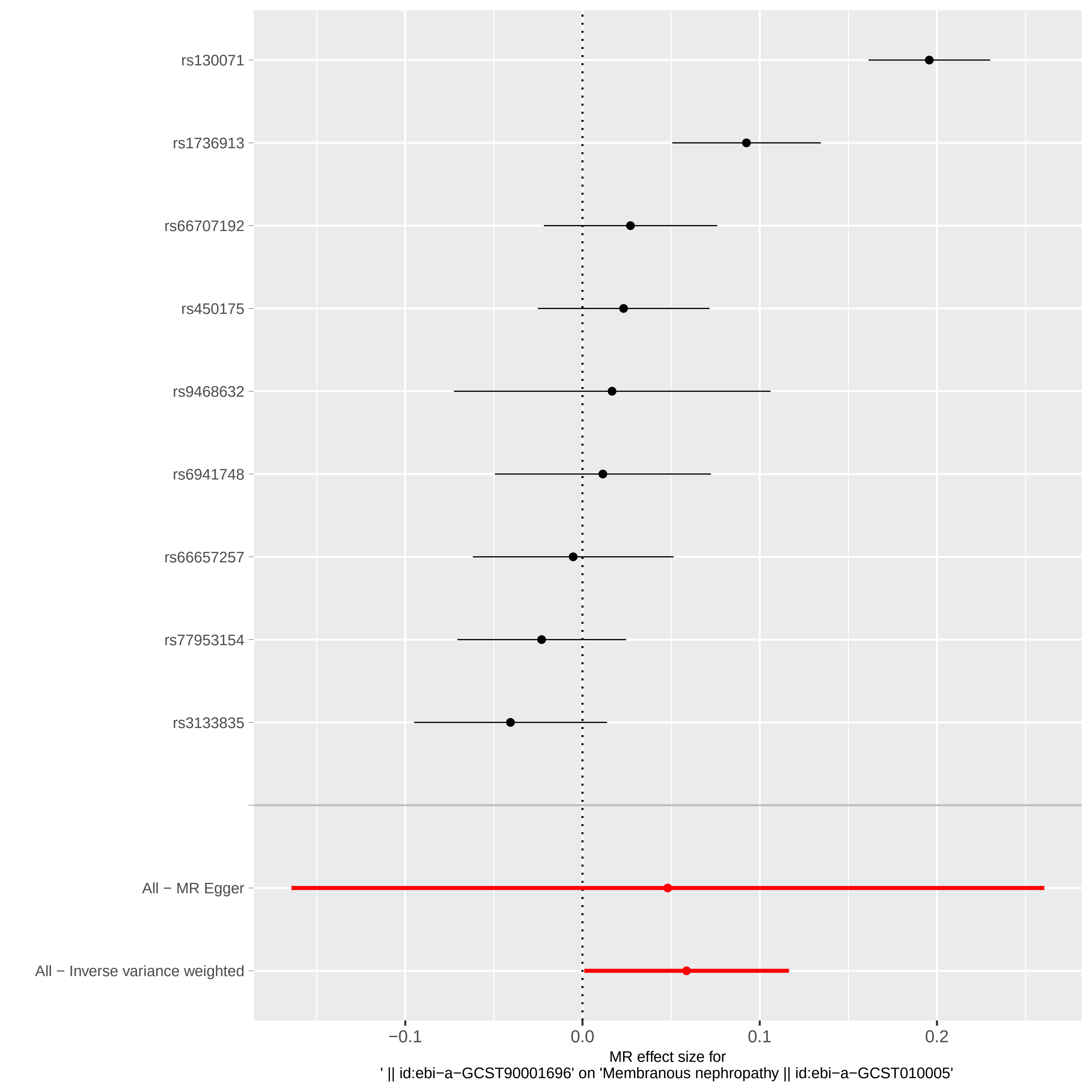

rs130071
rs1736913
rs66707192
rs450175
rs9468632
rs6941748
rs66657257
rs77953154
rs3133835
All − MR Egger
All − Inverse variance weighted
−0.1
0.0
0.1
0.2
MR effect size for
' || id:ebi−a−GCST90001696' on 'Membranous nephropathy || id:ebi−a−GCST010005'

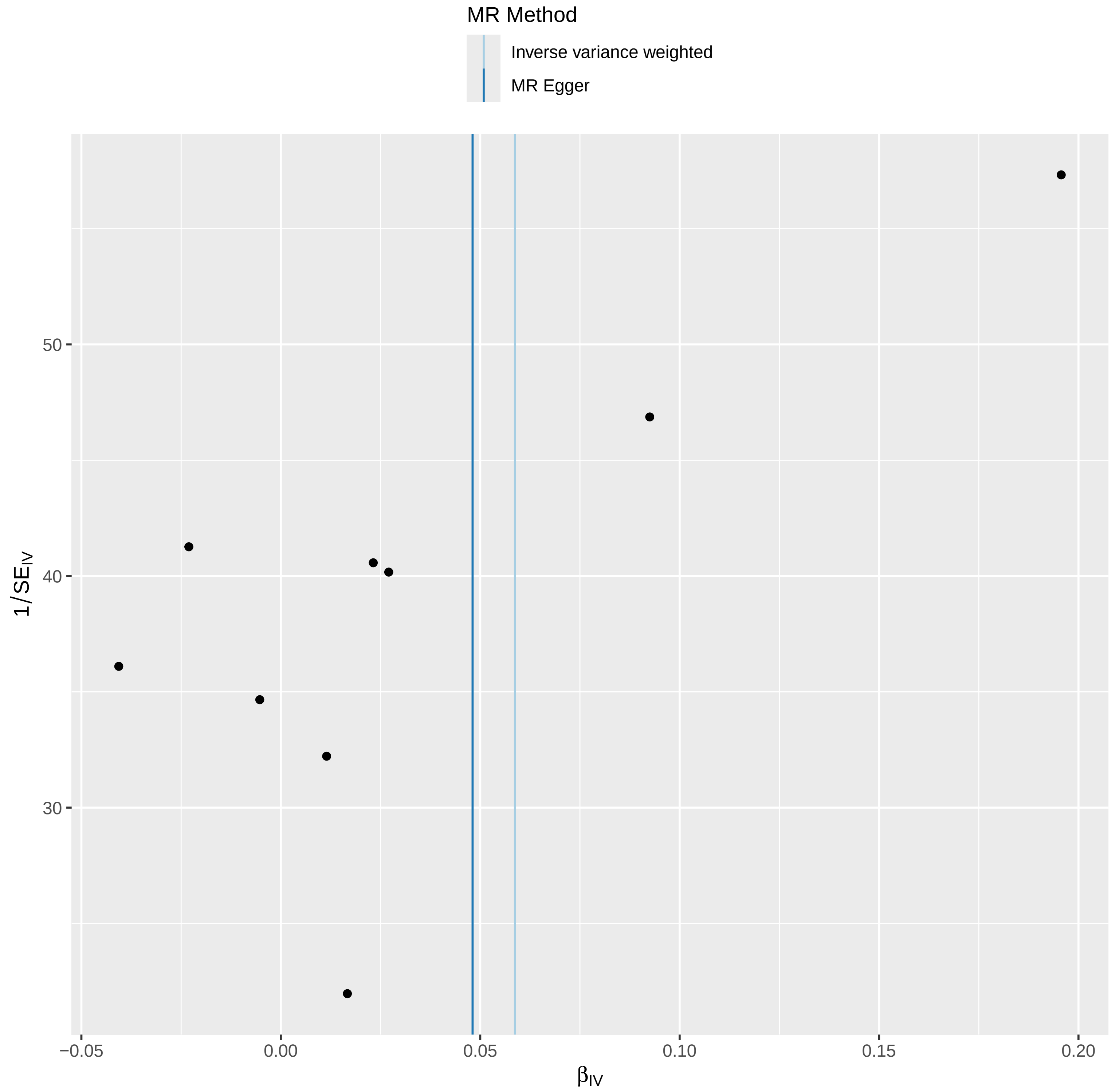
MR Method
Inverse variance weighted
MR Egger
50
40
30
1/SE$_{IV}$
−0.05
0.00
0.05
0.10
0.15
0.20
β$_{IV}$

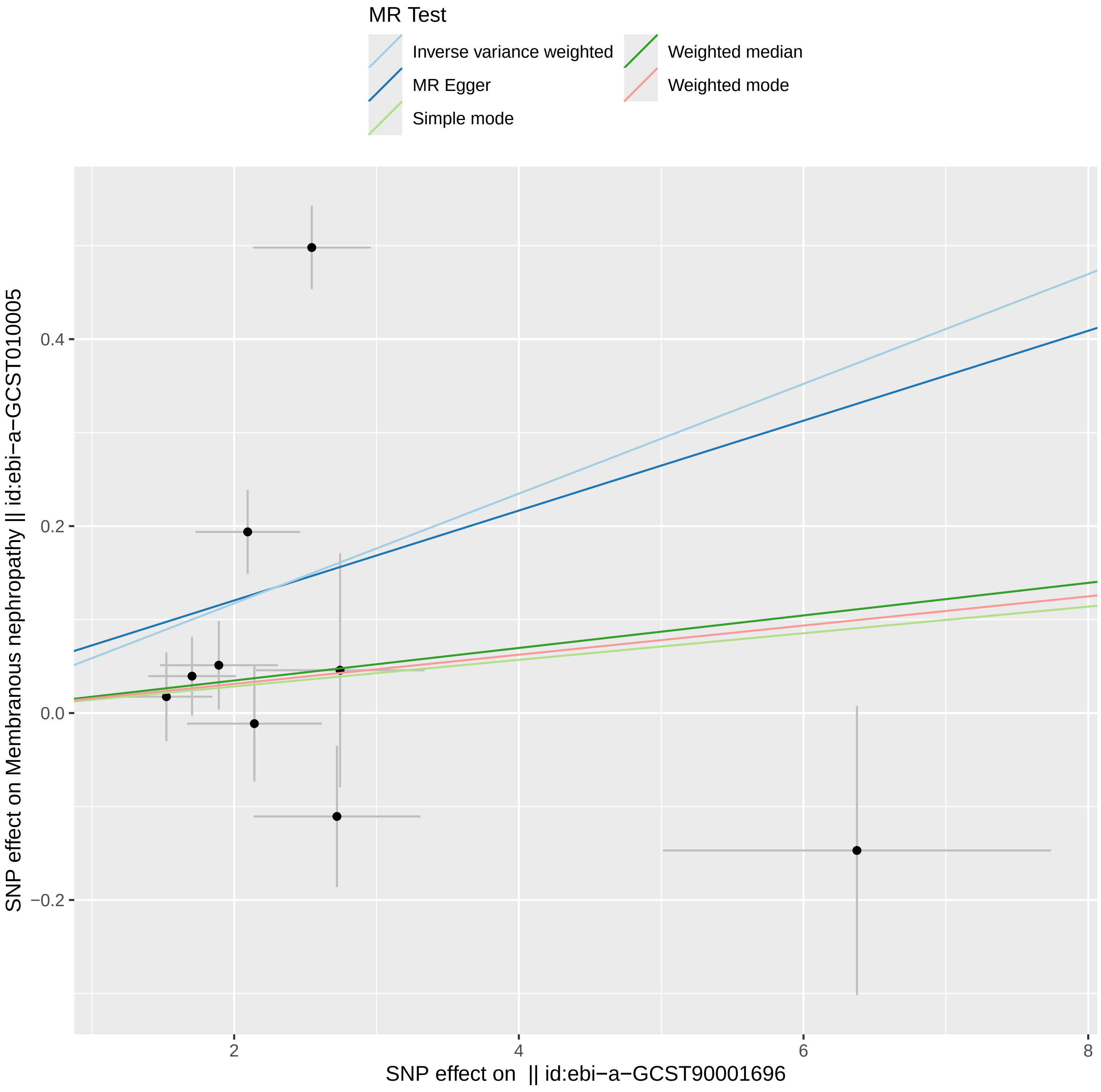

MR Test
Inverse variance weighted
MR Egger
Simple mode
Weighted median
Weighted mode
SNP effect on Membranous nephropathy || id:ebi−a−GCST010005
0.4
0.2
0.0
−0.2
2
4
6
8
SNP effect on || id:ebi−a−GCST90001696

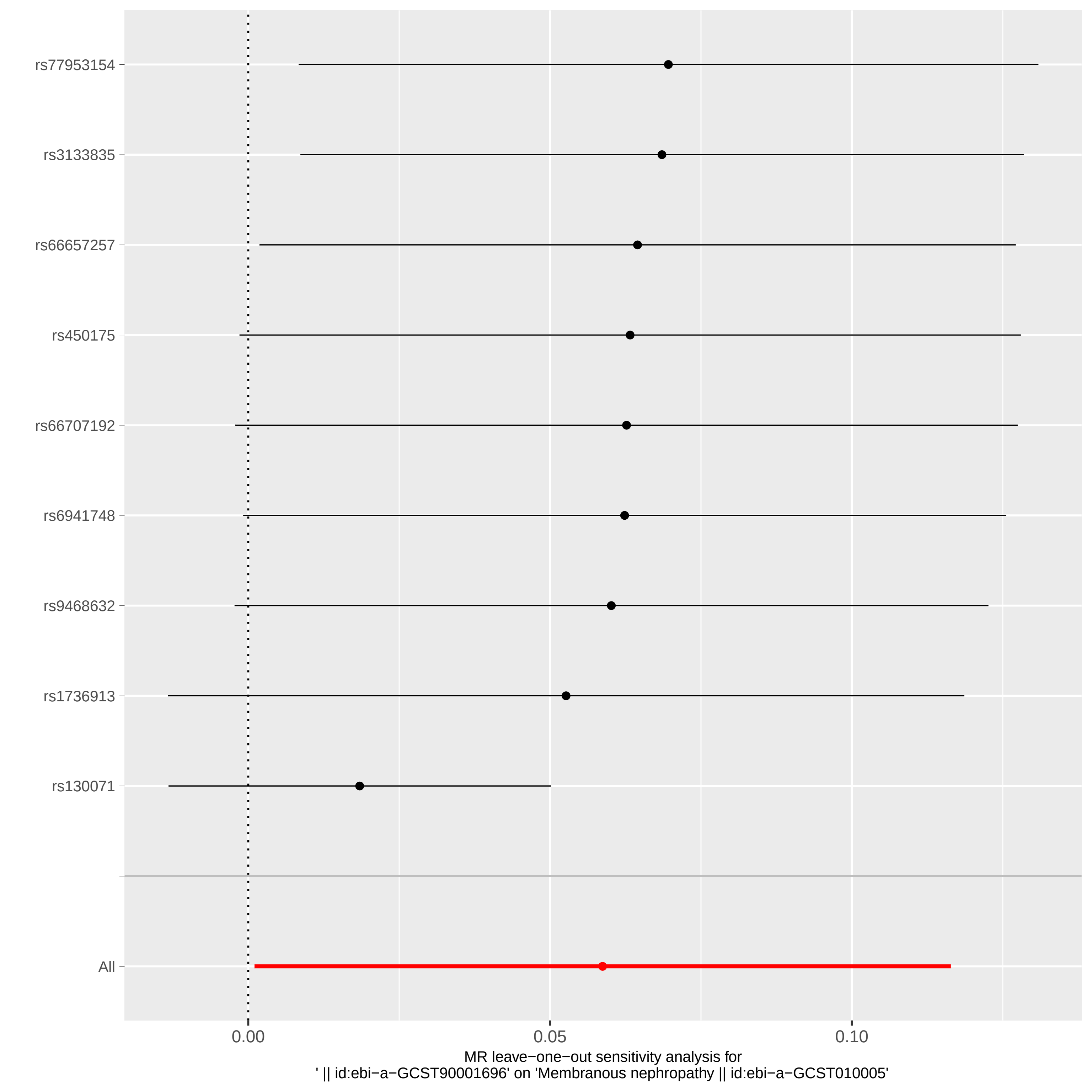
rs77953154
rs3133835
rs66657257
rs450175
rs66707192
rs6941748
rs9468632
rs1736913
rs130071
All
0.00
0.05
0.10
MR leave−one−out sensitivity analysis for
' || id:ebi−a−GCST90001696' on 'Membranous nephropathy || id:ebi−a−GCST010005'

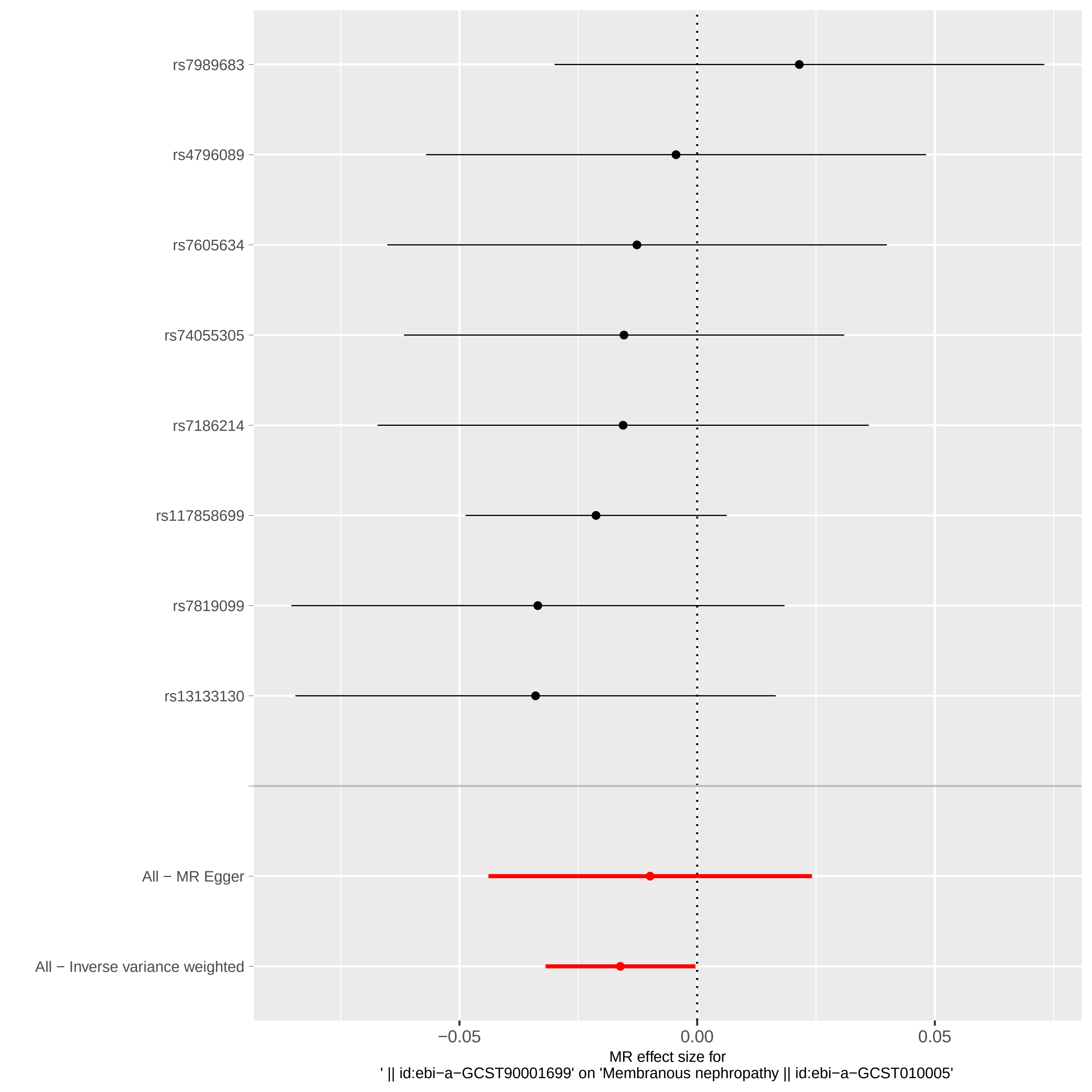
rs7989683
rs4796089
rs7605634
rs74055305
rs7186214
rs117858699
rs7819099
rs13133130
All − MR Egger
All − Inverse variance weighted
−0.05
0.00
0.05
MR effect size for
' || id:ebi−a−GCST90001699' on 'Membranous nephropathy || id:ebi−a−GCST010005'

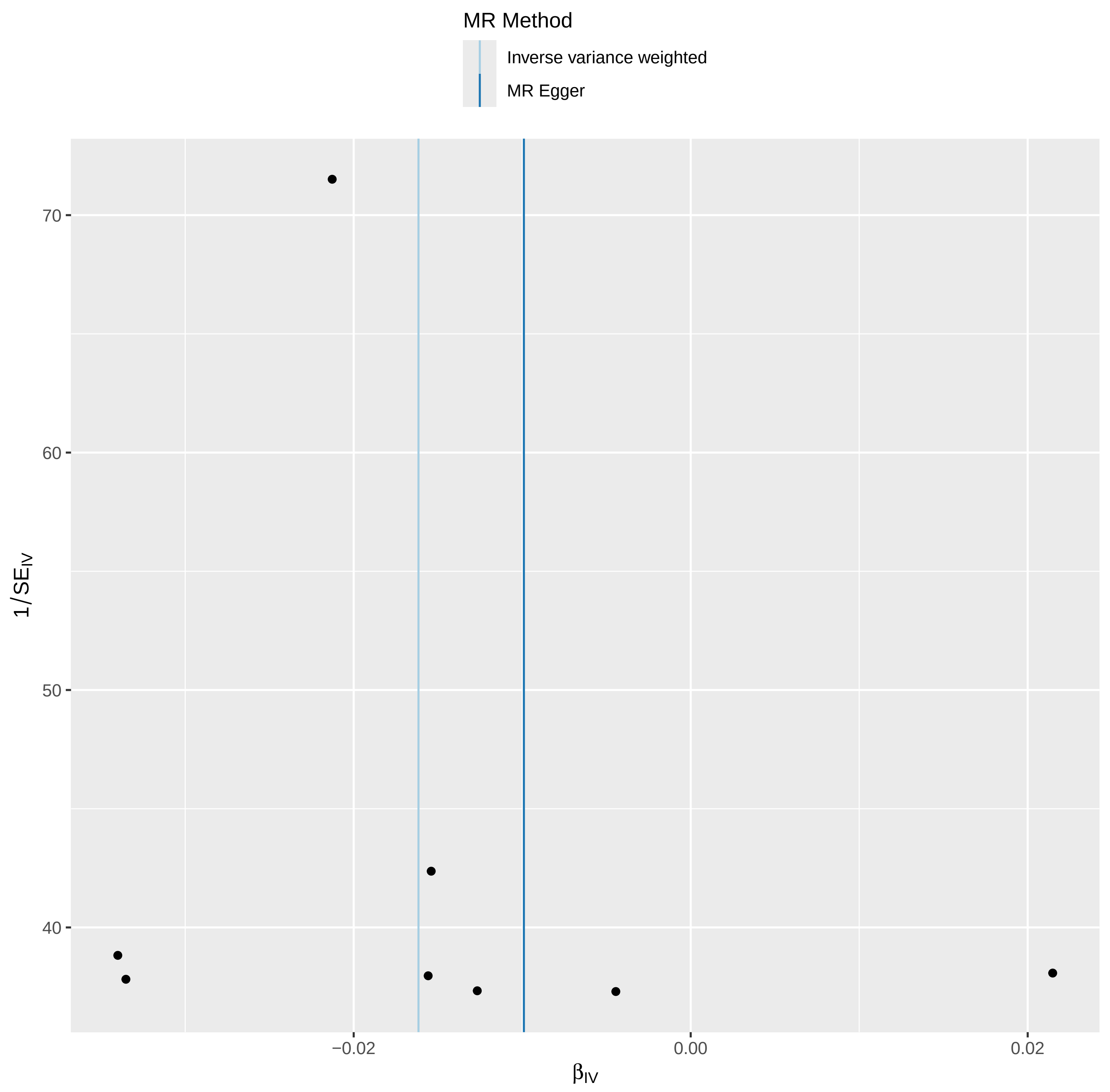
MR Method
Inverse variance weighted
MR Egger
70
60
50
40
1/SE$_{IV}$
−0.02
0.00
0.02
β$_{IV}$

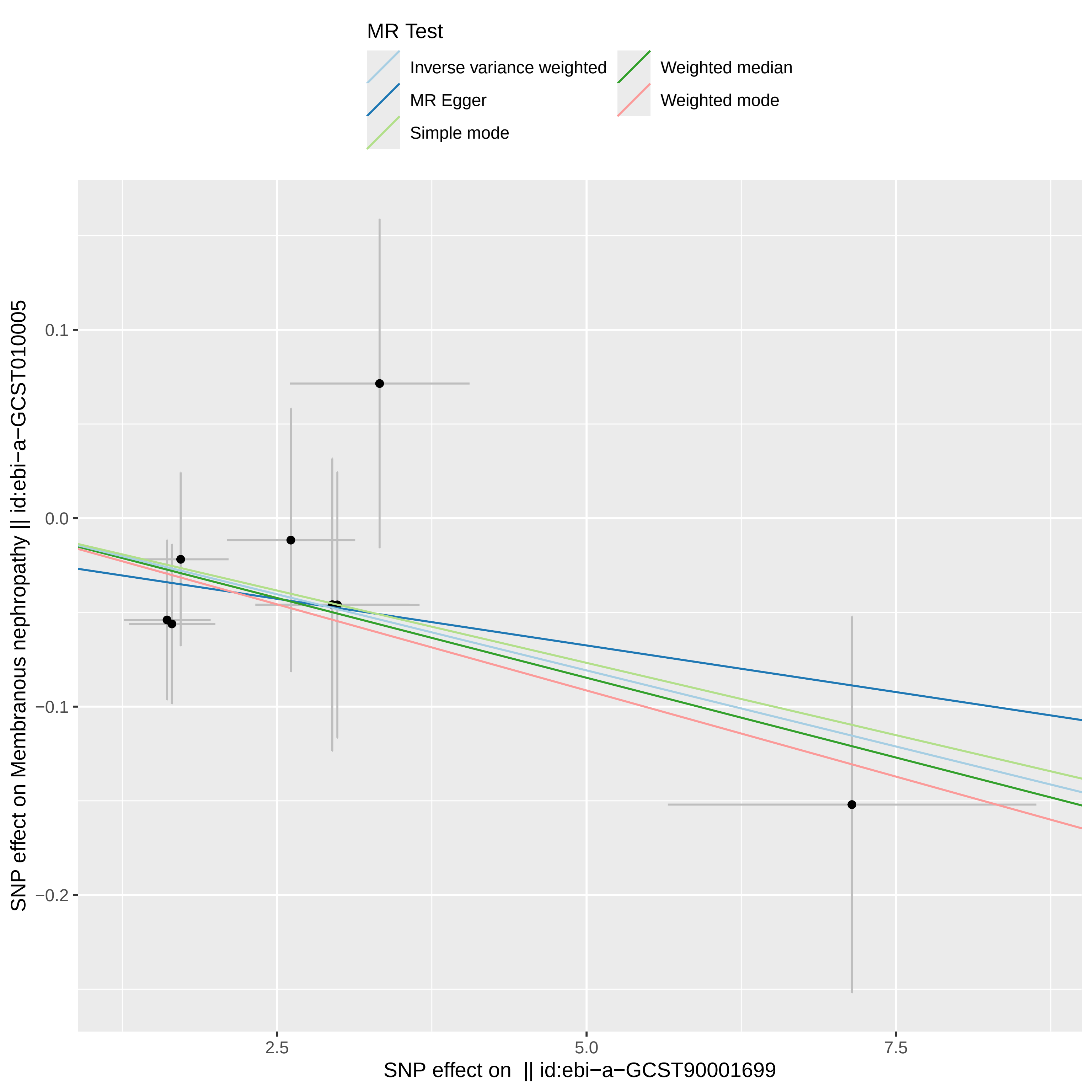

MR Test
Inverse variance weighted
MR Egger
Simple mode
Weighted median
Weighted mode
SNP effect on Membranous nephropathy || id:ebi−a−GCST010005
0.1
0.0
−0.1
−0.2
2.5
5.0
7.5
SNP effect on || id:ebi−a−GCST90001699

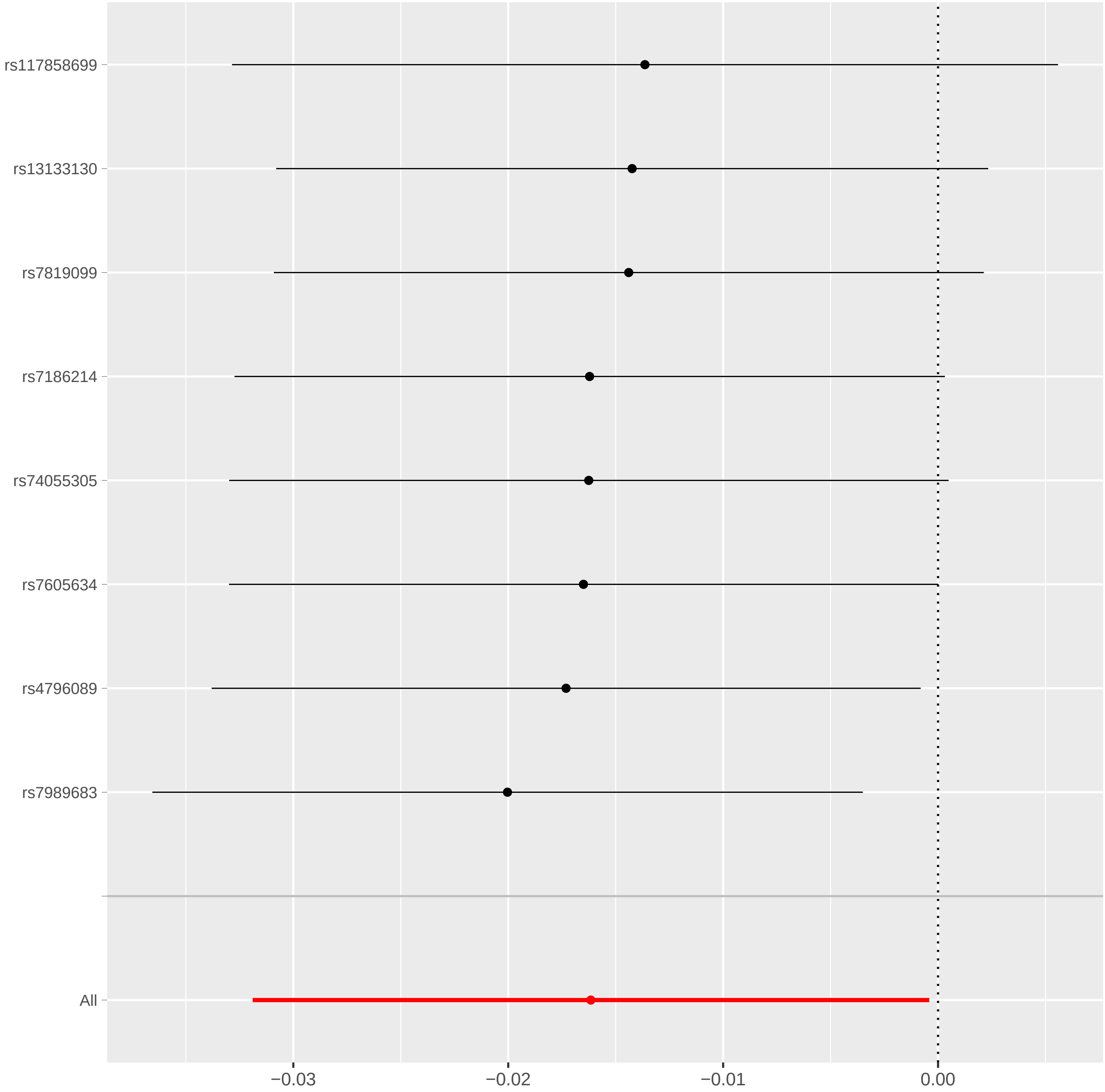

MR leave−one−out sensitivity analysis for
' || id:ebi−a−GCST90001699' on 'Membranous nephropathy || id:ebi−a−GCST010005'

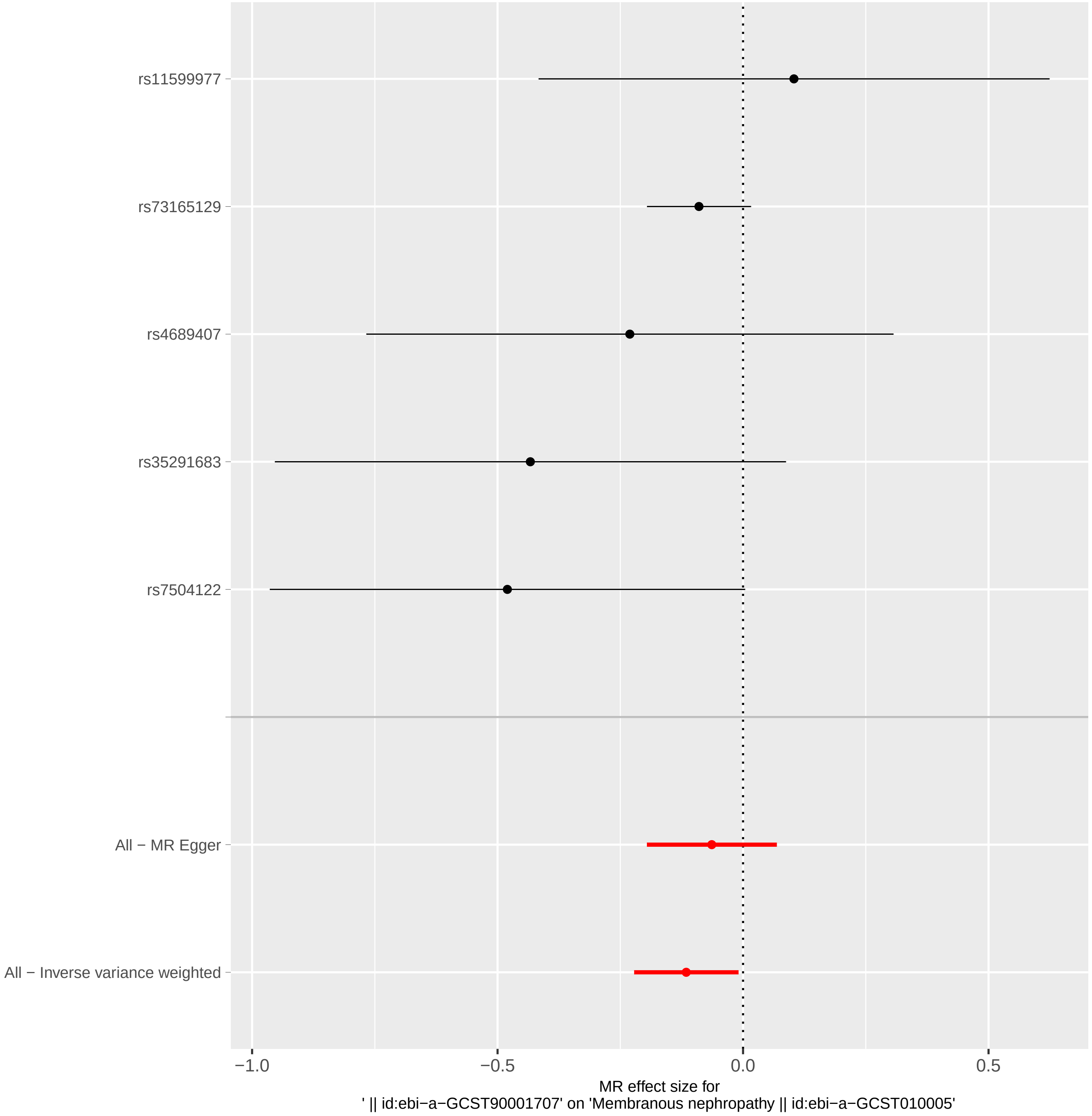
rs11599977
rs73165129
rs4689407
rs35291683
rs7504122
All − MR Egger
All − Inverse variance weighted
−1.0
−0.5
0.0
0.5
MR effect size for
' || id:ebi−a−GCST90001707' on 'Membranous nephropathy || id:ebi−a−GCST010005'

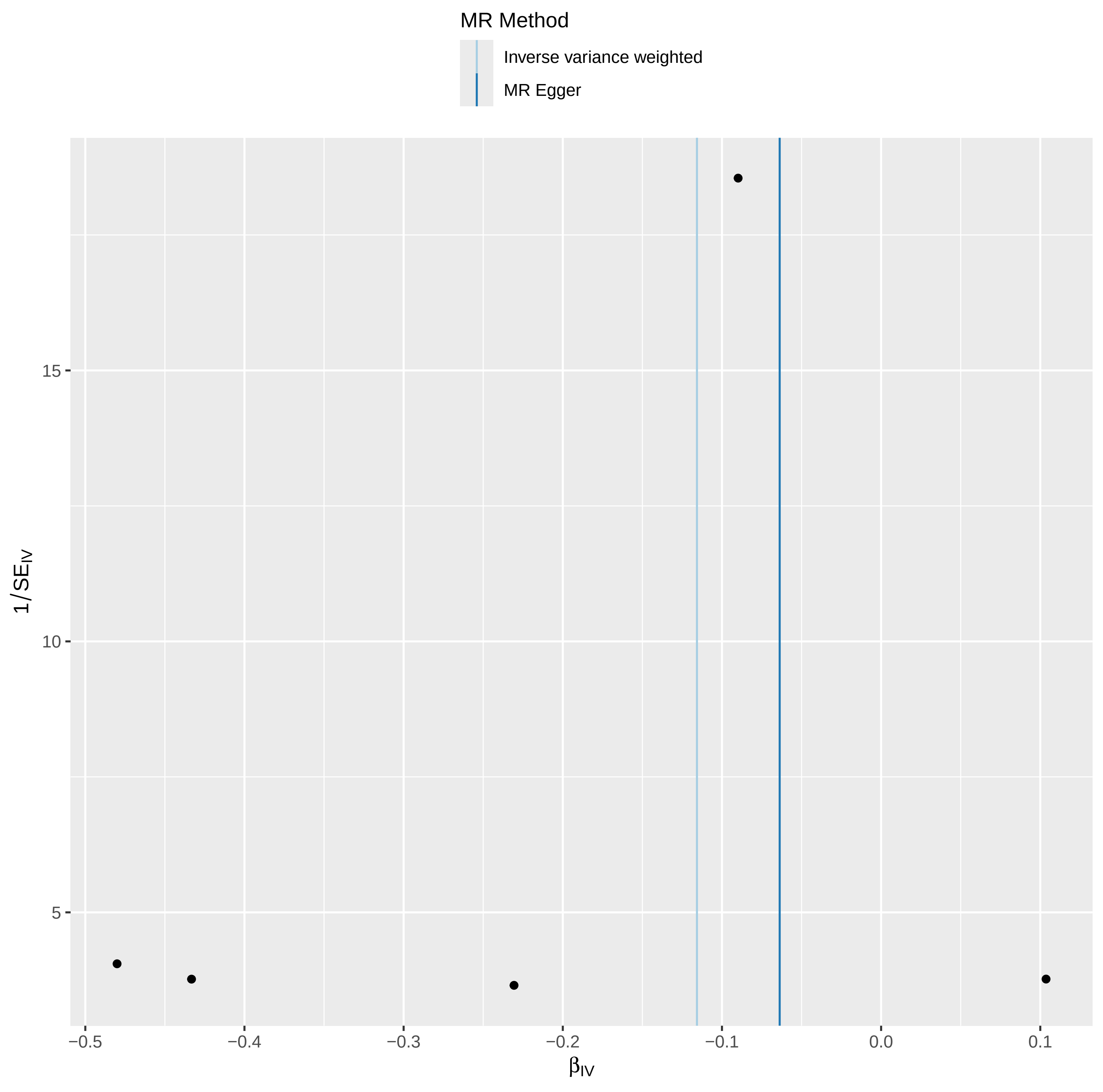

MR Method
Inverse variance weighted
MR Egger
15
10
5
1/SE$_{IV}$
−0.5
−0.4
−0.3
−0.2
−0.1
0.0
0.1
β$_{IV}$

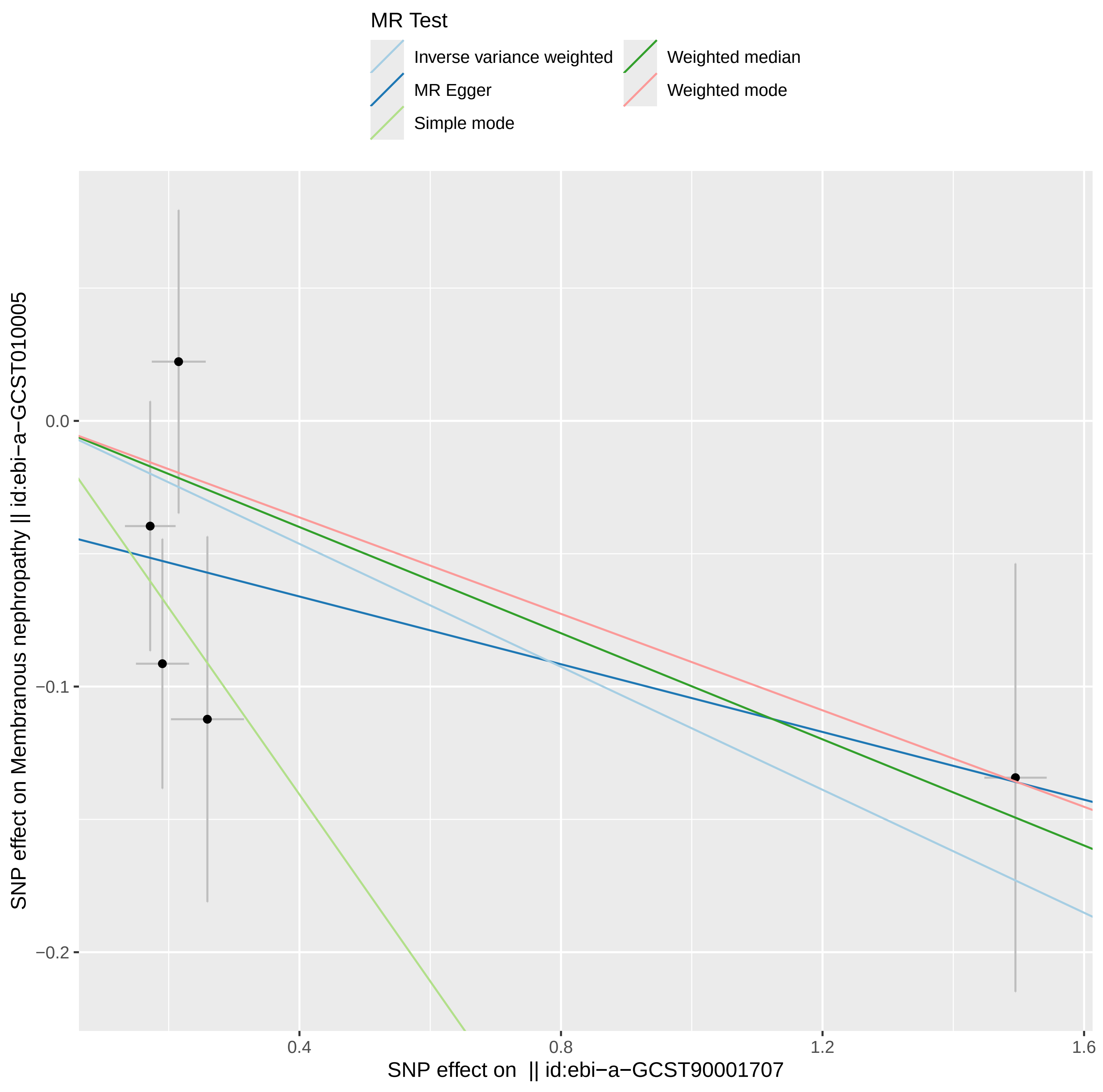
MR Test
Inverse variance weighted
MR Egger
Simple mode
Weighted median
Weighted mode
SNP effect on Membranous nephropathy || id:ebi−a−GCST010005
0.0
−0.1
−0.2
0.4
0.8
1.2
1.6
SNP effect on || id:ebi−a−GCST90001707

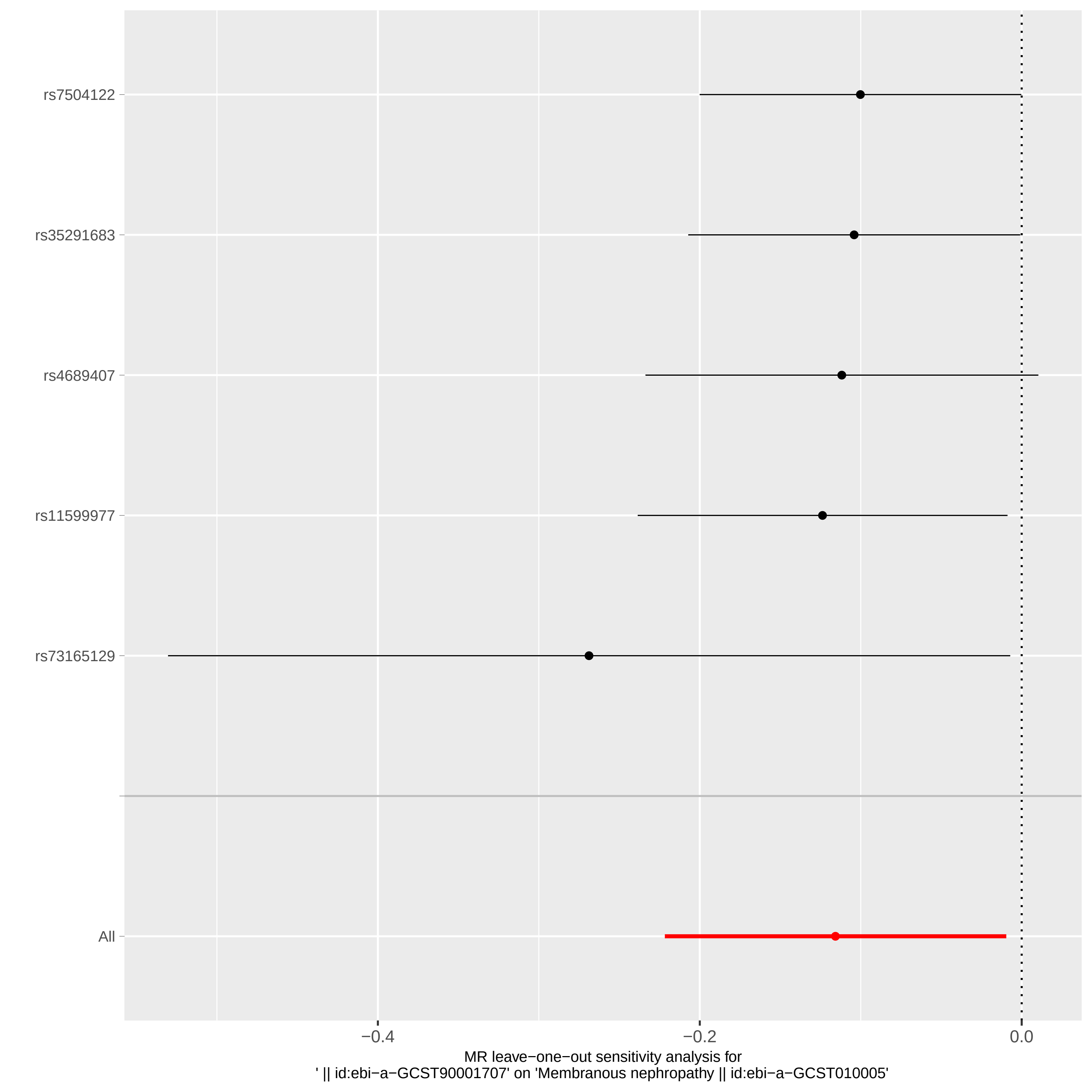
rs7504122
rs35291683
rs4689407
rs11599977
rs73165129
All
−0.4
−0.2
0.0
MR leave−one−out sensitivity analysis for
' || id:ebi−a−GCST90001707' on 'Membranous nephropathy || id:ebi−a−GCST010005'

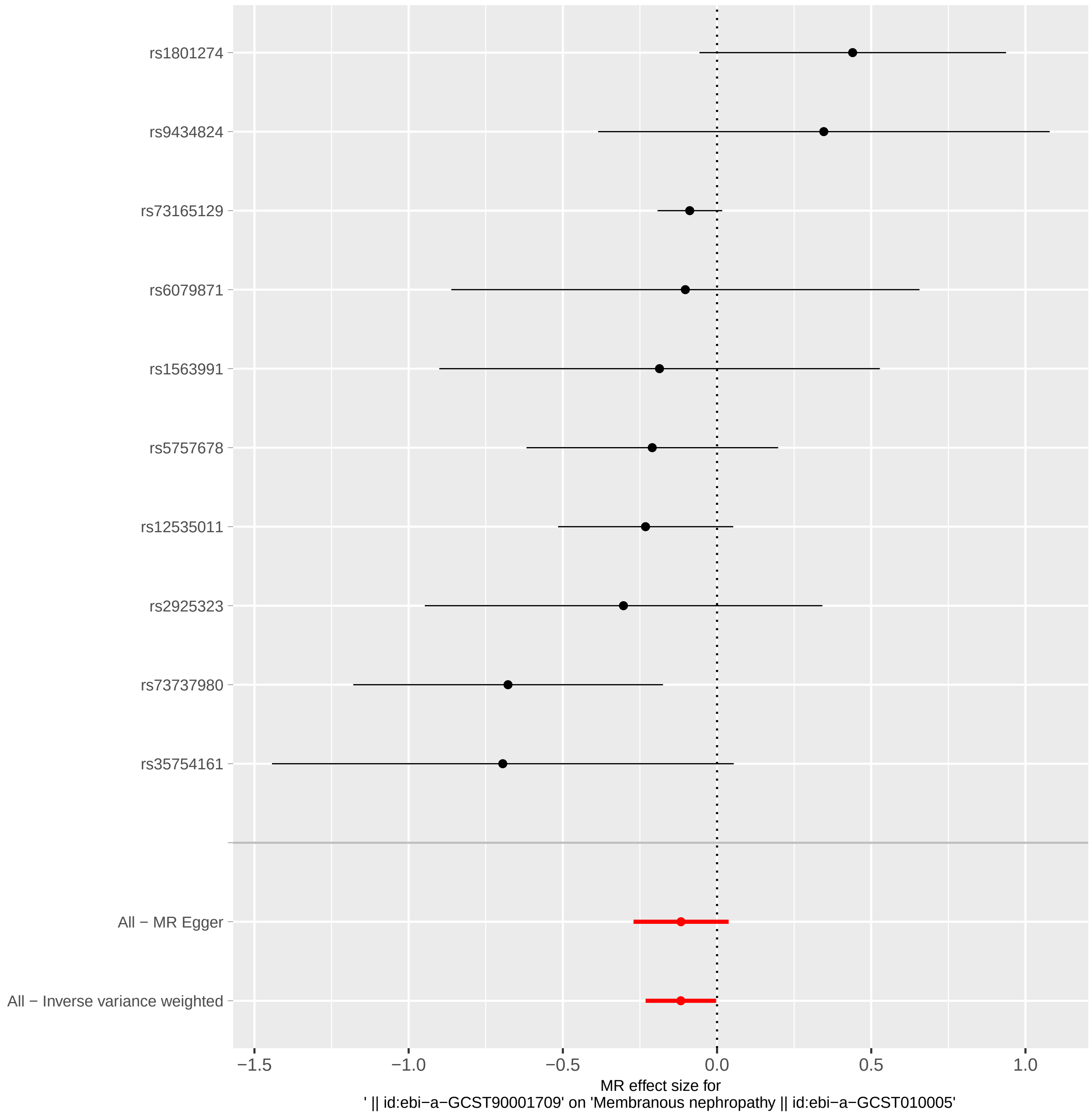
rs1801274
rs9434824
rs73165129
rs6079871
rs1563991
rs5757678
rs12535011
rs2925323
rs73737980
rs35754161
All − MR Egger
All − Inverse variance weighted
−1.5
−1.0
−0.5
0.0
0.5
1.0
MR effect size for
' || id:ebi−a−GCST90001709' on 'Membranous nephropathy || id:ebi−a−GCST010005'

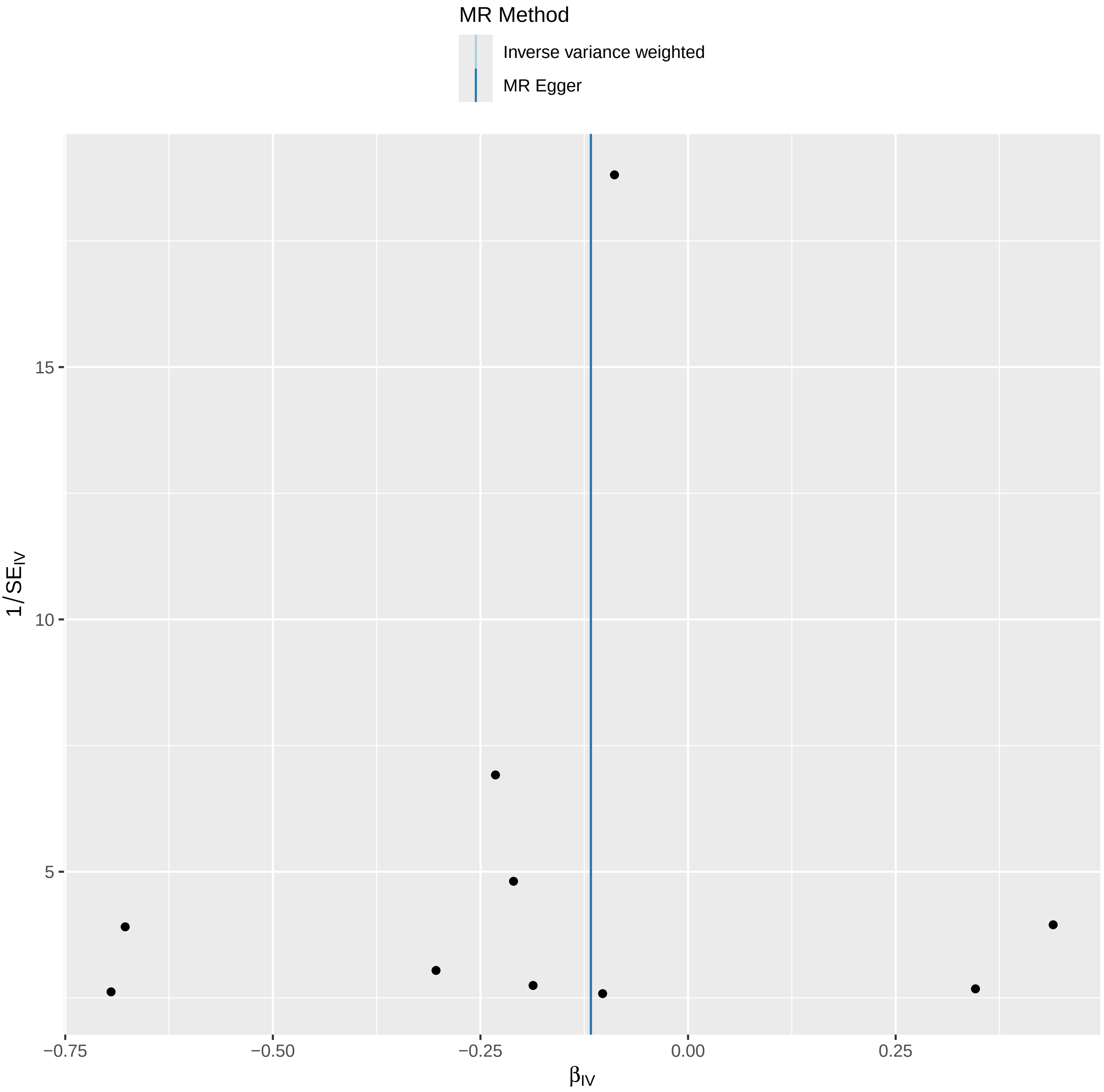
MR Method
Inverse variance weighted
MR Egger
1/SE$_{IV}$
15
10
5
−0.75
−0.50
−0.25
0.00
0.25
β$_{IV}$

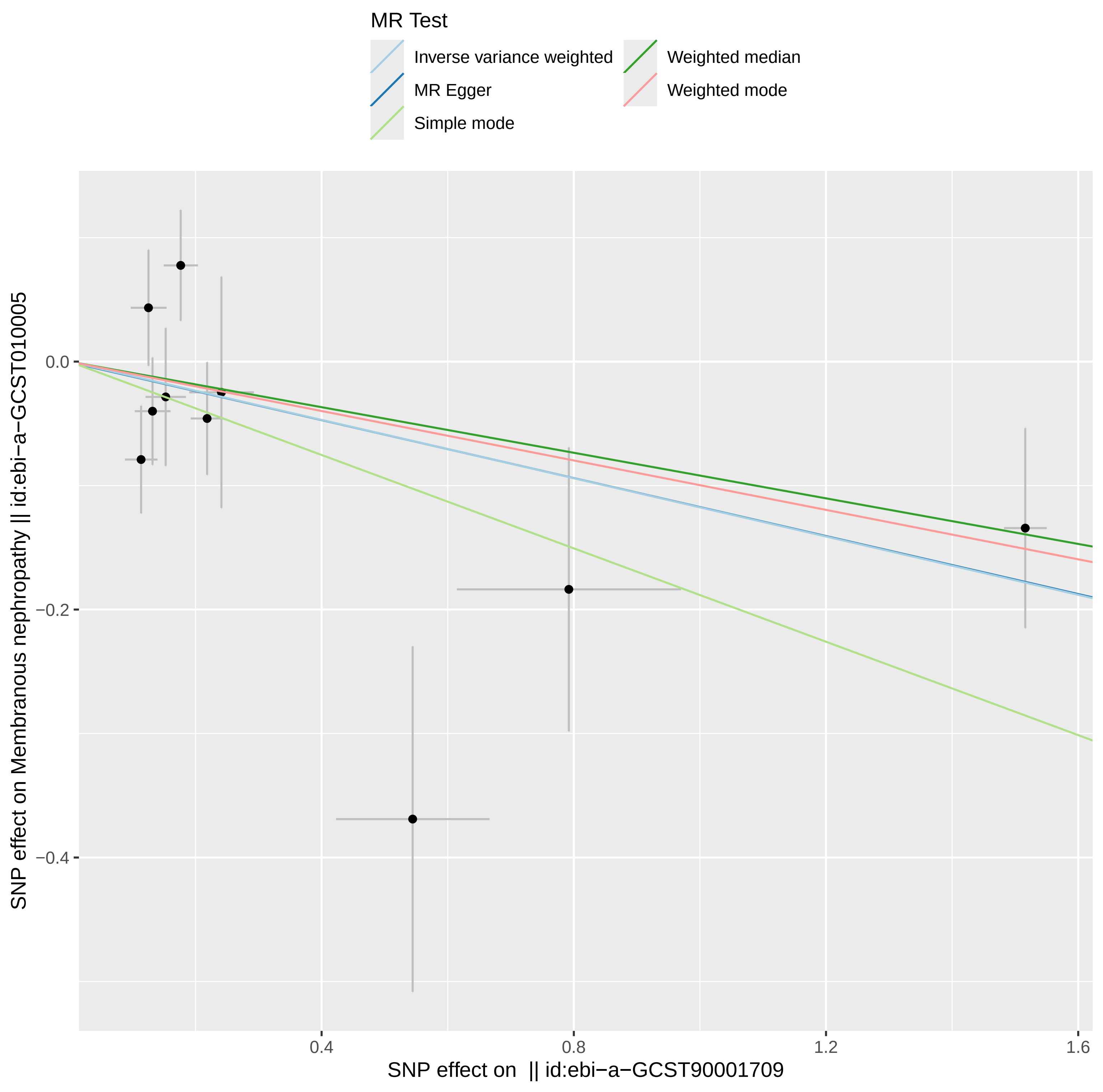

MR Test
Inverse variance weighted
MR Egger
Simple mode
Weighted median
Weighted mode
SNP effect on Membranous nephropathy || id:ebi−a−GCST010005
0.0
−0.2
−0.4
0.4
0.8
1.2
1.6
SNP effect on || id:ebi−a−GCST90001709

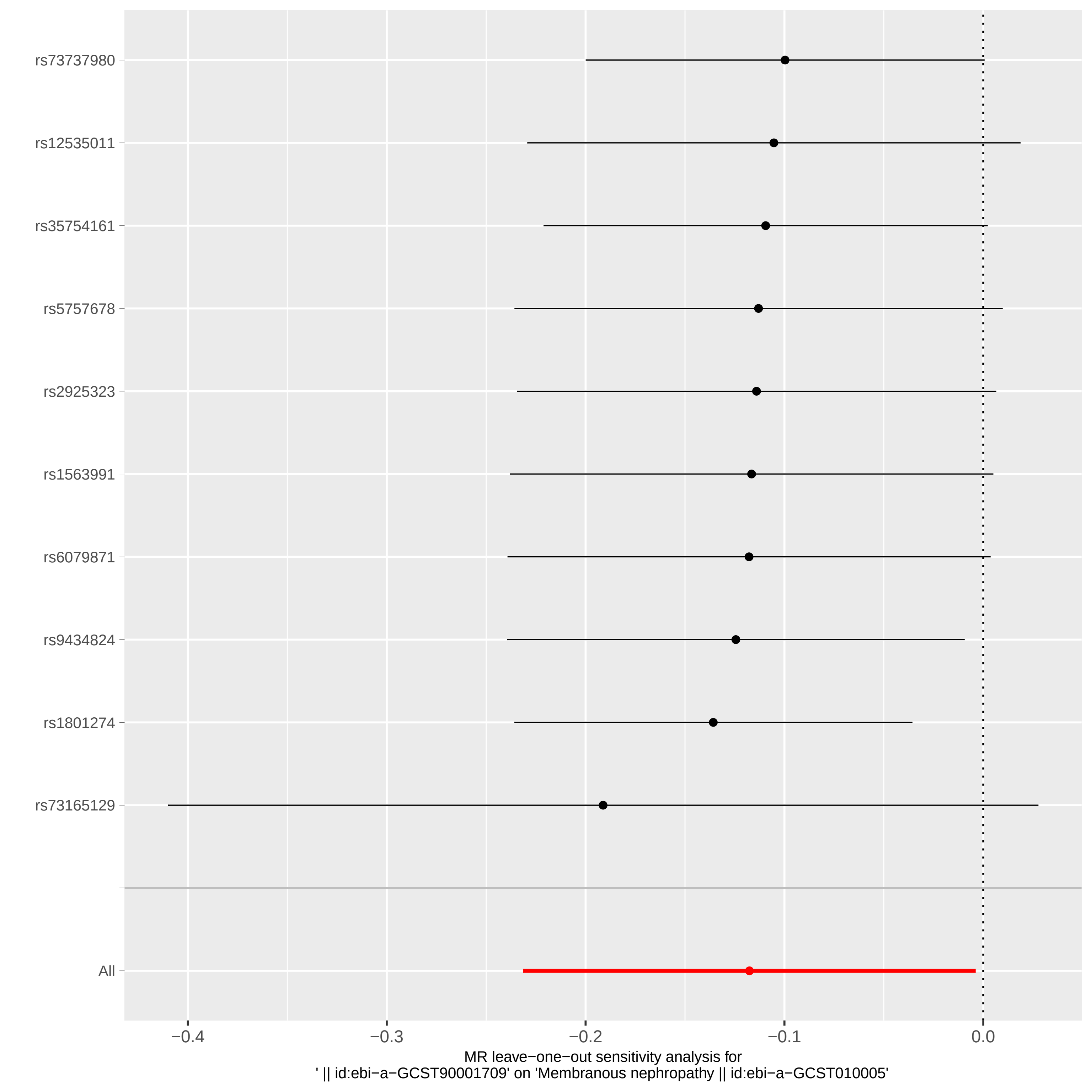
rs73737980
rs12535011
rs35754161
rs5757678
rs2925323
rs1563991
rs6079871
rs9434824
rs1801274
rs73165129
All
−0.4
−0.3
−0.2
−0.1
0.0
MR leave−one−out sensitivity analysis for
' || id:ebi−a−GCST90001709' on 'Membranous nephropathy || id:ebi−a−GCST010005'

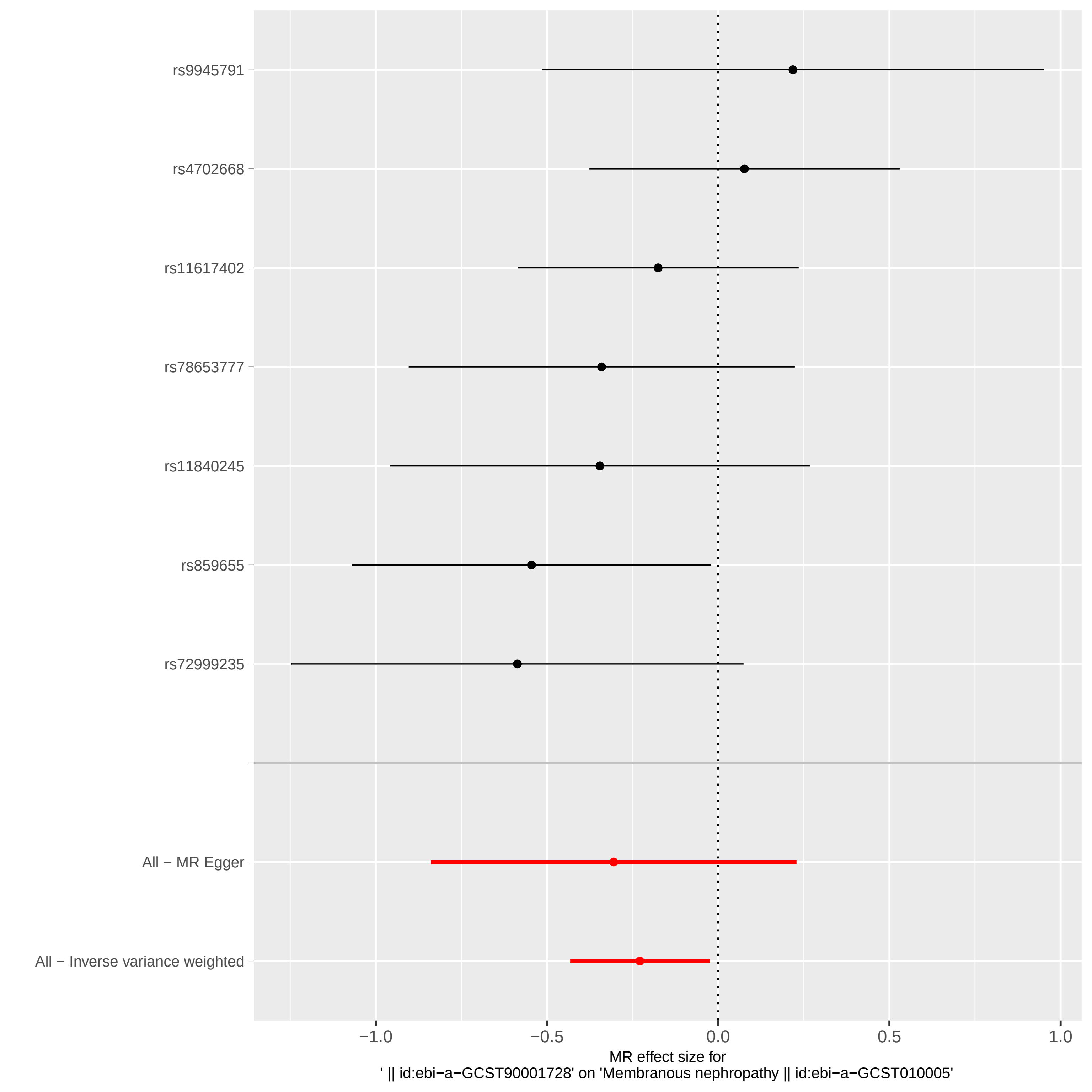
rs9945791
rs4702668
rs11617402
rs78653777
rs11840245
rs859655
rs72999235
All − MR Egger
All − Inverse variance weighted
−1.0
−0.5
0.0
0.5
1.0
MR effect size for
' || id:ebi−a−GCST90001728' on 'Membranous nephropathy || id:ebi−a−GCST010005'

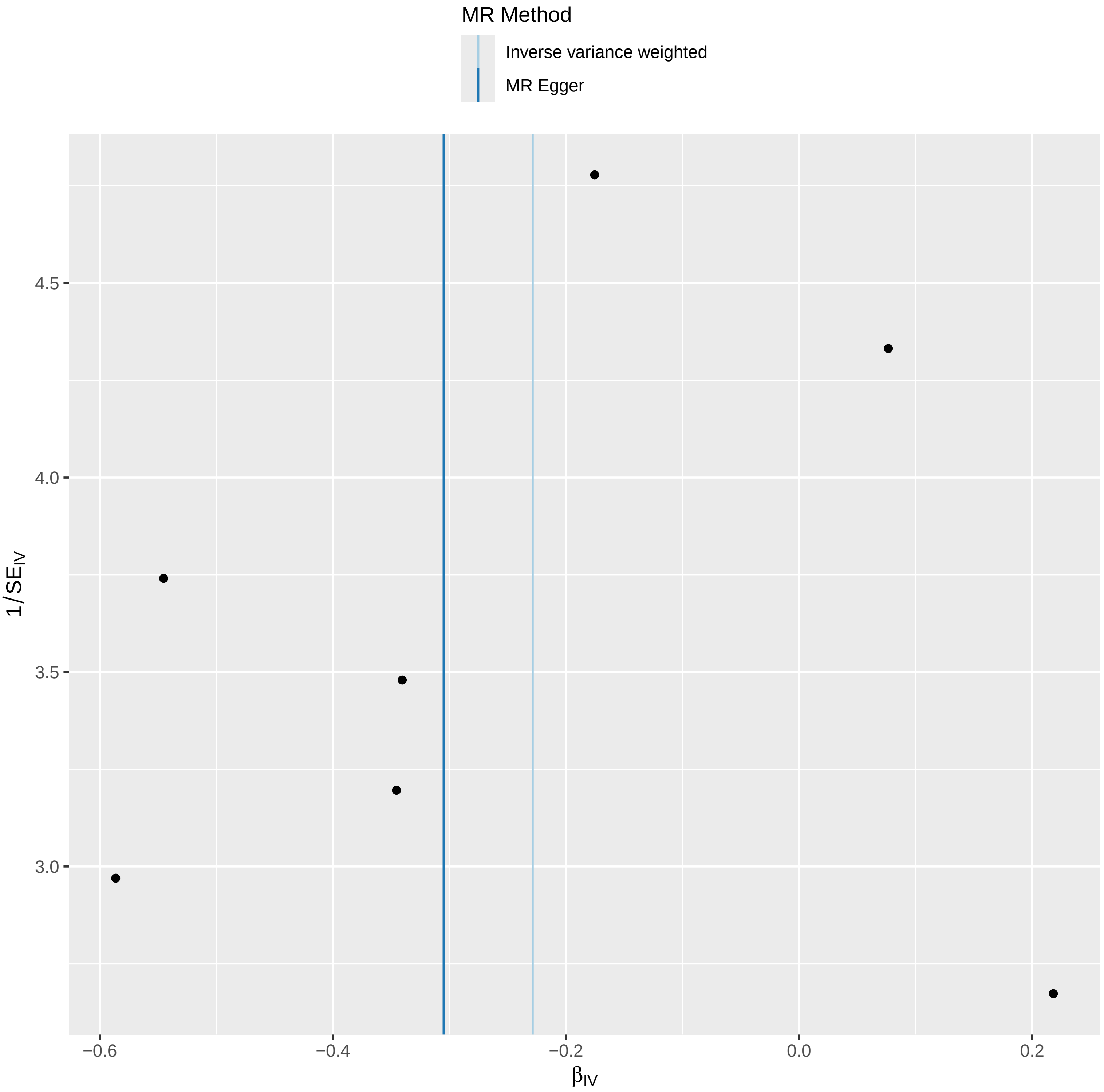

MR Method
Inverse variance weighted
MR Egger
4.5
4.0
3.5
3.0
1/SE_IV
−0.6
−0.4
−0.2
0.0
0.2
β_IV

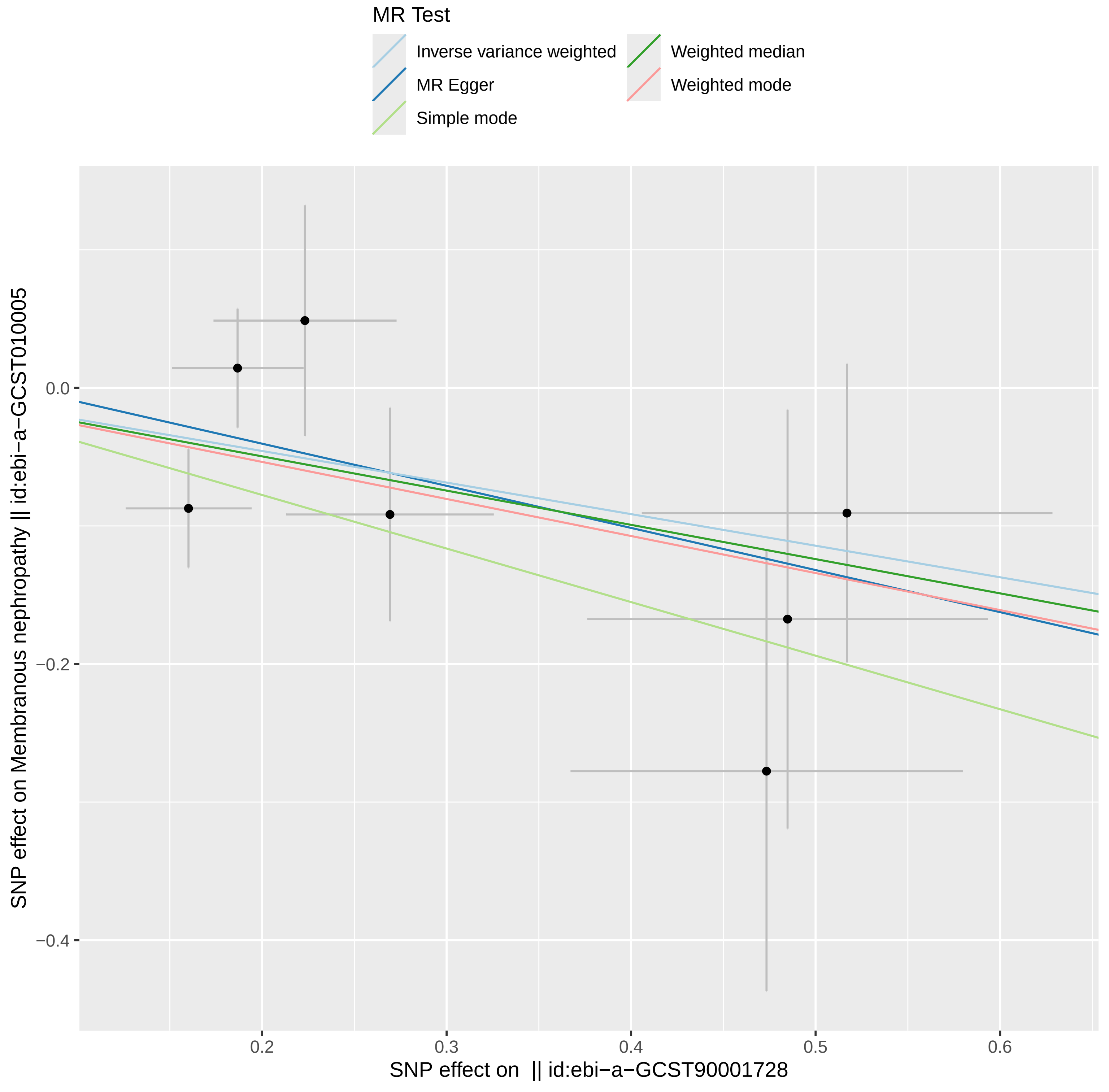
MR Test
Inverse variance weighted
MR Egger
Simple mode
Weighted median
Weighted mode
0.0
−0.2
−0.4
0.2
0.3
0.4
0.5
0.6
SNP effect on Membranous nephropathy || id:ebi−a−GCST010005
SNP effect on || id:ebi−a−GCST90001728

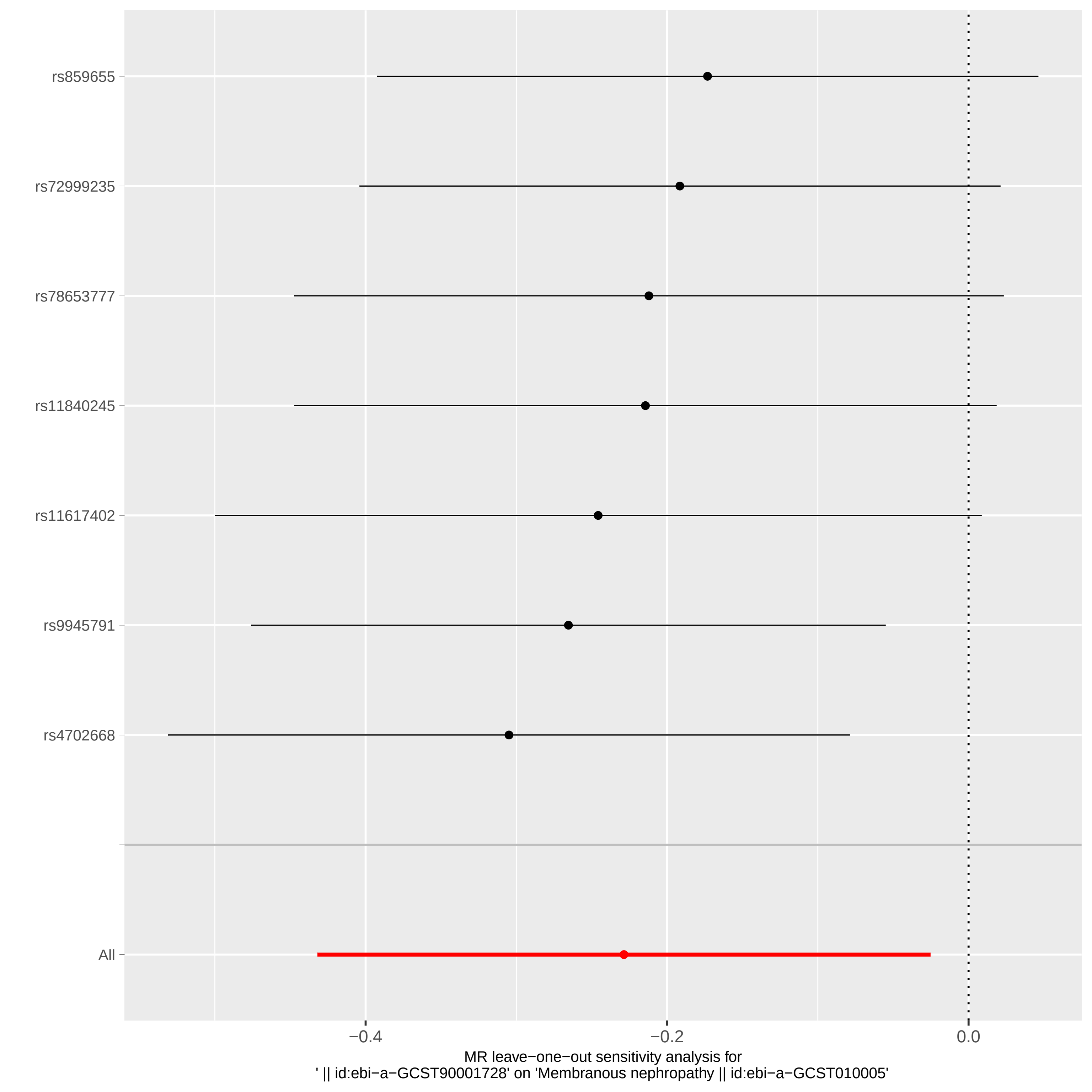

rs859655
rs72999235
rs78653777
rs11840245
rs11617402
rs9945791
rs4702668
All
−0.4
−0.2
0.0
MR leave−one−out sensitivity analysis for
' || id:ebi−a−GCST90001728' on 'Membranous nephropathy || id:ebi−a−GCST010005'

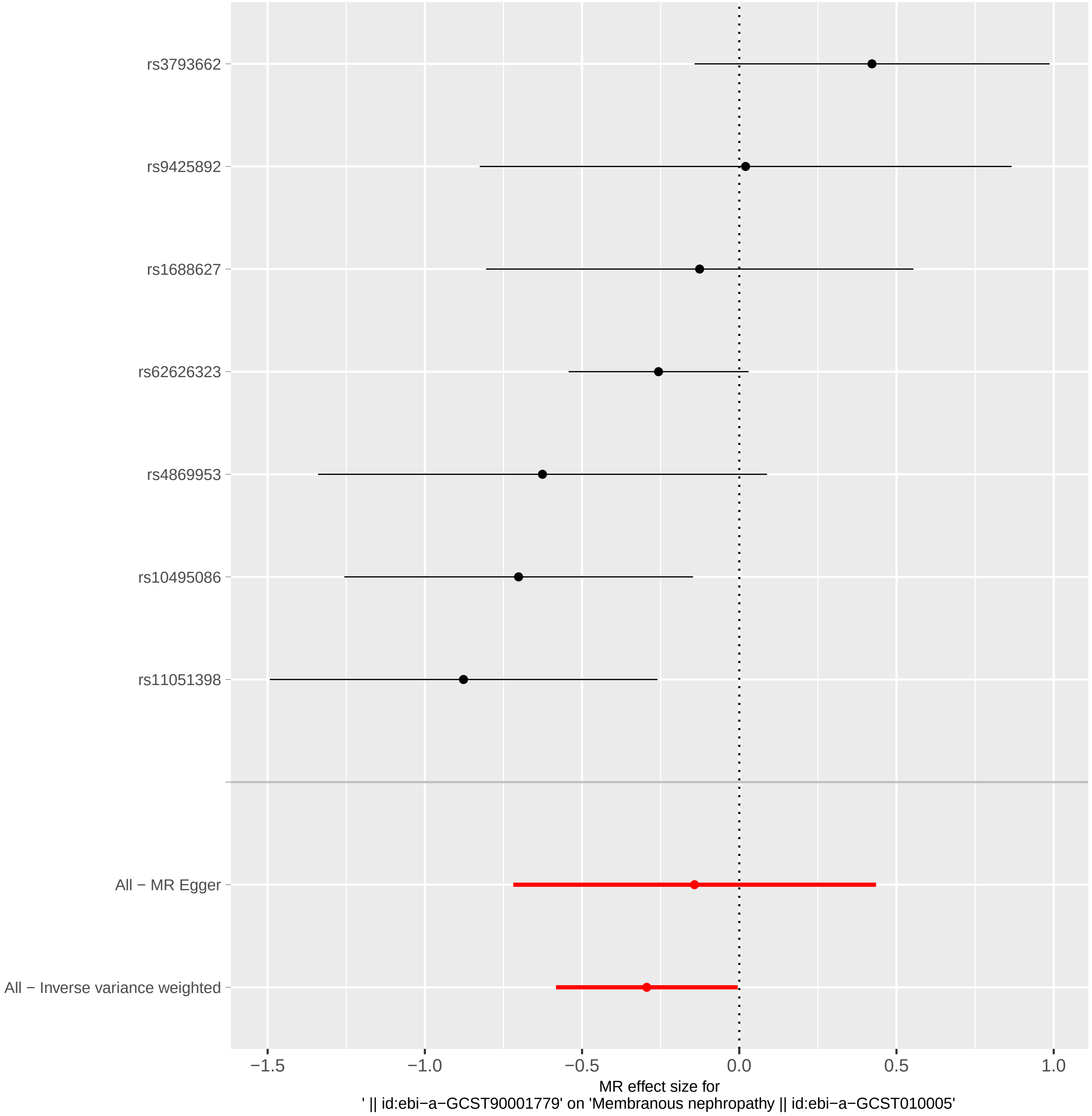
rs3793662
rs9425892
rs1688627
rs62626323
rs4869953
rs10495086
rs11051398
All − MR Egger
All − Inverse variance weighted
−1.5
−1.0
−0.5
0.0
0.5
1.0
MR effect size for
' || id:ebi−a−GCST90001779' on 'Membranous nephropathy || id:ebi−a−GCST010005'

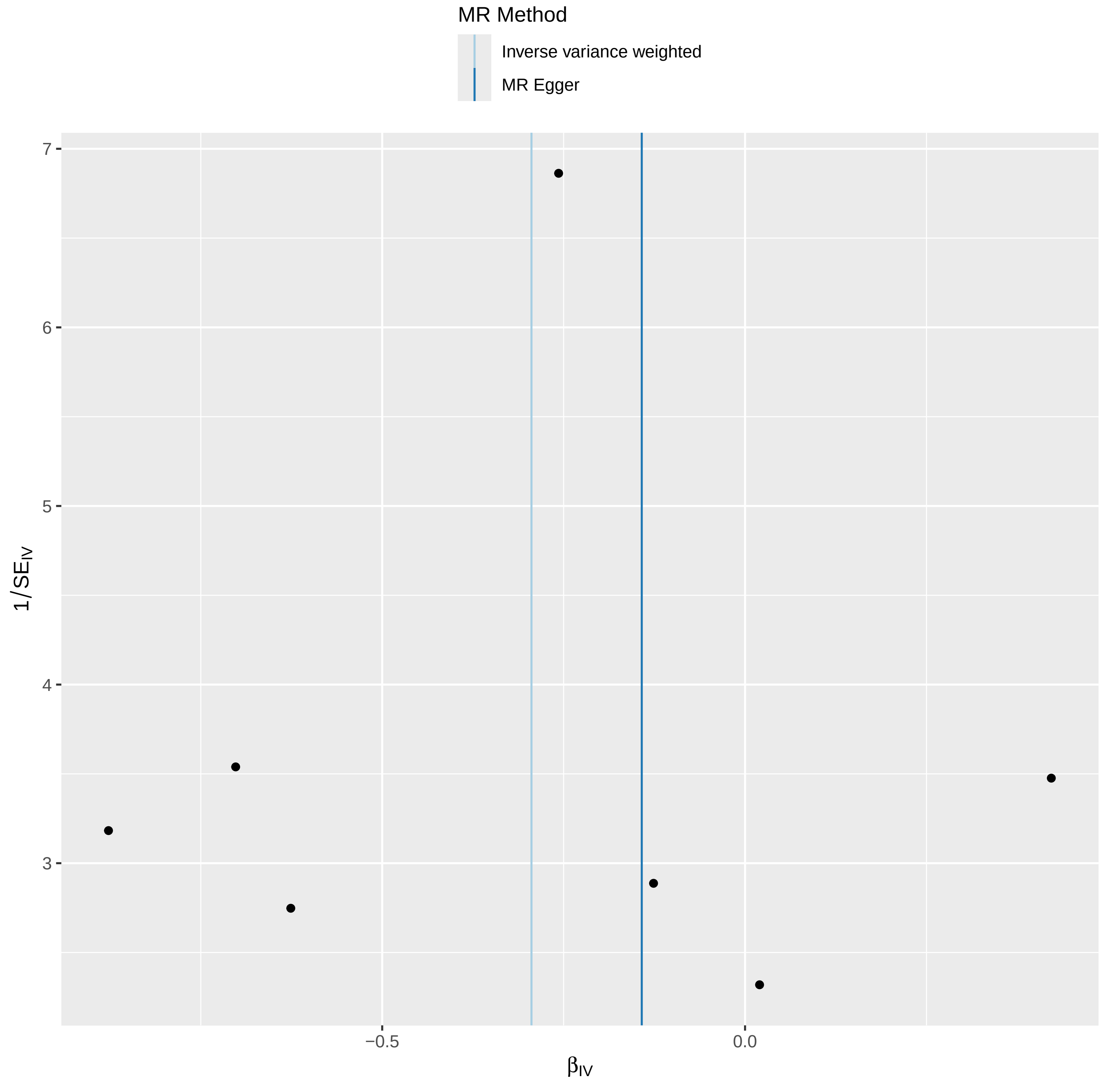

MR Method
Inverse variance weighted
MR Egger
7
6
5
4
3
1/SE_IV
−0.5
0.0
β_IV

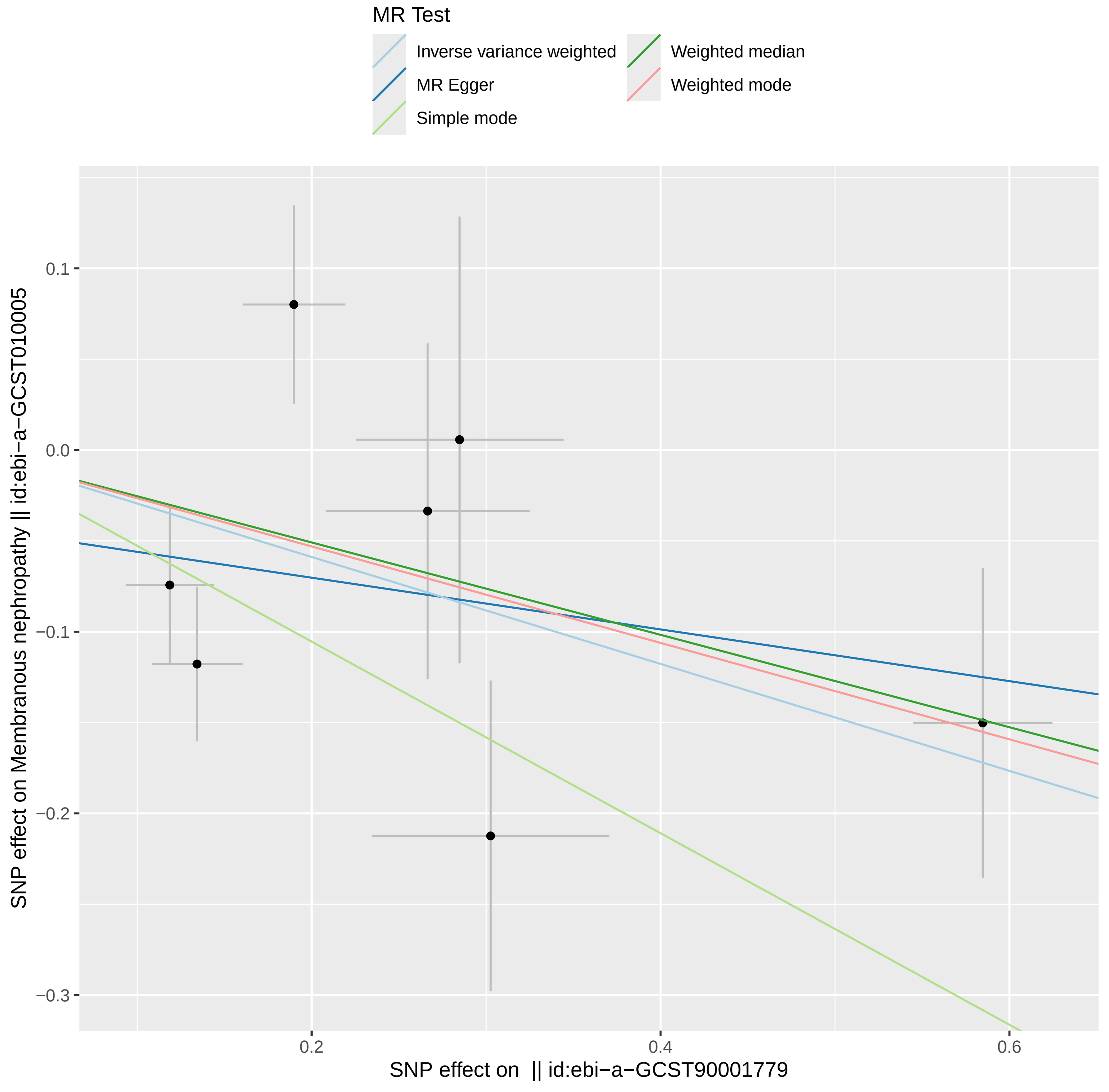
MR Test
Inverse variance weighted
MR Egger
Simple mode
Weighted median
Weighted mode
SNP effect on Membranous nephropathy || id:ebi−a−GCST010005
0.1
0.0
−0.1
−0.2
−0.3
0.2
0.4
0.6
SNP effect on || id:ebi−a−GCST90001779

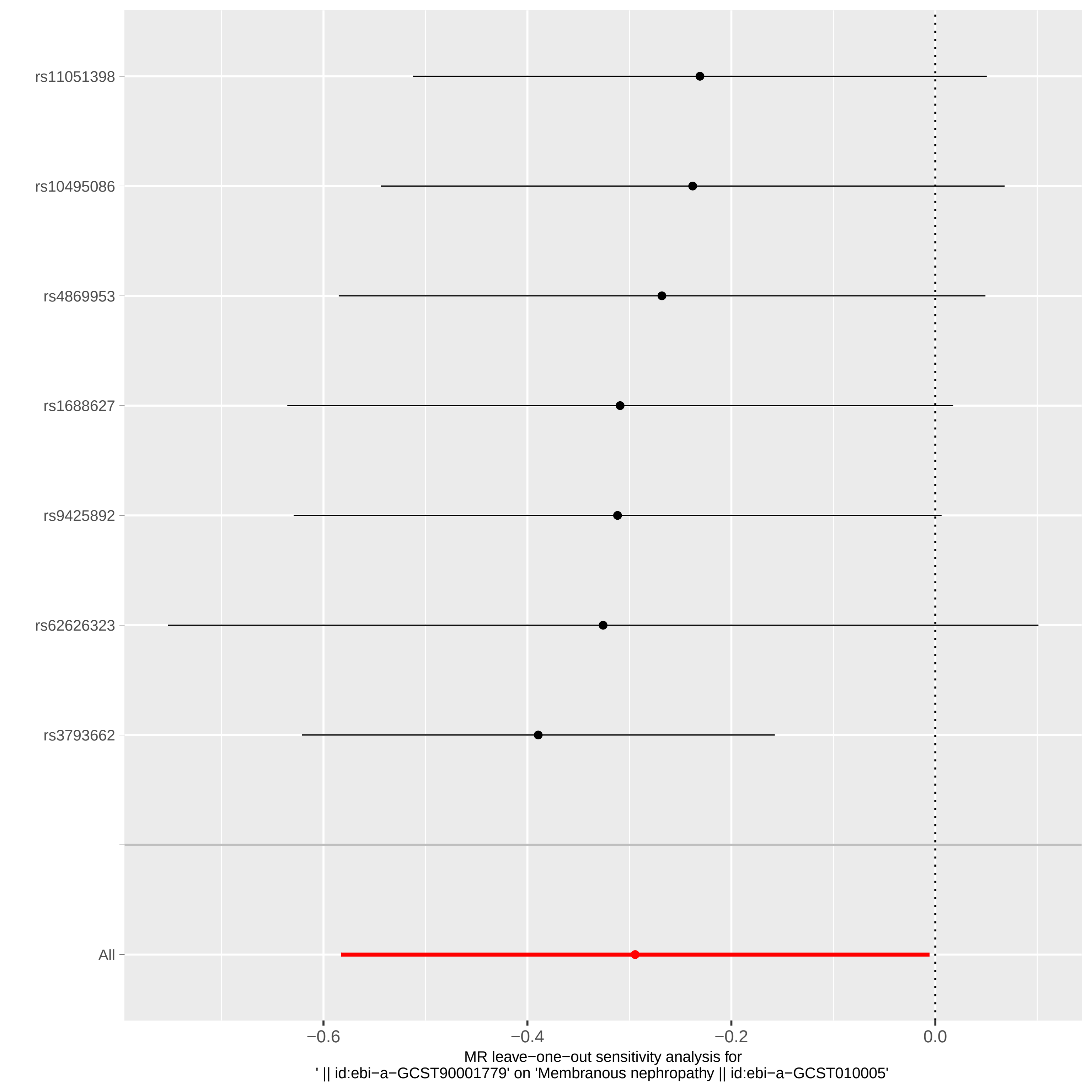
rs11051398
rs10495086
rs4869953
rs1688627
rs9425892
rs62626323
rs3793662
All
−0.6
−0.4
−0.2
0.0
MR leave−one−out sensitivity analysis for
' || id:ebi−a−GCST90001779' on 'Membranous nephropathy || id:ebi−a−GCST010005'

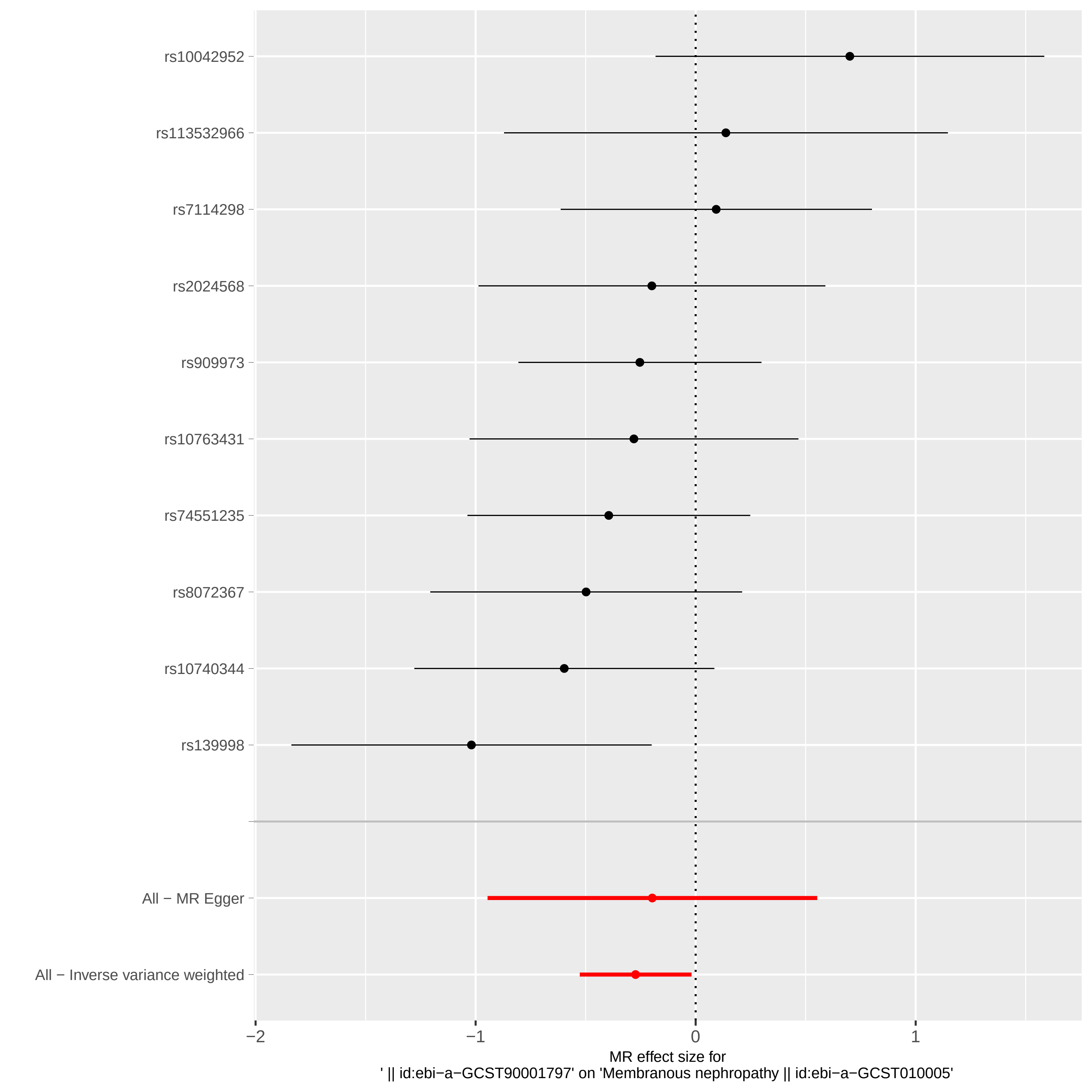
rs10042952
rs113532966
rs7114298
rs2024568
rs909973
rs10763431
rs74551235
rs8072367
rs10740344
rs139998
All − MR Egger
All − Inverse variance weighted
−2
−1
0
1
MR effect size for
' || id:ebi−a−GCST90001797' on 'Membranous nephropathy || id:ebi−a−GCST010005'

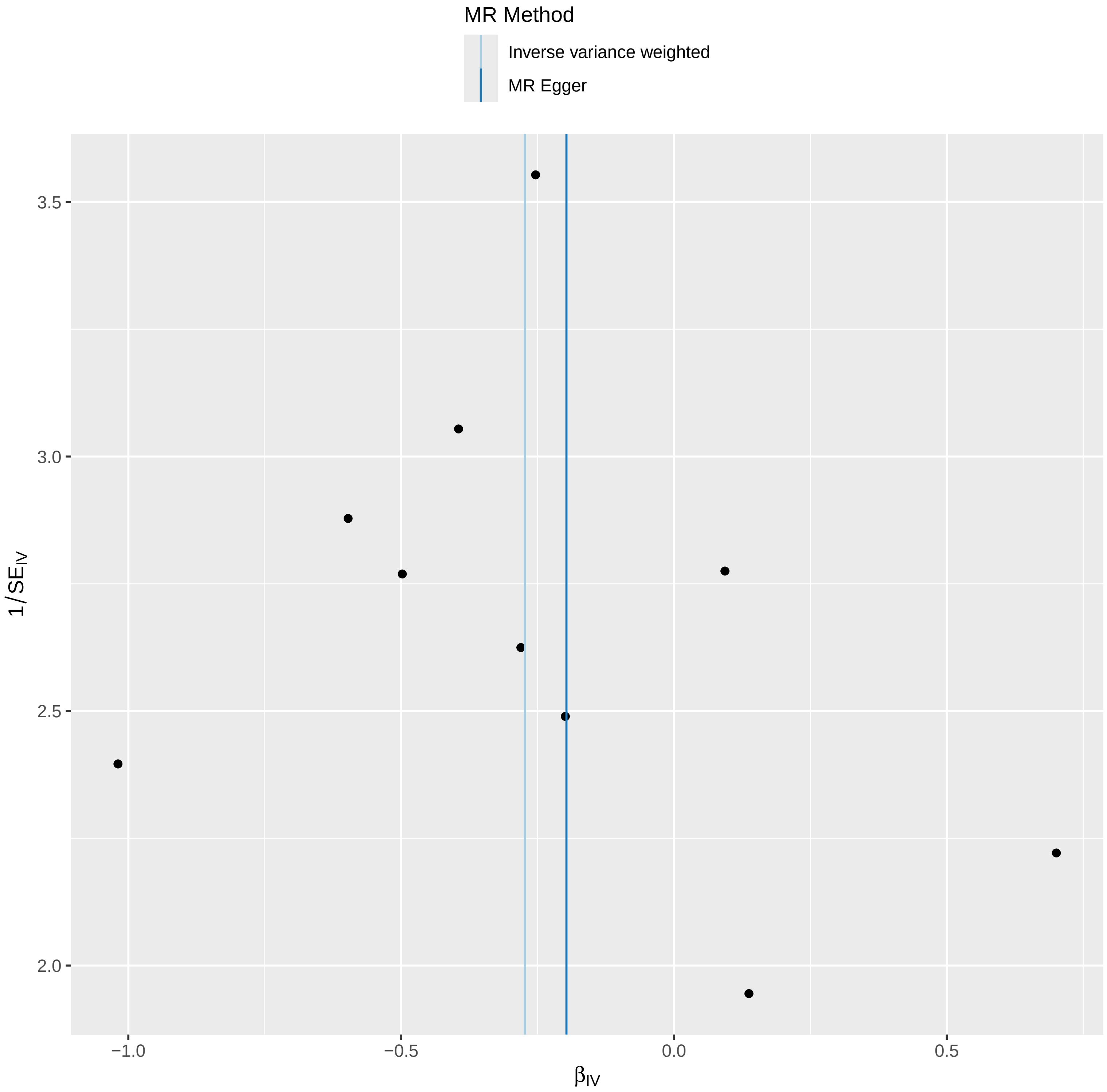

MR Method
Inverse variance weighted
MR Egger
3.5
3.0
2.5
2.0
1/SE$_{IV}$
−1.0
−0.5
0.0
0.5
β$_{IV}$

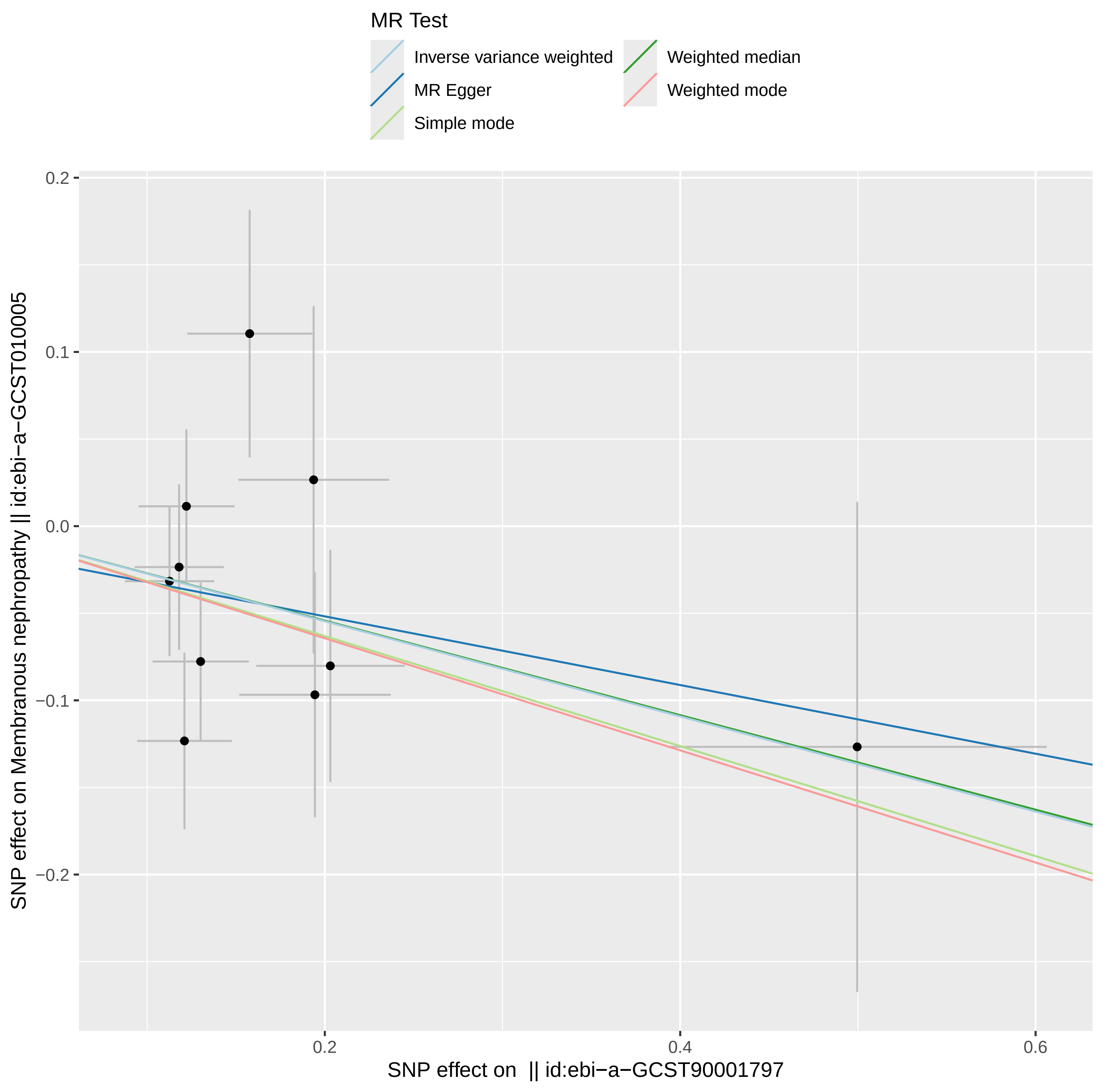

MR Test
Inverse variance weighted
MR Egger
Simple mode
Weighted median
Weighted mode
0.2
0.1
0.0
−0.1
−0.2
0.2
0.4
0.6
SNP effect on Membranous nephropathy || id:ebi−a−GCST010005
SNP effect on || id:ebi−a−GCST90001797

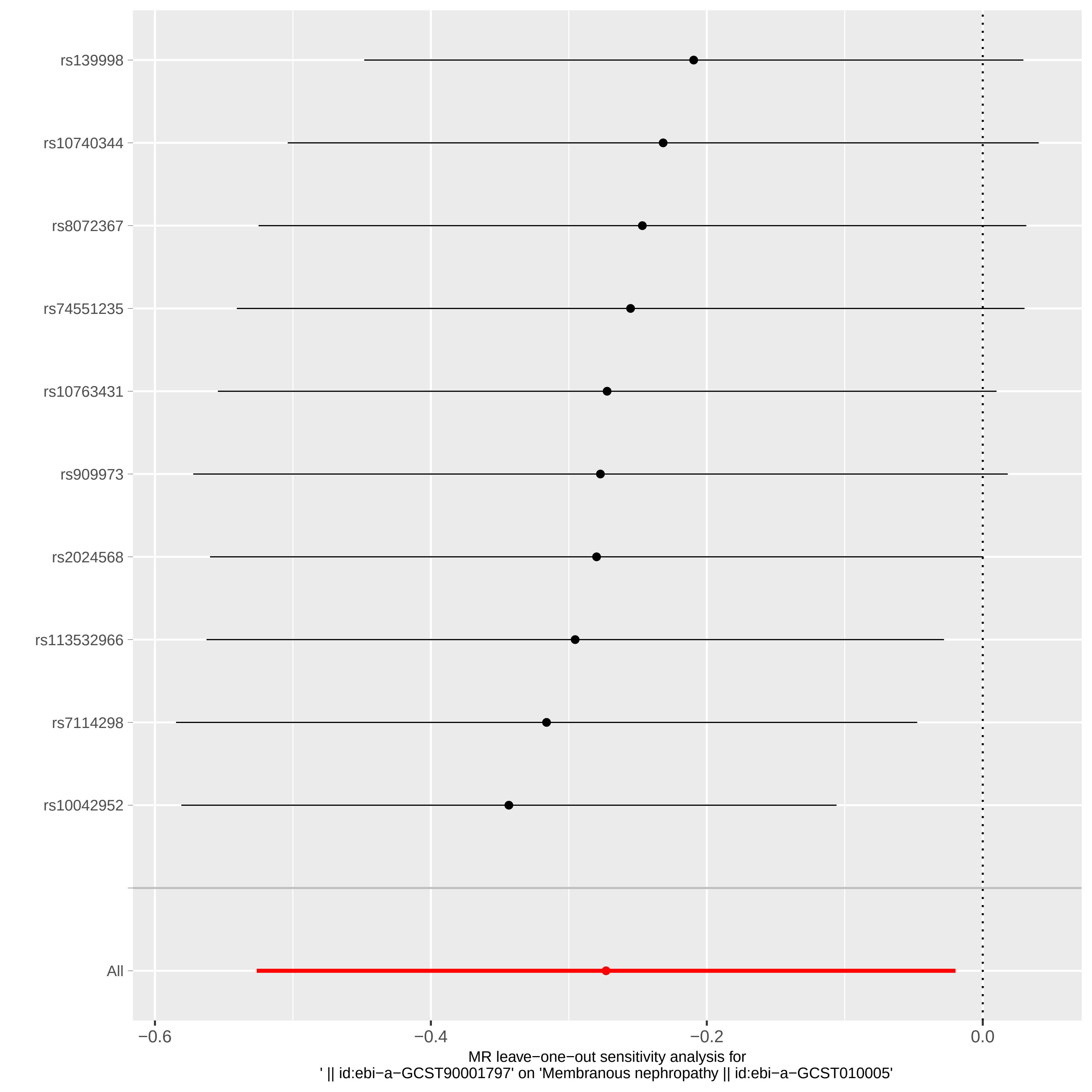
rs139998
rs10740344
rs8072367
rs74551235
rs10763431
rs909973
rs2024568
rs113532966
rs7114298
rs10042952
All
−0.6
−0.4
−0.2
0.0
MR leave−one−out sensitivity analysis for
' || id:ebi−a−GCST90001797' on 'Membranous nephropathy || id:ebi−a−GCST010005'

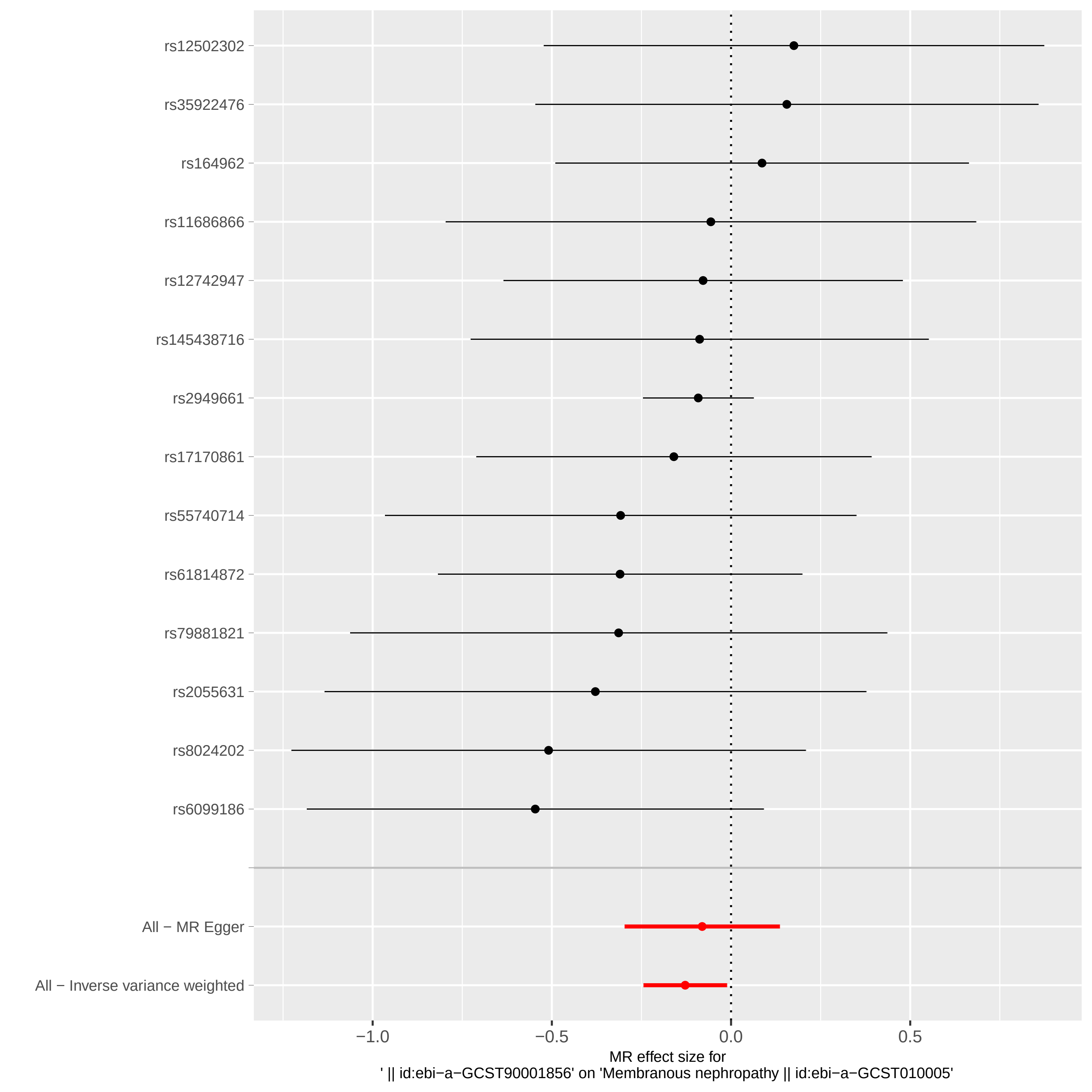
rs12502302
rs35922476
rs164962
rs11686866
rs12742947
rs145438716
rs2949661
rs17170861
rs55740714
rs61814872
rs79881821
rs2055631
rs8024202
rs6099186
All − MR Egger
All − Inverse variance weighted
−1.0
−0.5
0.0
0.5
MR effect size for
' || id:ebi−a−GCST90001856' on 'Membranous nephropathy || id:ebi−a−GCST010005'

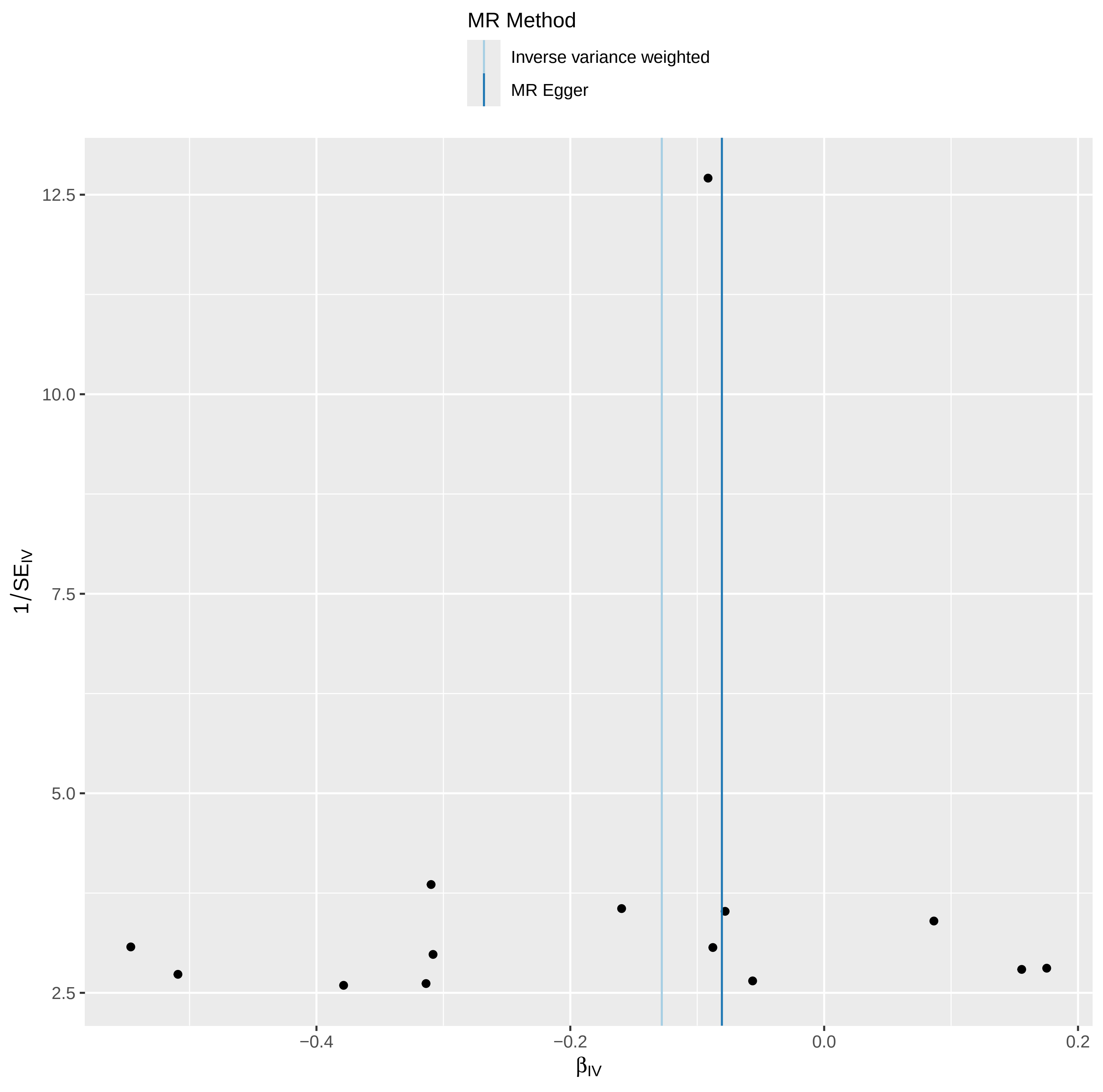

MR Method
Inverse variance weighted
MR Egger
12.5
10.0
7.5
5.0
2.5
$1/SE_{IV}$
−0.4
−0.2
0.0
0.2
β_{IV}

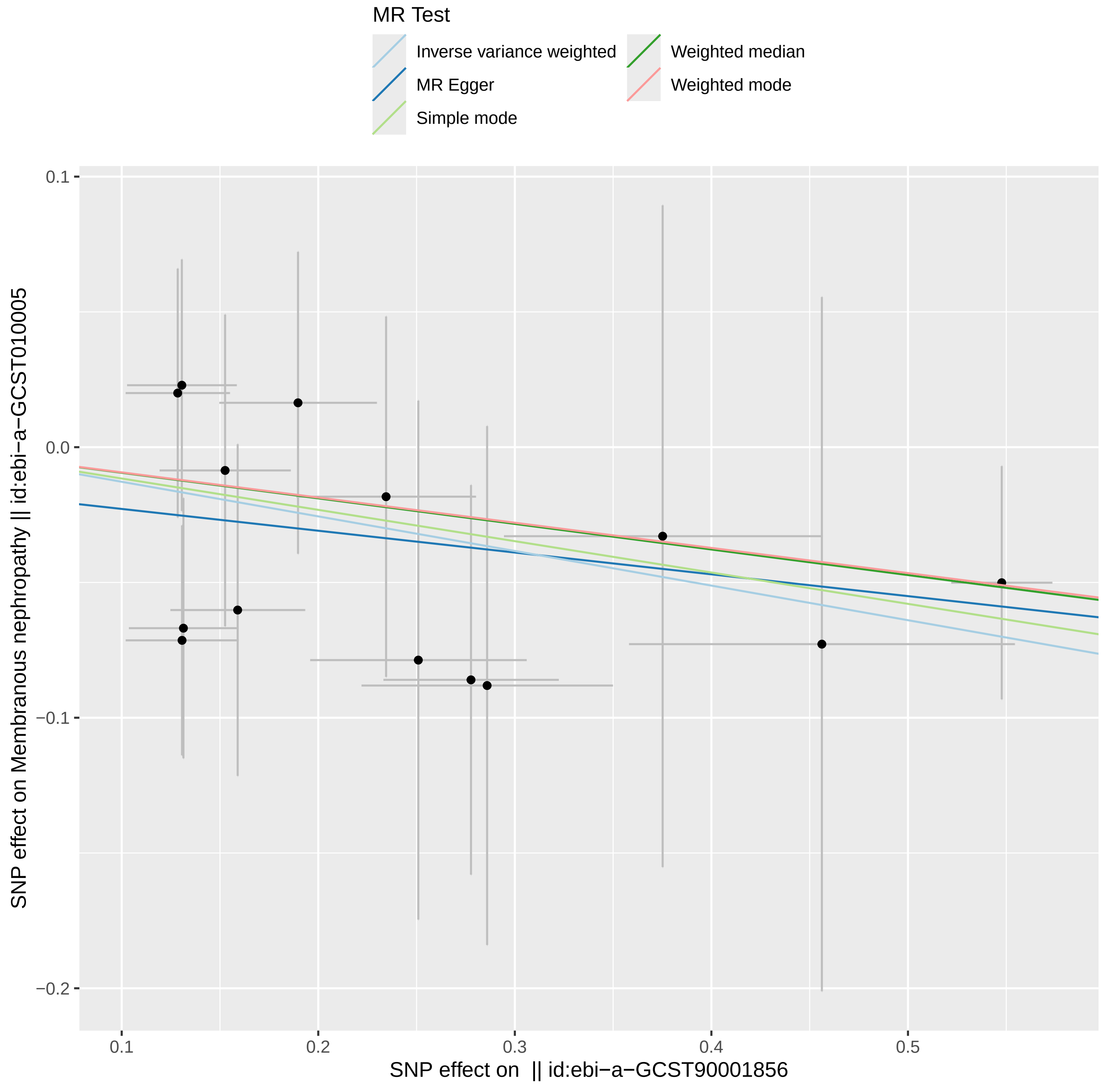
MR Test
Inverse variance weighted
MR Egger
Simple mode
Weighted median
Weighted mode
0.1
0.0
−0.1
−0.2
0.1
0.2
0.3
0.4
0.5
SNP effect on Membranous nephropathy || id:ebi−a−GCST010005
SNP effect on || id:ebi−a−GCST90001856

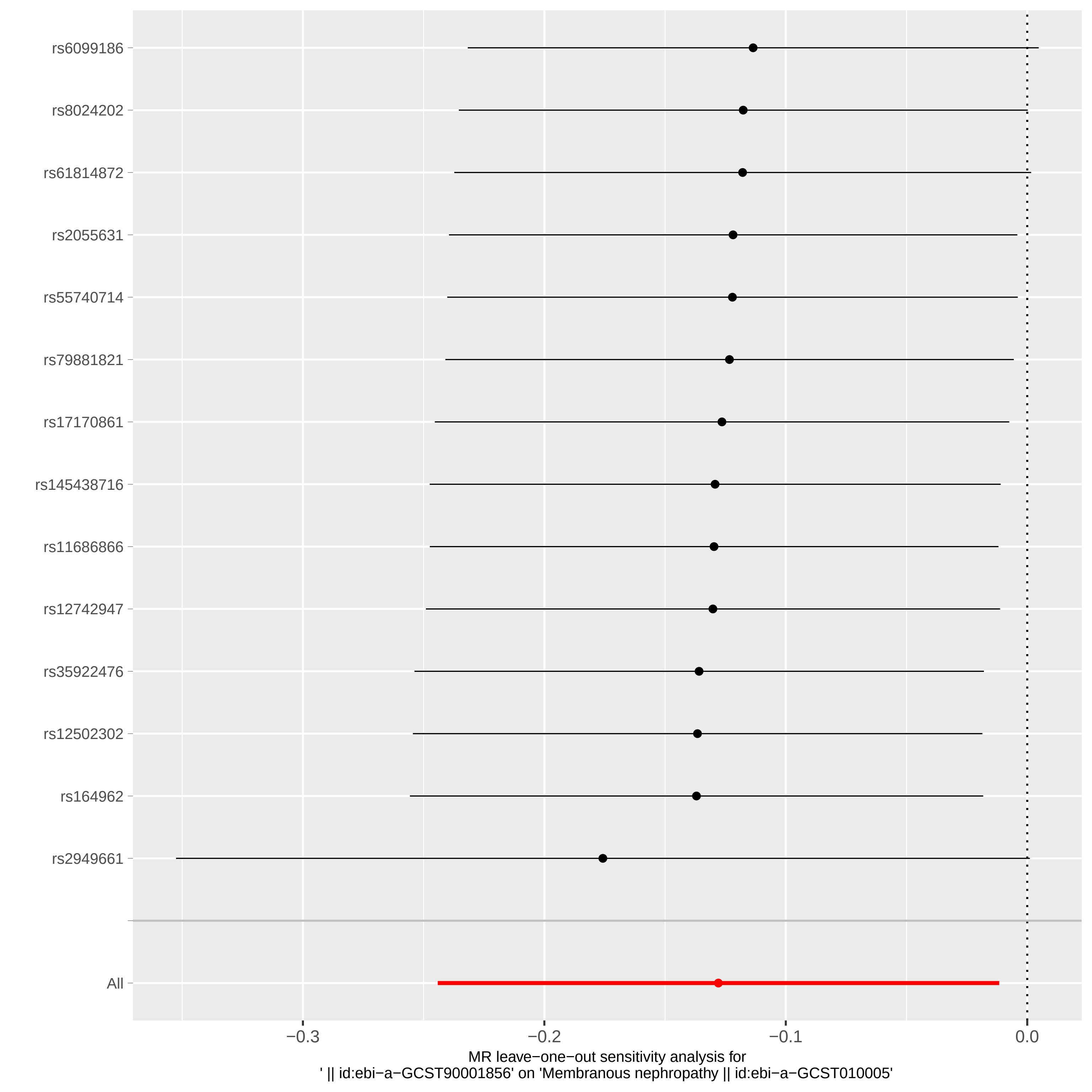
rs6099186
rs8024202
rs61814872
rs2055631
rs55740714
rs79881821
rs17170861
rs145438716
rs11686866
rs12742947
rs35922476
rs12502302
rs164962
rs2949661
All
−0.3
−0.2
−0.1
0.0
MR leave−one−out sensitivity analysis for
' || id:ebi−a−GCST90001856' on 'Membranous nephropathy || id:ebi−a−GCST010005'

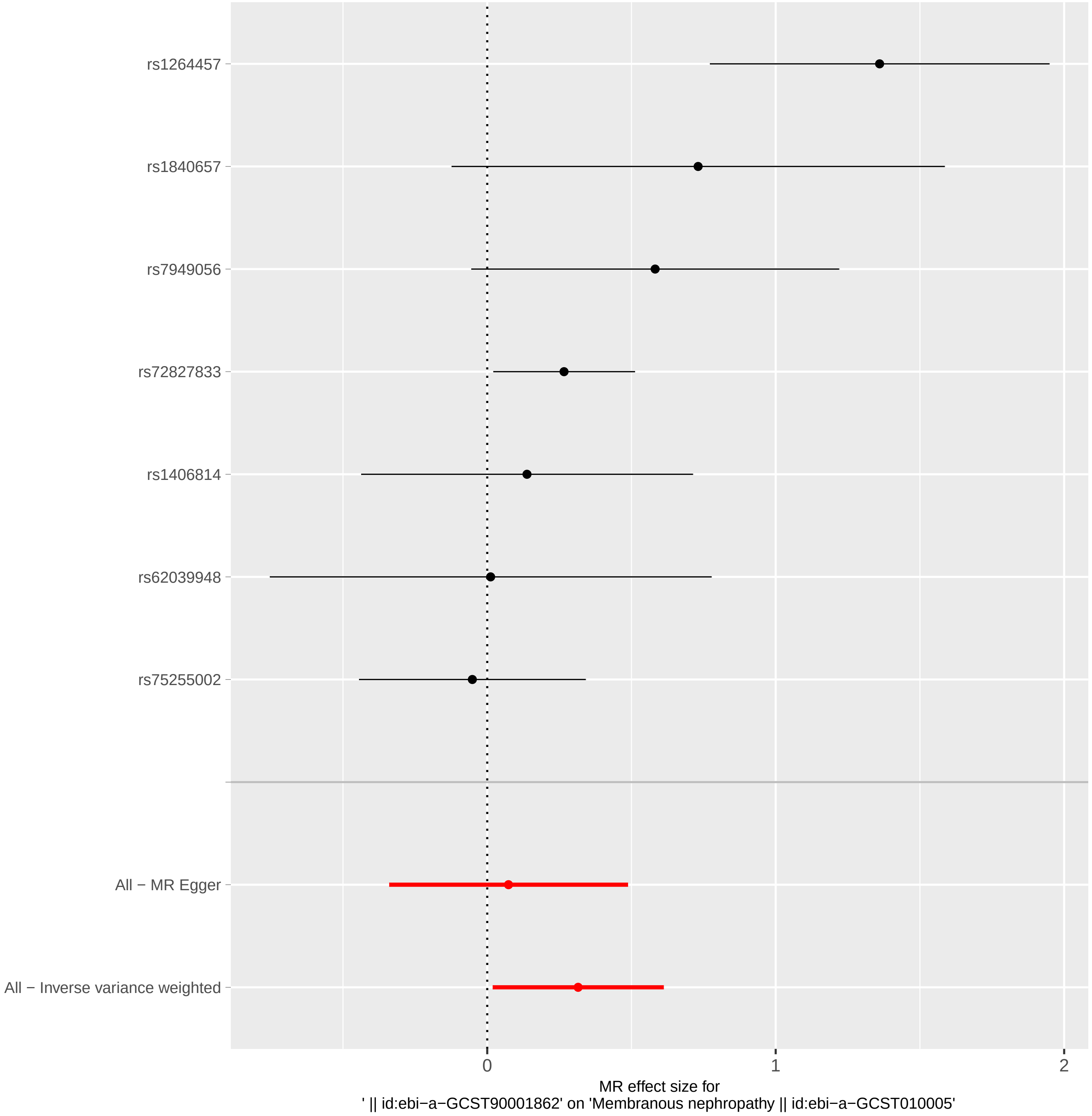
rs1264457
rs1840657
rs7949056
rs72827833
rs1406814
rs62039948
rs75255002
All − MR Egger
All − Inverse variance weighted
0
1
2
MR effect size for
' || id:ebi−a−GCST90001862' on 'Membranous nephropathy || id:ebi−a−GCST010005'

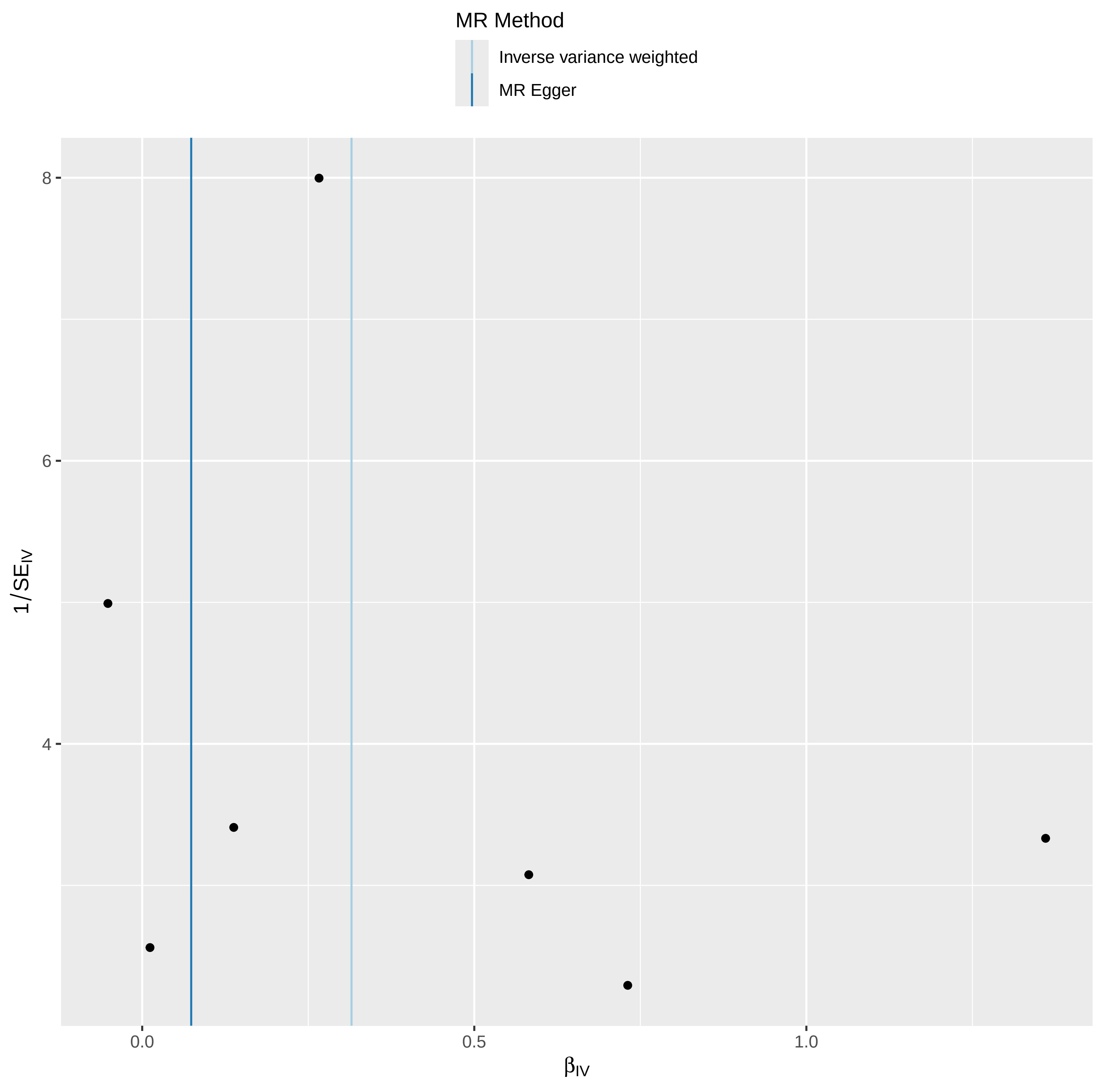
MR Method
Inverse variance weighted
MR Egger
8
6
4
1/SE_IV
0.0
0.5
1.0
β_IV

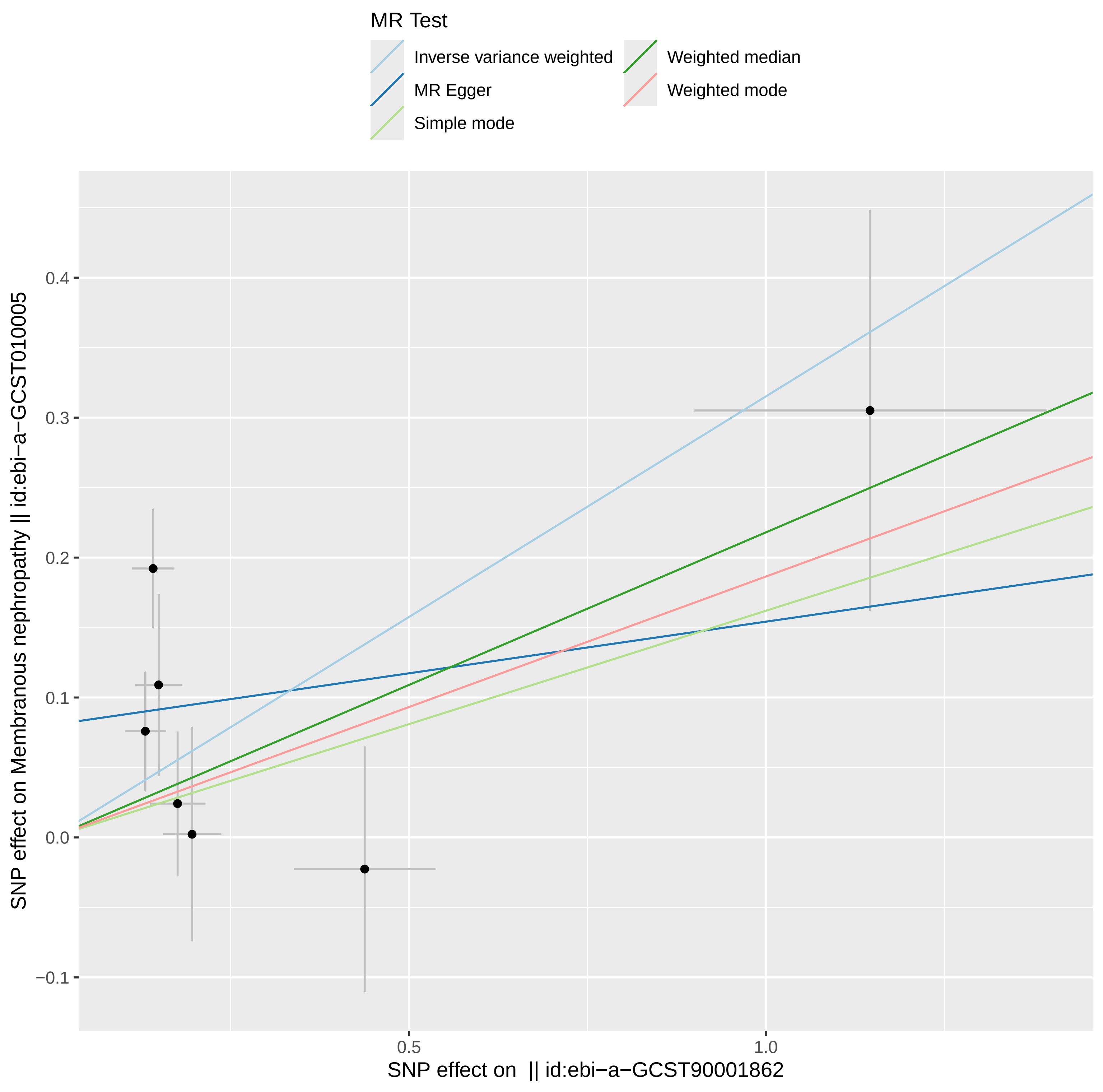
MR Test
Inverse variance weighted
MR Egger
Simple mode
Weighted median
Weighted mode
SNP effect on Membranous nephropathy || id:ebi−a−GCST010005
0.4
0.3
0.2
0.1
0.0
−0.1
0.5
1.0
SNP effect on || id:ebi−a−GCST90001862

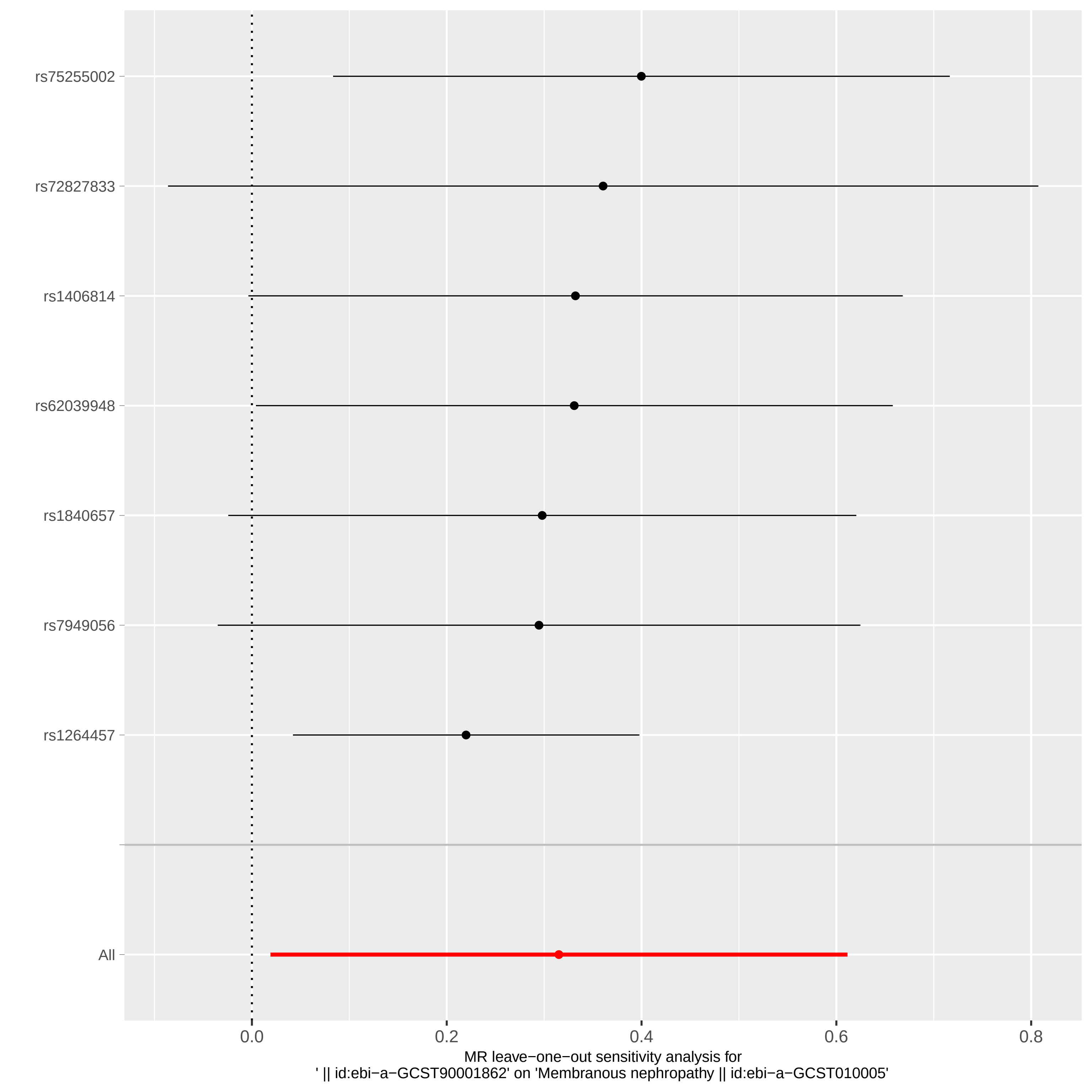

rs75255002
rs72827833
rs1406814
rs62039948
rs1840657
rs7949056
rs1264457
All
0.0
0.2
0.4
0.6
0.8
MR leave−one−out sensitivity analysis for
' || id:ebi−a−GCST90001862' on 'Membranous nephropathy || id:ebi−a−GCST010005'

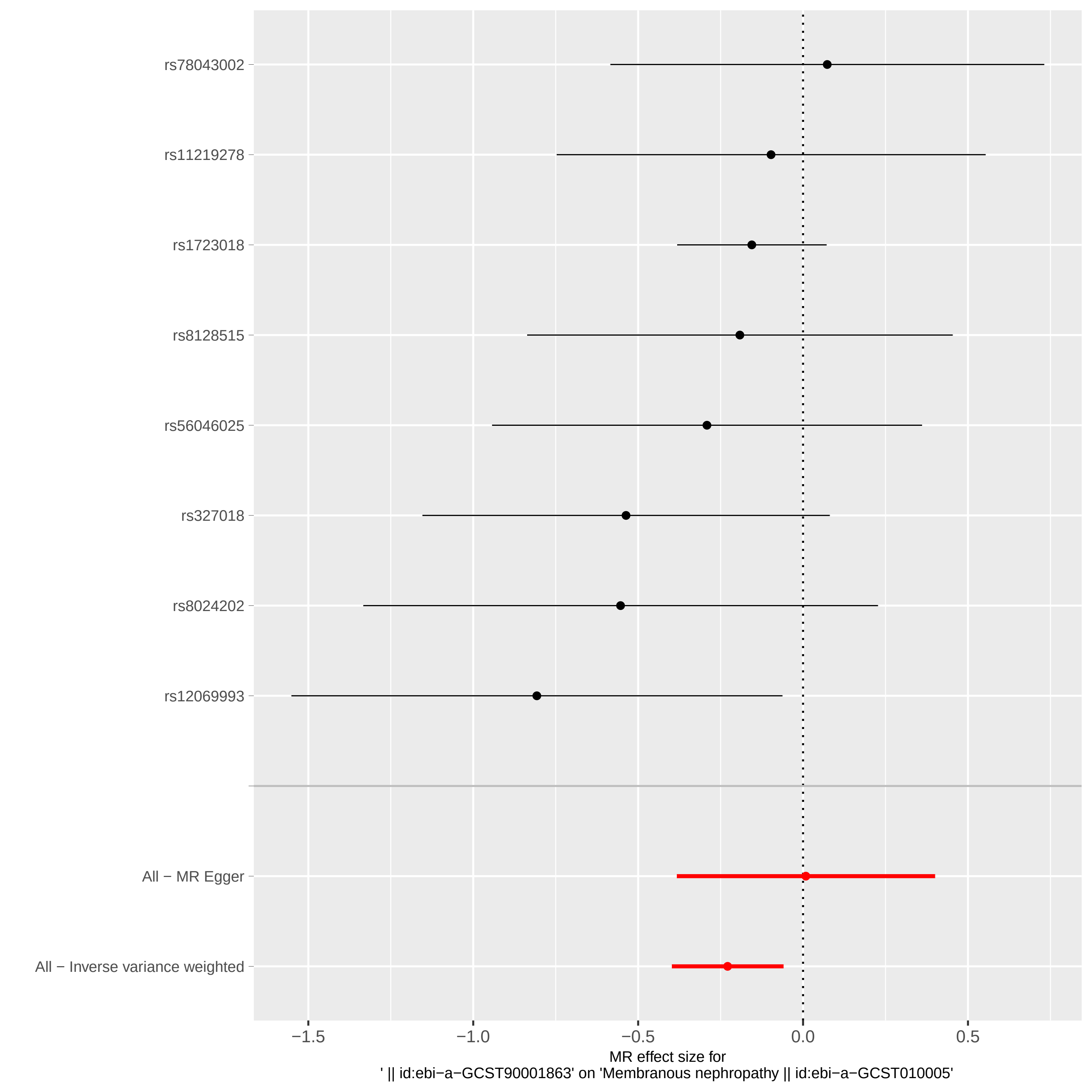

rs78043002
rs11219278
rs1723018
rs8128515
rs56046025
rs327018
rs8024202
rs12069993
All − MR Egger
All − Inverse variance weighted
−1.5
−1.0
−0.5
0.0
0.5
MR effect size for
' || id:ebi−a−GCST90001863' on 'Membranous nephropathy || id:ebi−a−GCST010005'

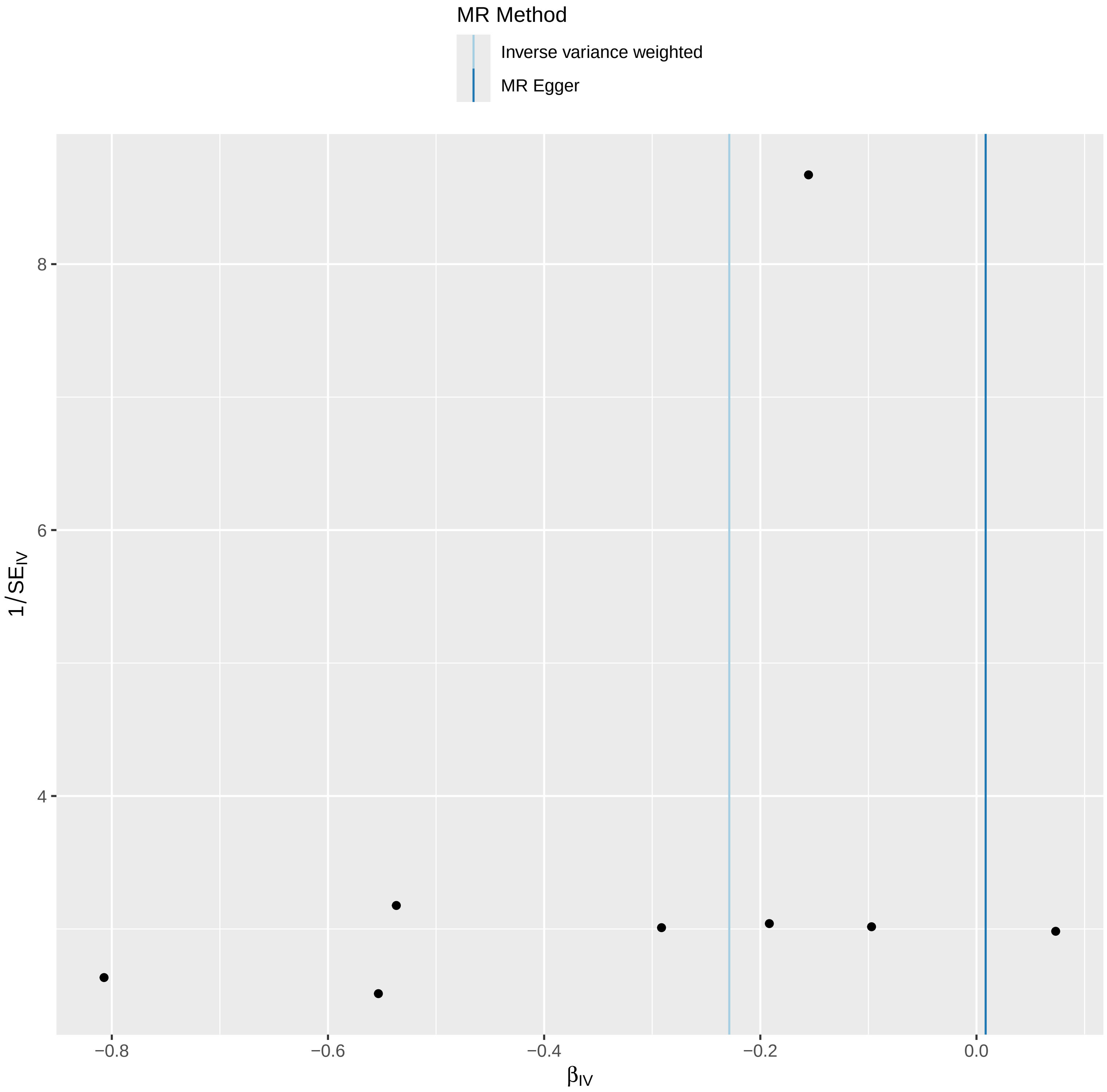
MR Method
Inverse variance weighted
MR Egger
1/SE$_{IV}$
β$_{IV}$
8
6
4
−0.8
−0.6
−0.4
−0.2
0.0

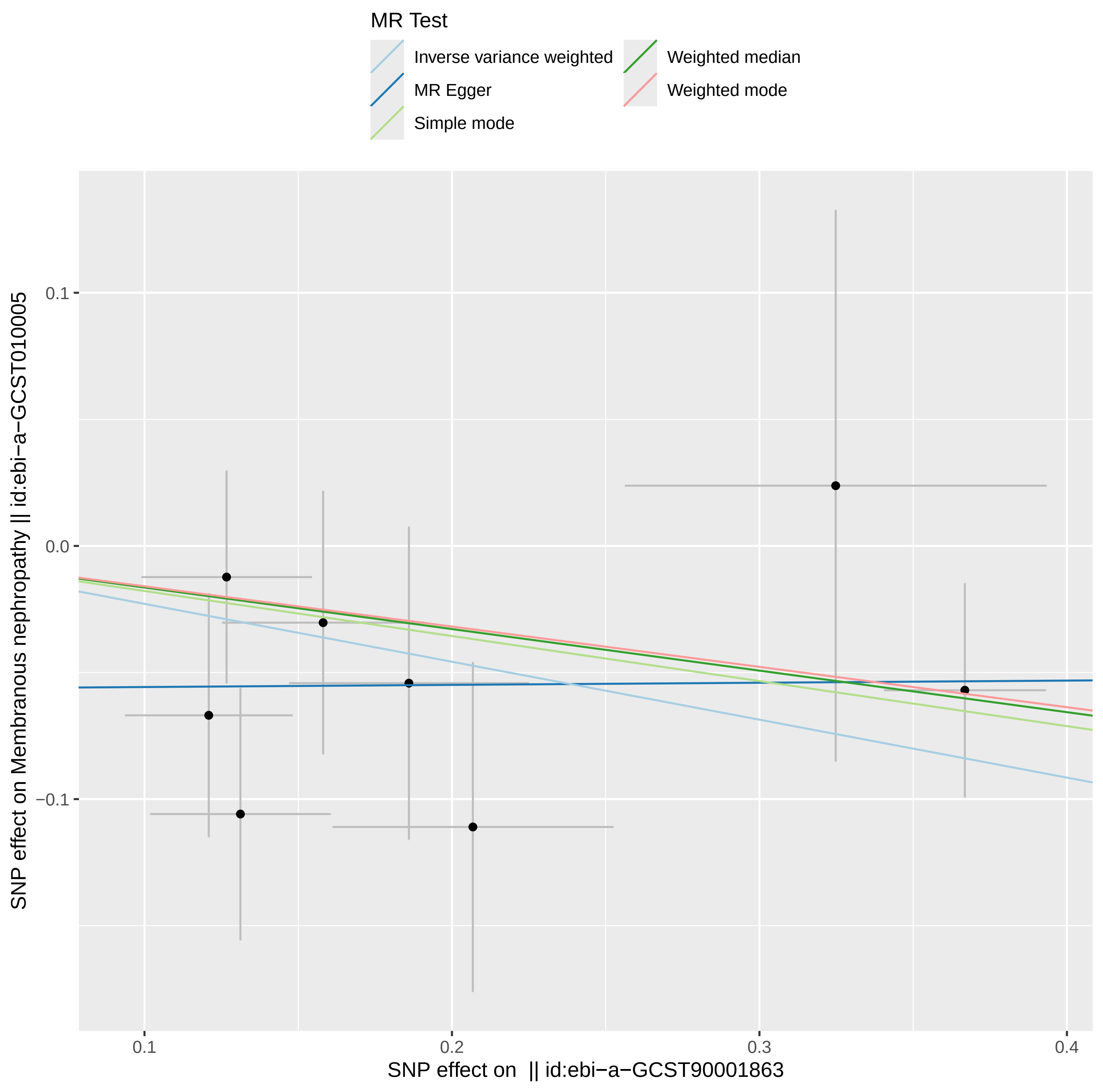

MR Test
Inverse variance weighted
MR Egger
Simple mode
Weighted median
Weighted mode
SNP effect on Membranous nephropathy || id:ebi−a−GCST010005
0.1
0.0
−0.1
0.1
0.2
0.3
0.4
SNP effect on || id:ebi−a−GCST90001863

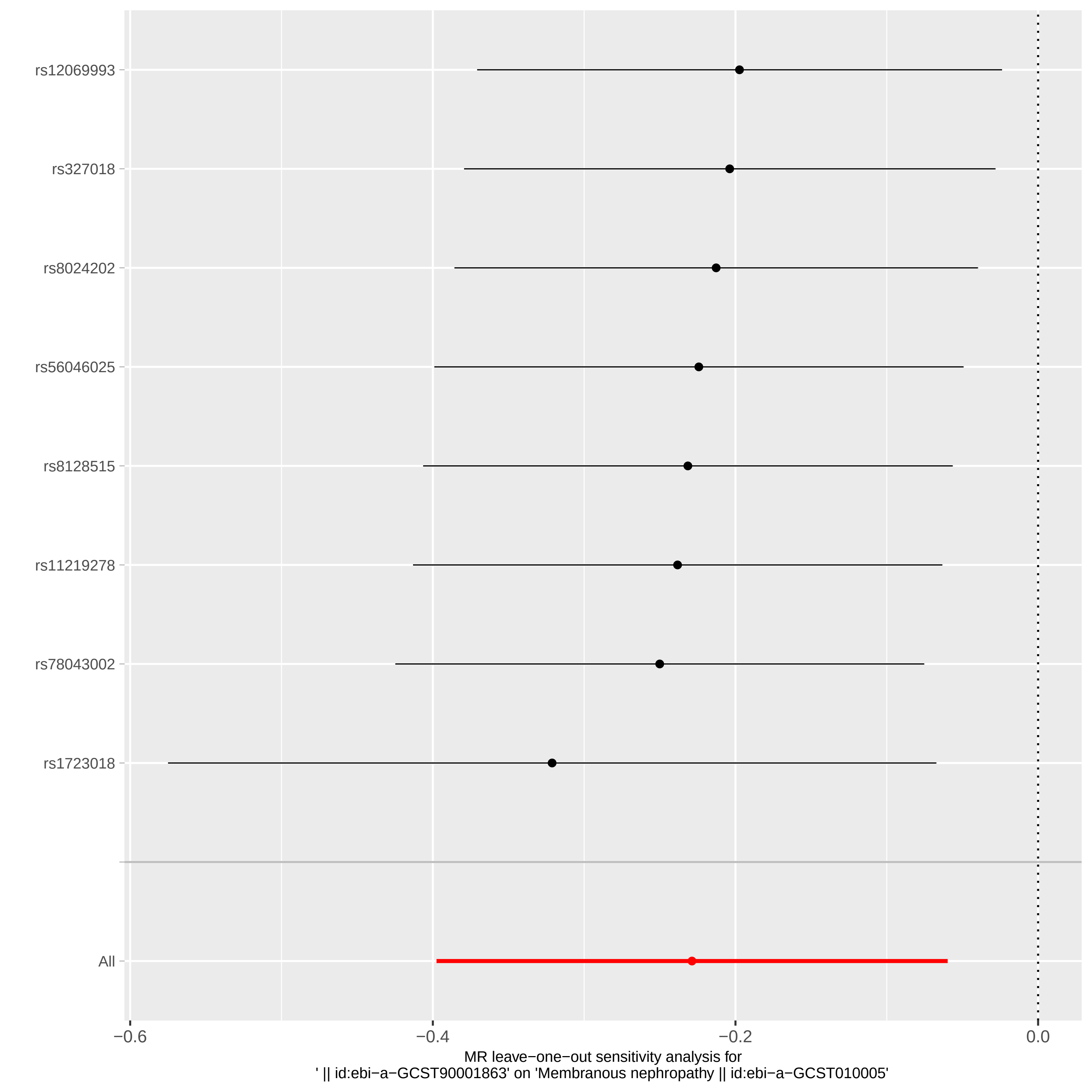

rs12069993
rs327018
rs8024202
rs56046025
rs8128515
rs11219278
rs78043002
rs1723018
All
−0.6
−0.4
−0.2
0.0
MR leave−one−out sensitivity analysis for
' || id:ebi−a−GCST90001863' on 'Membranous nephropathy || id:ebi−a−GCST010005'

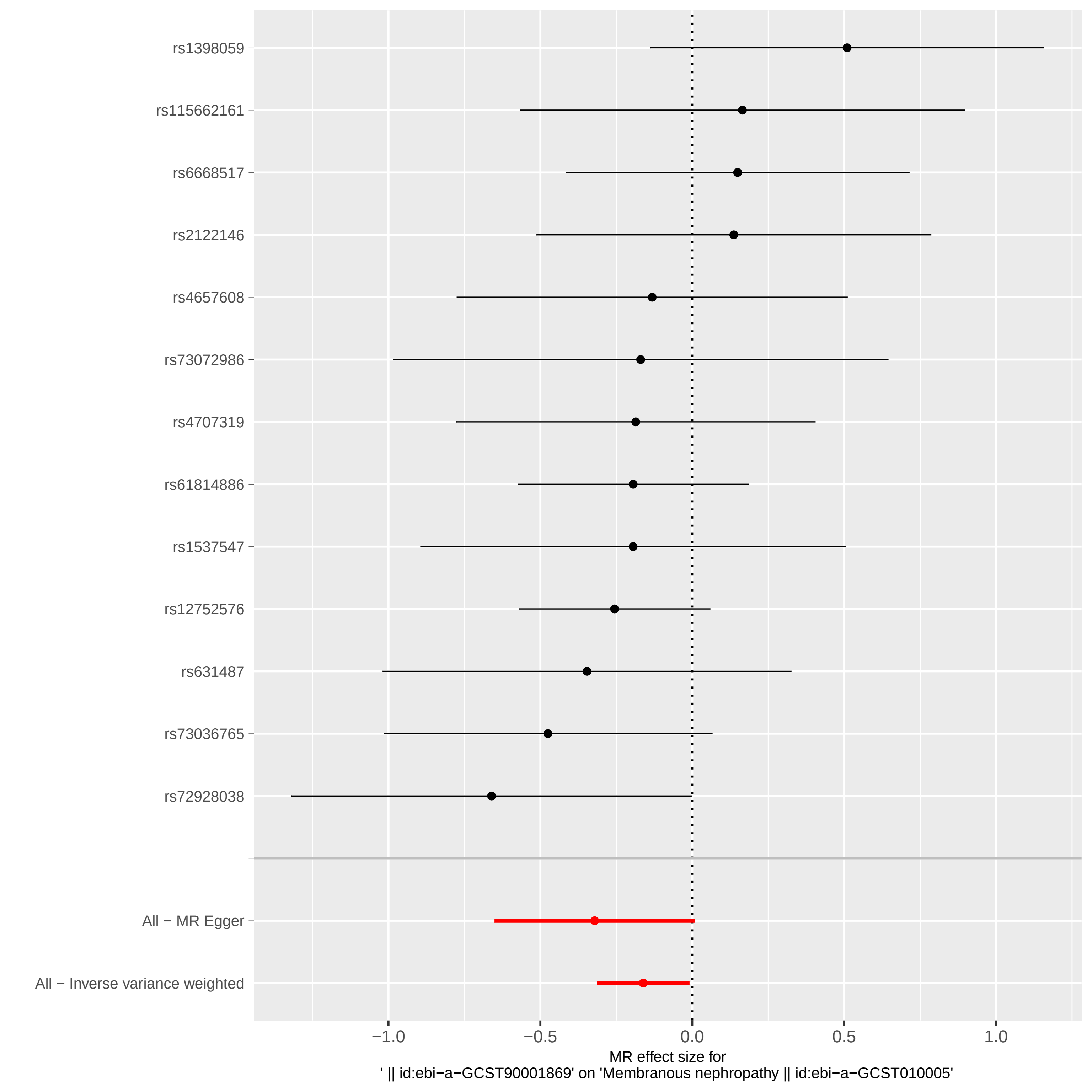
rs1398059
rs115662161
rs6668517
rs2122146
rs4657608
rs73072986
rs4707319
rs61814886
rs1537547
rs12752576
rs631487
rs73036765
rs72928038
All − MR Egger
All − Inverse variance weighted
−1.0
−0.5
0.0
0.5
1.0
MR effect size for
' || id:ebi−a−GCST90001869' on 'Membranous nephropathy || id:ebi−a−GCST010005'

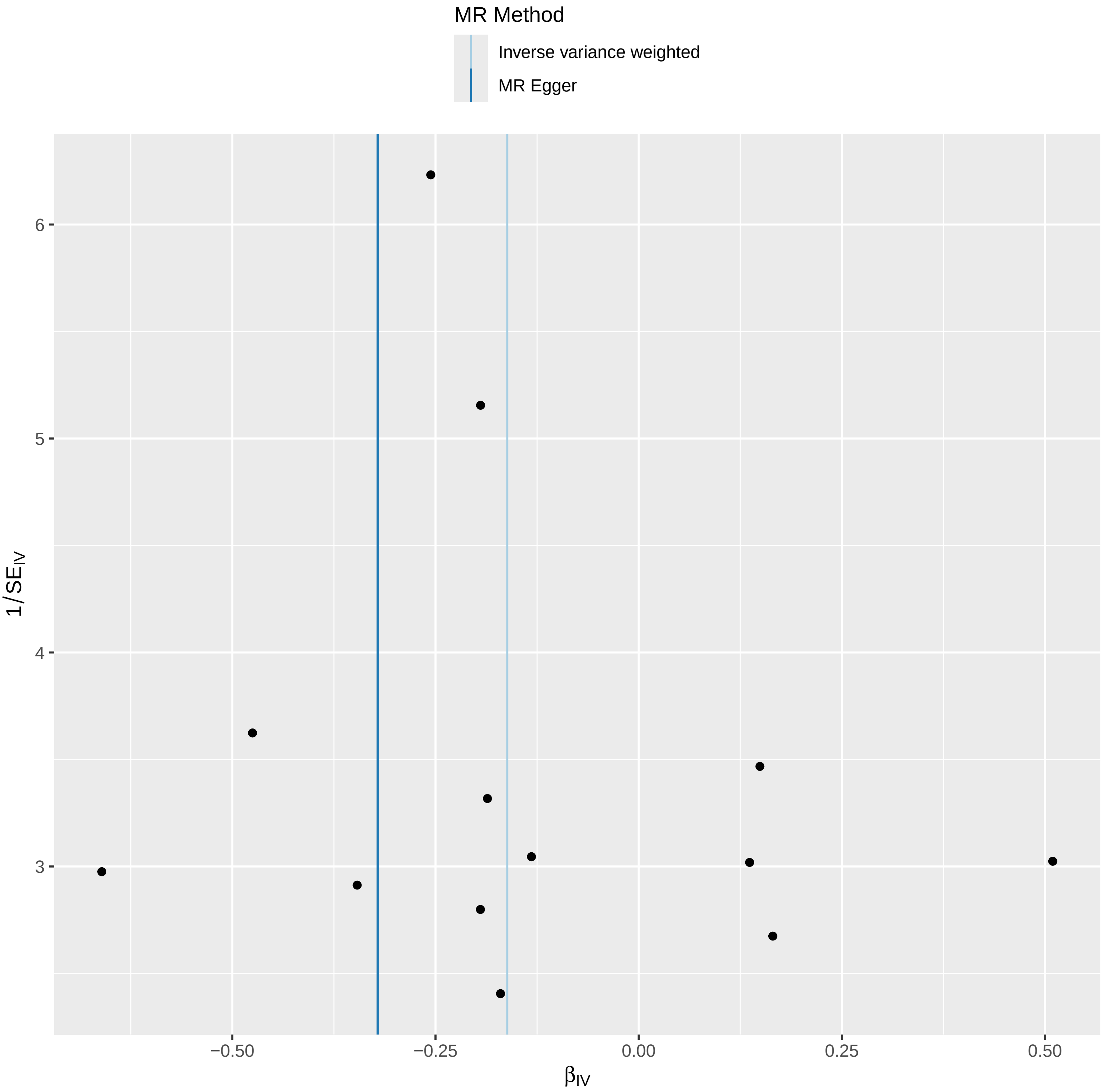
MR Method
Inverse variance weighted
MR Egger
6
5
4
3
1/SE_IV
−0.50
−0.25
0.00
0.25
0.50
β_IV

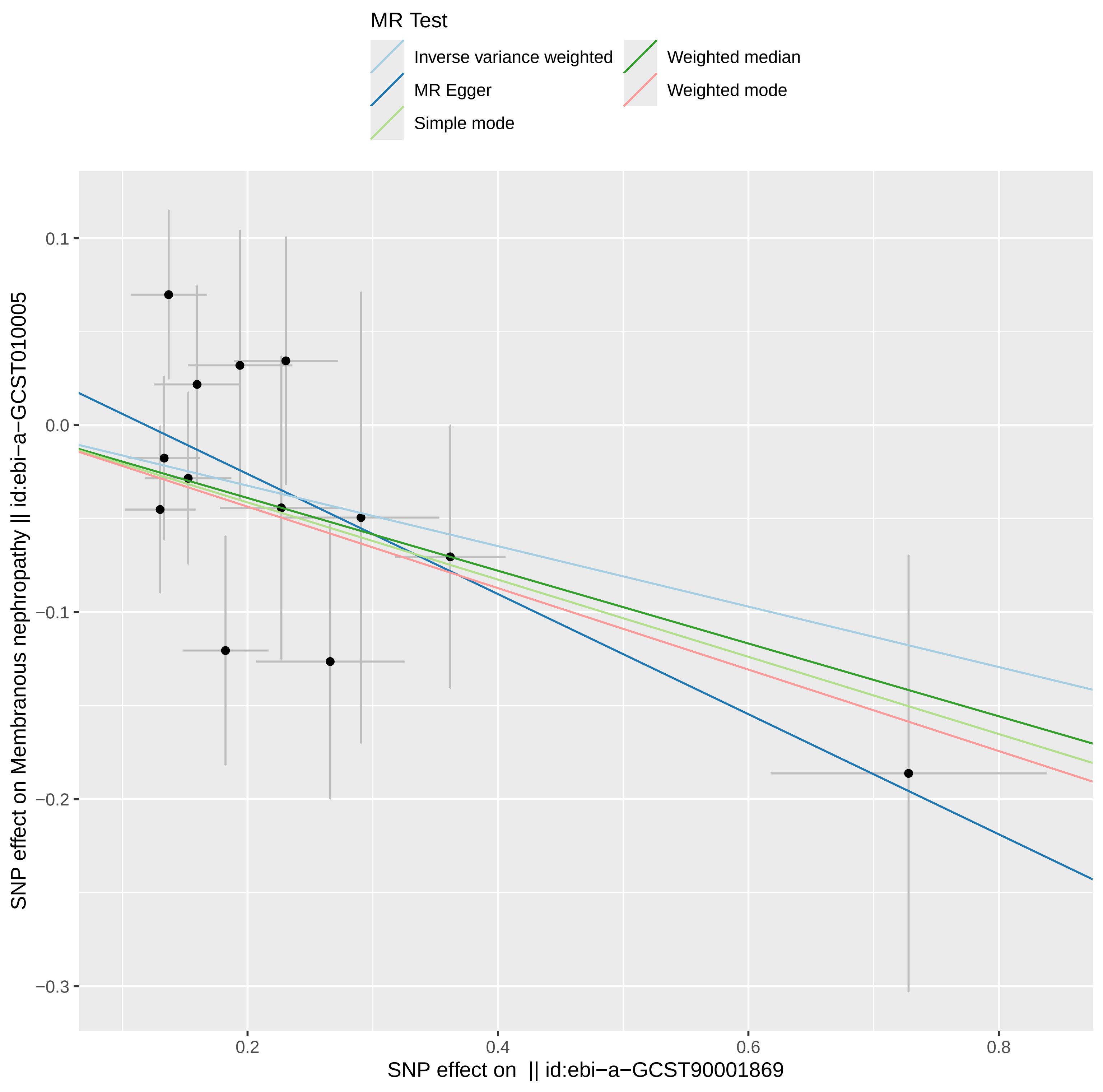

MR Test
Inverse variance weighted
MR Egger
Simple mode
Weighted median
Weighted mode
SNP effect on Membranous nephropathy || id:ebi−a−GCST010005
0.1
0.0
−0.1
−0.2
−0.3
SNP effect on || id:ebi−a−GCST90001869
0.2
0.4
0.6
0.8

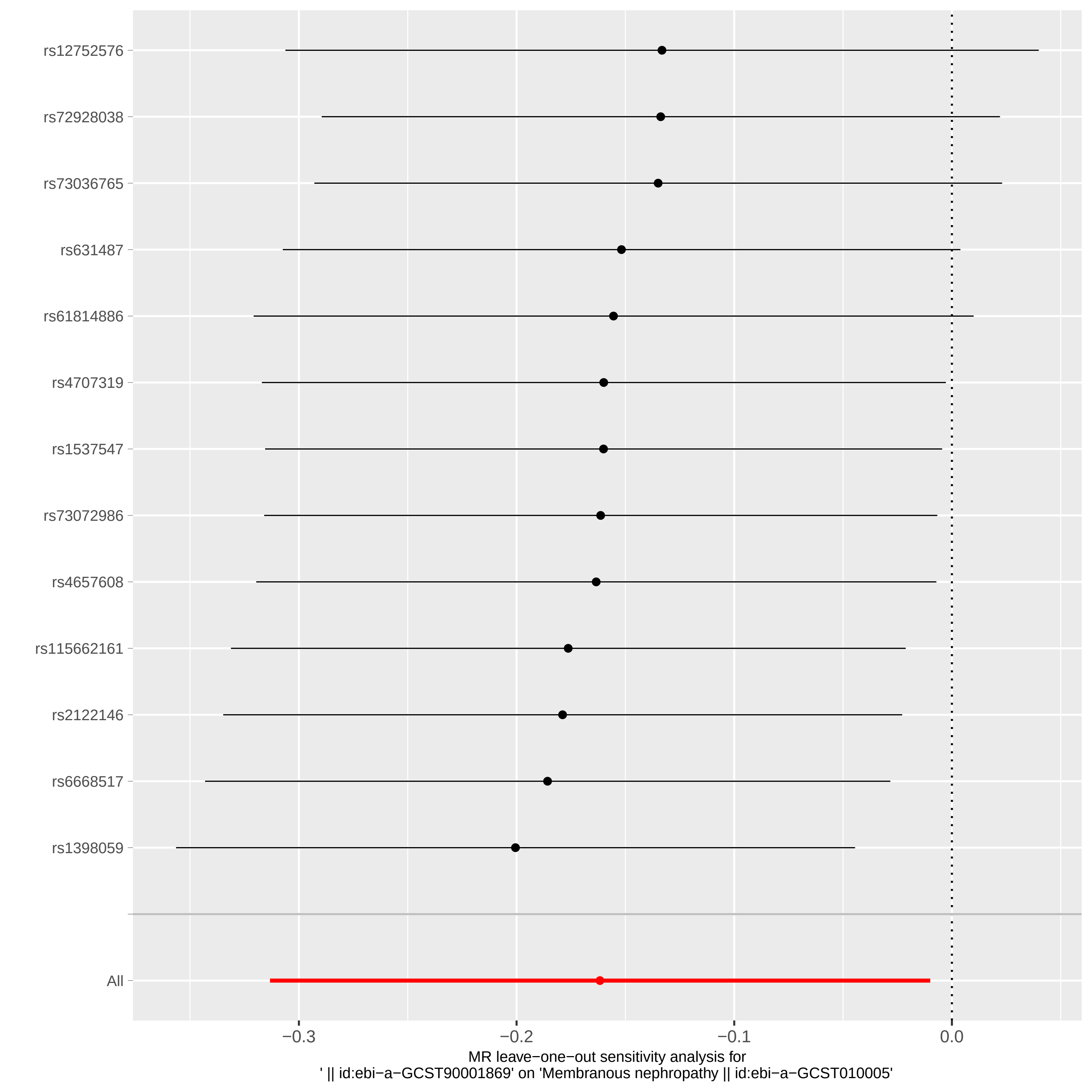

rs12752576
rs72928038
rs73036765
rs631487
rs61814886
rs4707319
rs1537547
rs73072986
rs4657608
rs115662161
rs2122146
rs6668517
rs1398059
All
−0.3
−0.2
−0.1
0.0
MR leave−one−out sensitivity analysis for
' || id:ebi−a−GCST90001869' on 'Membranous nephropathy || id:ebi−a−GCST010005'

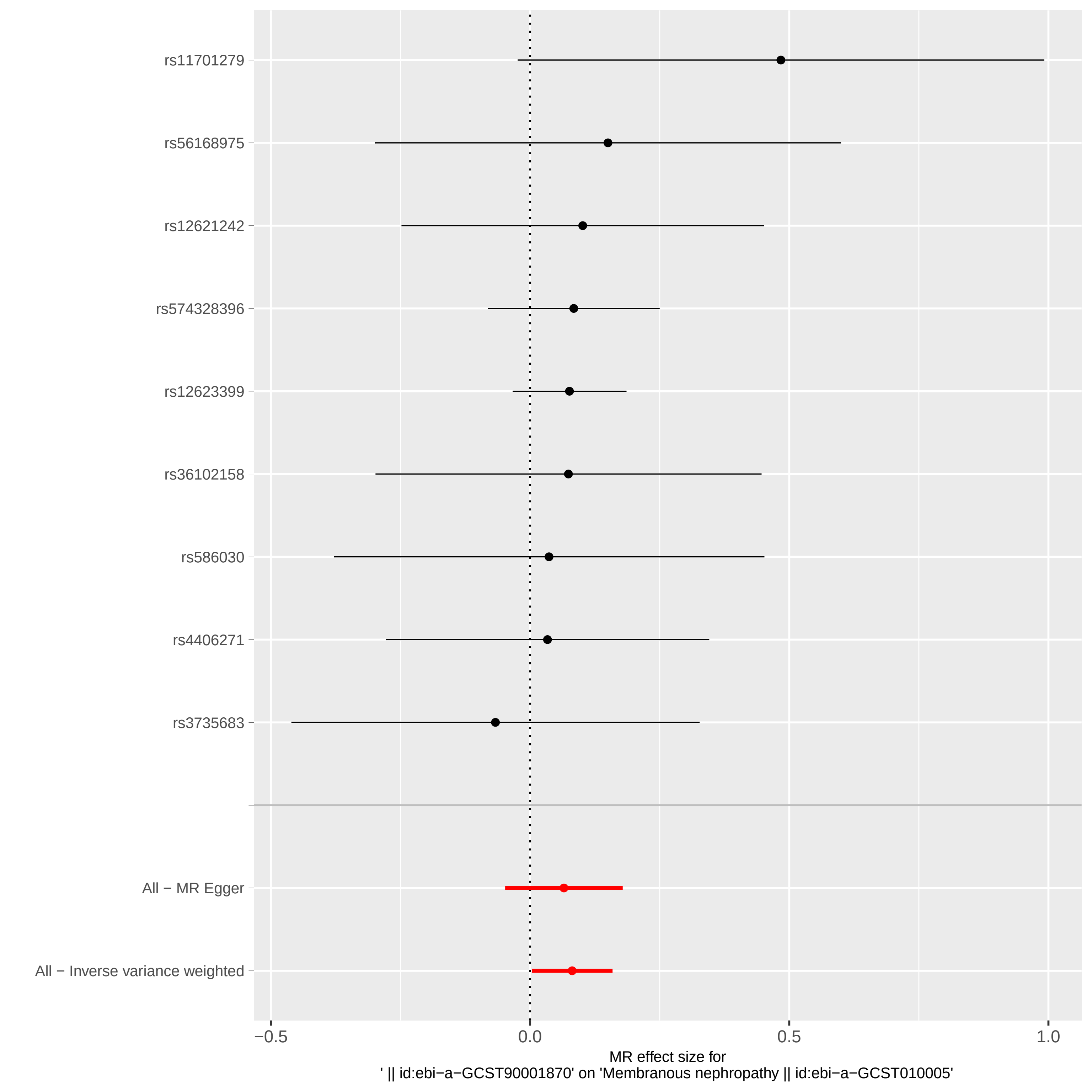
rs11701279
rs56168975
rs12621242
rs574328396
rs12623399
rs36102158
rs586030
rs4406271
rs3735683
All − MR Egger
All − Inverse variance weighted
−0.5
0.0
0.5
1.0
MR effect size for
' || id:ebi−a−GCST90001870' on 'Membranous nephropathy || id:ebi−a−GCST010005'

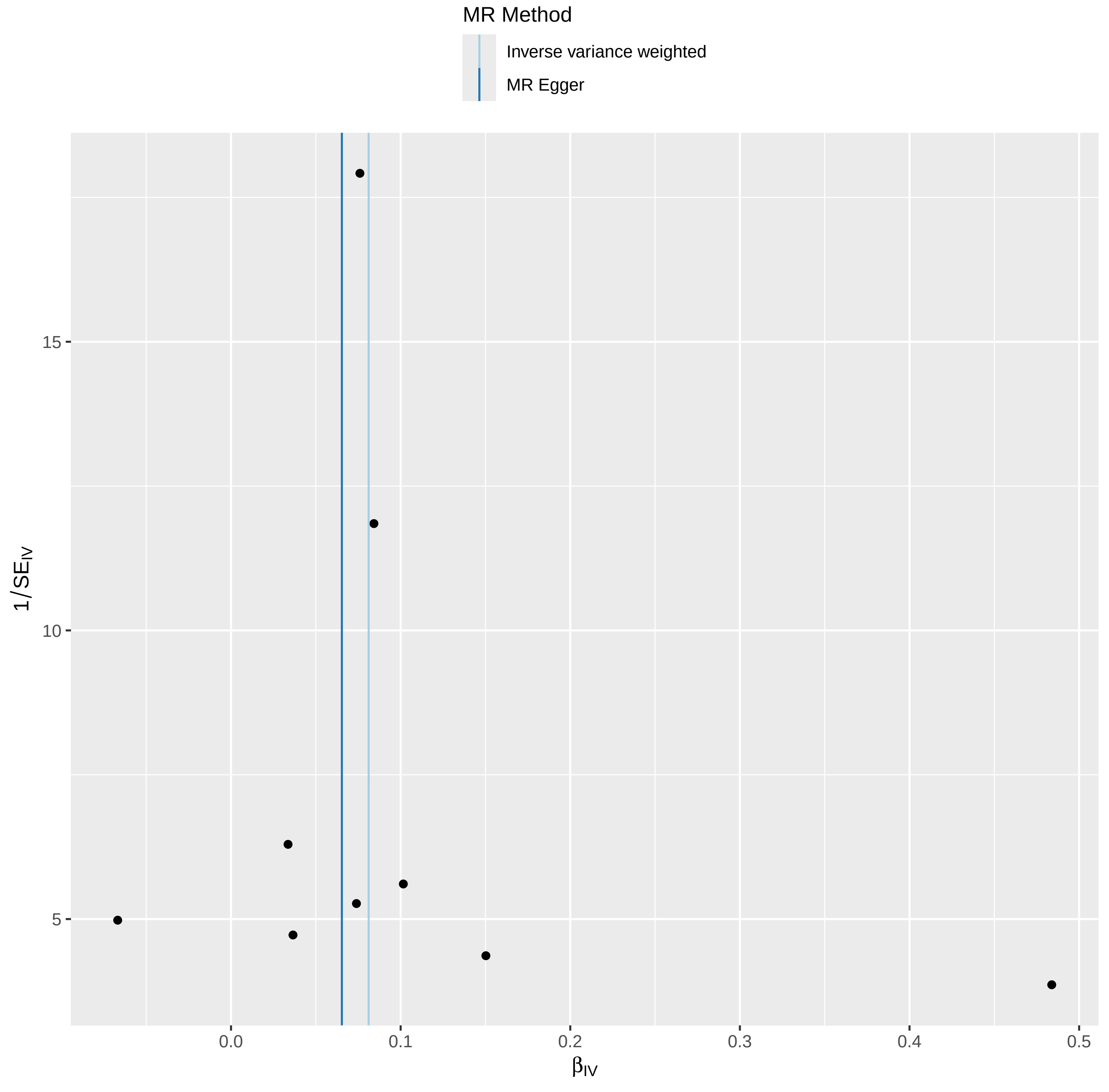
MR Method
Inverse variance weighted
MR Egger
15
10
5
1/SE_IV
0.0
0.1
0.2
0.3
0.4
0.5
β_IV

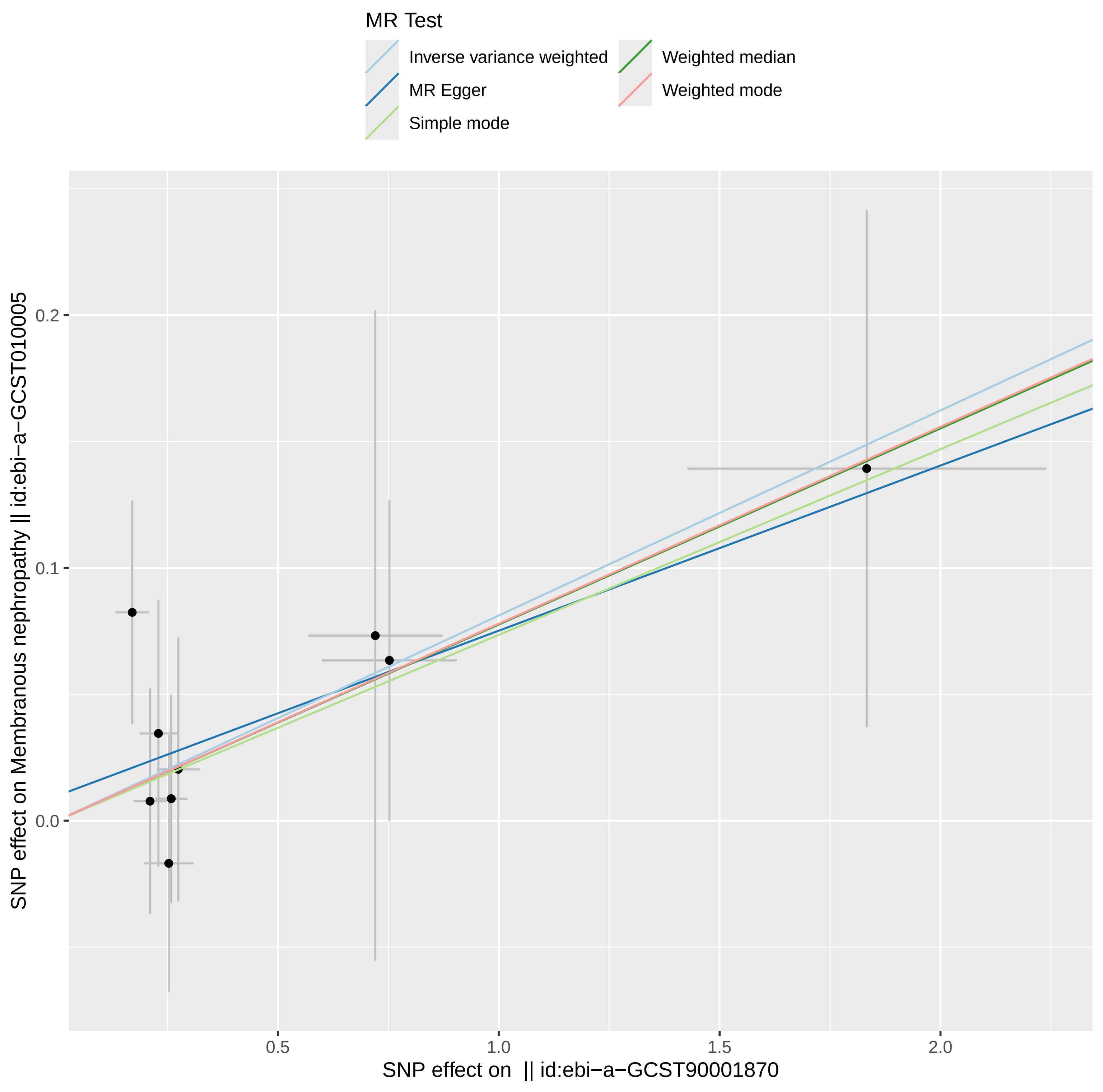
MR Test
Inverse variance weighted
MR Egger
Simple mode
Weighted median
Weighted mode
0.2
0.1
0.0
0.5
1.0
1.5
2.0
SNP effect on Membranous nephropathy || id:ebi−a−GCST010005
SNP effect on || id:ebi−a−GCST90001870

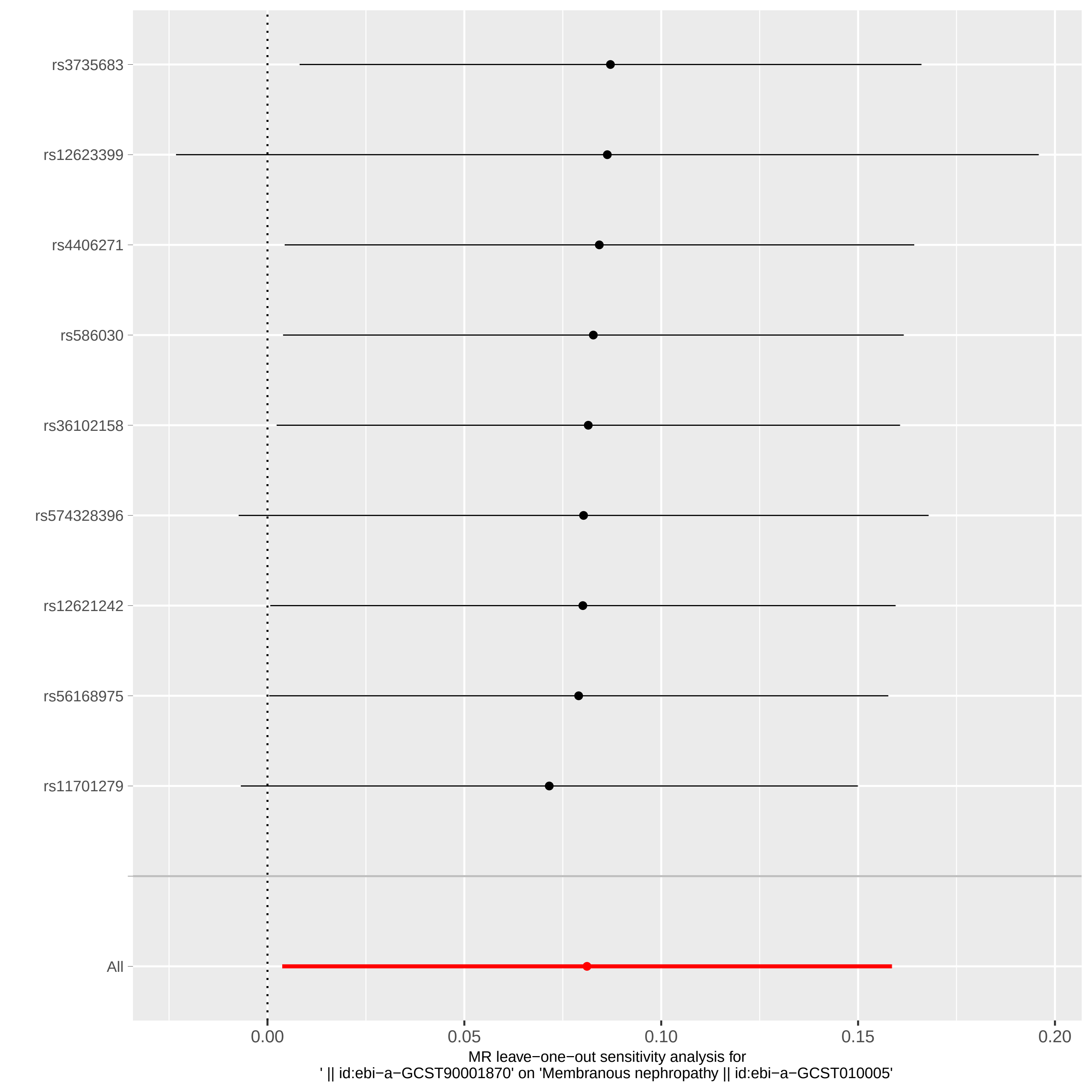
rs3735683
rs12623399
rs4406271
rs586030
rs36102158
rs574328396
rs12621242
rs56168975
rs11701279
All
0.00
0.05
0.10
0.15
0.20
MR leave−one−out sensitivity analysis for
' || id:ebi−a−GCST90001870' on 'Membranous nephropathy || id:ebi−a−GCST010005'

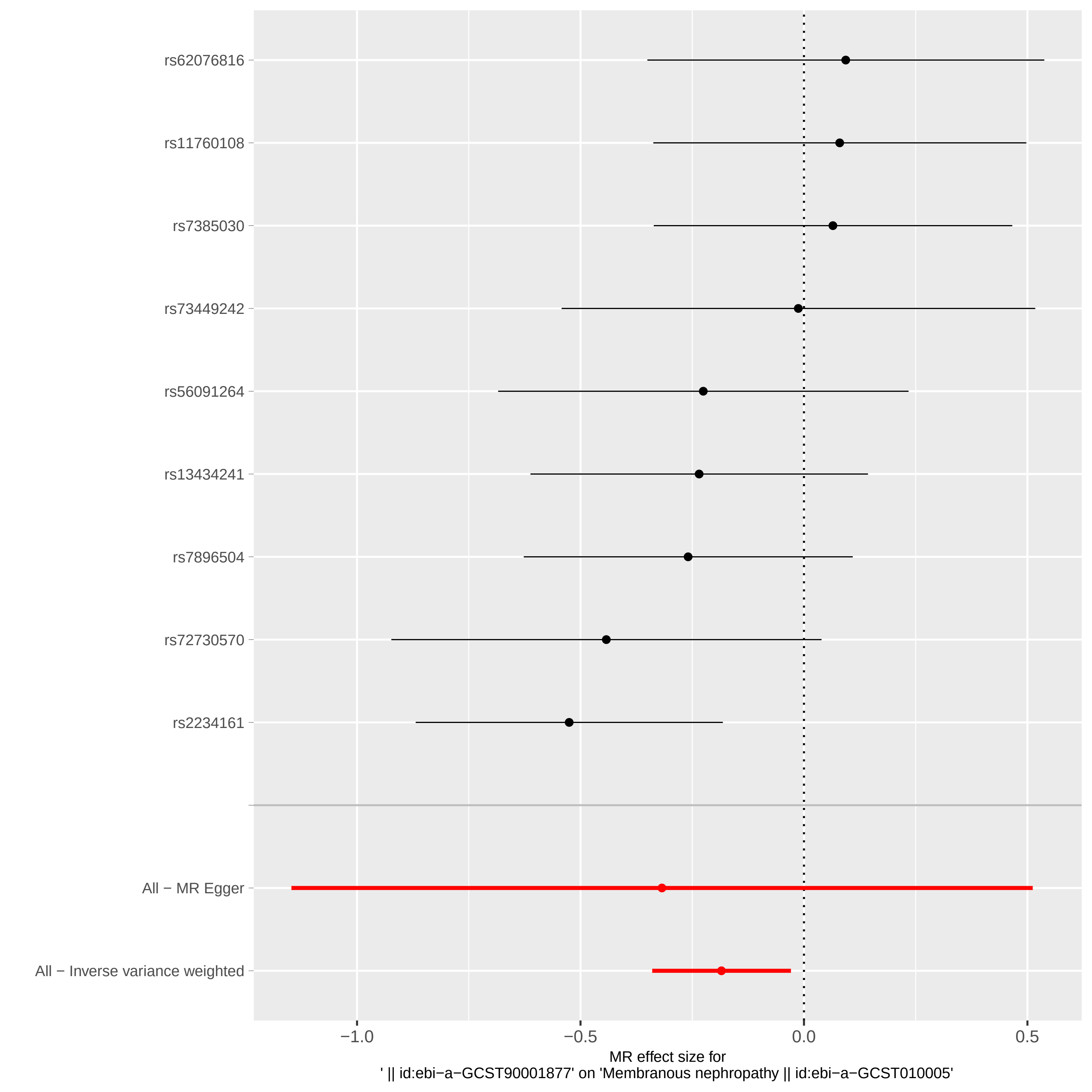
rs62076816
rs11760108
rs7385030
rs73449242
rs56091264
rs13434241
rs7896504
rs72730570
rs2234161
All − MR Egger
All − Inverse variance weighted
−1.0
−0.5
0.0
0.5
MR effect size for
' || id:ebi−a−GCST90001877' on 'Membranous nephropathy || id:ebi−a−GCST010005'

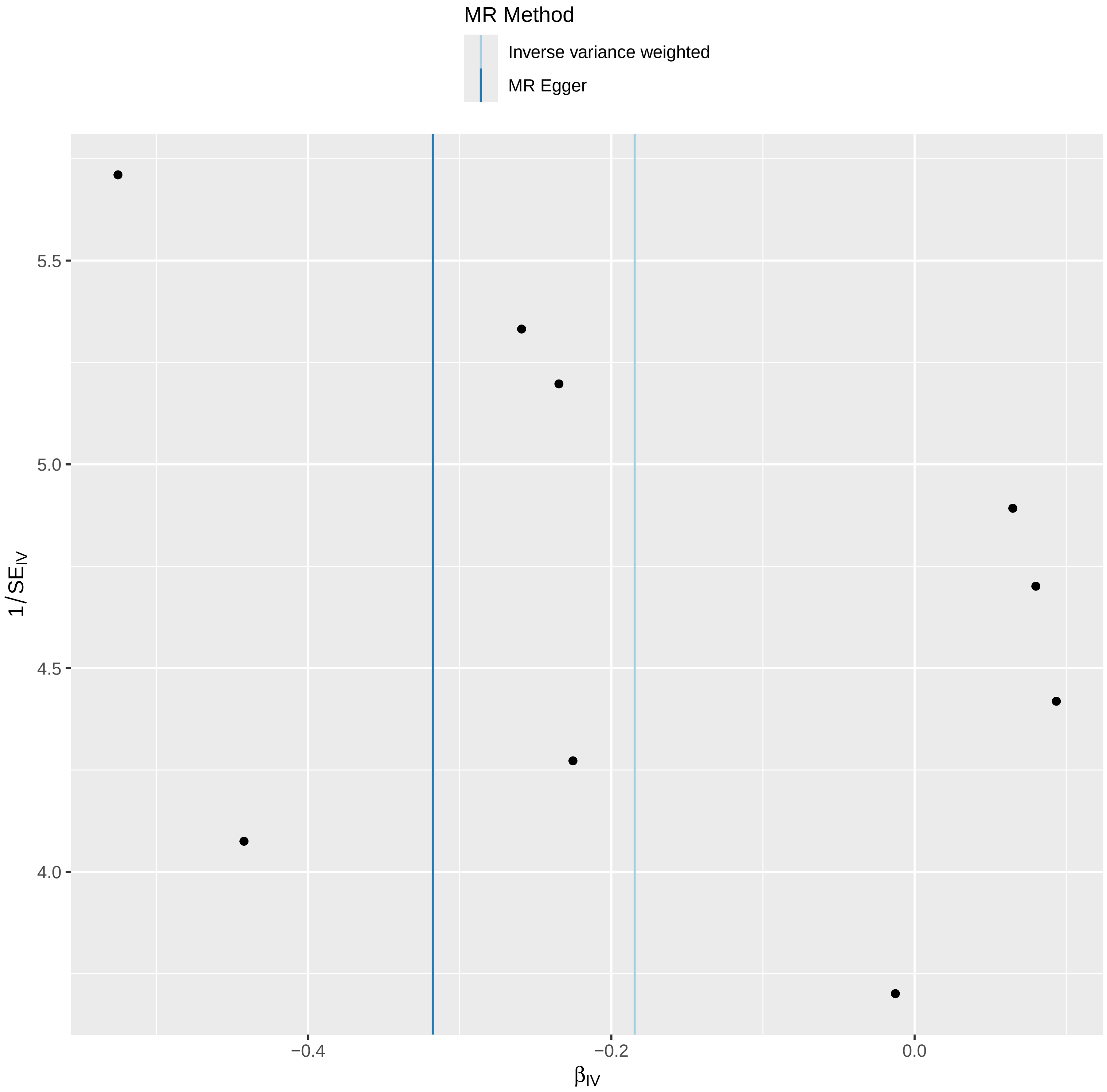

MR Method
Inverse variance weighted
MR Egger
5.5
5.0
4.5
4.0
1/SE_IV
−0.4
−0.2
0.0
β_IV

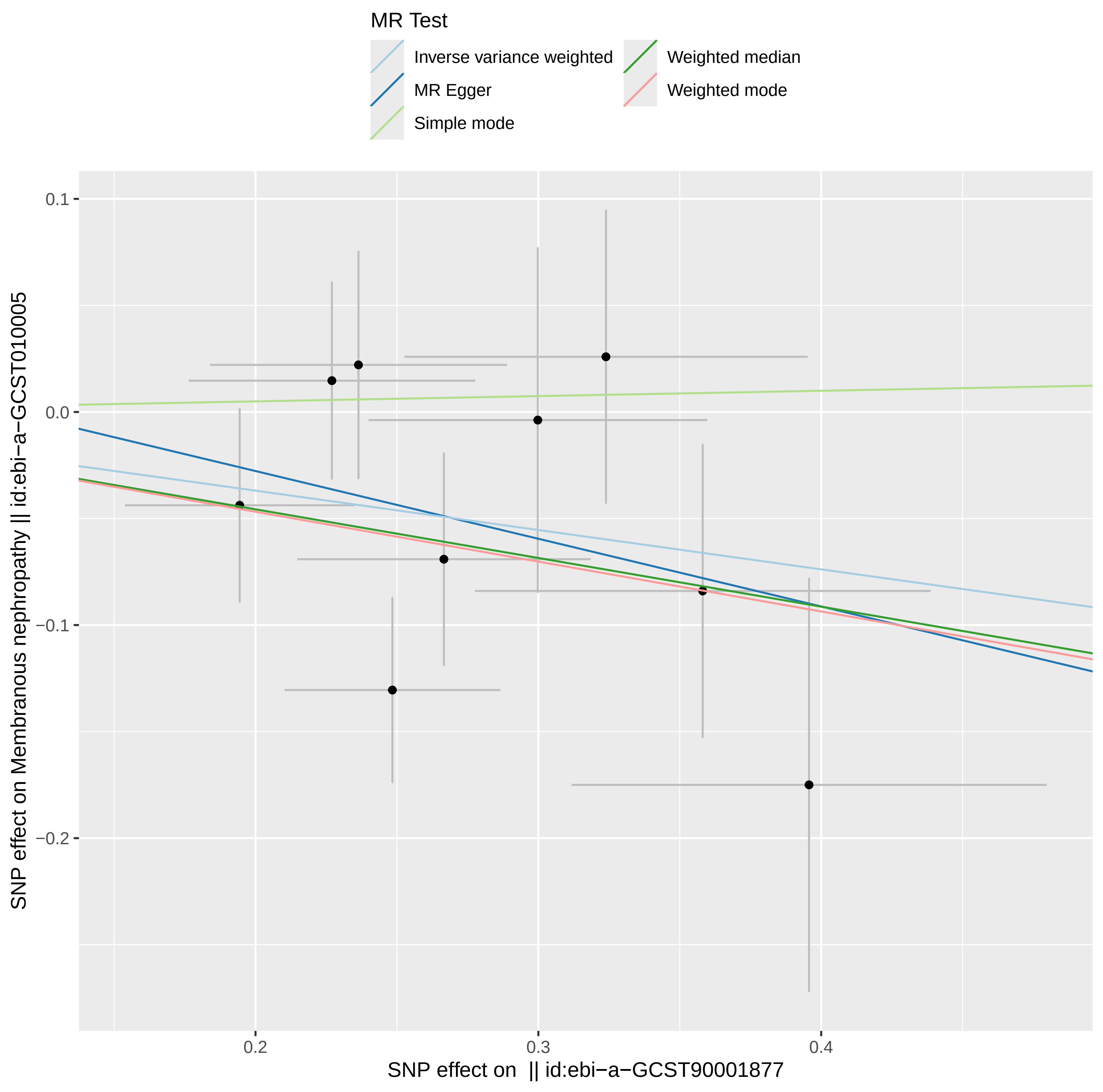
MR Test
Inverse variance weighted
MR Egger
Simple mode
Weighted median
Weighted mode
0.1
0.0
−0.1
−0.2
0.2
0.3
0.4
SNP effect on Membranous nephropathy || id:ebi−a−GCST010005
SNP effect on || id:ebi−a−GCST90001877

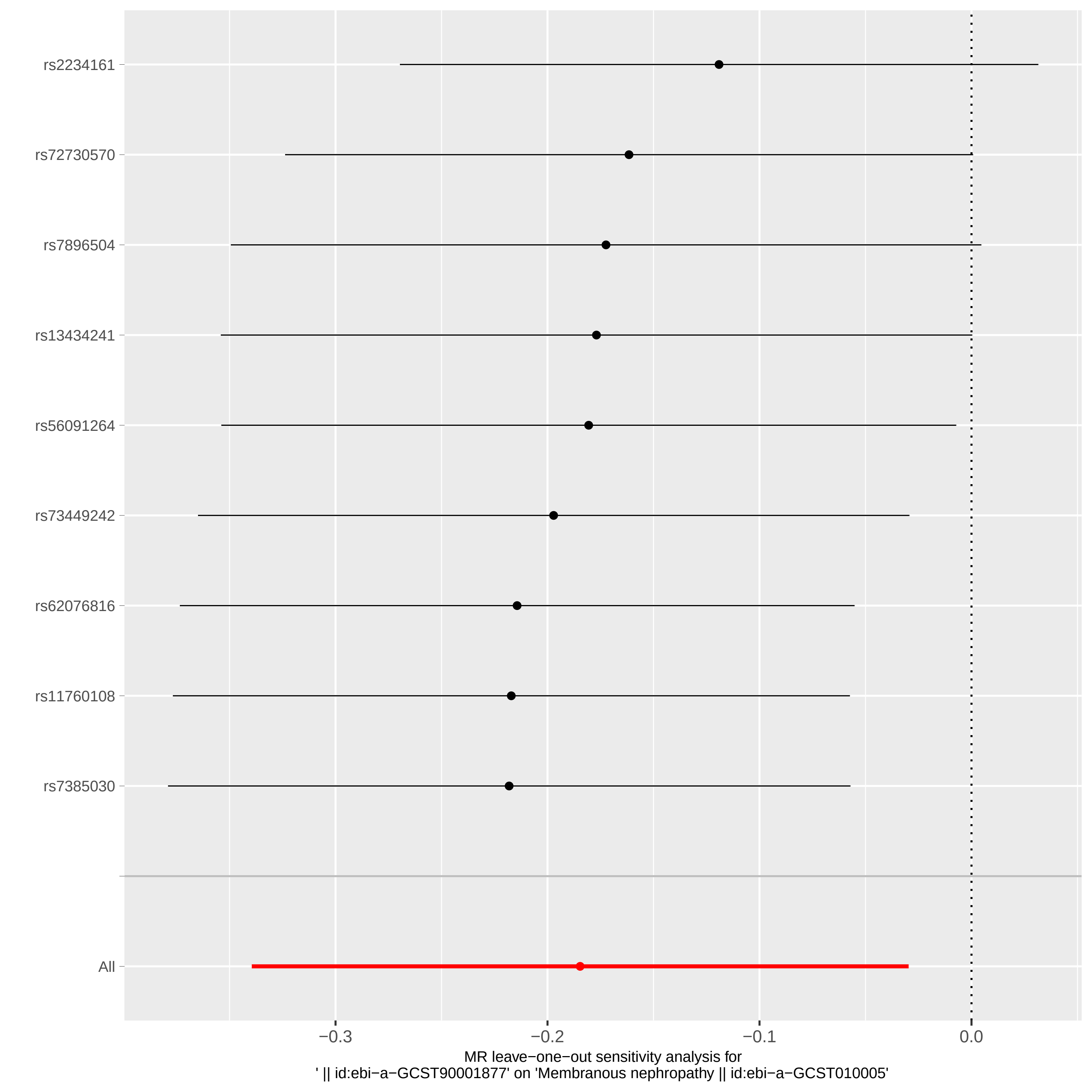
rs2234161
rs72730570
rs7896504
rs13434241
rs56091264
rs73449242
rs62076816
rs11760108
rs7385030
All
−0.3
−0.2
−0.1
0.0
MR leave−one−out sensitivity analysis for
' || id:ebi−a−GCST90001877' on 'Membranous nephropathy || id:ebi−a−GCST010005'

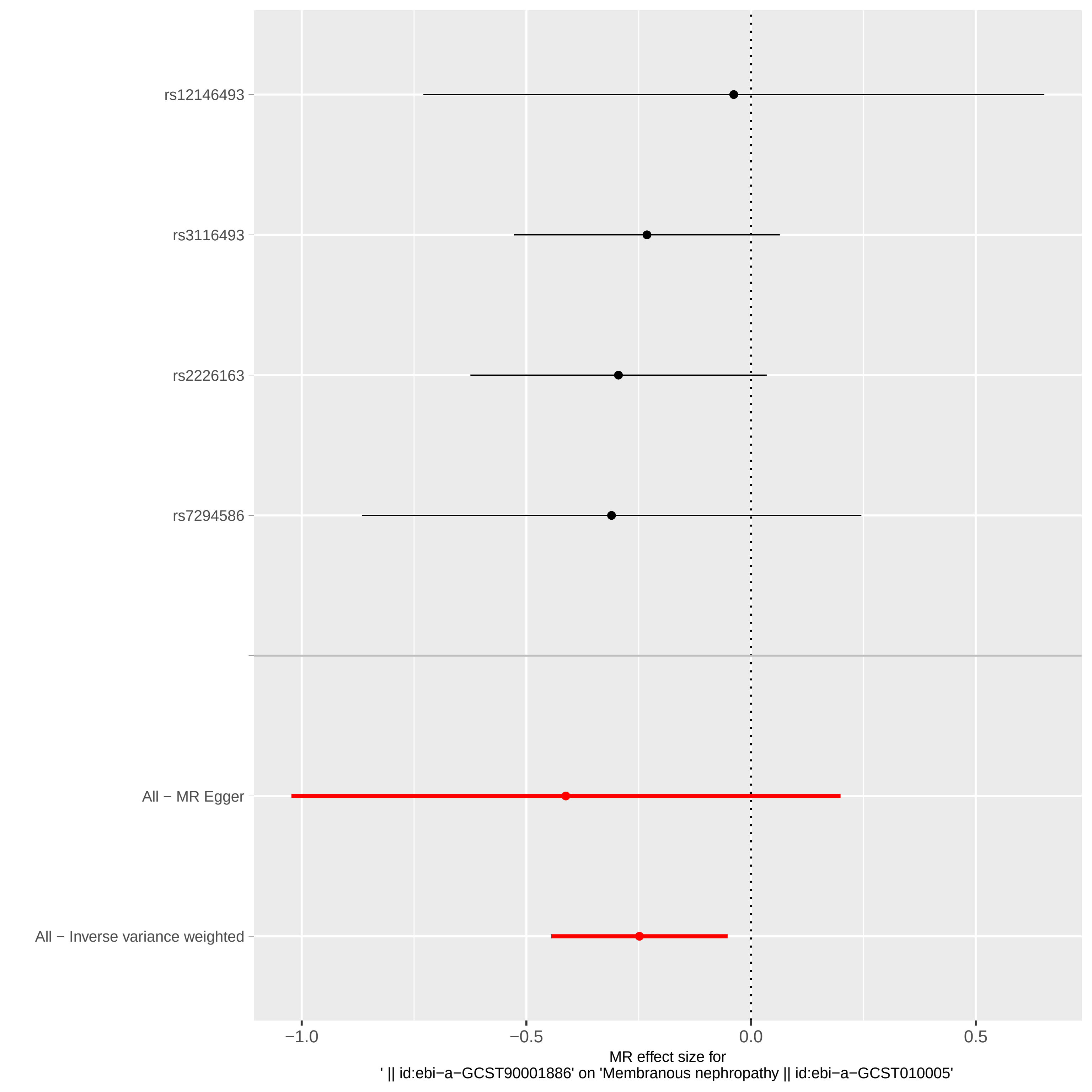
rs12146493
rs3116493
rs2226163
rs7294586
All − MR Egger
All − Inverse variance weighted
−1.0
−0.5
0.0
0.5
MR effect size for
' || id:ebi−a−GCST90001886' on 'Membranous nephropathy || id:ebi−a−GCST010005'

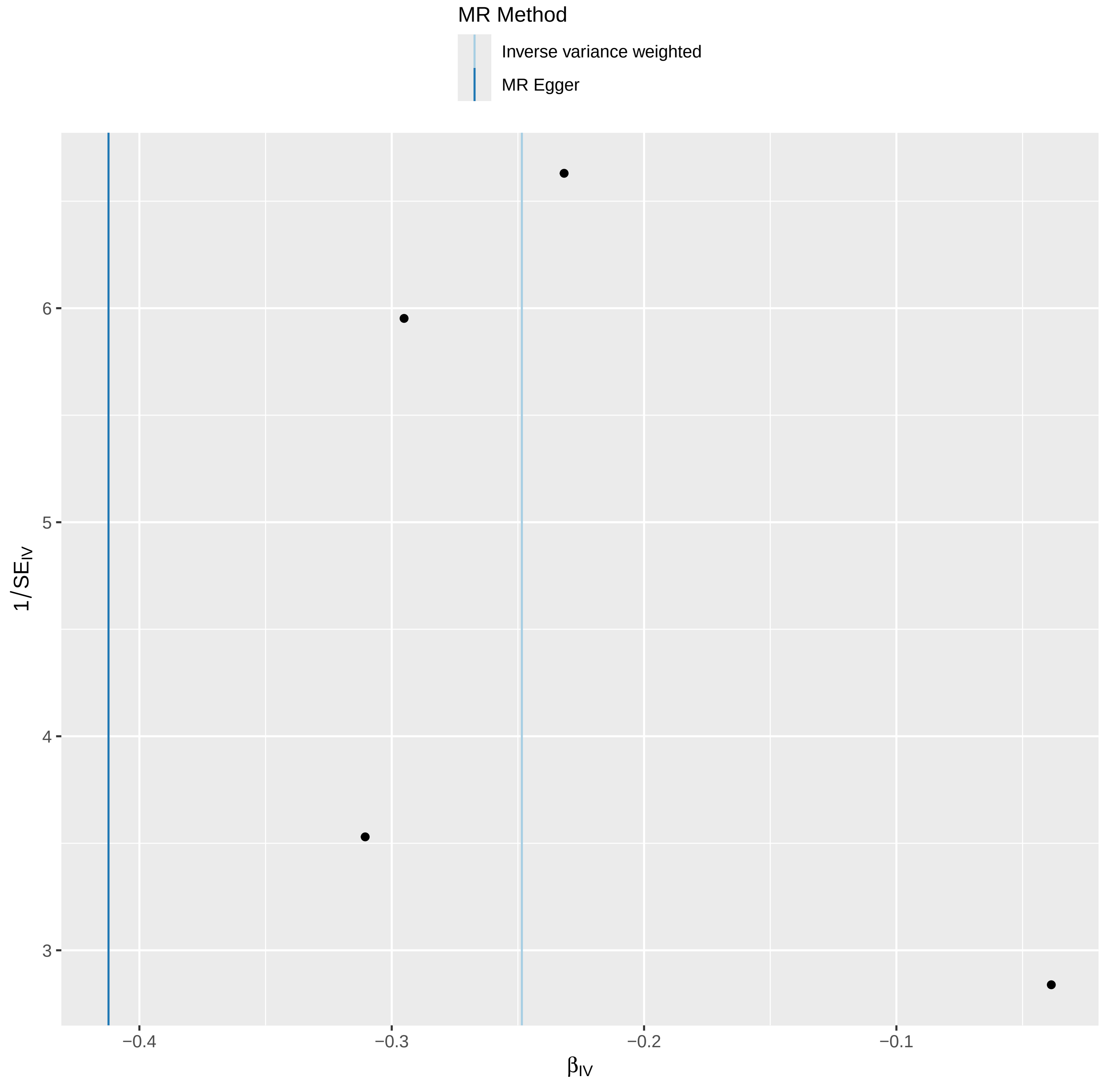
MR Method
Inverse variance weighted
MR Egger
1/SE$_{IV}$
6
5
4
3
β$_{IV}$
−0.4
−0.3
−0.2
−0.1

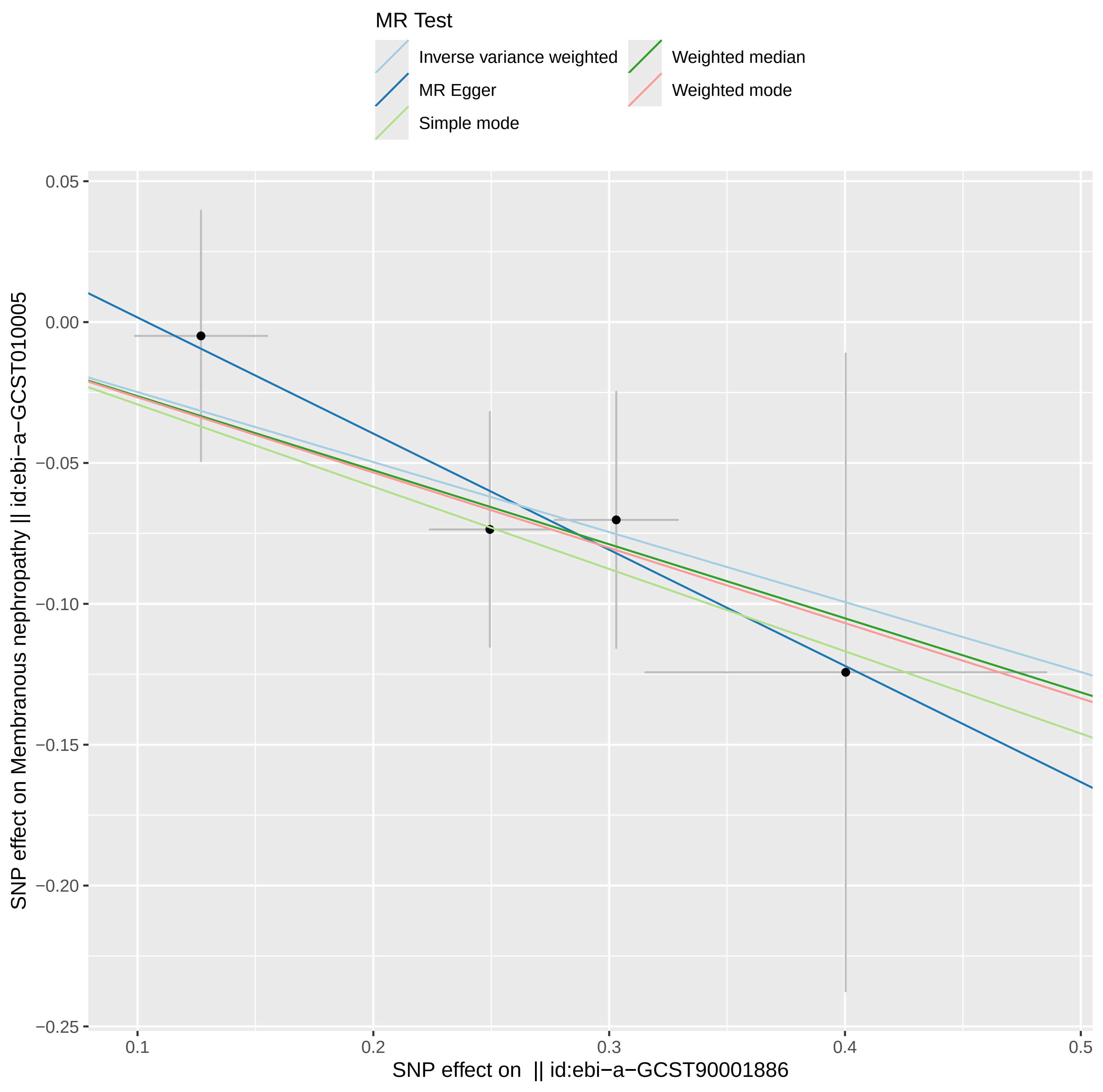

MR Test
Inverse variance weighted
MR Egger
Simple mode
Weighted median
Weighted mode
0.05
0.00
−0.05
−0.10
−0.15
−0.20
−0.25
0.1
0.2
0.3
0.4
0.5
SNP effect on Membranous nephropathy || id:ebi−a−GCST010005
SNP effect on || id:ebi−a−GCST90001886

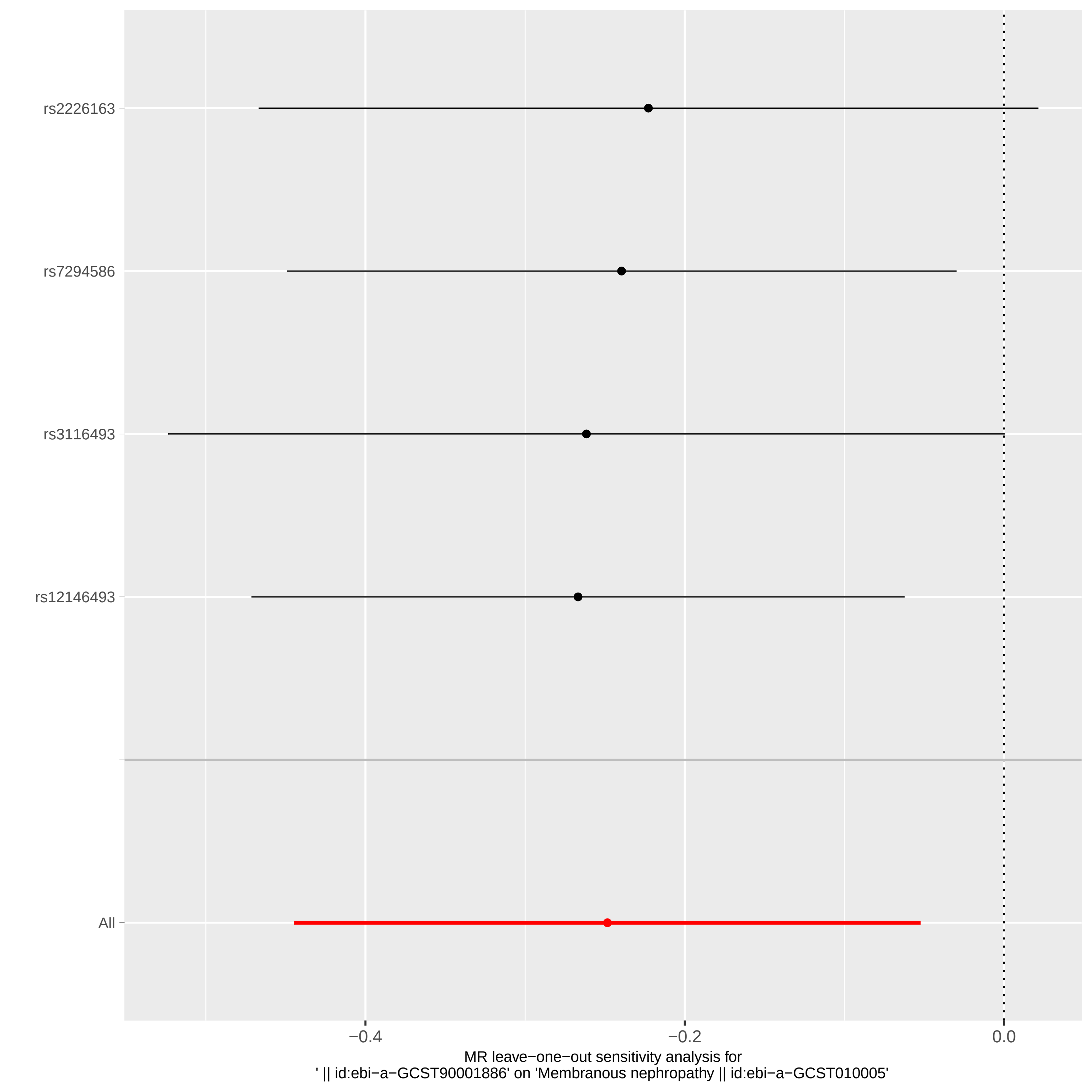
rs2226163
rs7294586
rs3116493
rs12146493
All
−0.4
−0.2
0.0
MR leave−one−out sensitivity analysis for
' || id:ebi−a−GCST90001886' on 'Membranous nephropathy || id:ebi−a−GCST010005'

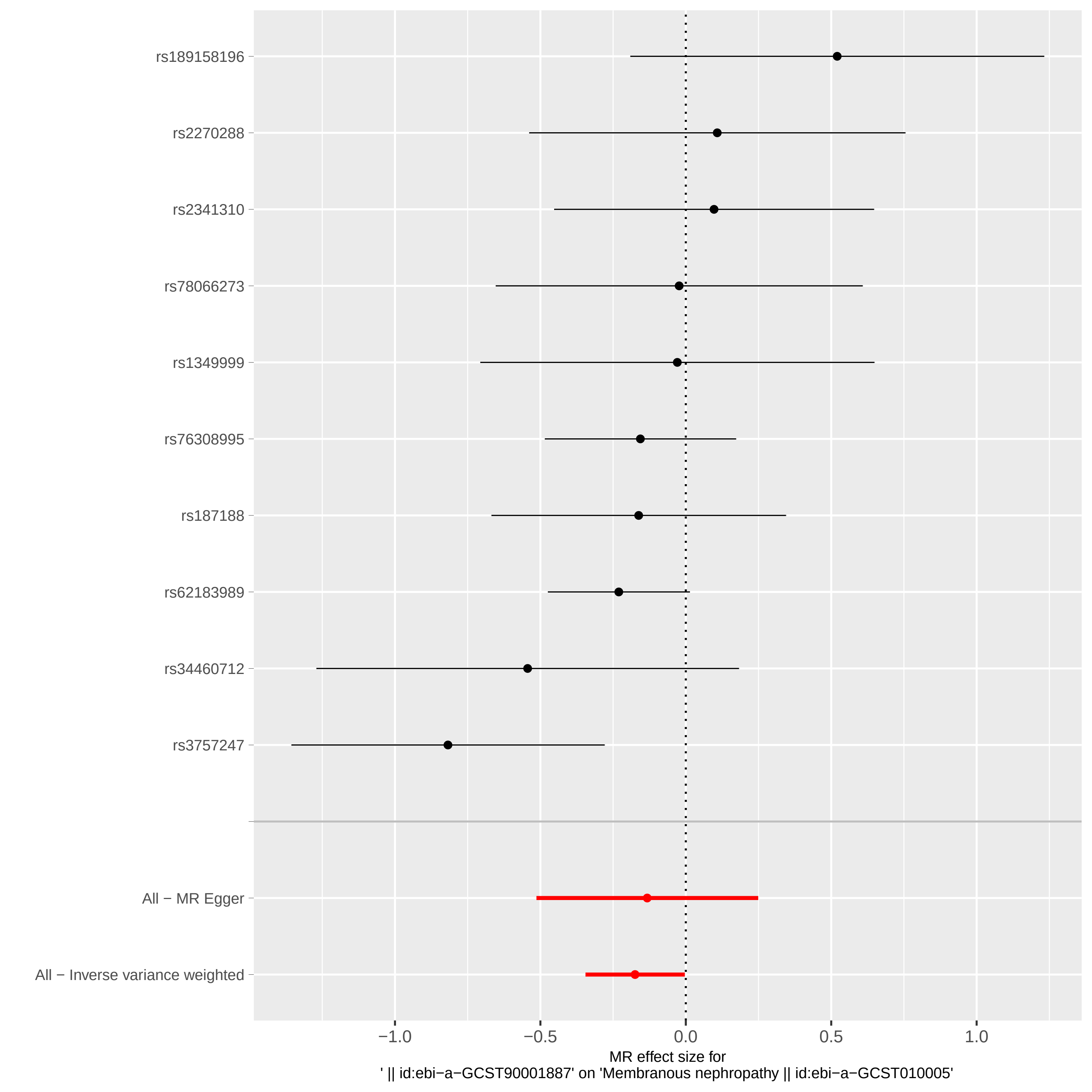
rs189158196
rs2270288
rs2341310
rs78066273
rs1349999
rs76308995
rs187188
rs62183989
rs34460712
rs3757247
All − MR Egger
All − Inverse variance weighted
−1.0
−0.5
0.0
0.5
1.0
MR effect size for
' || id:ebi−a−GCST90001887' on 'Membranous nephropathy || id:ebi−a−GCST010005'

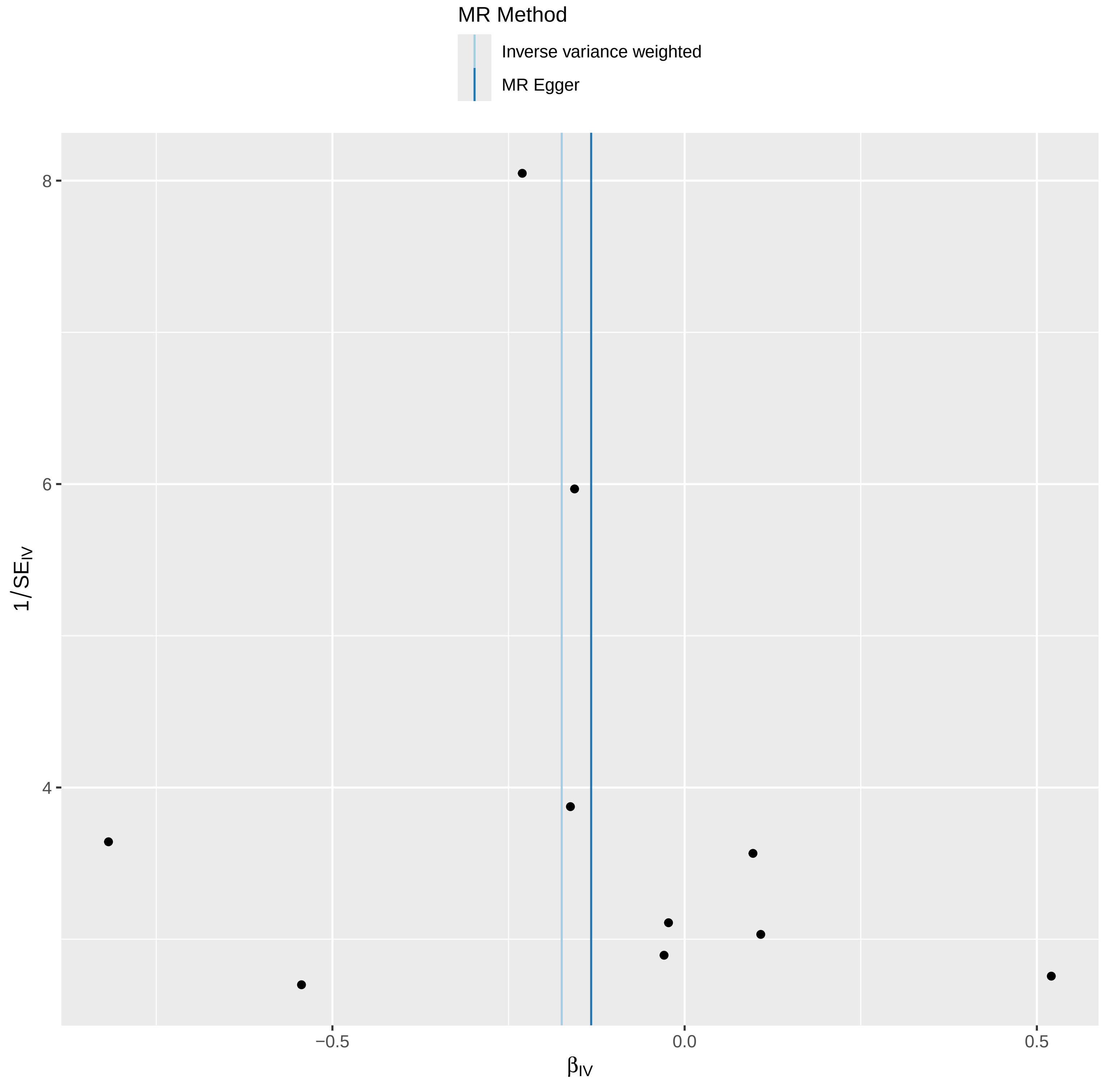

MR Method
Inverse variance weighted
MR Egger
8
6
4
1/SE$_{IV}$
−0.5
0.0
0.5
β$_{IV}$

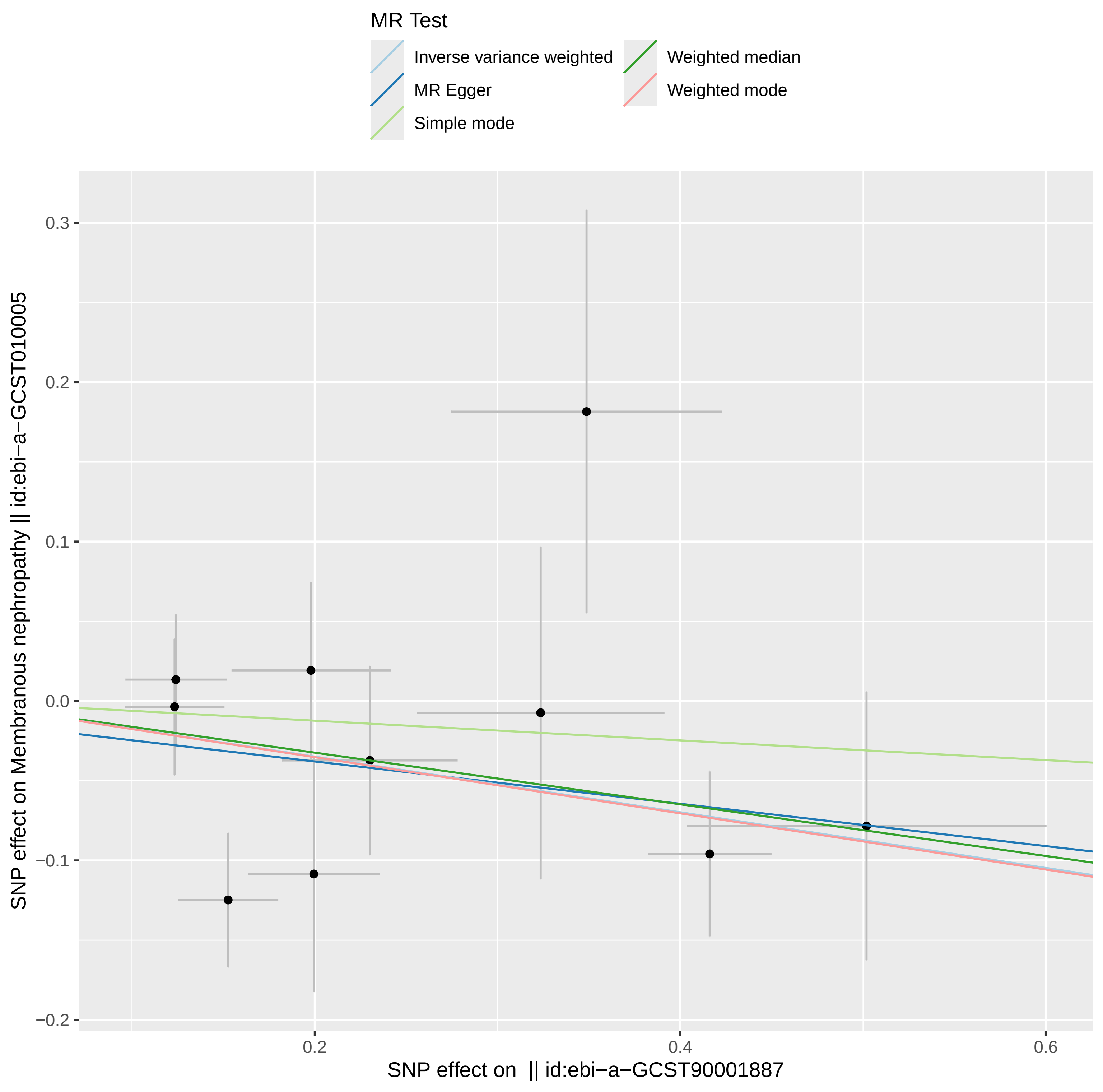
MR Test
Inverse variance weighted
MR Egger
Simple mode
Weighted median
Weighted mode
SNP effect on Membranous nephropathy || id:ebi−a−GCST010005
SNP effect on || id:ebi−a−GCST90001887
0.3
0.2
0.1
0.0
−0.1
−0.2
0.2
0.4
0.6

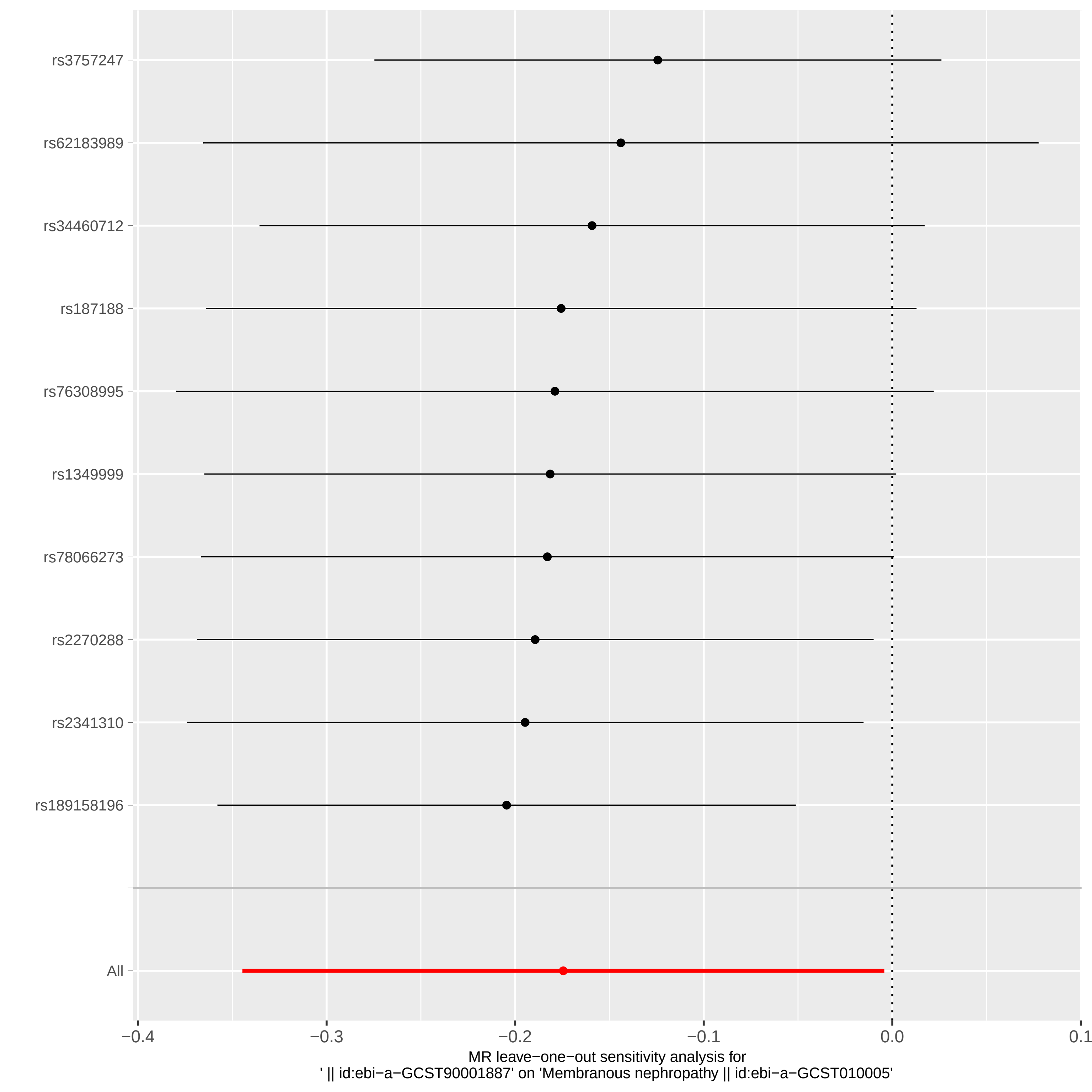
rs3757247
rs62183989
rs34460712
rs187188
rs76308995
rs1349999
rs78066273
rs2270288
rs2341310
rs189158196
All
−0.4
−0.3
−0.2
−0.1
0.0
0.1
MR leave−one−out sensitivity analysis for
' || id:ebi−a−GCST90001887' on 'Membranous nephropathy || id:ebi−a−GCST010005'

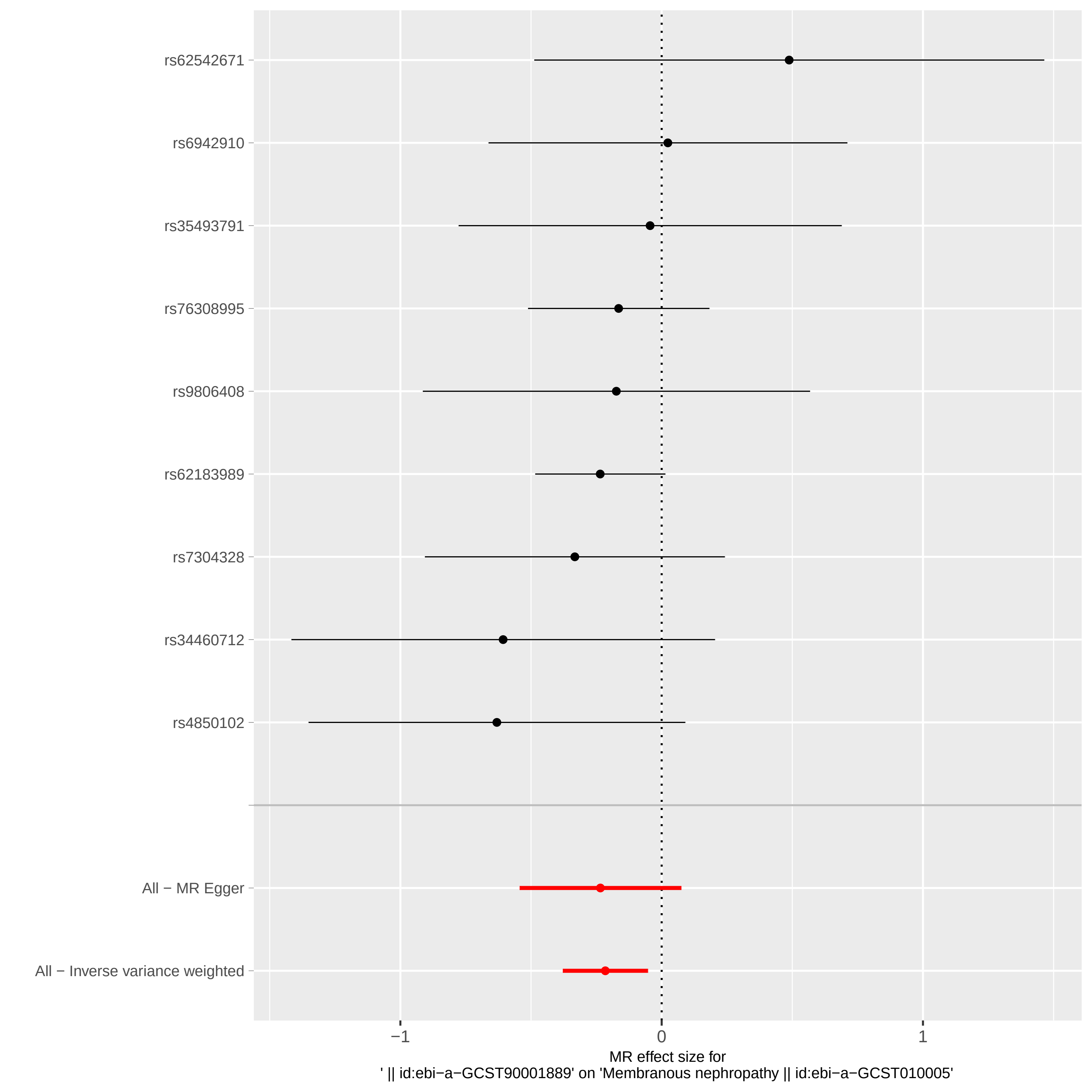

rs62542671
rs6942910
rs35493791
rs76308995
rs9806408
rs62183989
rs7304328
rs34460712
rs4850102
All − MR Egger
All − Inverse variance weighted
−1
0
1
MR effect size for
' || id:ebi−a−GCST90001889' on 'Membranous nephropathy || id:ebi−a−GCST010005'

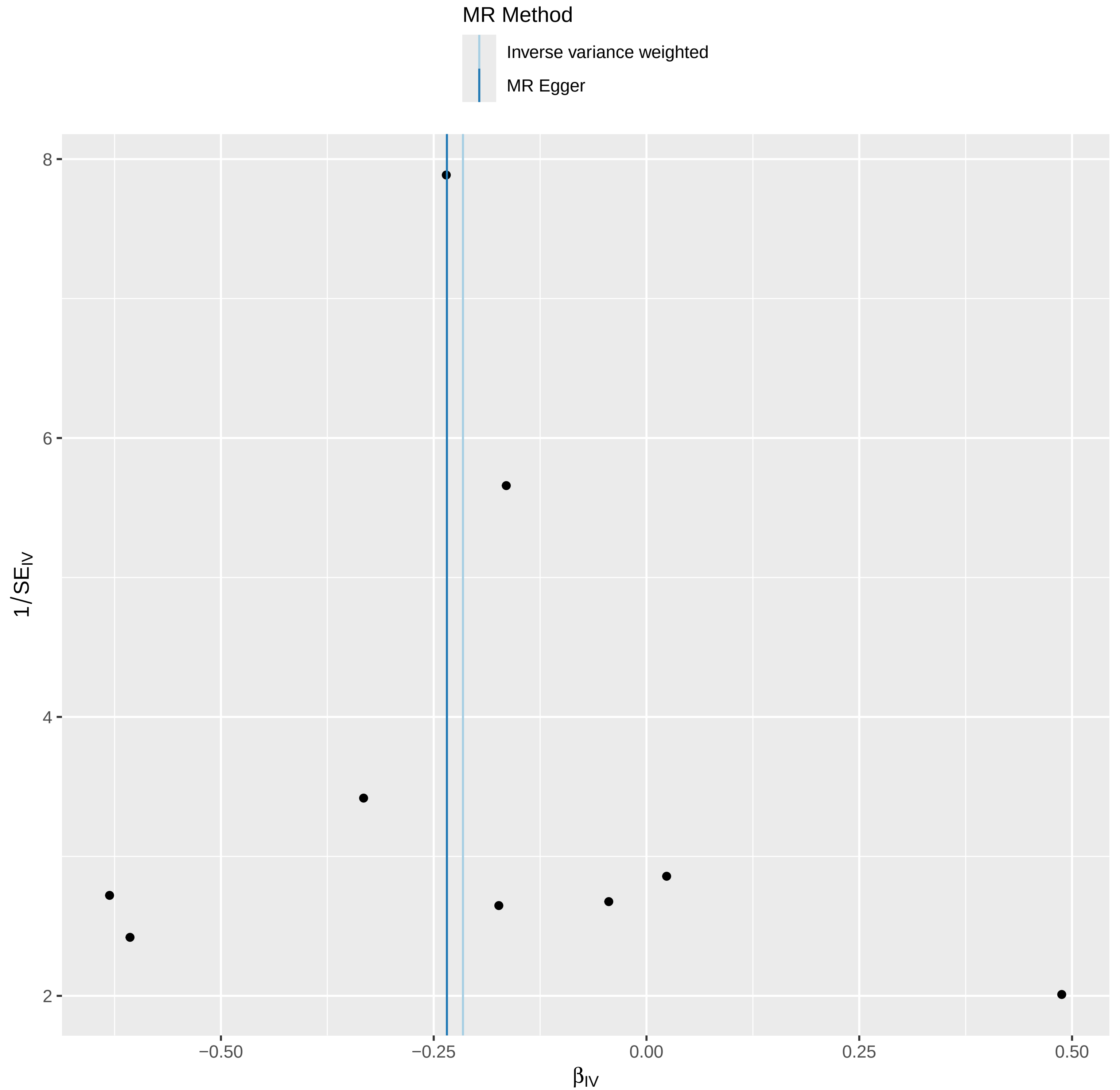
MR Method
Inverse variance weighted
MR Egger
1/SE_IV
β_IV
−0.50
−0.25
0.00
0.25
0.50
2
4
6
8

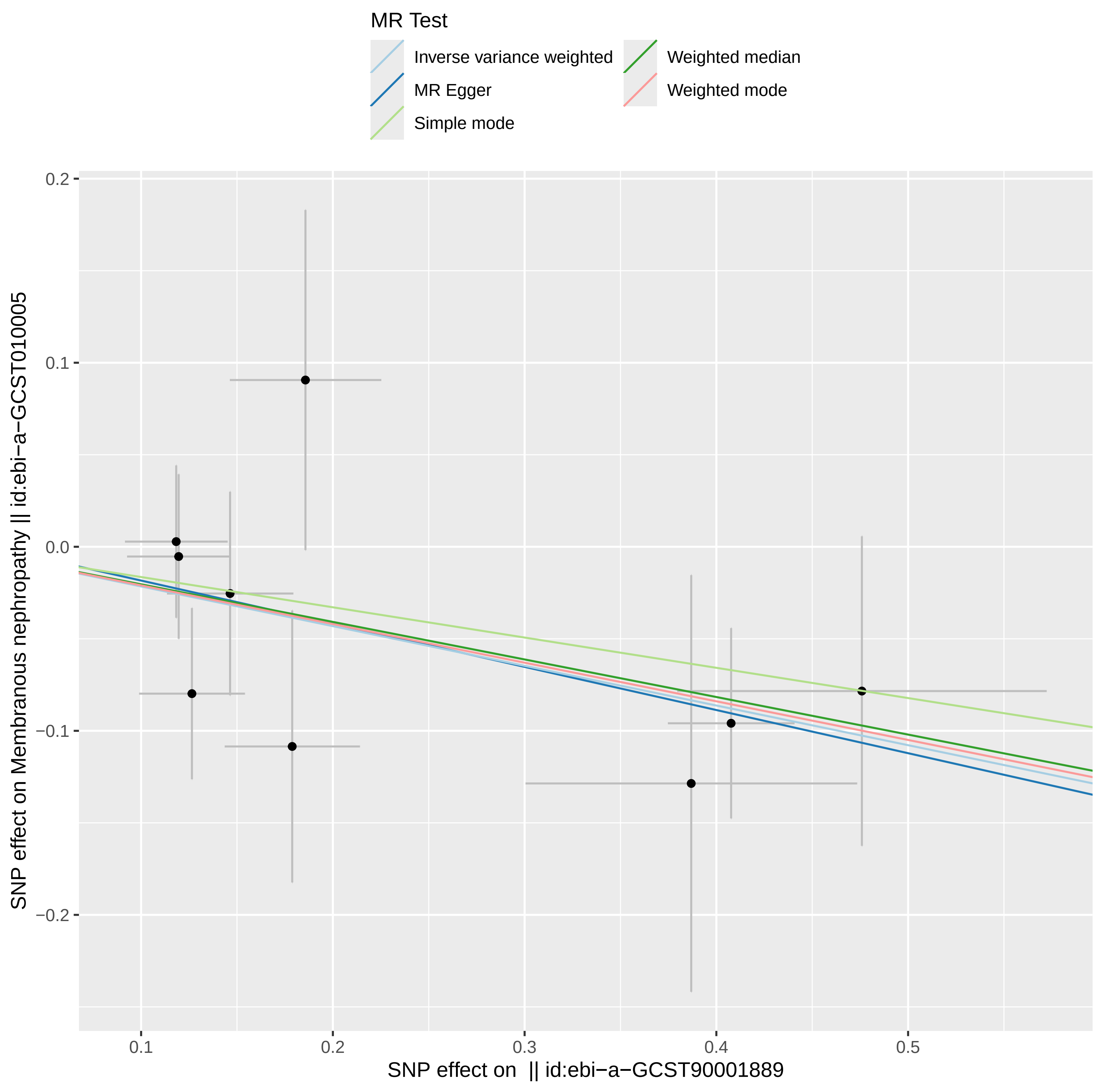

MR Test
Inverse variance weighted
MR Egger
Simple mode
Weighted median
Weighted mode
SNP effect on Membranous nephropathy || id:ebi−a−GCST010005
0.2
0.1
0.0
−0.1
−0.2
0.1
0.2
0.3
0.4
0.5
SNP effect on || id:ebi−a−GCST90001889

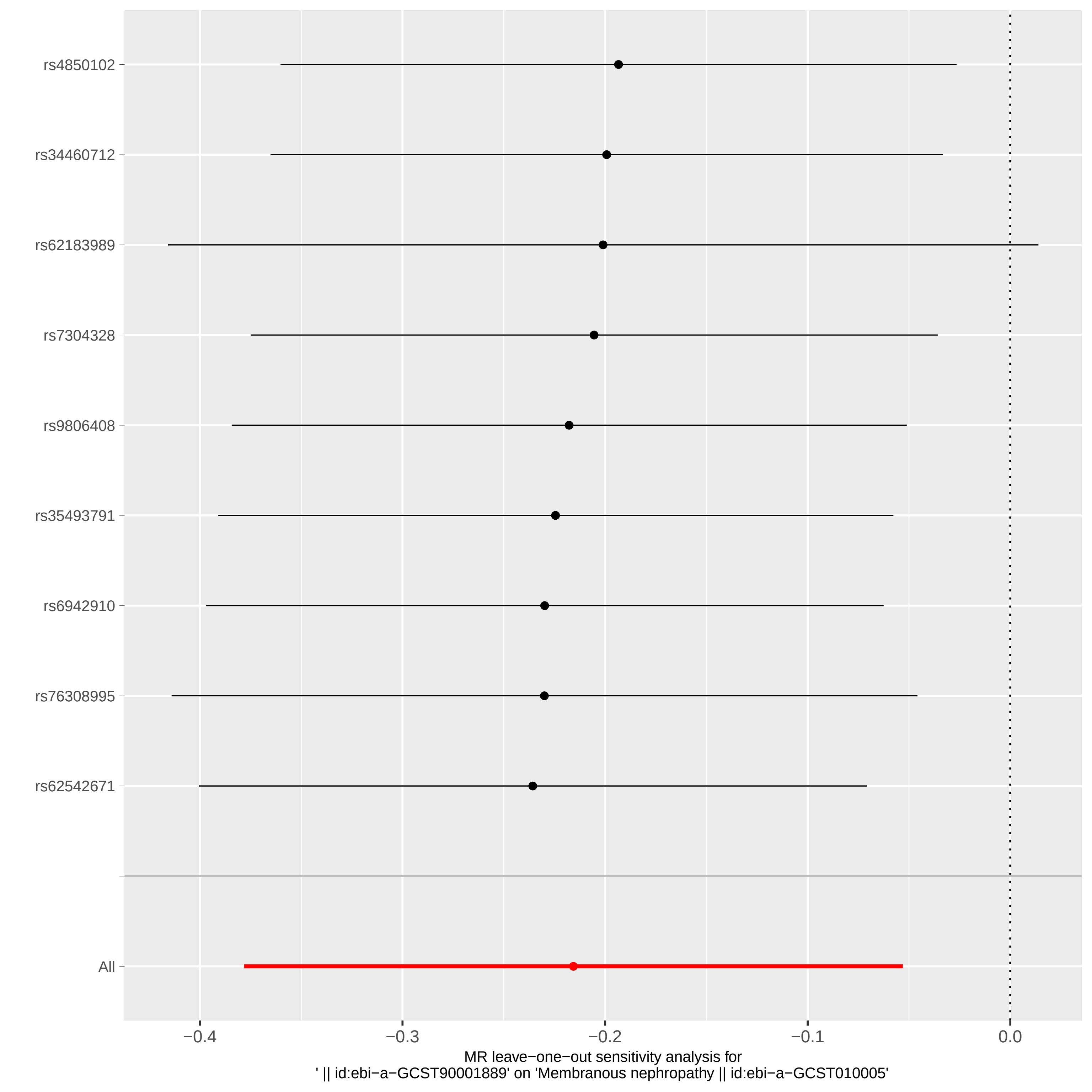

rs4850102
rs34460712
rs62183989
rs7304328
rs9806408
rs35493791
rs6942910
rs76308995
rs62542671
All
−0.4
−0.3
−0.2
−0.1
0.0
MR leave−one−out sensitivity analysis for
' || id:ebi−a−GCST90001889' on 'Membranous nephropathy || id:ebi−a−GCST010005'

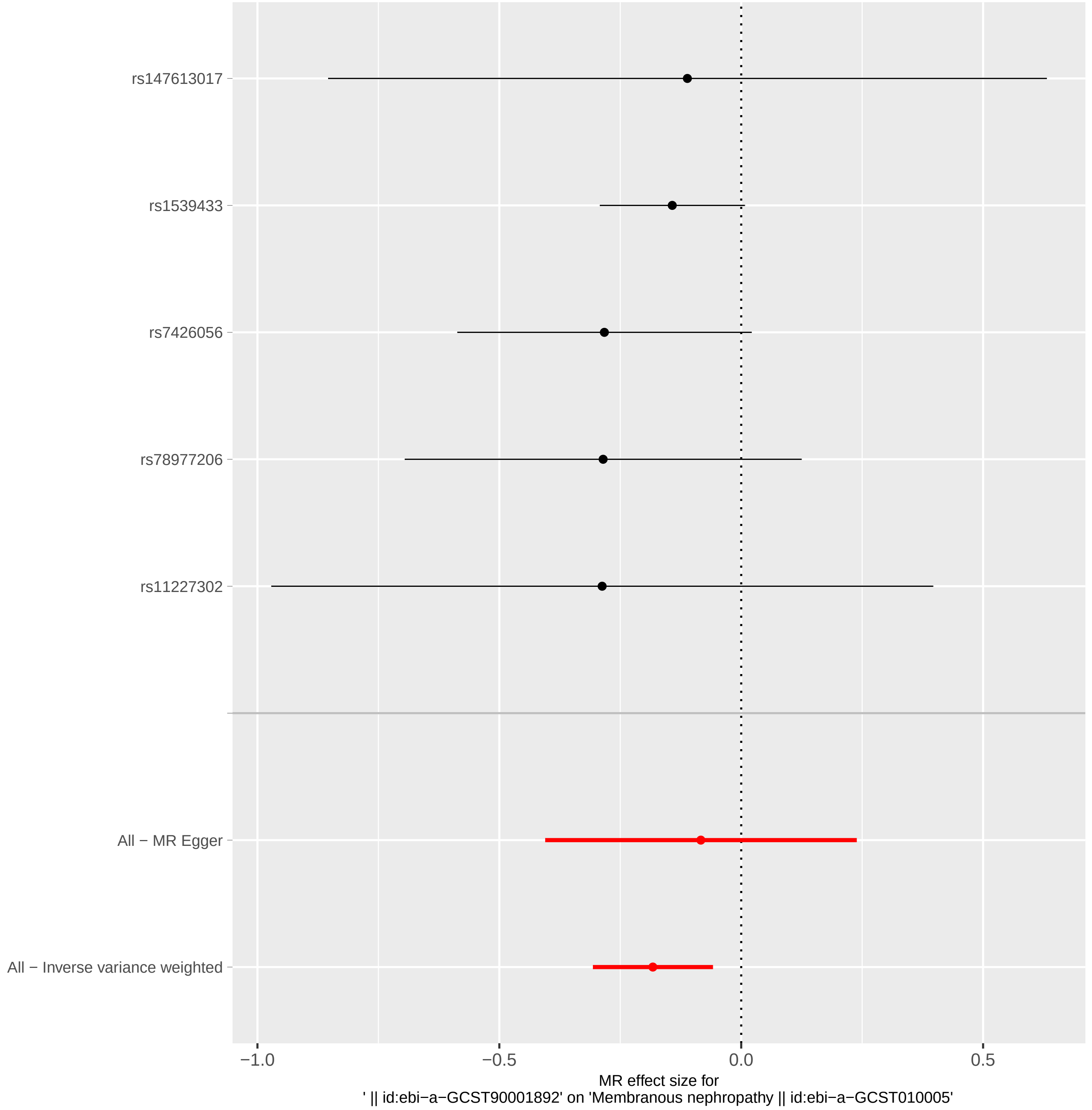
rs147613017
rs1539433
rs7426056
rs78977206
rs11227302
All − MR Egger
All − Inverse variance weighted
−1.0
−0.5
0.0
0.5
MR effect size for
' || id:ebi−a−GCST90001892' on 'Membranous nephropathy || id:ebi−a−GCST010005'

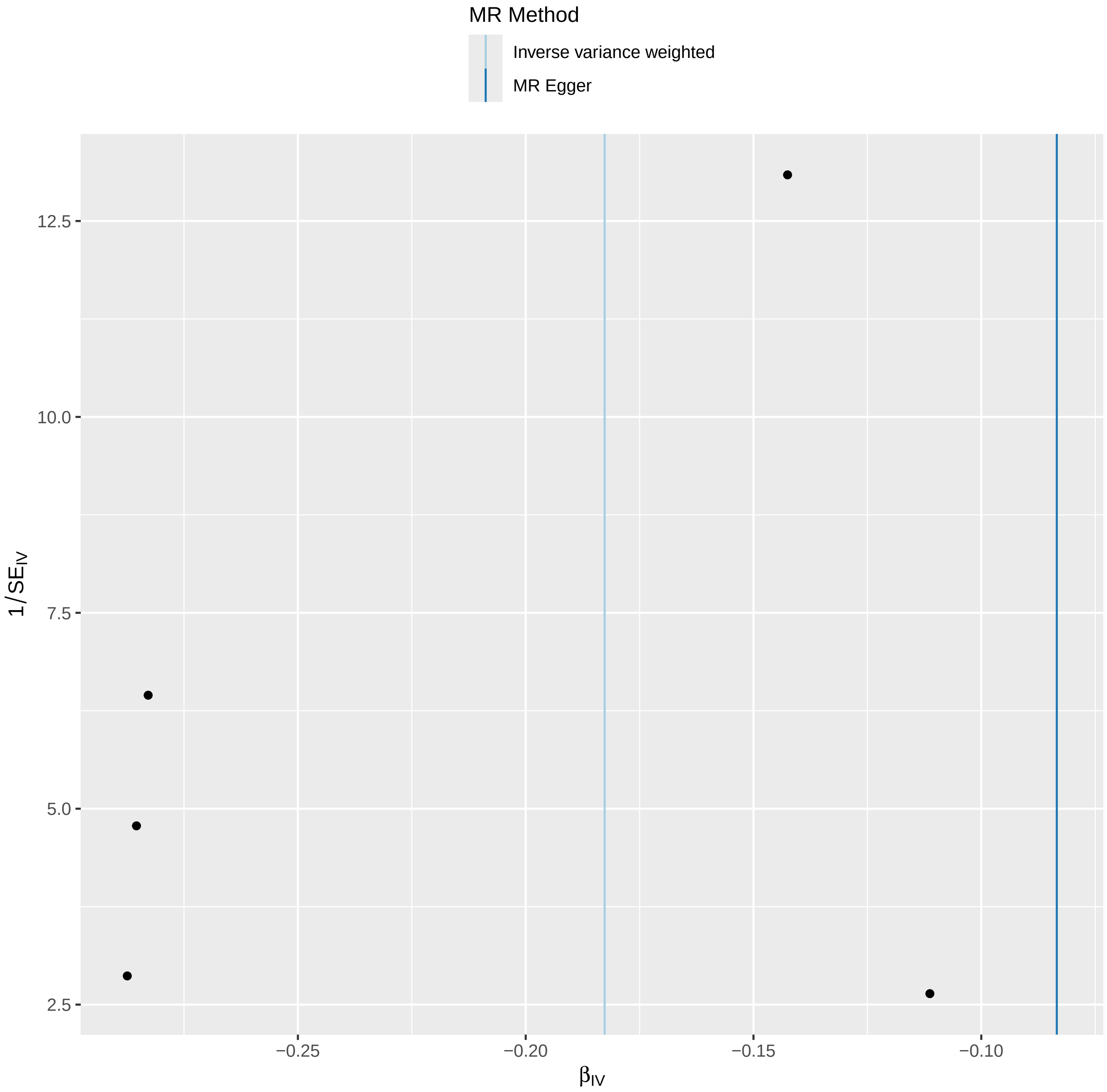

MR Method
Inverse variance weighted
MR Egger
12.5
10.0
7.5
5.0
2.5
1/SE$_{IV}$
−0.25
−0.20
−0.15
−0.10
β$_{IV}$

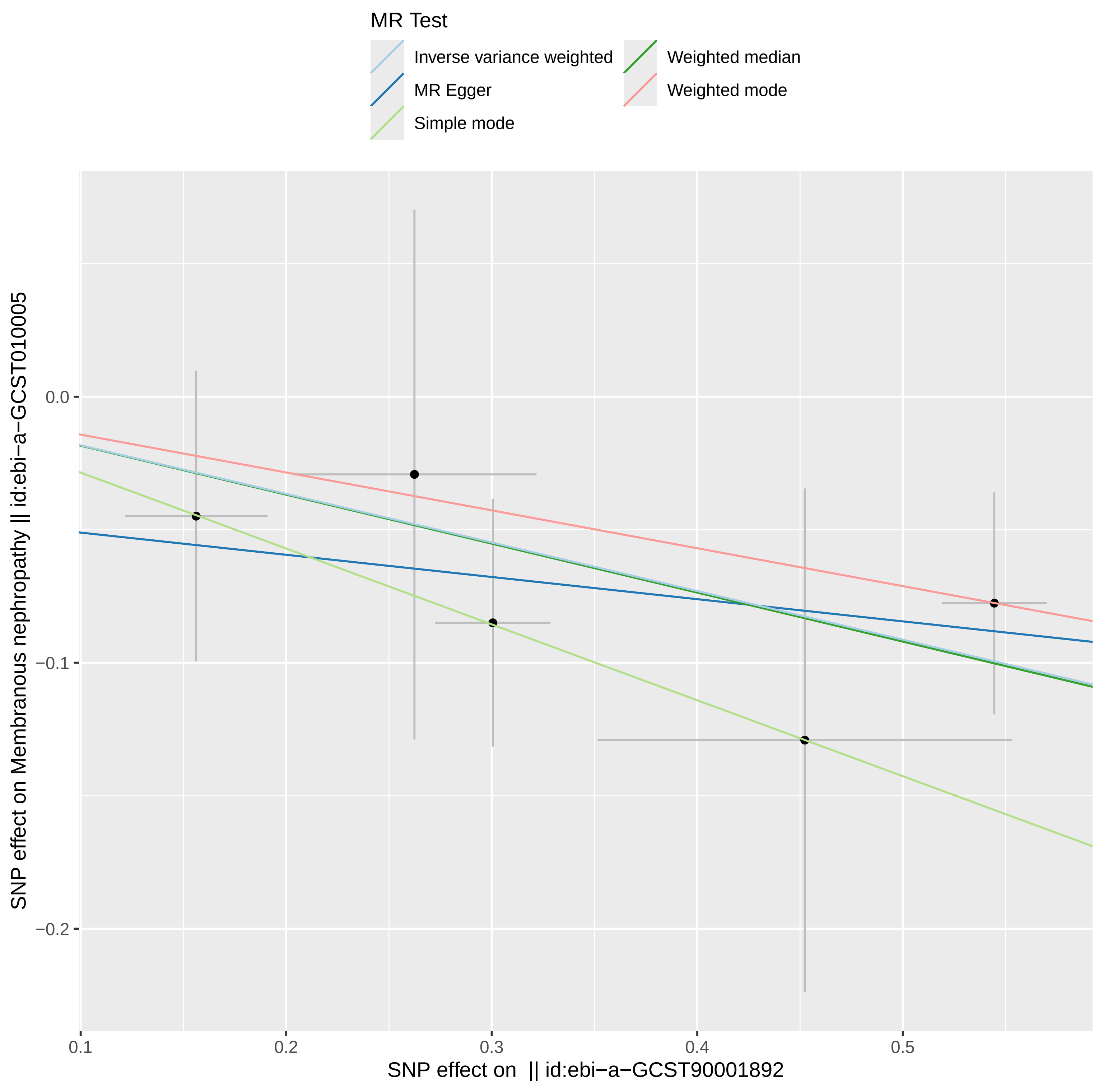
MR Test
Inverse variance weighted
MR Egger
Simple mode
Weighted median
Weighted mode
0.0
−0.1
−0.2
0.1
0.2
0.3
0.4
0.5
SNP effect on Membranous nephropathy || id:ebi−a−GCST010005
SNP effect on || id:ebi−a−GCST90001892

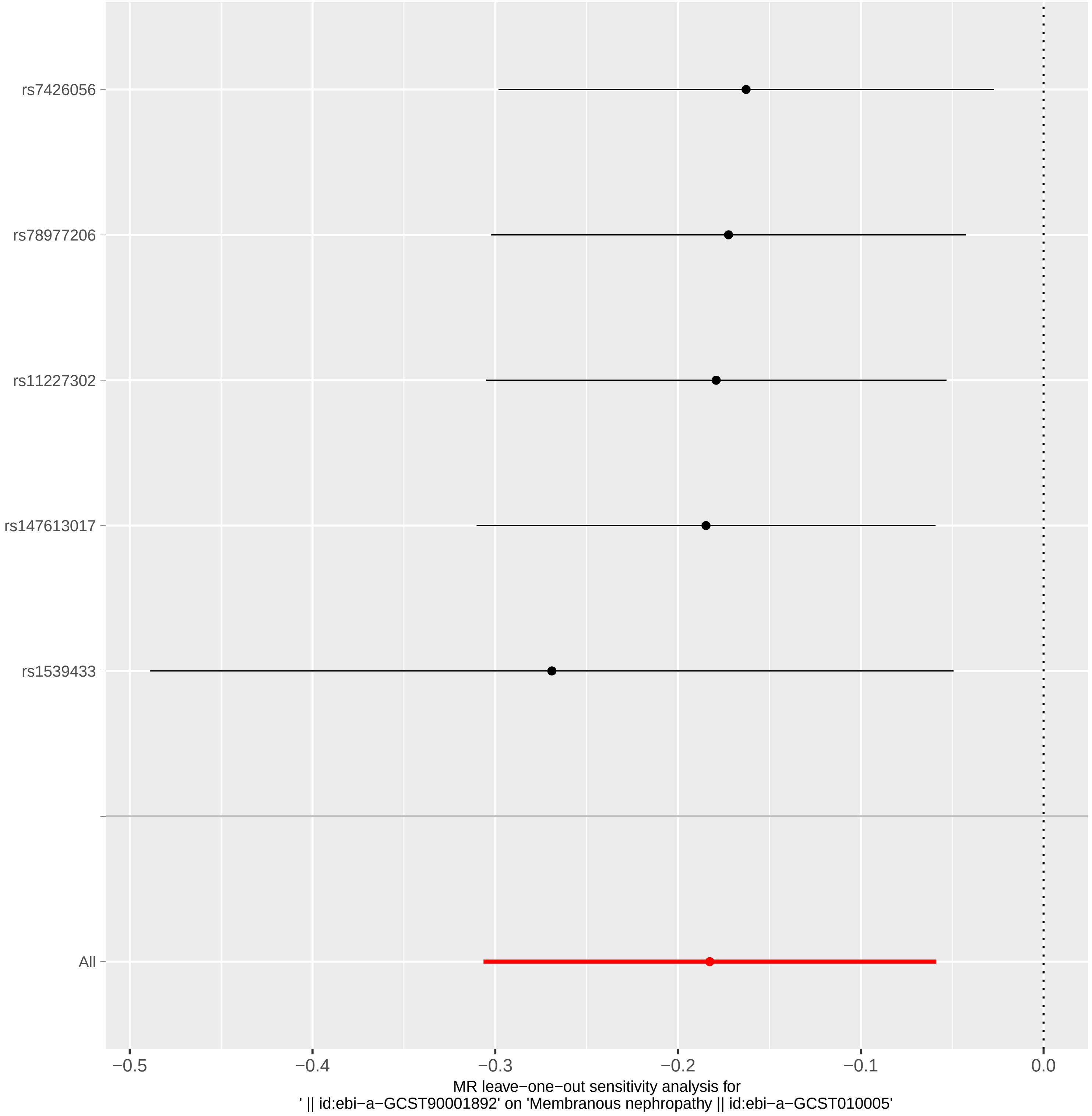
rs7426056
rs78977206
rs11227302
rs147613017
rs1539433
All
−0.5
−0.4
−0.3
−0.2
−0.1
0.0
MR leave−one−out sensitivity analysis for
' || id:ebi−a−GCST90001892' on 'Membranous nephropathy || id:ebi−a−GCST010005'

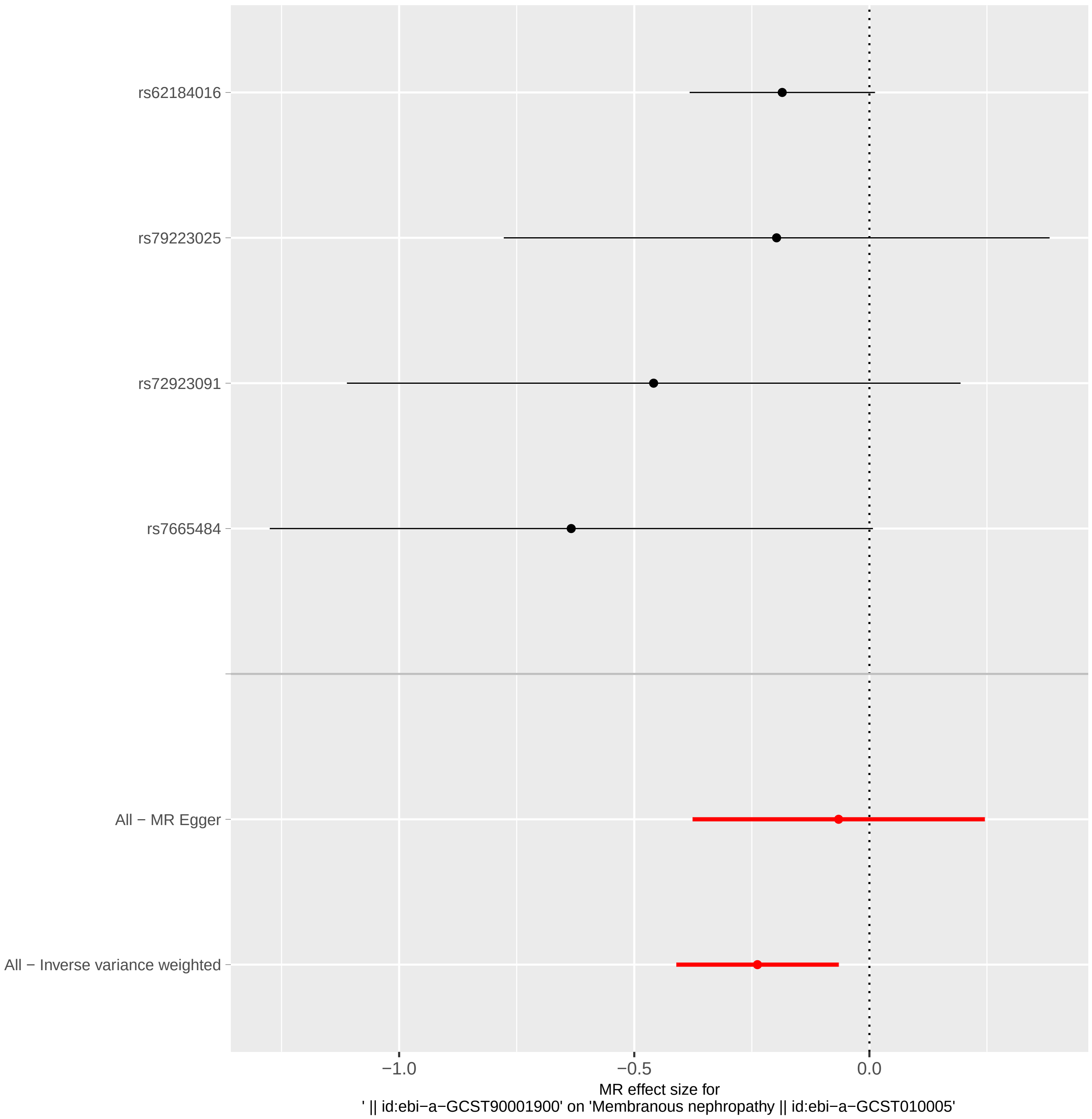
rs62184016
rs79223025
rs72923091
rs7665484
All − MR Egger
All − Inverse variance weighted
−1.0
−0.5
0.0
MR effect size for
' || id:ebi−a−GCST90001900' on 'Membranous nephropathy || id:ebi−a−GCST010005'

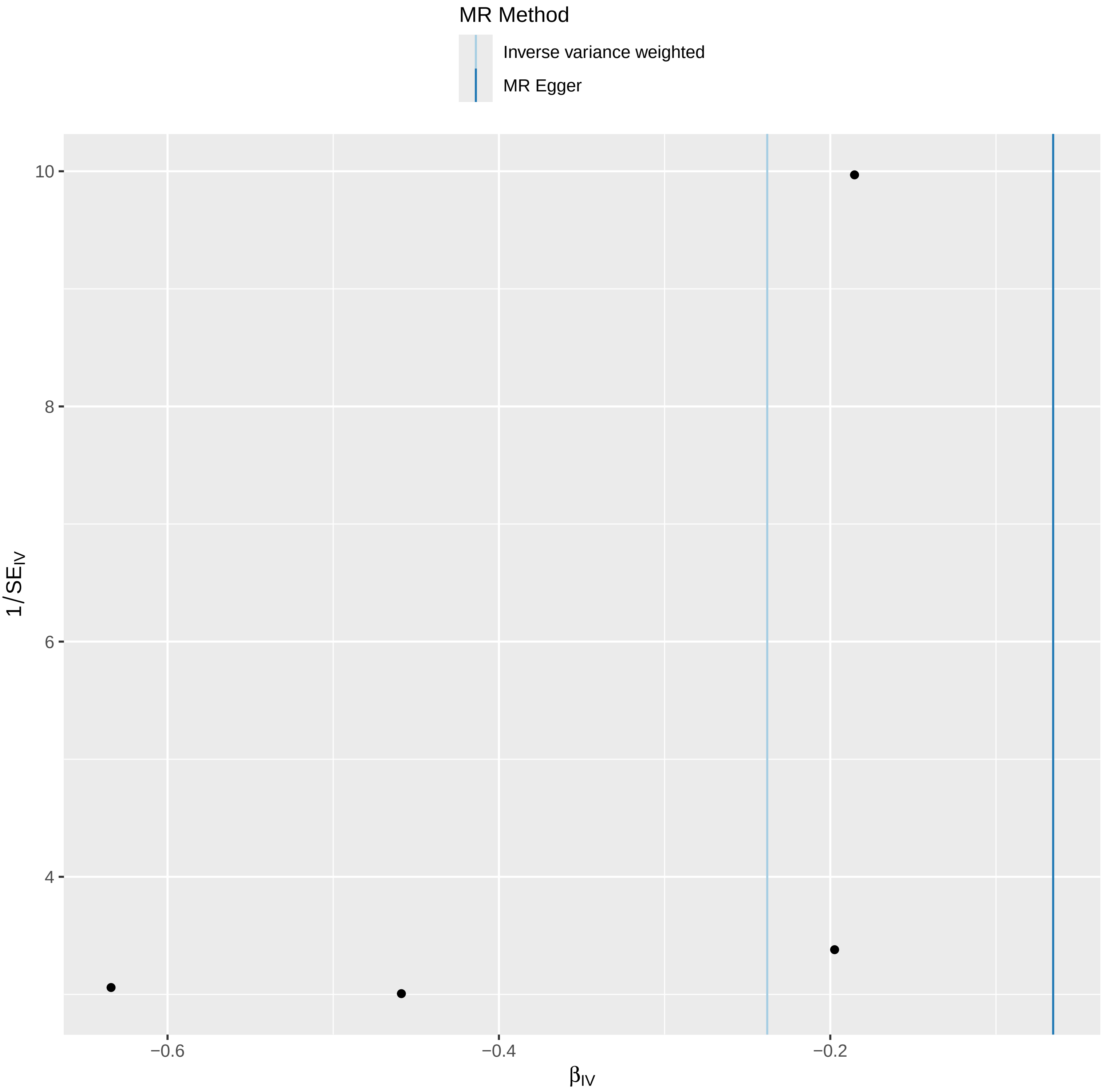
MR Method
Inverse variance weighted
MR Egger
10
8
6
4
1/SE$_{IV}$
−0.6
−0.4
−0.2
β$_{IV}$

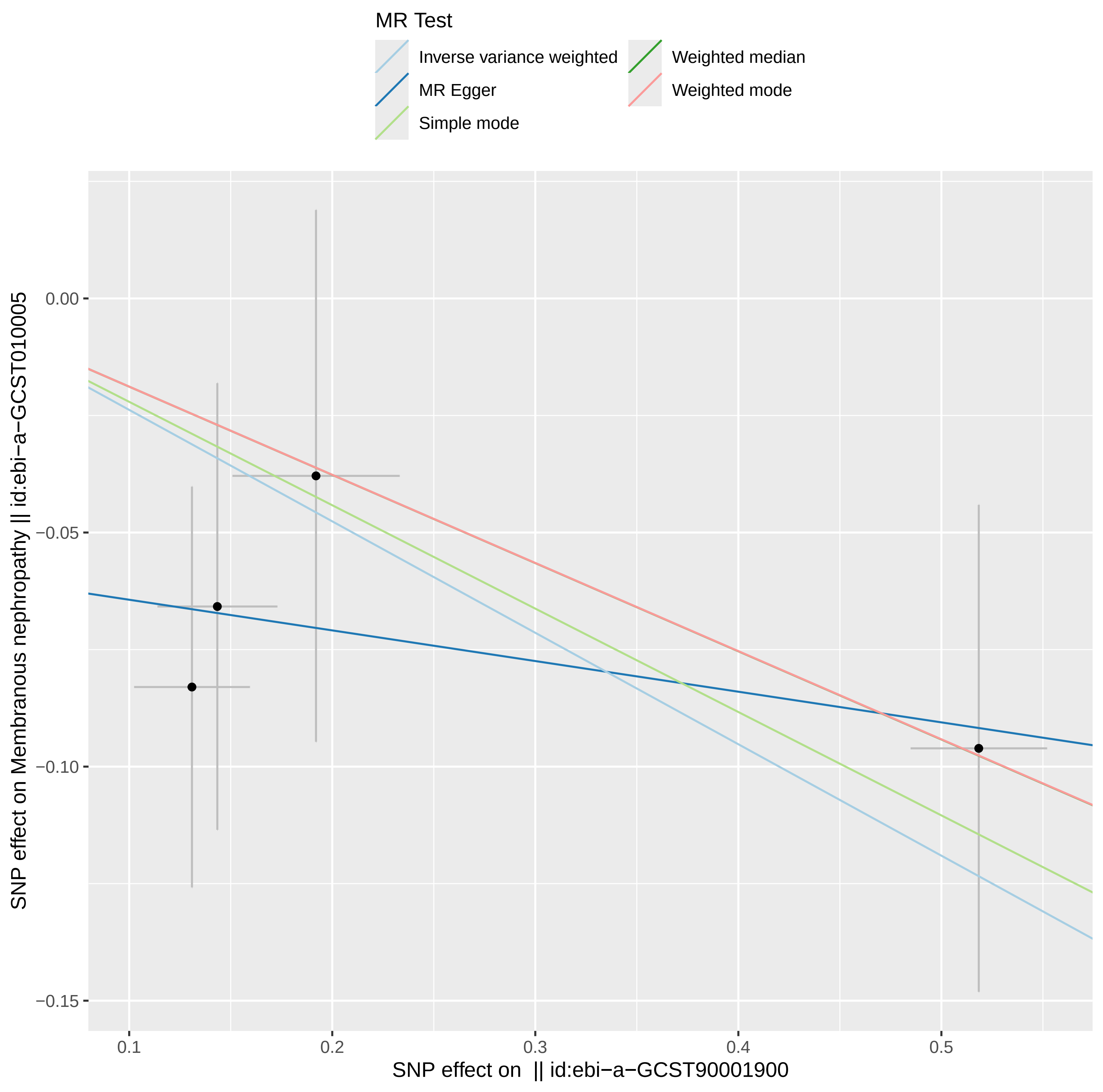

MR Test
Inverse variance weighted
MR Egger
Simple mode
Weighted median
Weighted mode
0.00
−0.05
−0.10
−0.15
0.1
0.2
0.3
0.4
0.5
SNP effect on Membranous nephropathy || id:ebi−a−GCST010005
SNP effect on || id:ebi−a−GCST90001900

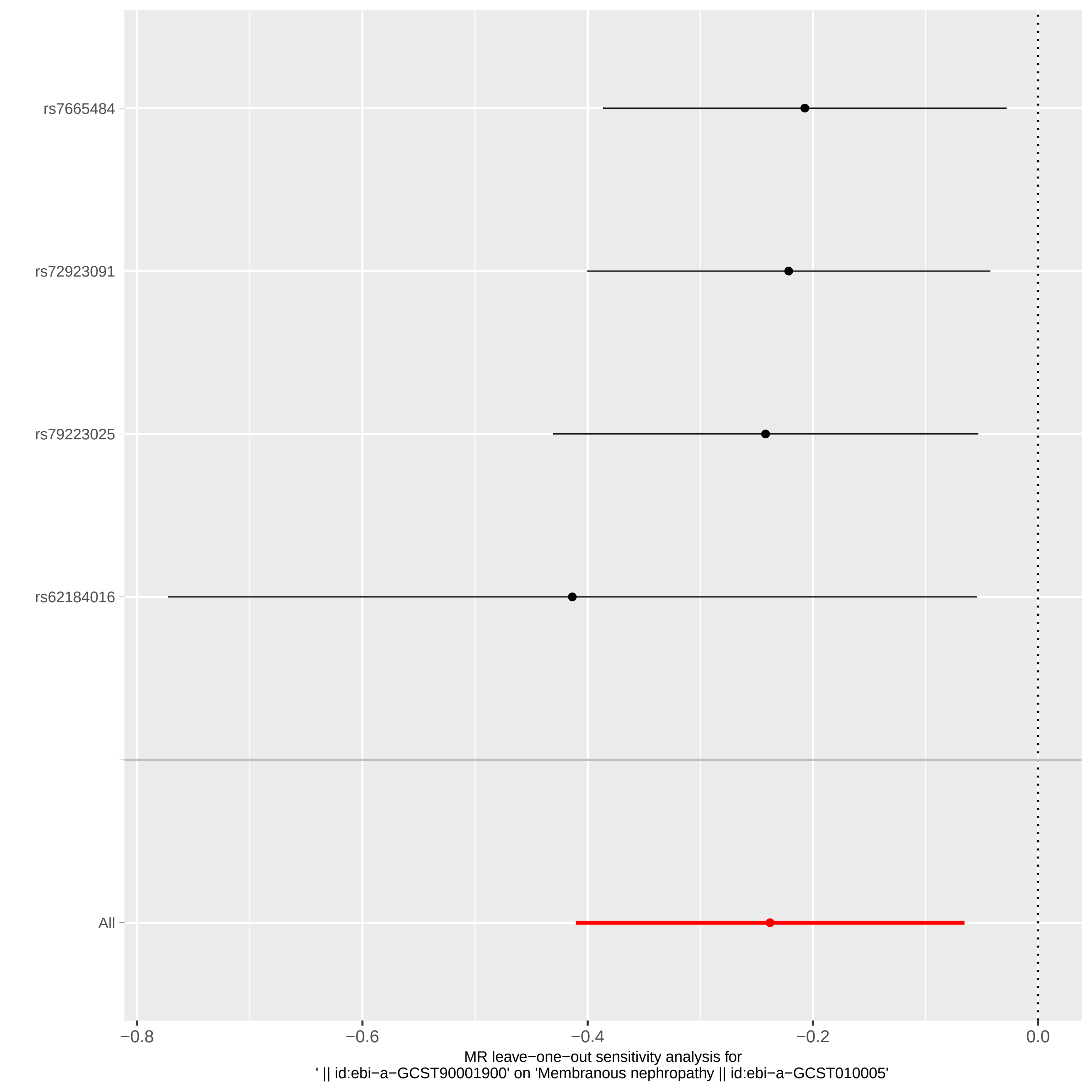
rs7665484
rs72923091
rs79223025
rs62184016
All
−0.8
−0.6
−0.4
−0.2
0.0
MR leave−one−out sensitivity analysis for
' || id:ebi−a−GCST90001900' on 'Membranous nephropathy || id:ebi−a−GCST010005'

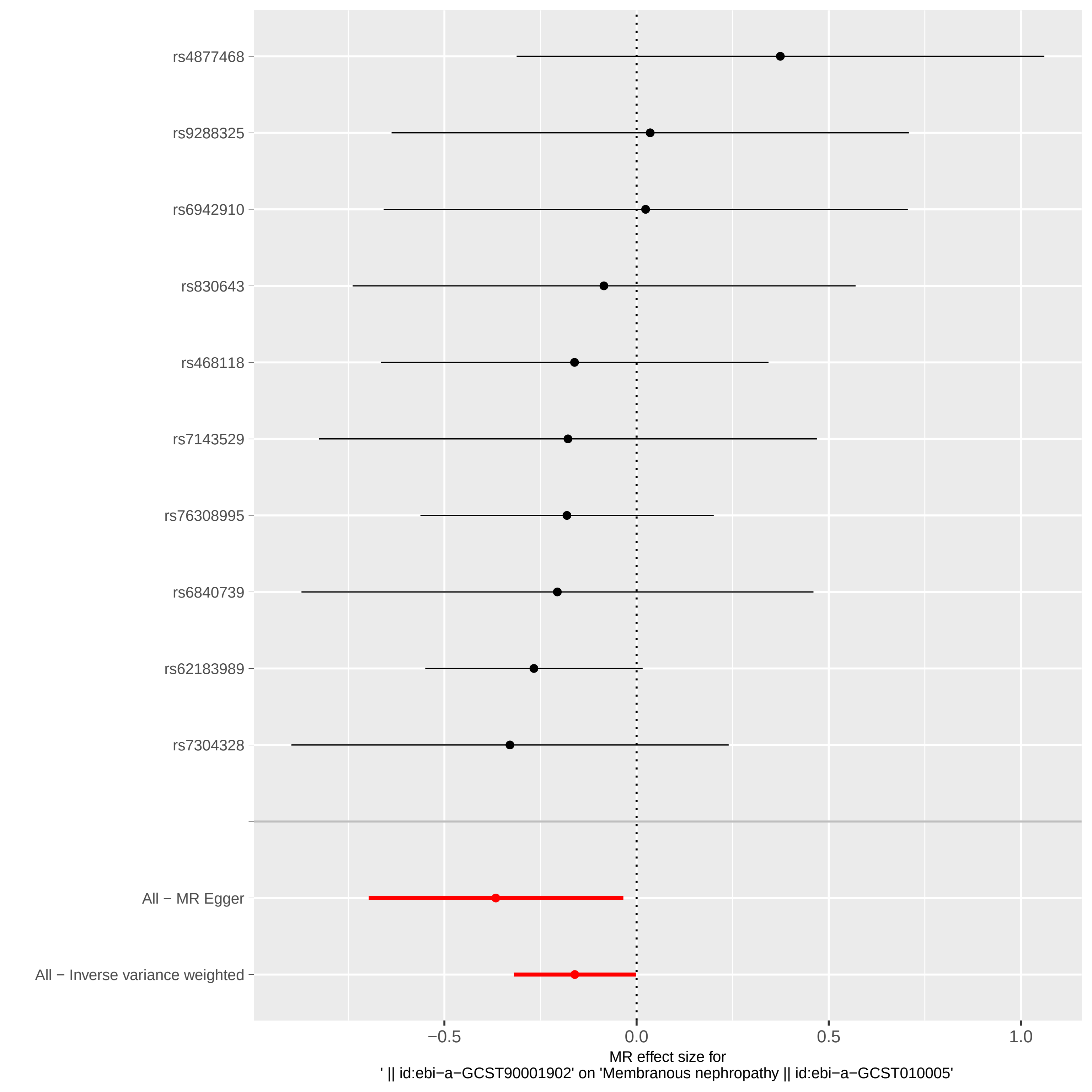
rs4877468
rs9288325
rs6942910
rs830643
rs468118
rs7143529
rs76308995
rs6840739
rs62183989
rs7304328
All − MR Egger
All − Inverse variance weighted
−0.5
0.0
0.5
1.0
MR effect size for
' || id:ebi−a−GCST90001902' on 'Membranous nephropathy || id:ebi−a−GCST010005'

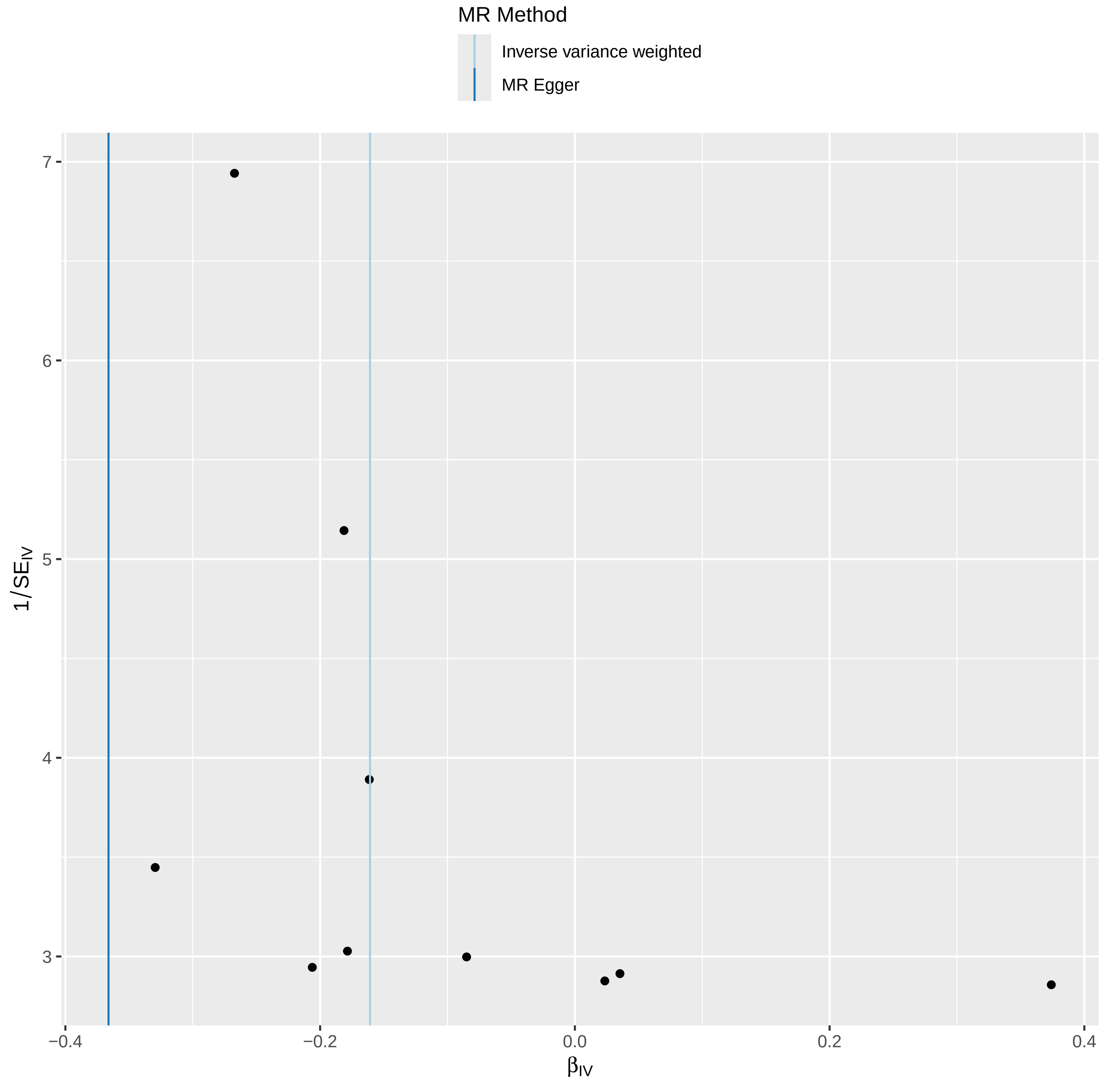

MR Method
Inverse variance weighted
MR Egger
7
6
5
4
3
1/SE_IV
−0.4
−0.2
0.0
0.2
0.4
β_IV

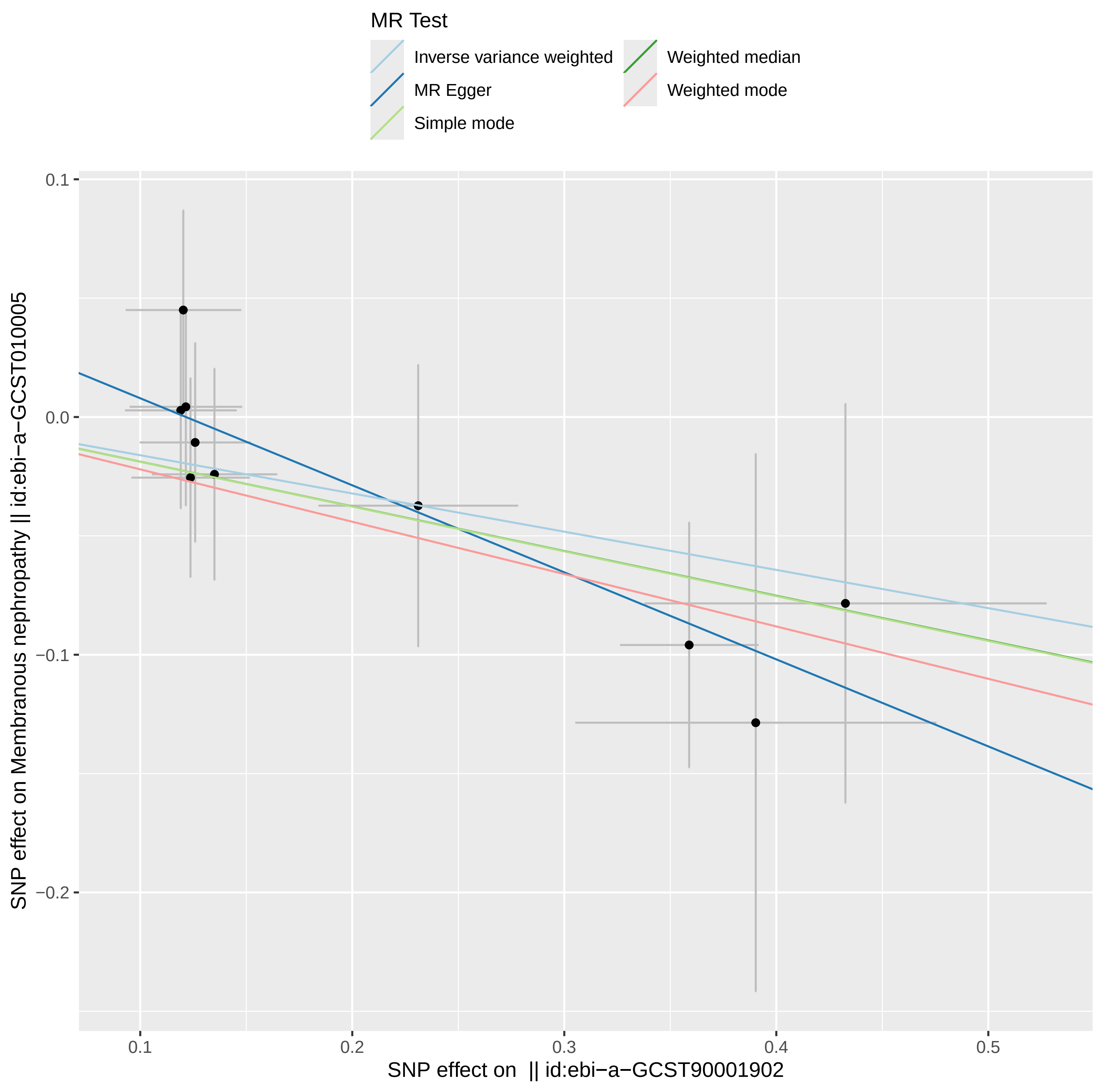
MR Test
Inverse variance weighted
MR Egger
Simple mode
Weighted median
Weighted mode
0.1
0.0
−0.1
−0.2
0.1
0.2
0.3
0.4
0.5
SNP effect on Membranous nephropathy || id:ebi−a−GCST010005
SNP effect on || id:ebi−a−GCST90001902

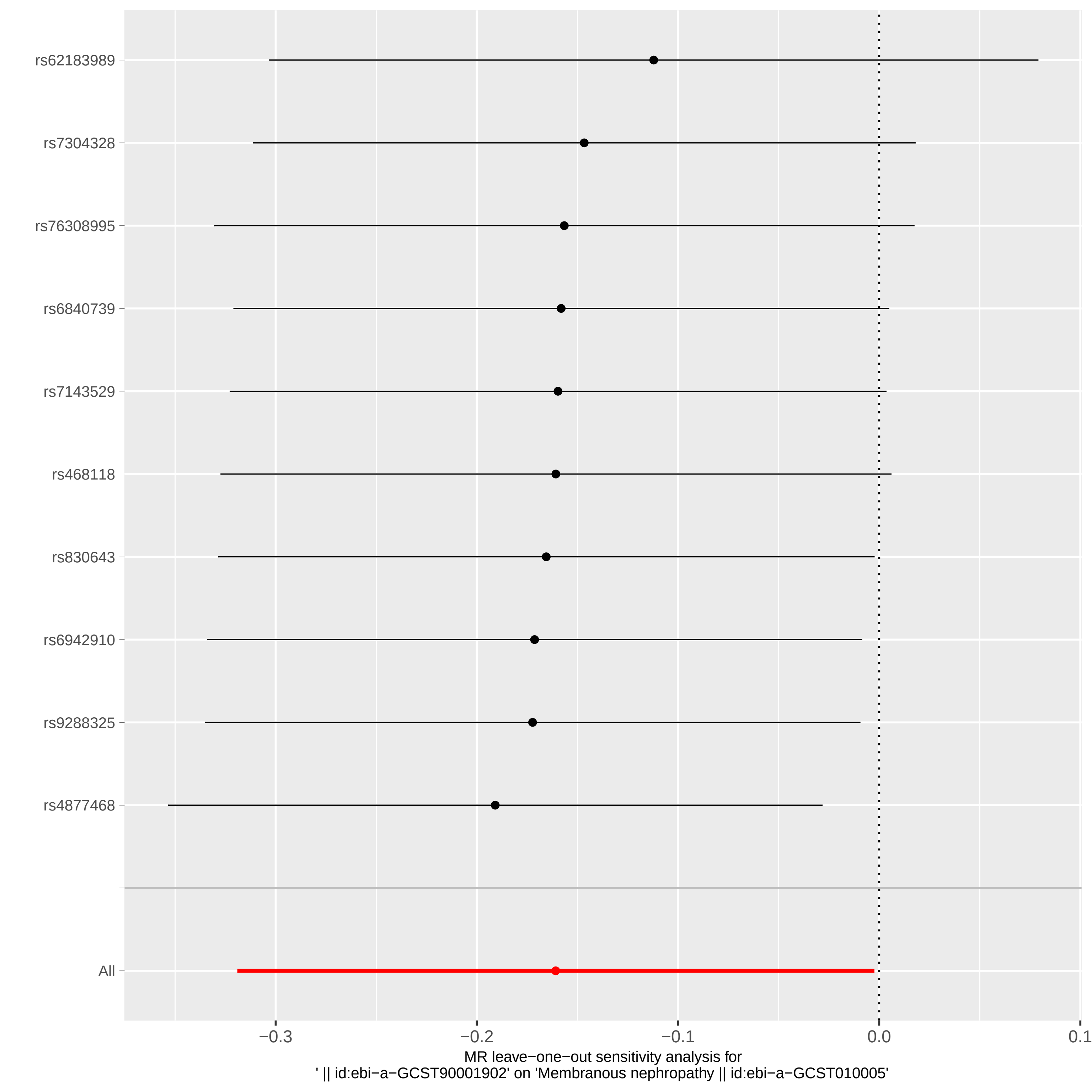

rs62183989
rs7304328
rs76308995
rs6840739
rs7143529
rs468118
rs830643
rs6942910
rs9288325
rs4877468
All
−0.3
−0.2
−0.1
0.0
0.1
MR leave−one−out sensitivity analysis for
' || id:ebi−a−GCST90001902' on 'Membranous nephropathy || id:ebi−a−GCST010005'

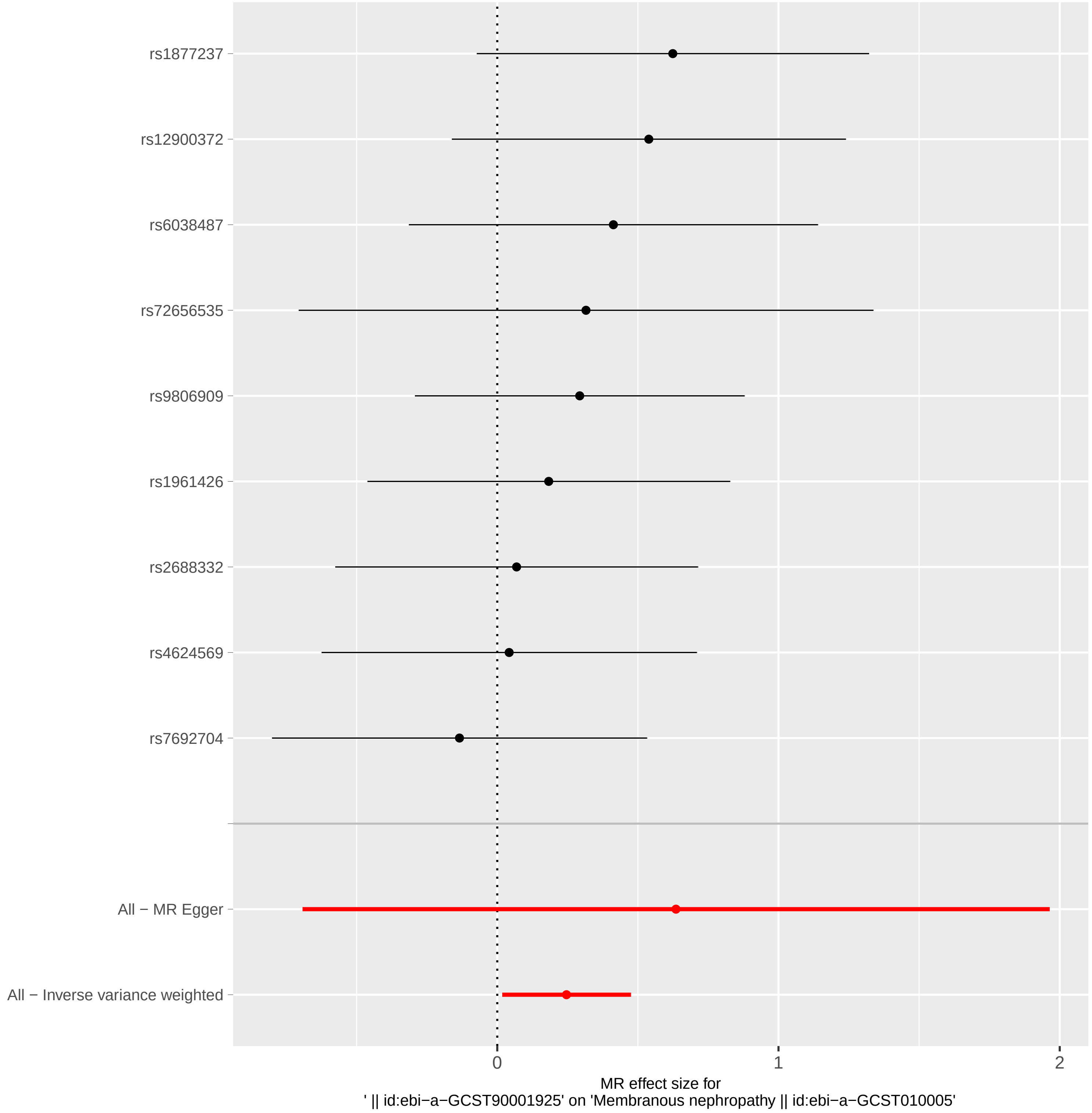
rs1877237
rs12900372
rs6038487
rs72656535
rs9806909
rs1961426
rs2688332
rs4624569
rs7692704
All − MR Egger
All − Inverse variance weighted
0
1
2
MR effect size for
' || id:ebi−a−GCST90001925' on 'Membranous nephropathy || id:ebi−a−GCST010005'

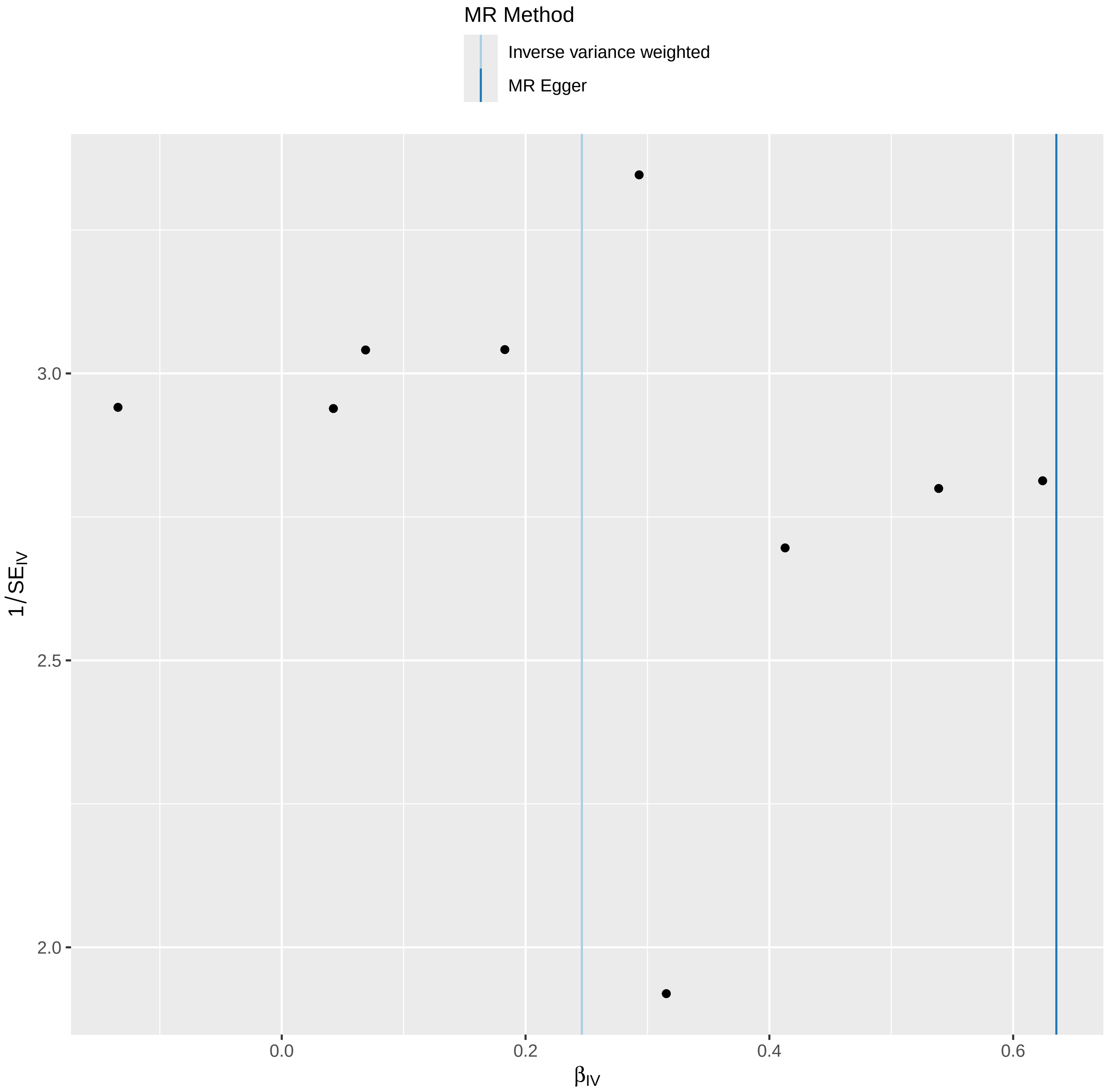
MR Method
Inverse variance weighted
MR Egger
3.0
2.5
2.0
0.0
0.2
0.4
0.6
1/SE_IV
β_IV

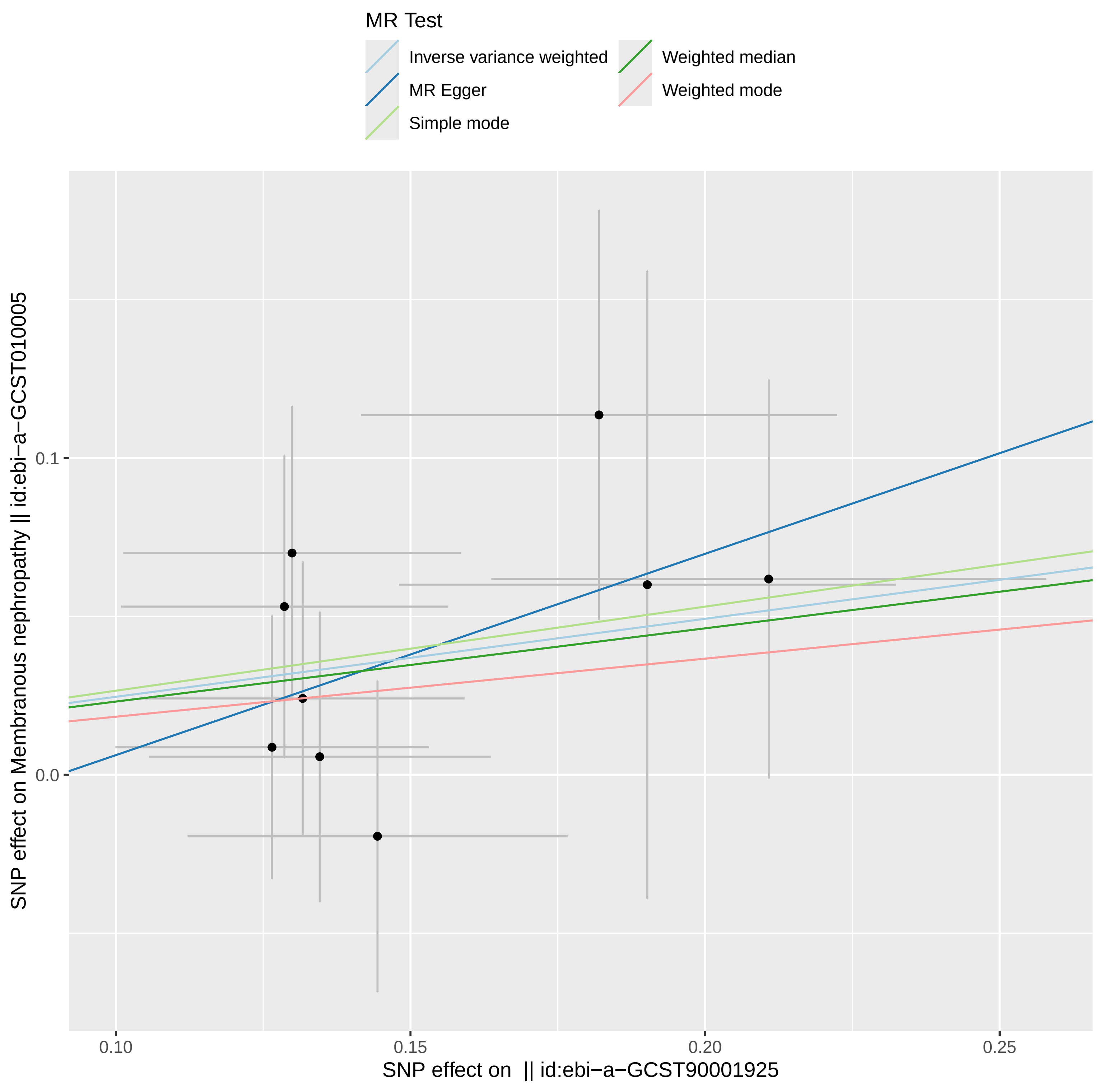

MR Test
Inverse variance weighted
MR Egger
Simple mode
Weighted median
Weighted mode
SNP effect on Membranous nephropathy || id:ebi−a−GCST010005
0.1
0.0
0.10
0.15
0.20
0.25
SNP effect on || id:ebi−a−GCST90001925

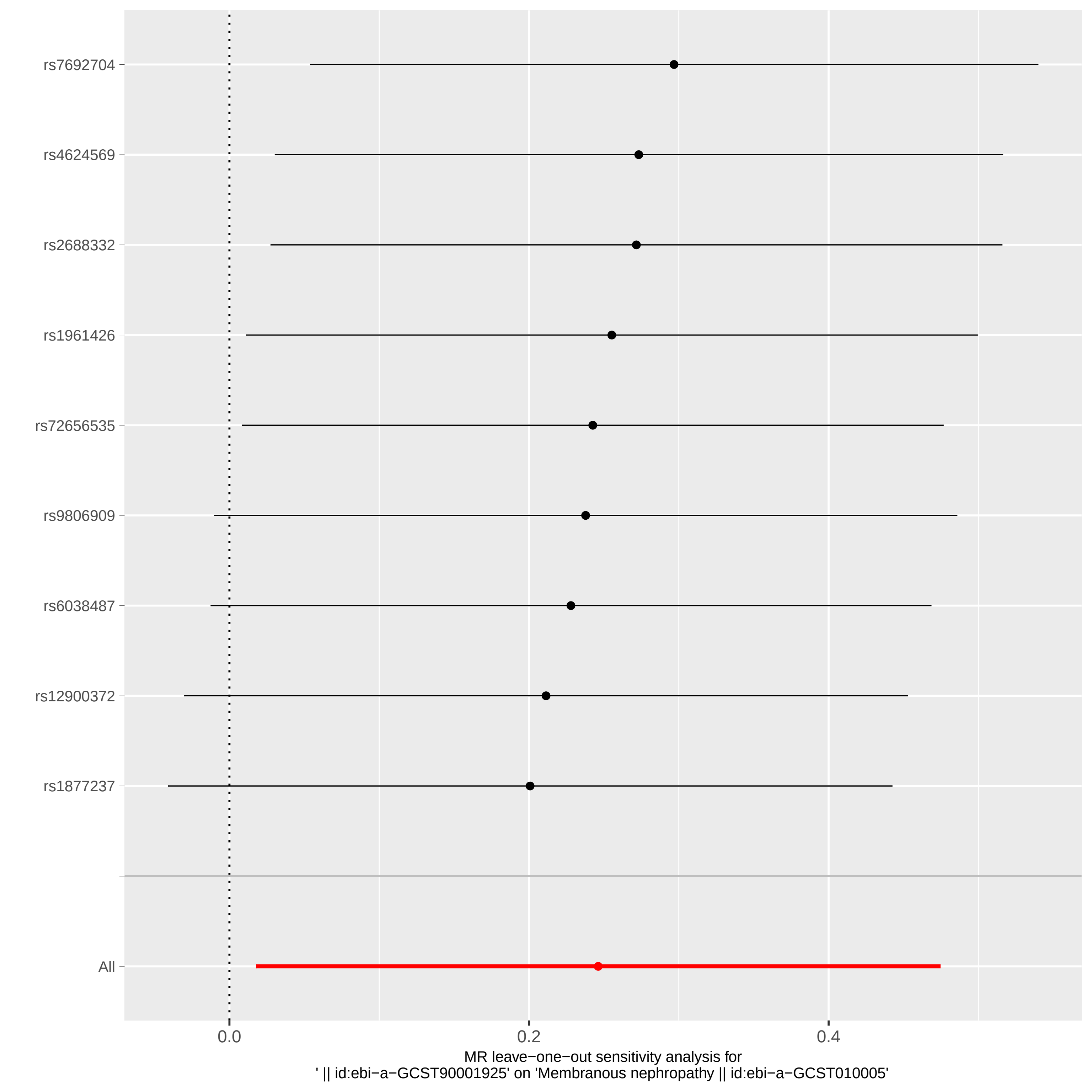
rs7692704
rs4624569
rs2688332
rs1961426
rs72656535
rs9806909
rs6038487
rs12900372
rs1877237
All
0.0
0.2
0.4
MR leave−one−out sensitivity analysis for
' || id:ebi−a−GCST90001925' on 'Membranous nephropathy || id:ebi−a−GCST010005'

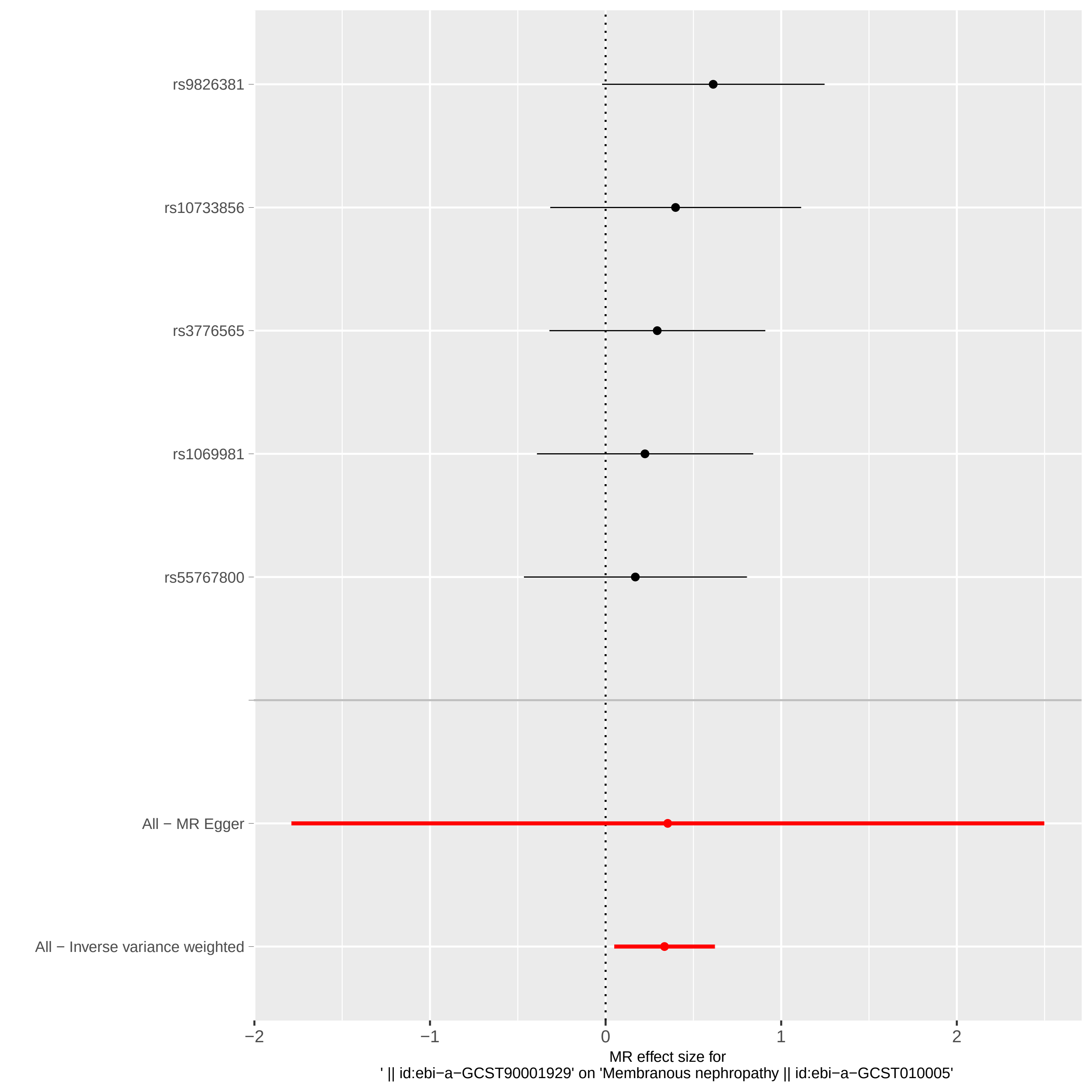
rs9826381
rs10733856
rs3776565
rs1069981
rs55767800
All − MR Egger
All − Inverse variance weighted
−2
−1
0
1
2
MR effect size for
' || id:ebi−a−GCST90001929' on 'Membranous nephropathy || id:ebi−a−GCST010005'

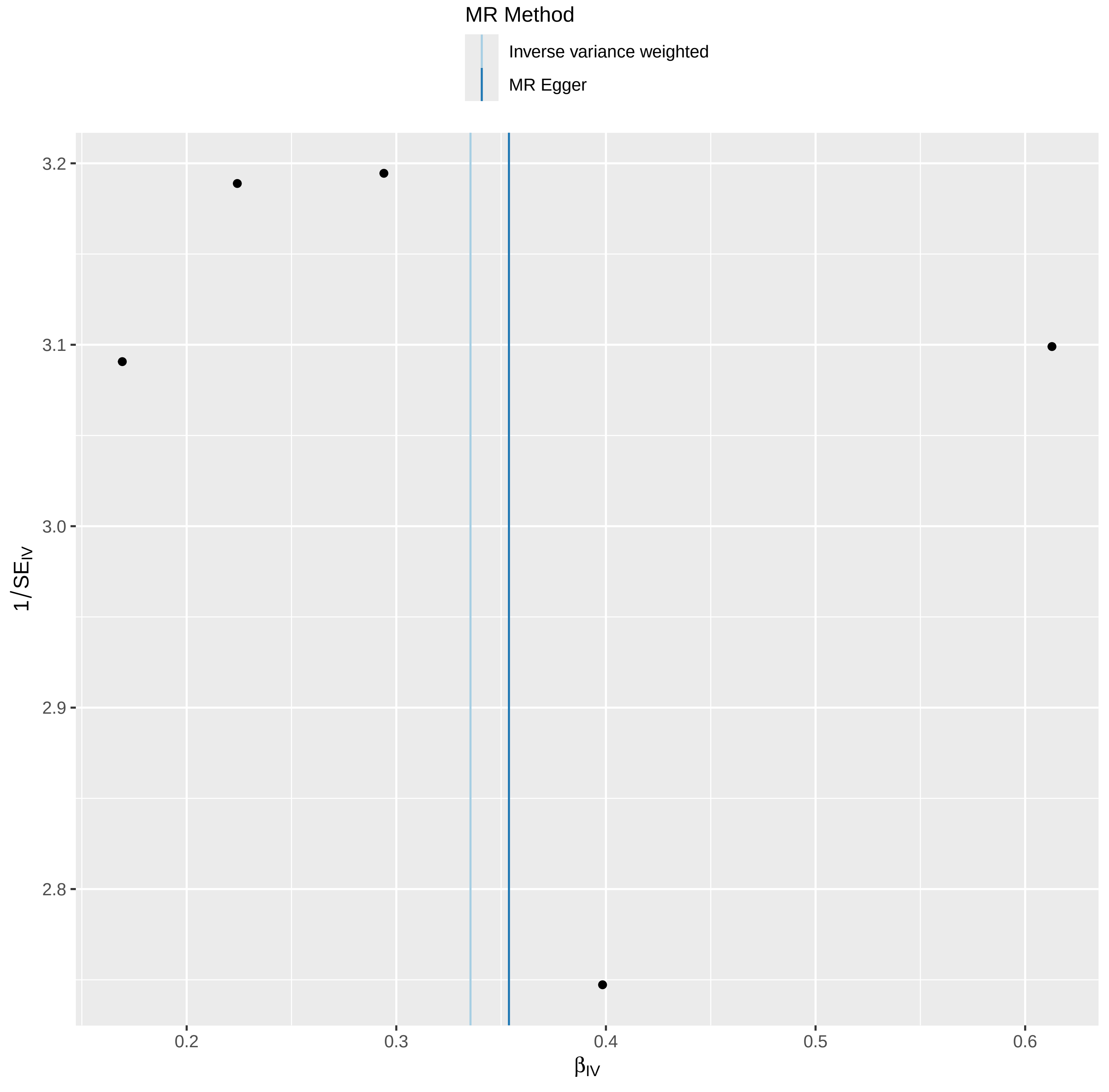

MR Method
Inverse variance weighted
MR Egger
3.2
3.1
3.0
2.9
2.8
1/SE_IV
0.2
0.3
0.4
0.5
0.6
β_IV

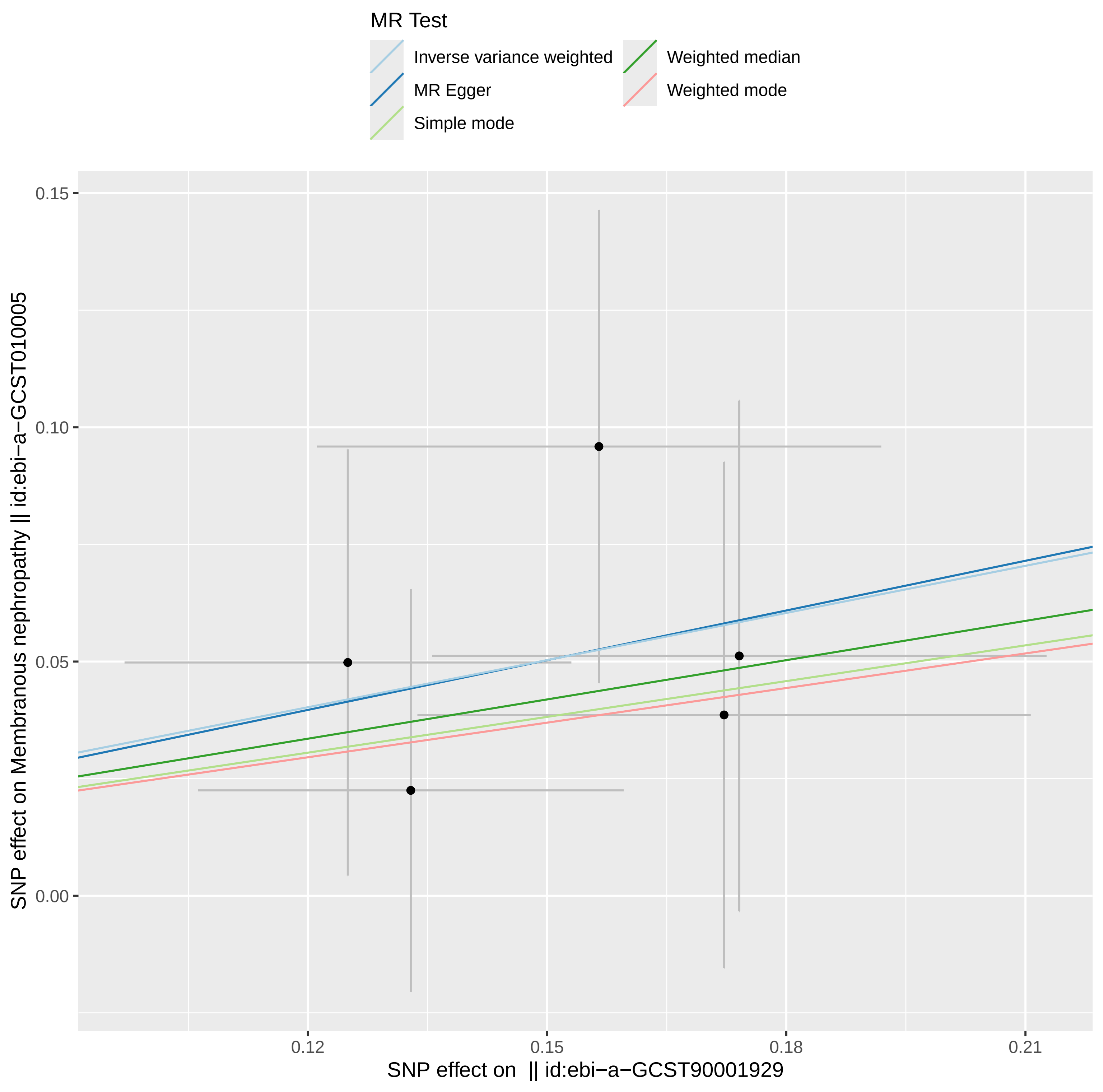
MR Test
Inverse variance weighted
MR Egger
Simple mode
Weighted median
Weighted mode
0.15
0.10
0.05
0.00
0.12
0.15
0.18
0.21
SNP effect on Membranous nephropathy || id:ebi−a−GCST010005
SNP effect on || id:ebi−a−GCST90001929

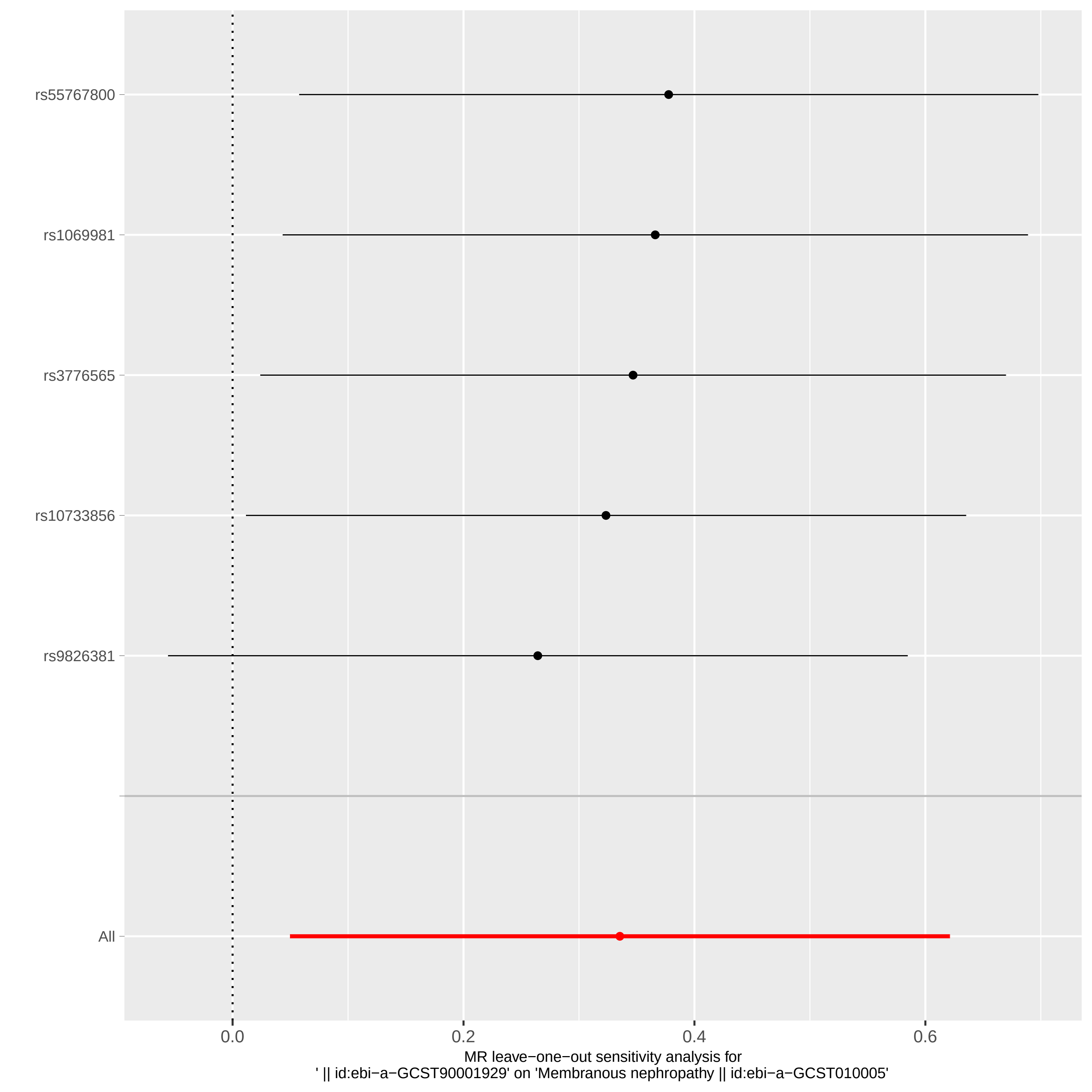

rs55767800
rs1069981
rs3776565
rs10733856
rs9826381
All
0.0
0.2
0.4
0.6
MR leave−one−out sensitivity analysis for
' || id:ebi−a−GCST90001929' on 'Membranous nephropathy || id:ebi−a−GCST010005'

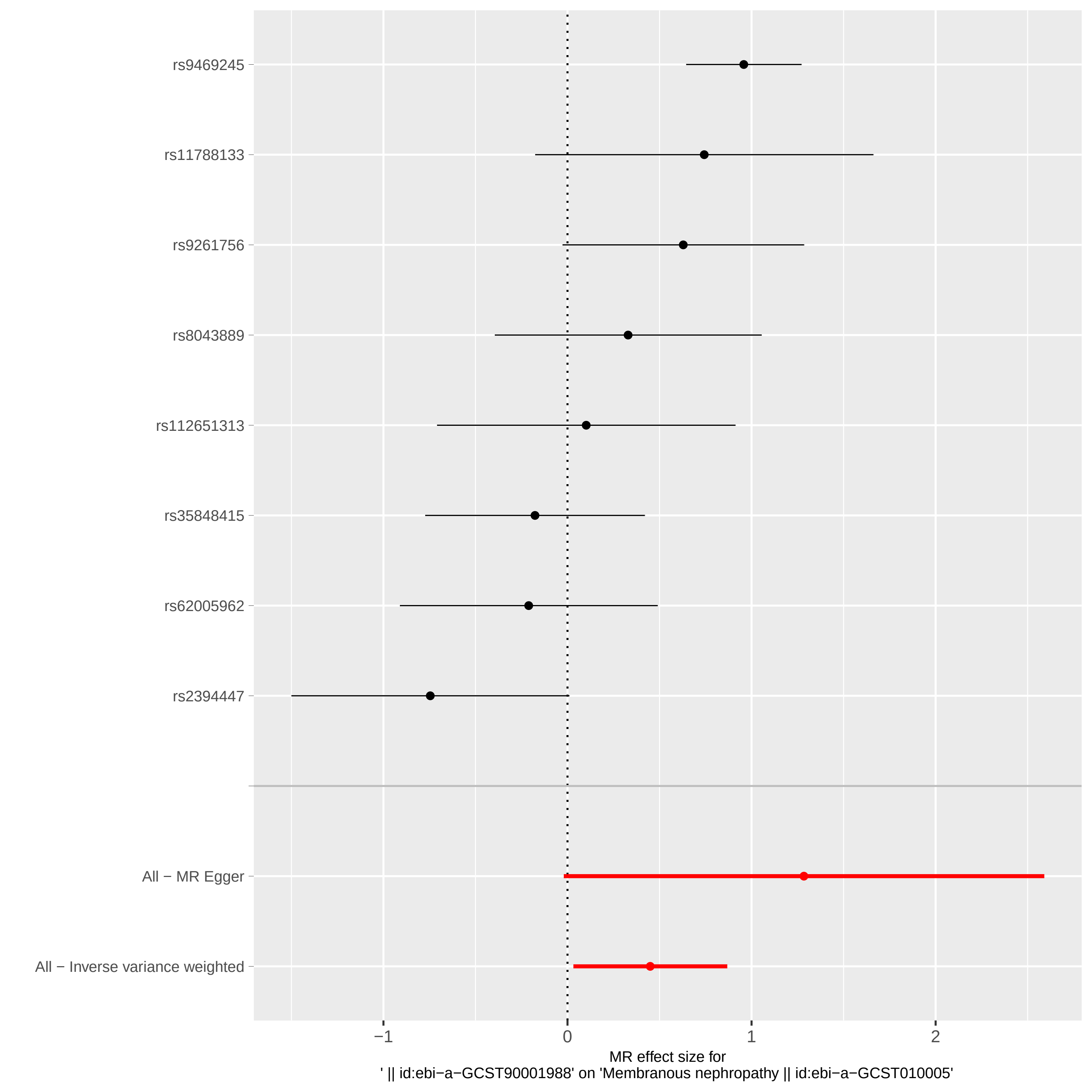
rs9469245
rs11788133
rs9261756
rs8043889
rs112651313
rs35848415
rs62005962
rs2394447
All − MR Egger
All − Inverse variance weighted
−1
0
1
2
MR effect size for
' || id:ebi−a−GCST90001988' on 'Membranous nephropathy || id:ebi−a−GCST010005'

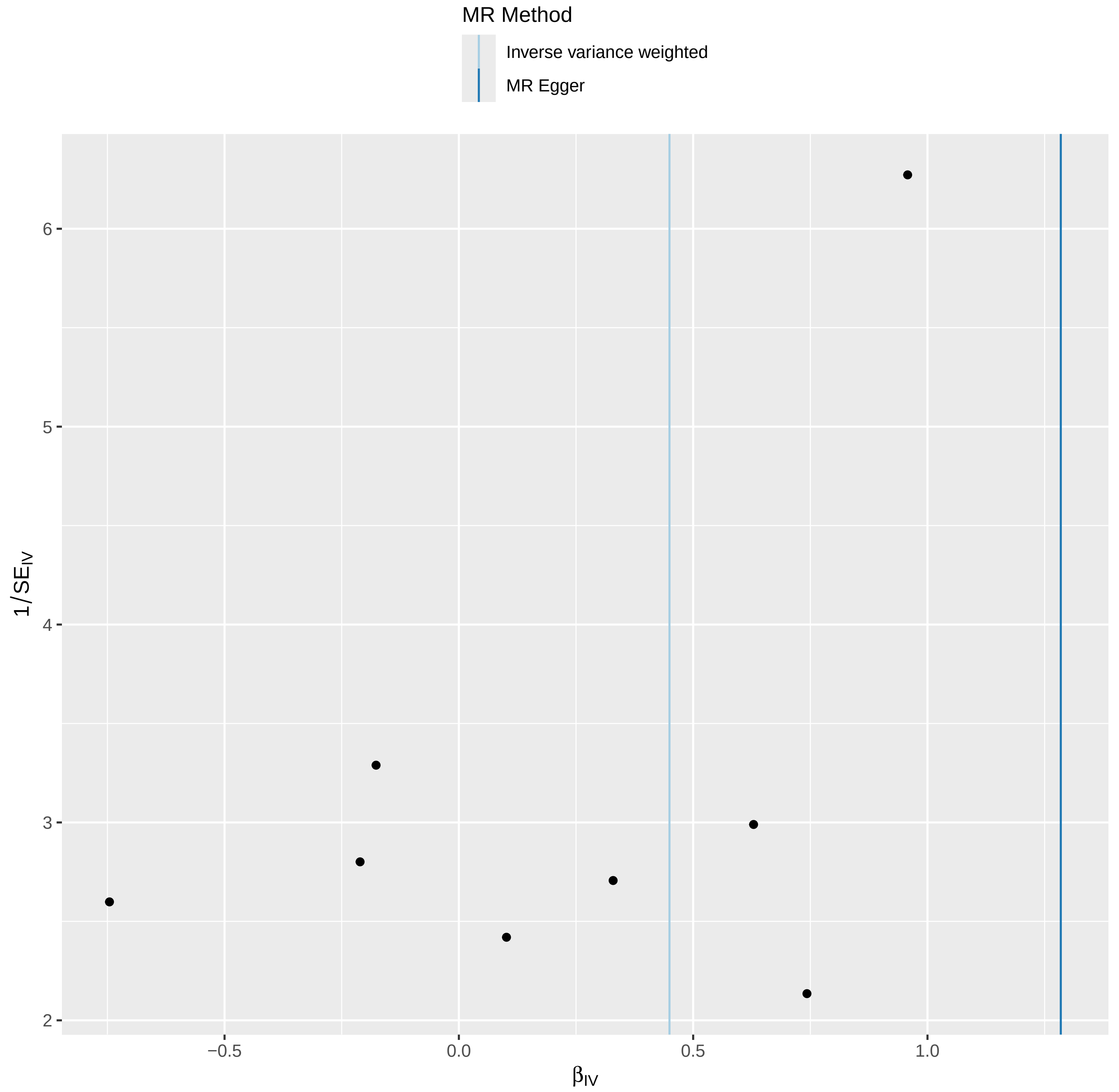

MR Method
Inverse variance weighted
MR Egger
1/SE_IV
6
5
4
3
2
−0.5
0.0
0.5
1.0
β_IV

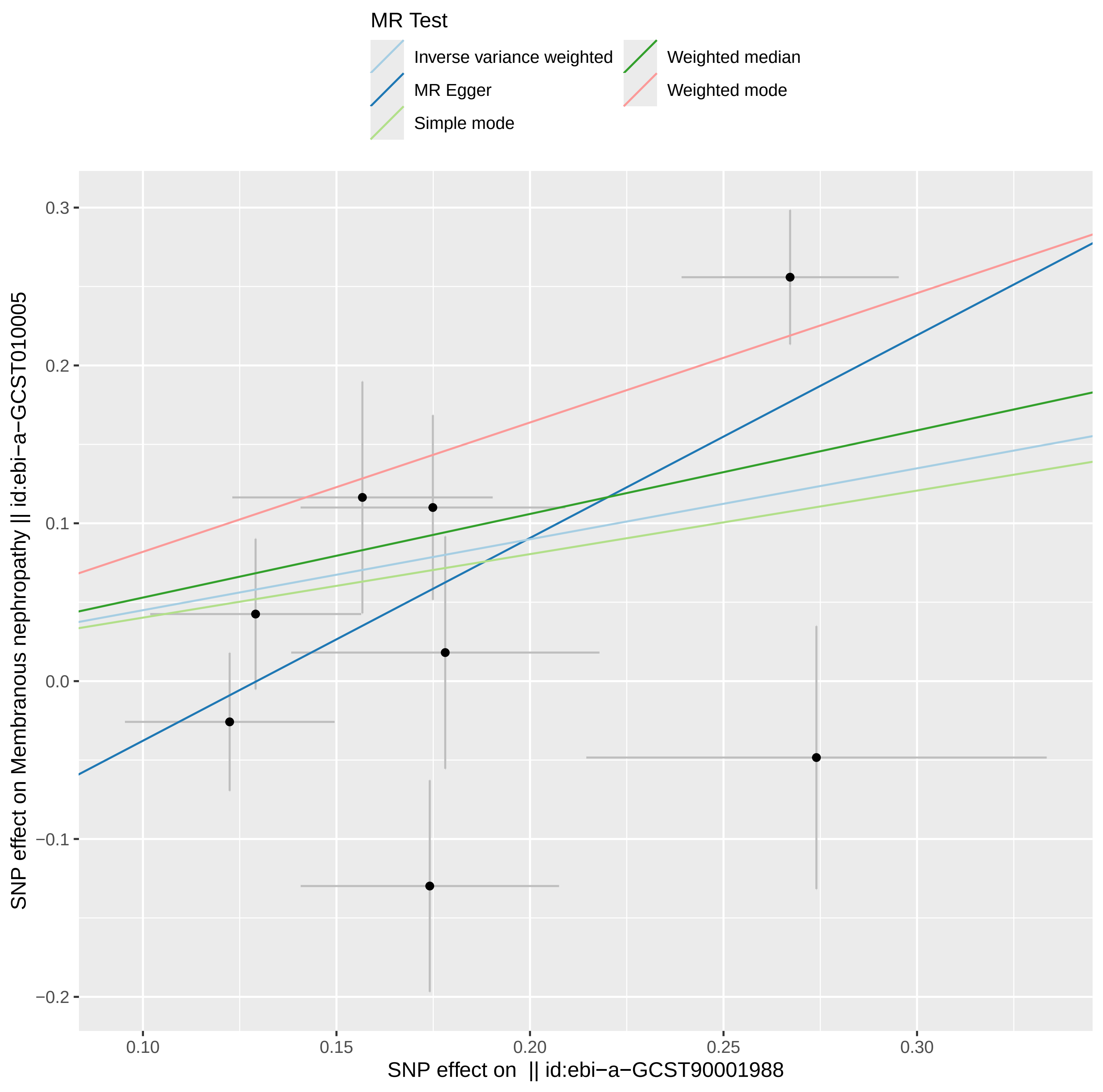
MR Test
Inverse variance weighted
MR Egger
Simple mode
Weighted median
Weighted mode
0.3
0.2
0.1
0.0
−0.1
−0.2
0.10
0.15
0.20
0.25
0.30
SNP effect on Membranous nephropathy || id:ebi−a−GCST010005
SNP effect on || id:ebi−a−GCST90001988

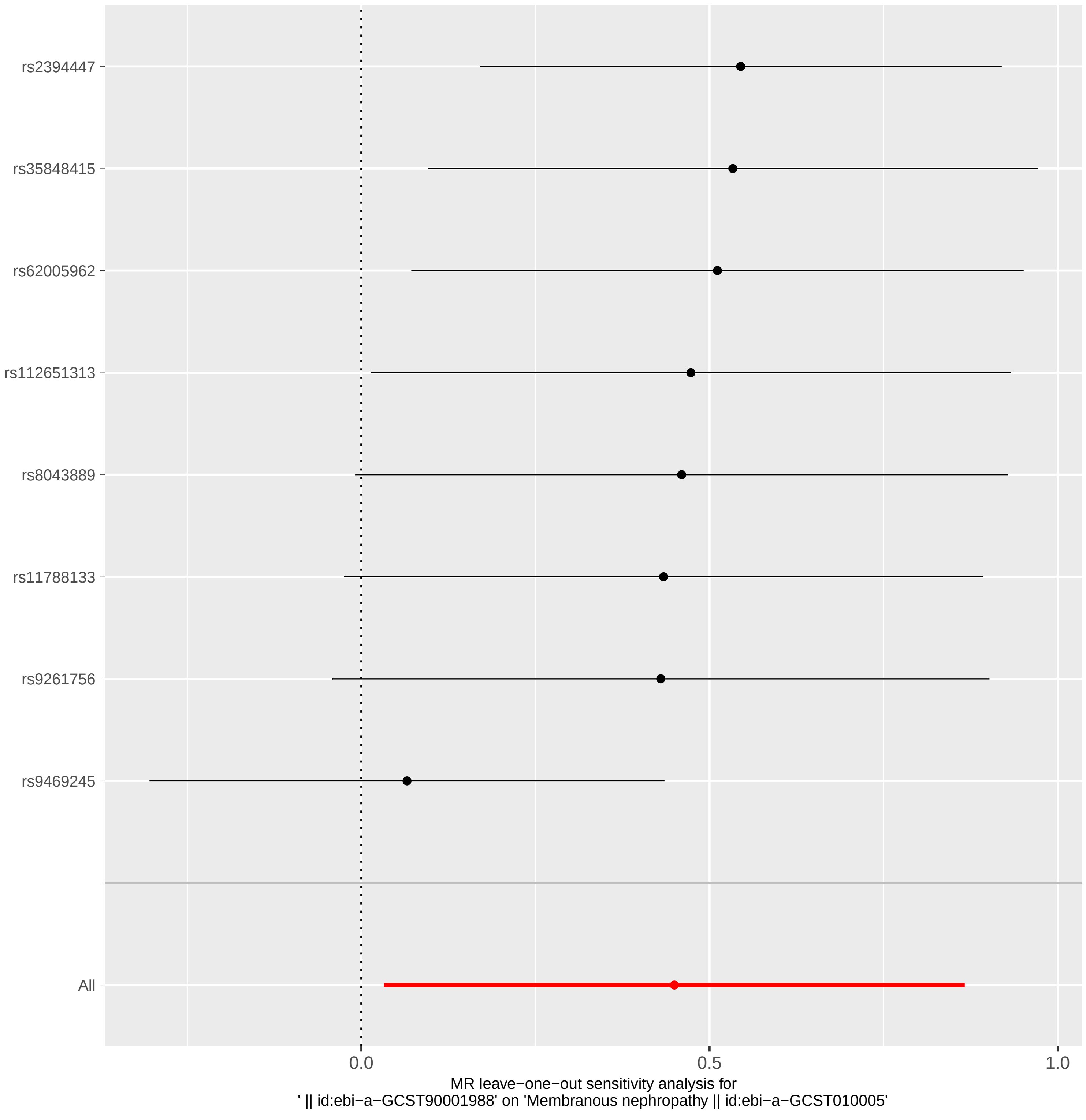
rs2394447
rs35848415
rs62005962
rs112651313
rs8043889
rs11788133
rs9261756
rs9469245
All
0.0
0.5
1.0
MR leave−one−out sensitivity analysis for
' || id:ebi−a−GCST90001988' on 'Membranous nephropathy || id:ebi−a−GCST010005'

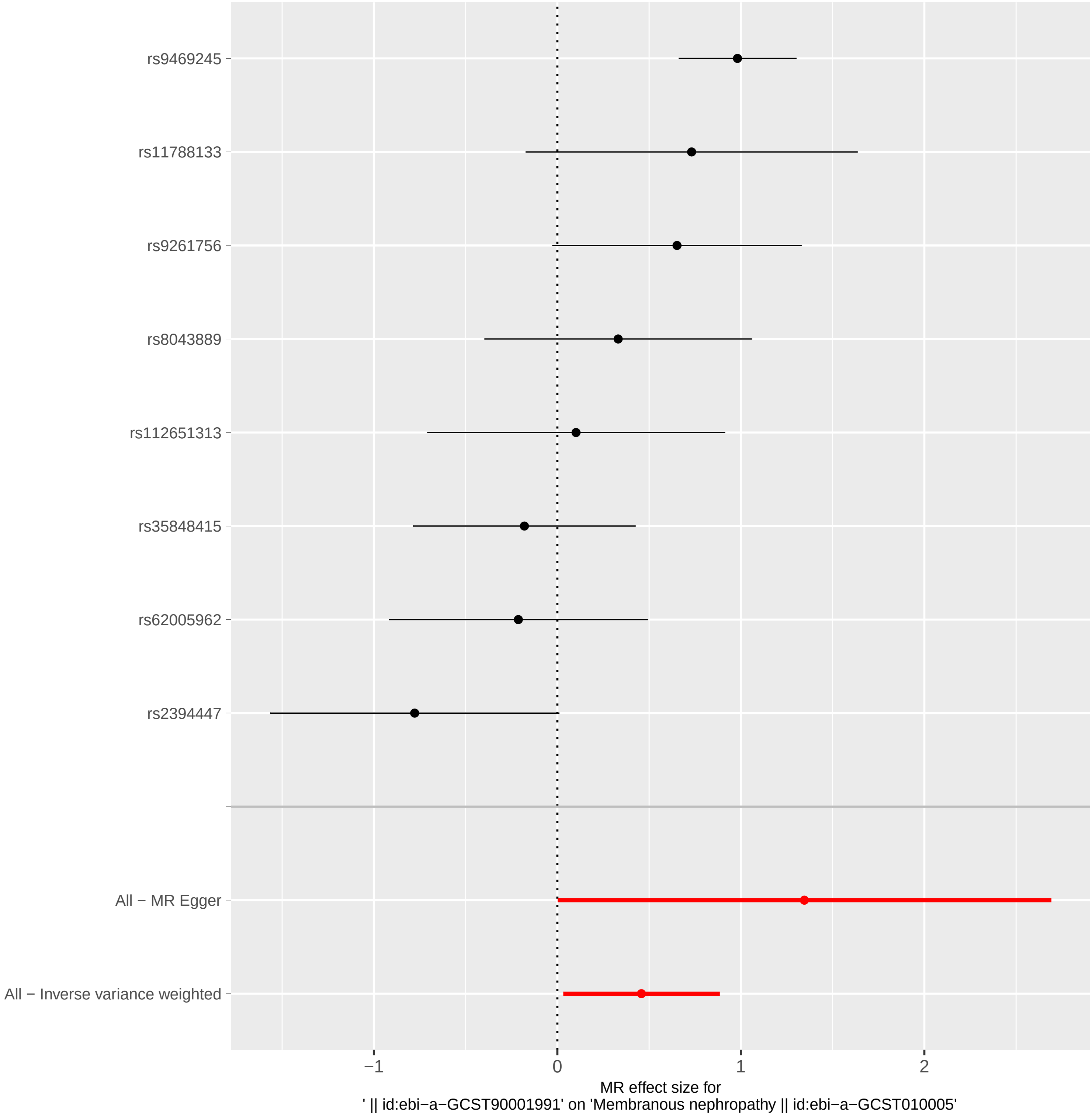

rs9469245
rs11788133
rs9261756
rs8043889
rs112651313
rs35848415
rs62005962
rs2394447
All − MR Egger
All − Inverse variance weighted
−1
0
1
2
MR effect size for
' || id:ebi−a−GCST90001991' on 'Membranous nephropathy || id:ebi−a−GCST010005'

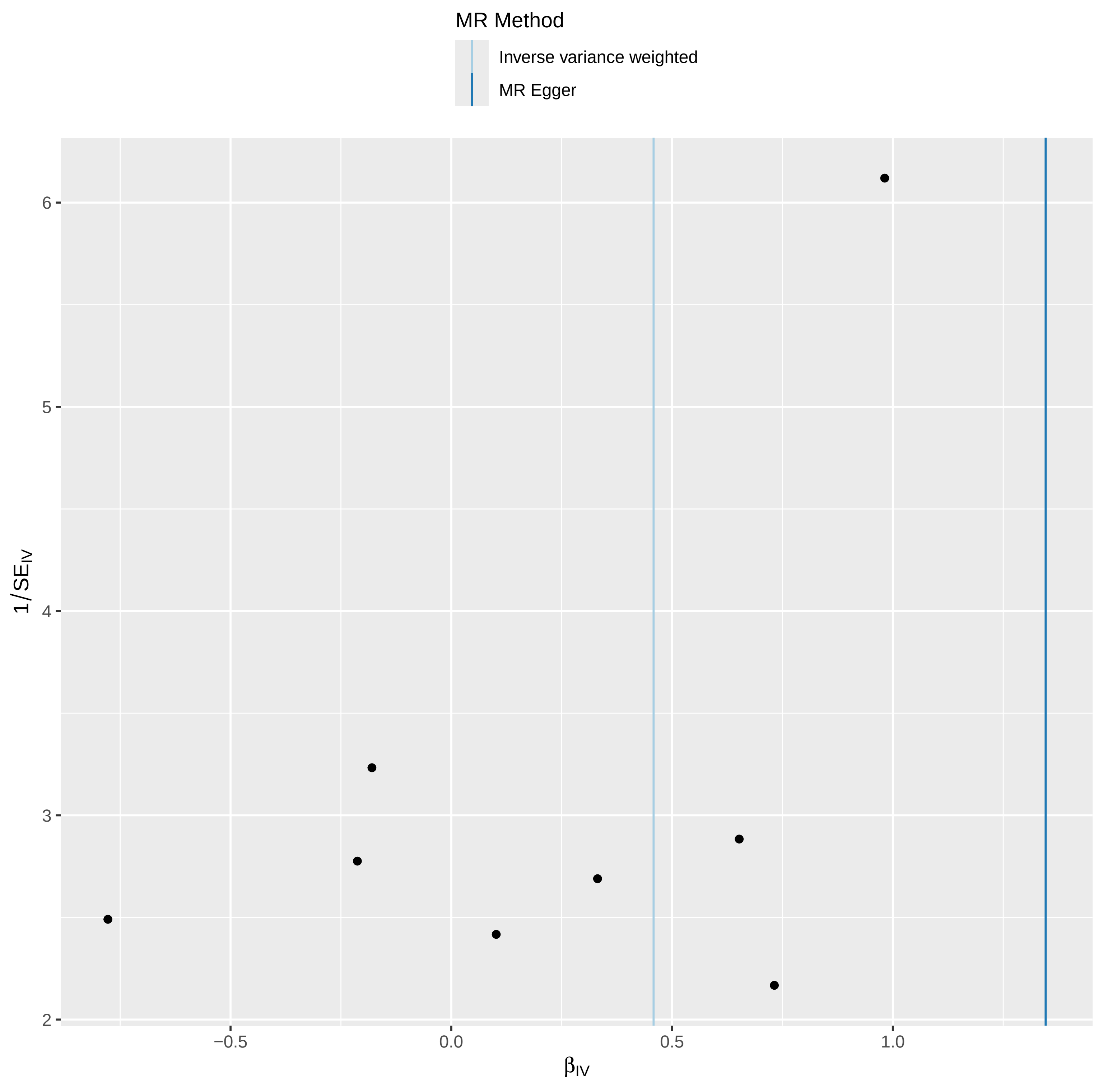

MR Method
Inverse variance weighted
MR Egger
6
5
4
3
2
−0.5
0.0
0.5
1.0
$1/SE_{IV}$
β_{IV}

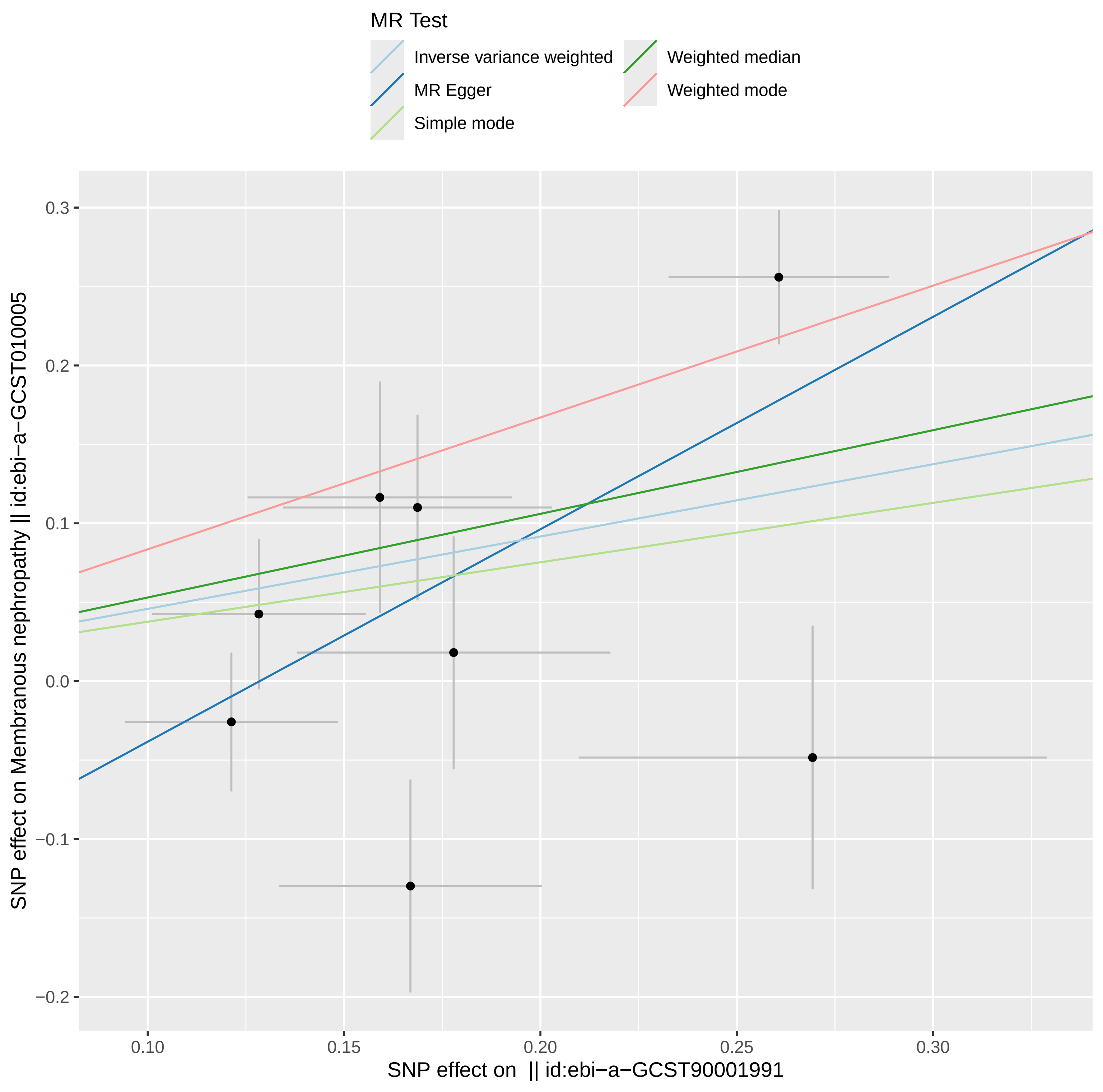
MR Test
Inverse variance weighted
MR Egger
Simple mode
Weighted median
Weighted mode
SNP effect on Membranous nephropathy || id:ebi−a−GCST010005
0.3
0.2
0.1
0.0
−0.1
−0.2
0.10
0.15
0.20
0.25
0.30
SNP effect on || id:ebi−a−GCST90001991

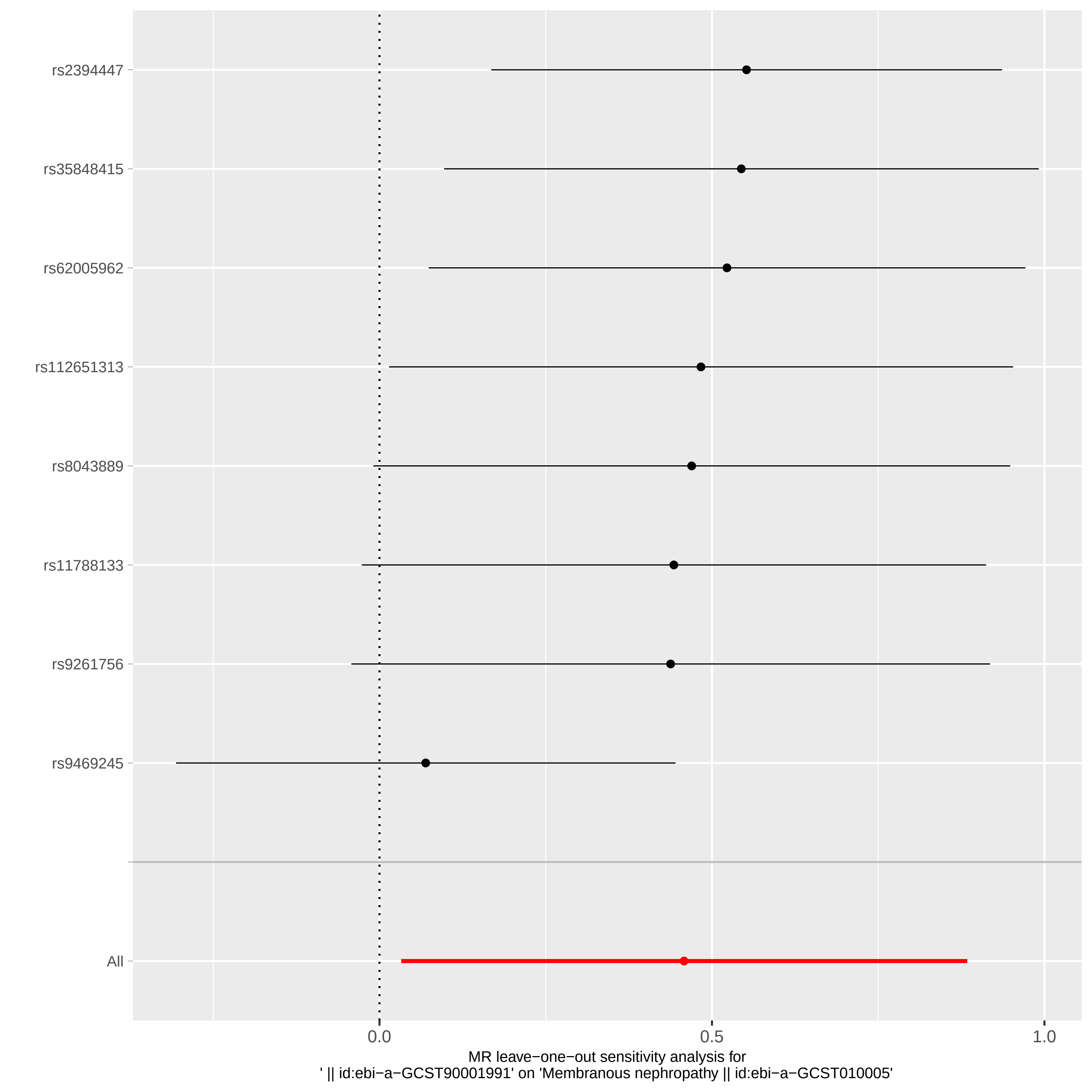

rs2394447
rs35848415
rs62005962
rs112651313
rs8043889
rs11788133
rs9261756
rs9469245
All
0.0
0.5
1.0
MR leave−one−out sensitivity analysis for
' || id:ebi−a−GCST90001991' on 'Membranous nephropathy || id:ebi−a−GCST010005'

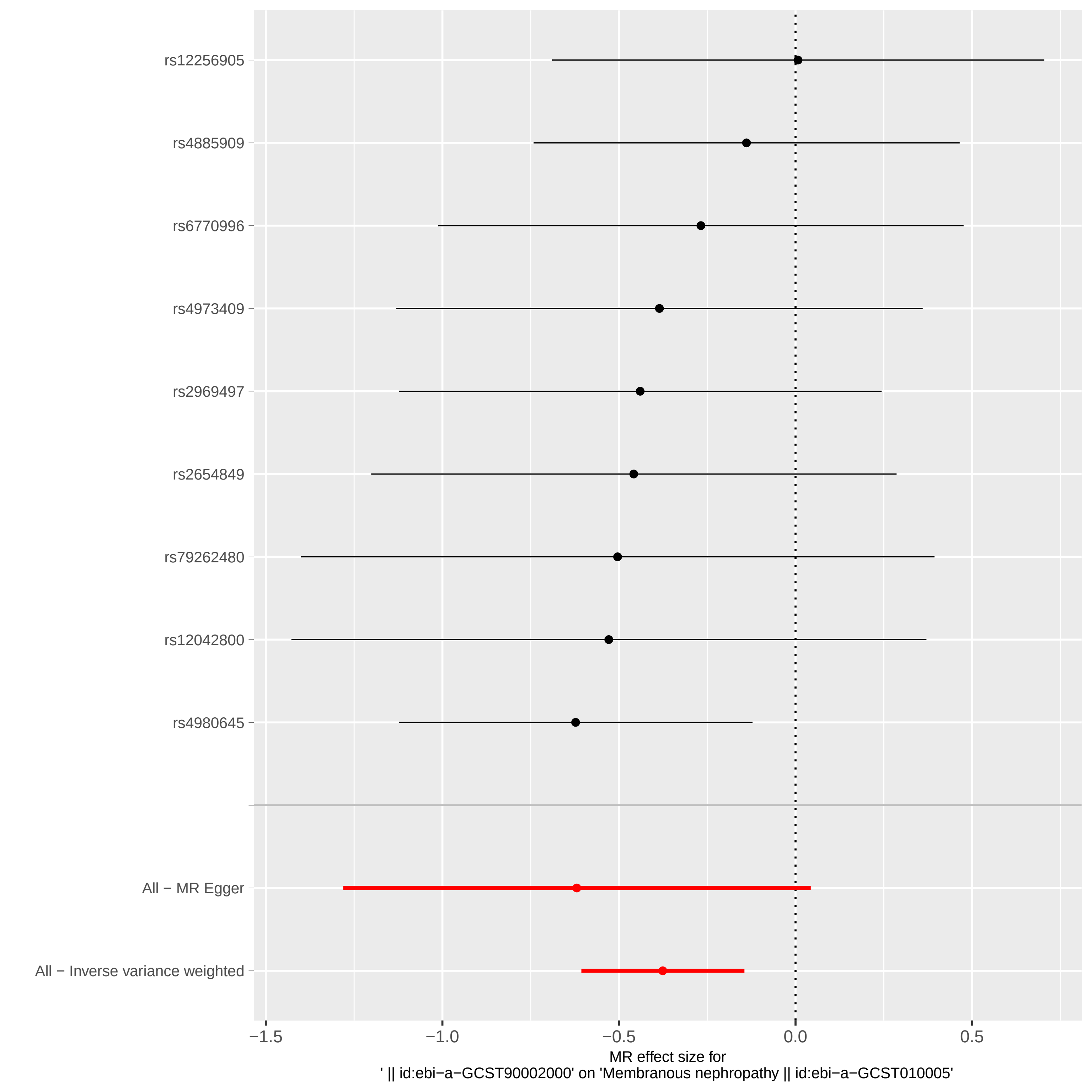
rs12256905
rs4885909
rs6770996
rs4973409
rs2969497
rs2654849
rs79262480
rs12042800
rs4980645
All − MR Egger
All − Inverse variance weighted
−1.5
−1.0
−0.5
0.0
0.5
MR effect size for
' || id:ebi−a−GCST90002000' on 'Membranous nephropathy || id:ebi−a−GCST010005'

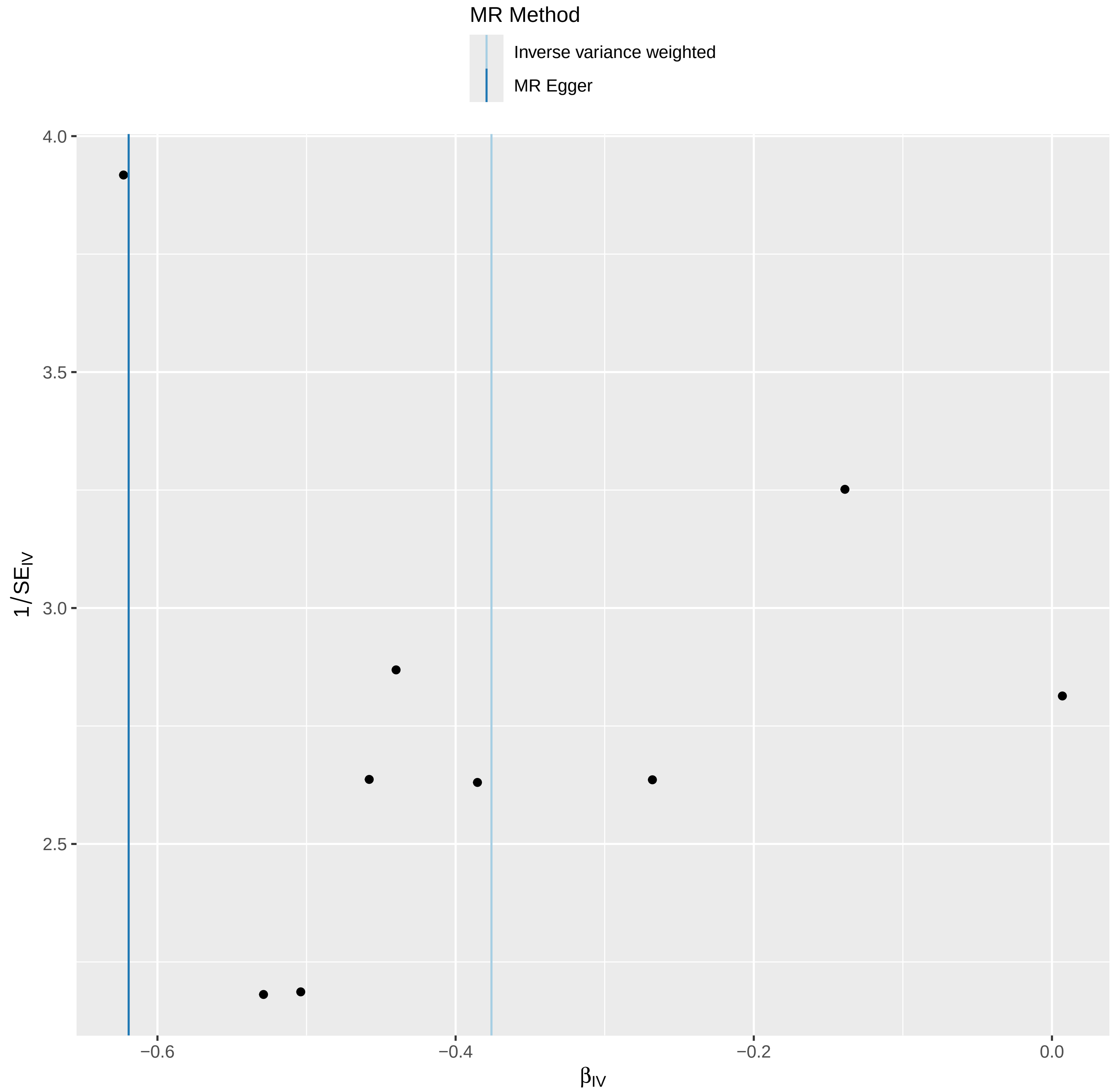

MR Method
Inverse variance weighted
MR Egger
4.0
3.5
3.0
2.5
1/SE_IV
−0.6
−0.4
−0.2
0.0
β_IV

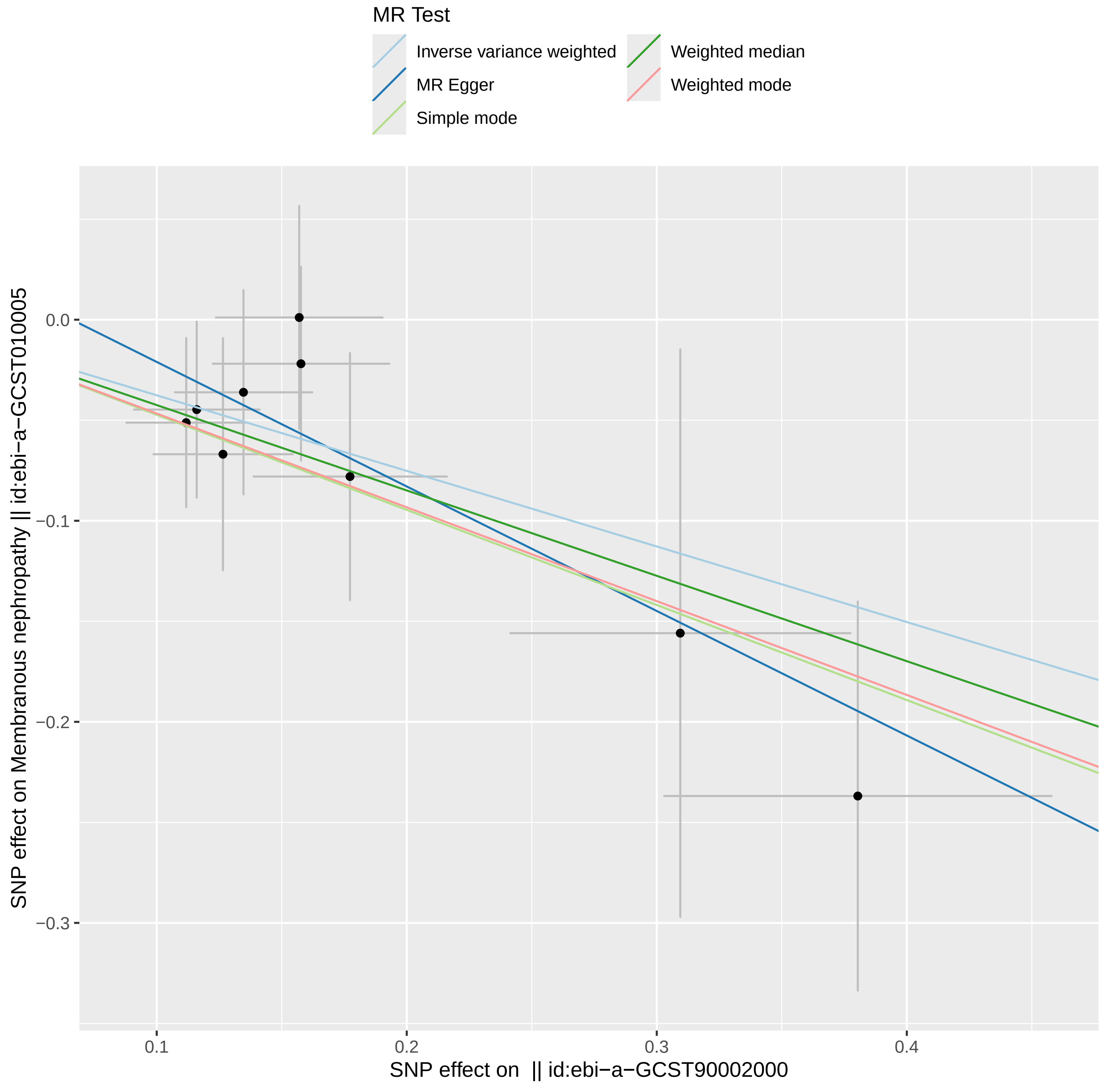
MR Test
Inverse variance weighted
MR Egger
Simple mode
Weighted median
Weighted mode
SNP effect on Membranous nephropathy || id:ebi−a−GCST010005
0.0
−0.1
−0.2
−0.3
0.1
0.2
0.3
0.4
SNP effect on || id:ebi−a−GCST90002000

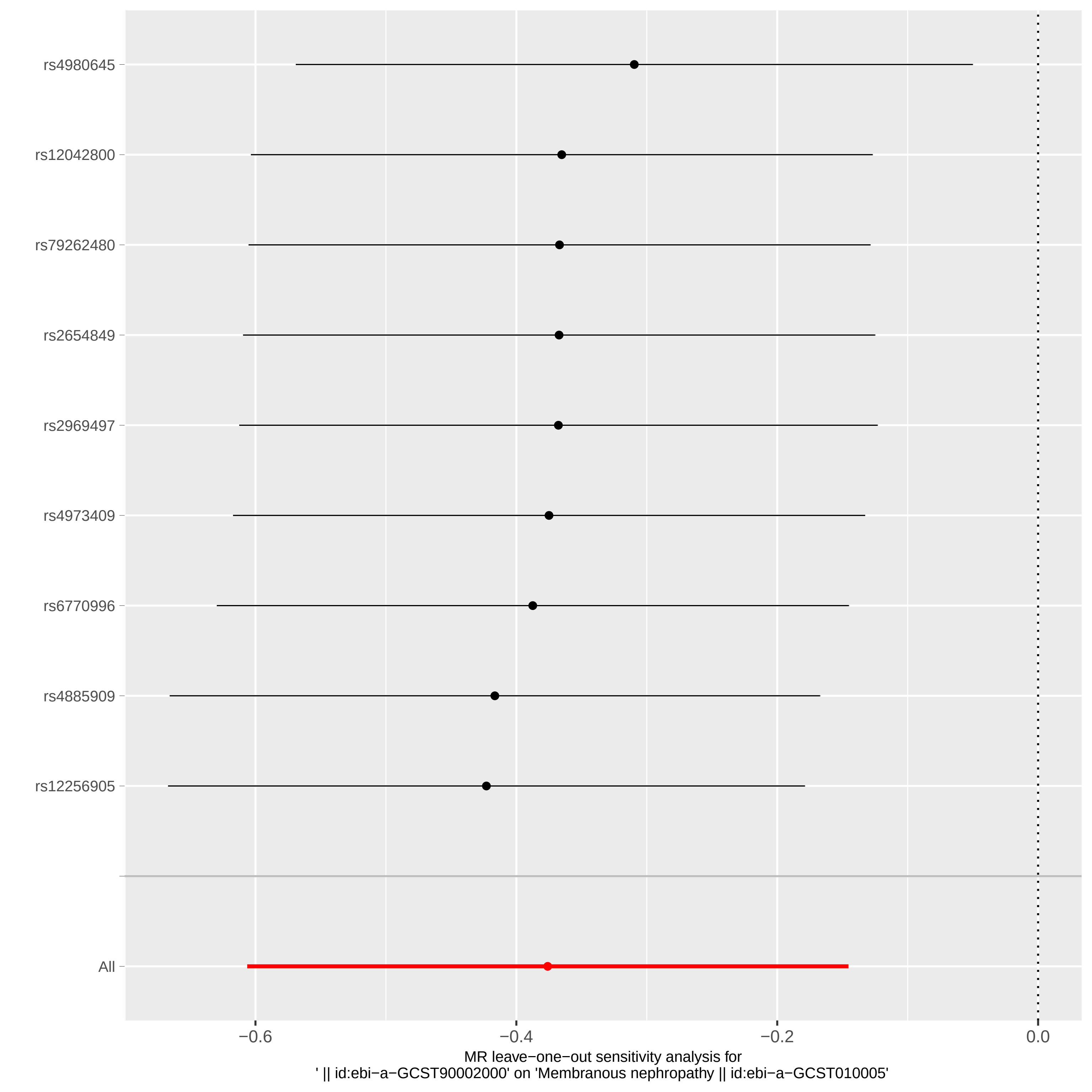
rs4980645
rs12042800
rs79262480
rs2654849
rs2969497
rs4973409
rs6770996
rs4885909
rs12256905
All
−0.6
−0.4
−0.2
0.0
MR leave−one−out sensitivity analysis for
' || id:ebi−a−GCST90002000' on 'Membranous nephropathy || id:ebi−a−GCST010005'

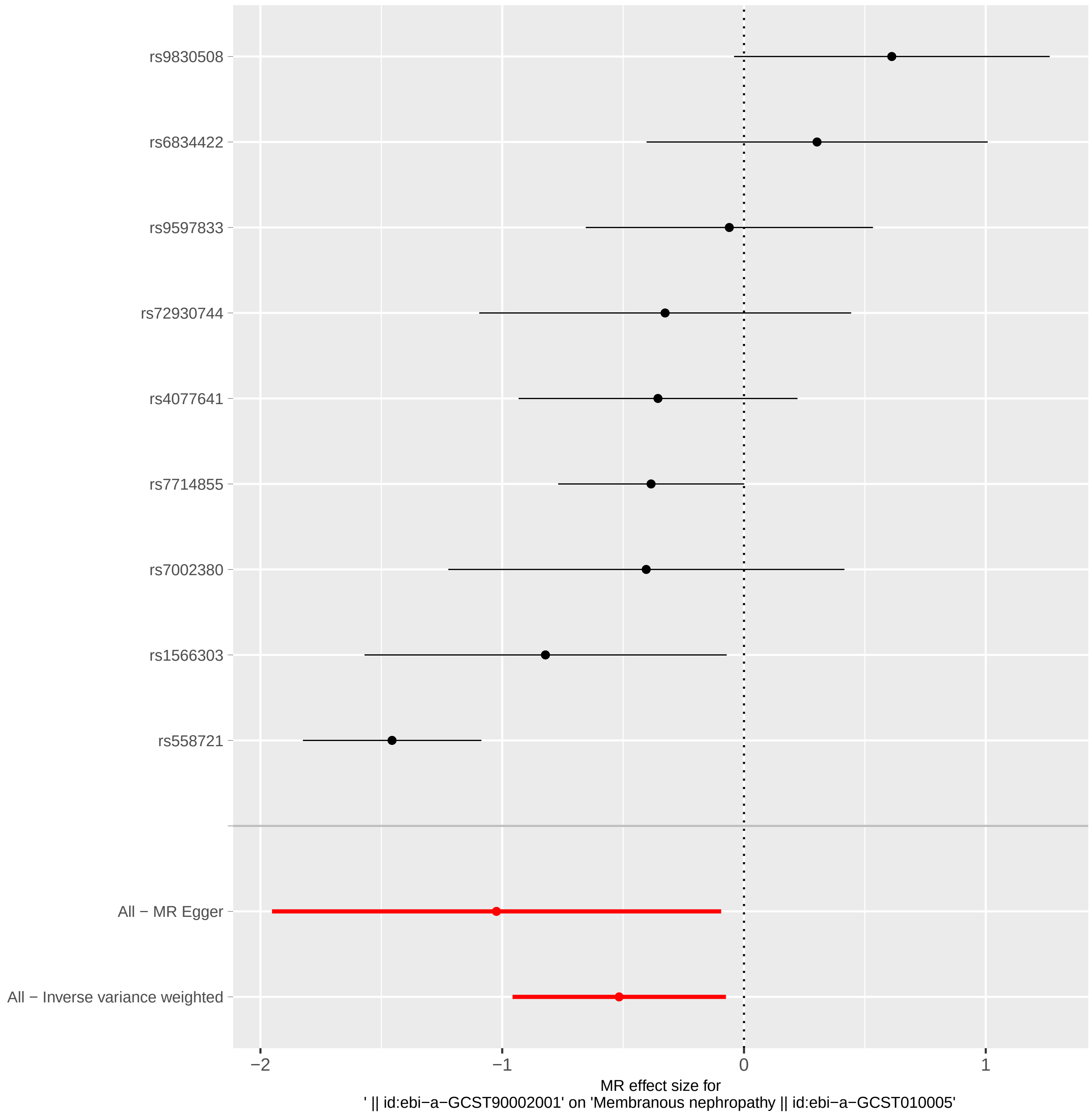
rs9830508
rs6834422
rs9597833
rs72930744
rs4077641
rs7714855
rs7002380
rs1566303
rs558721
All − MR Egger
All − Inverse variance weighted
−2
−1
0
1
MR effect size for
' || id:ebi−a−GCST90002001' on 'Membranous nephropathy || id:ebi−a−GCST010005'

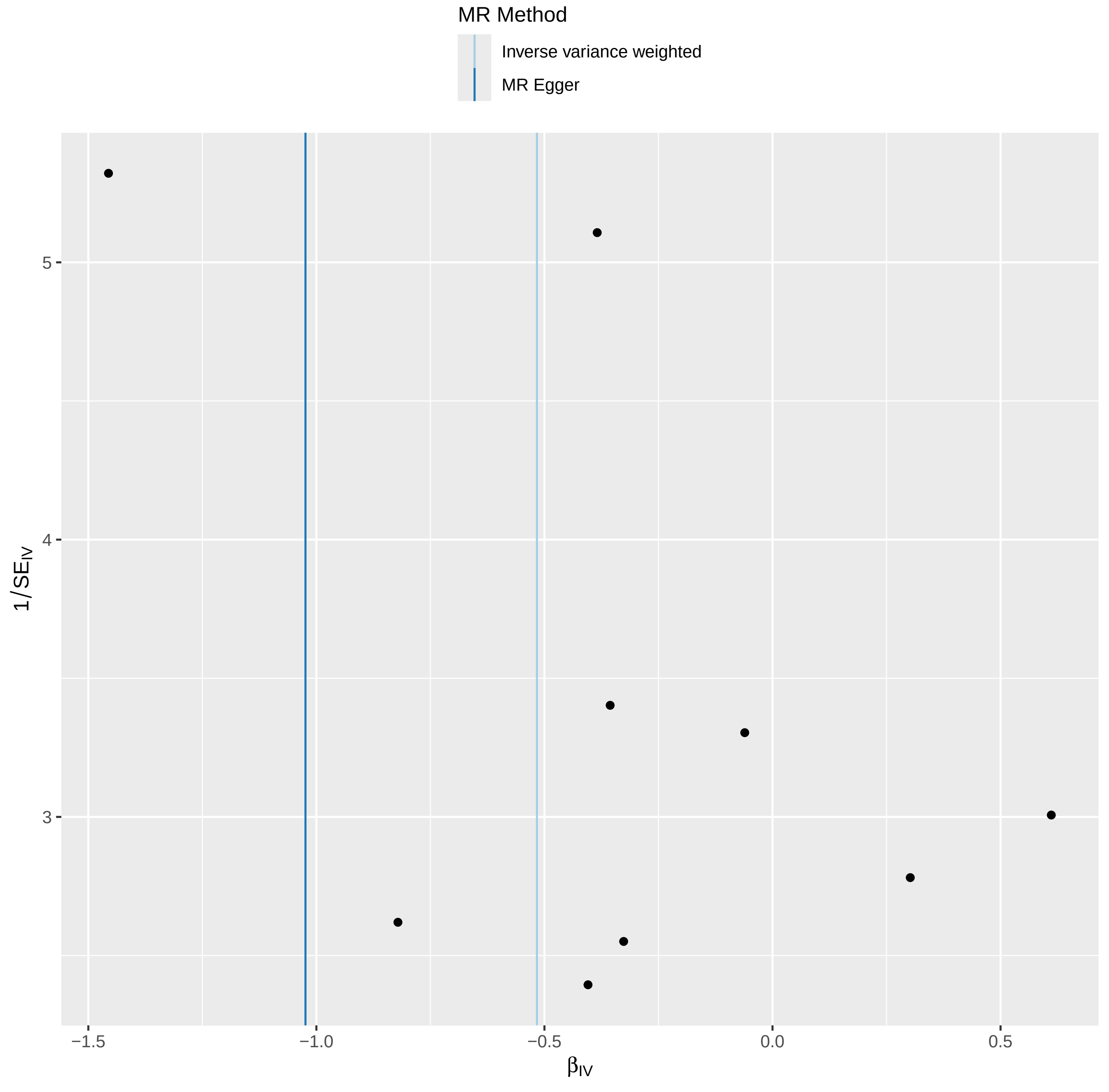

MR Method
Inverse variance weighted
MR Egger
5
4
3
1/SE$_{IV}$
−1.5
−1.0
−0.5
0.0
0.5
β$_{IV}$

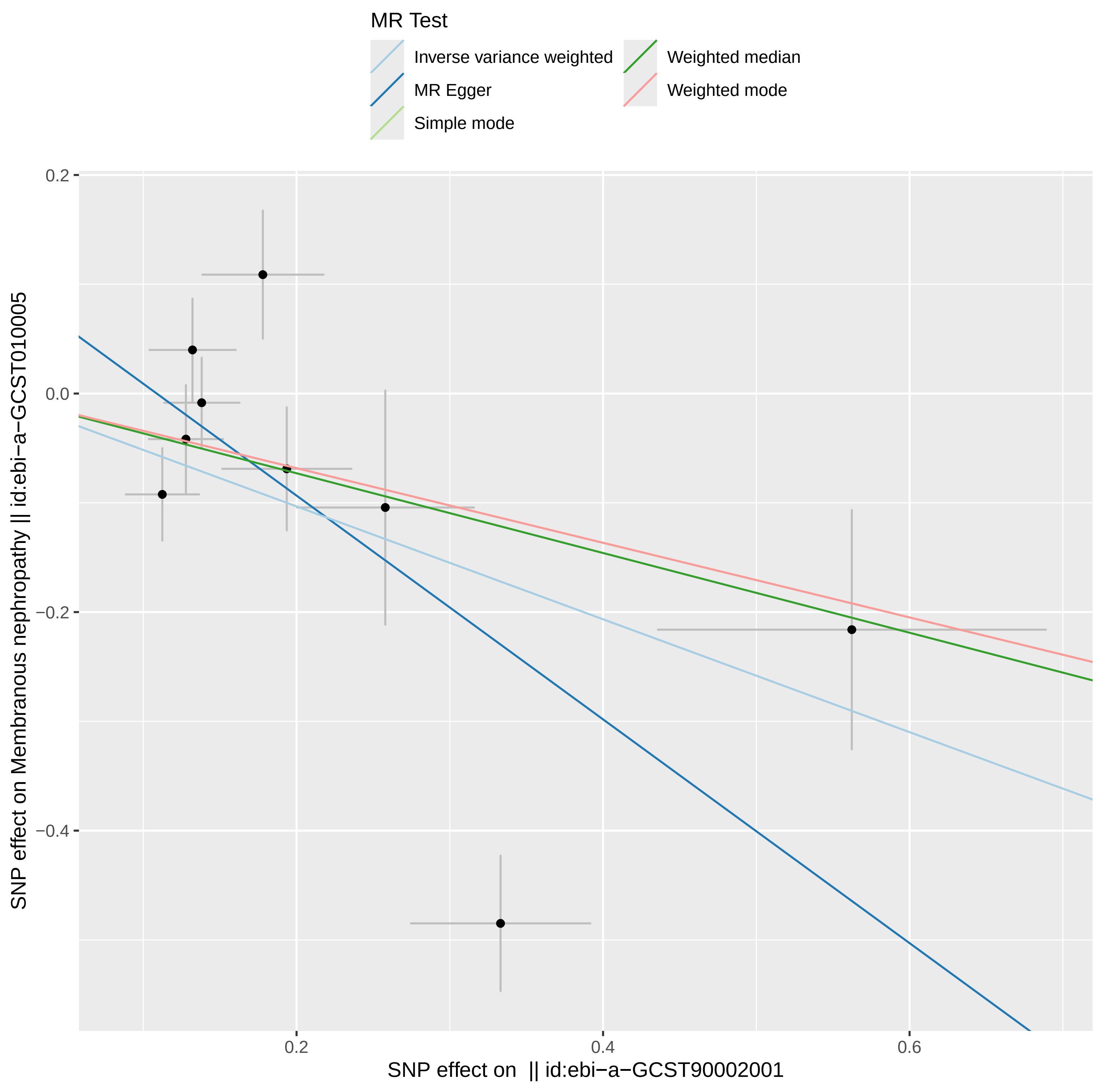
MR Test
Inverse variance weighted
MR Egger
Simple mode
Weighted median
Weighted mode
0.2
0.0
−0.2
−0.4
0.2
0.4
0.6
SNP effect on Membranous nephropathy || id:ebi−a−GCST010005
SNP effect on || id:ebi−a−GCST90002001

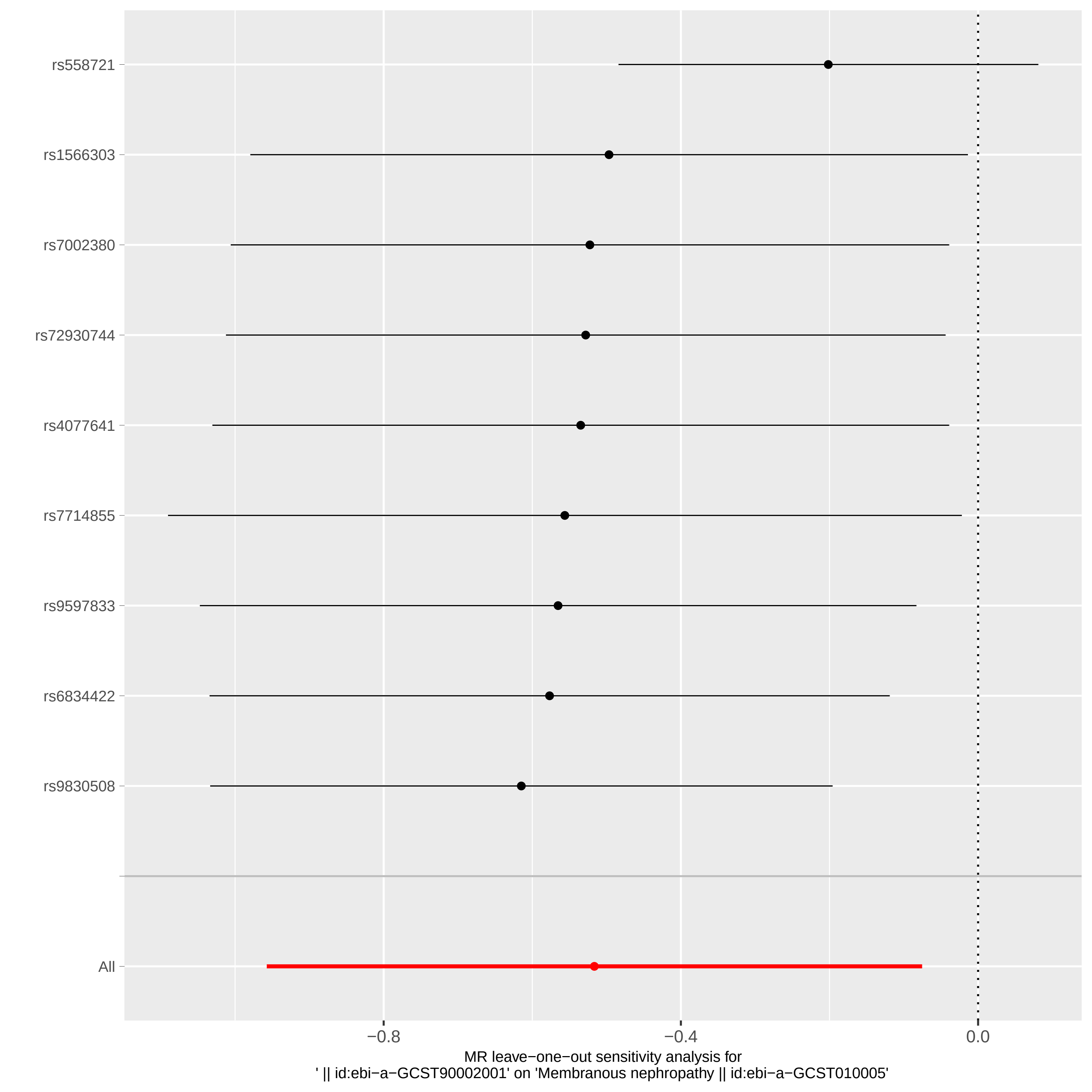
rs558721
rs1566303
rs7002380
rs72930744
rs4077641
rs7714855
rs9597833
rs6834422
rs9830508
All
−0.8
−0.4
0.0
MR leave−one−out sensitivity analysis for
' || id:ebi−a−GCST90002001' on 'Membranous nephropathy || id:ebi−a−GCST010005'

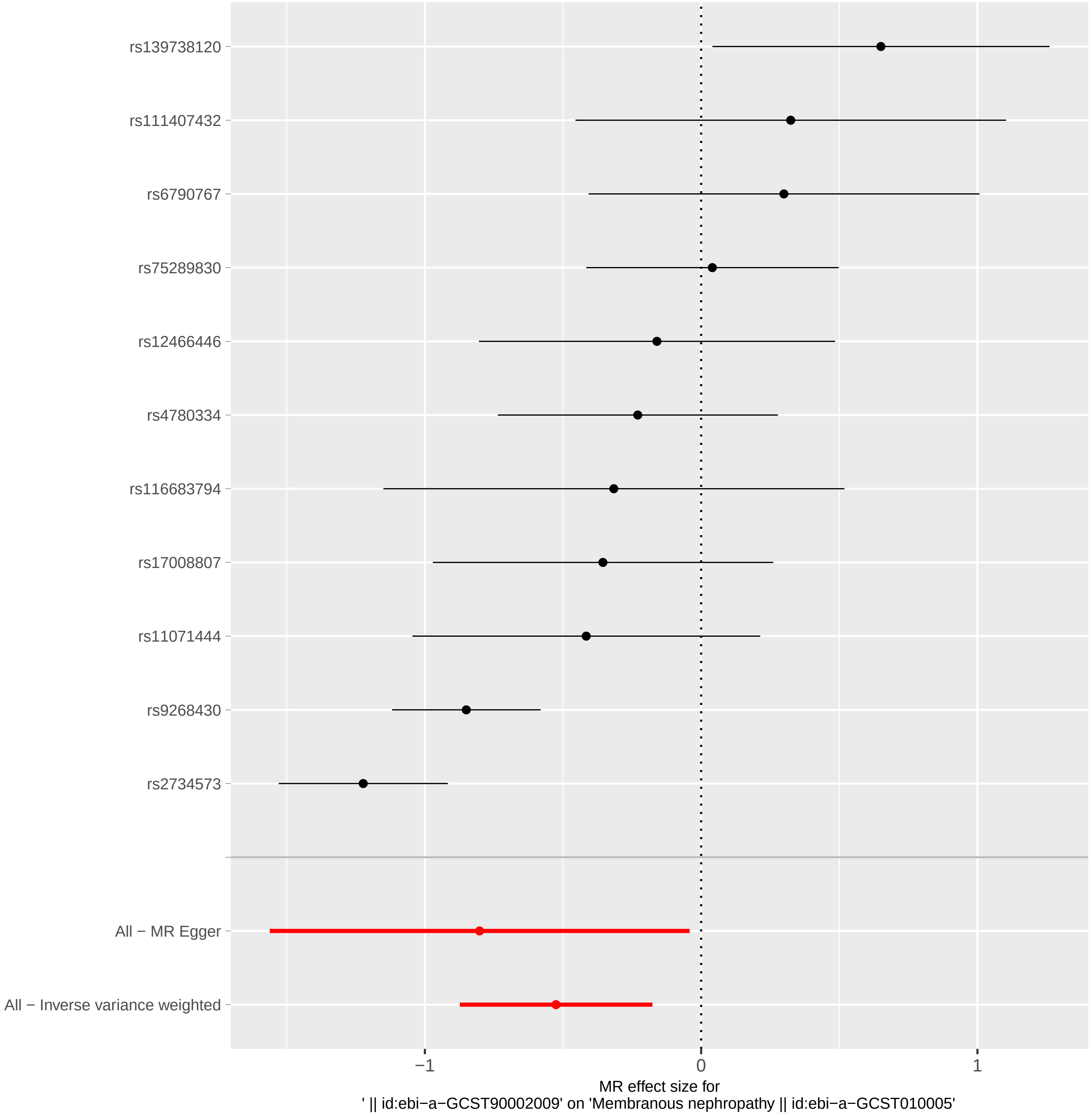
rs139738120
rs111407432
rs6790767
rs75289830
rs12466446
rs4780334
rs116683794
rs17008807
rs11071444
rs9268430
rs2734573
All − MR Egger
All − Inverse variance weighted
−1
0
1
MR effect size for
' || id:ebi−a−GCST90002009' on 'Membranous nephropathy || id:ebi−a−GCST010005'

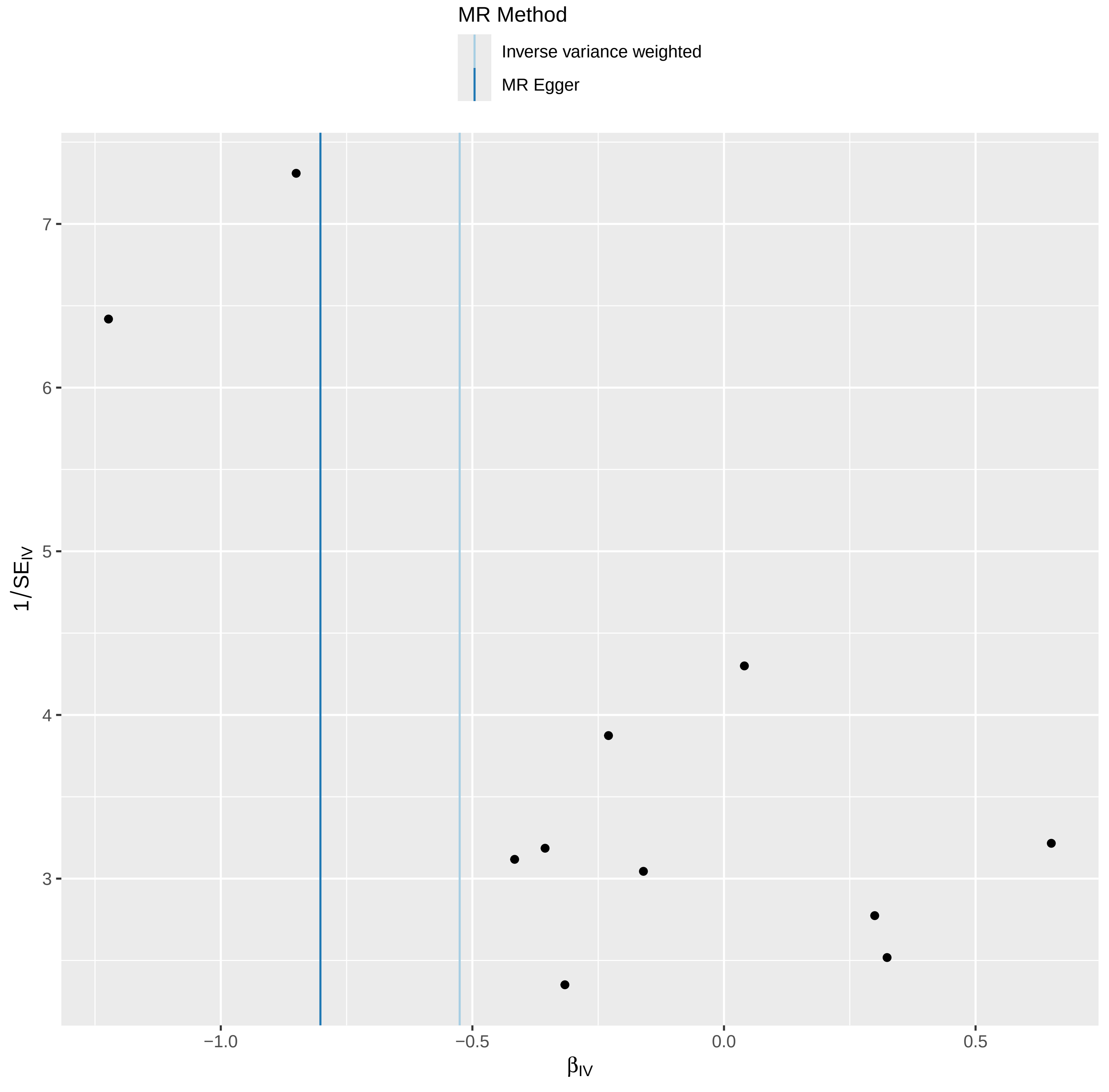

MR Method
Inverse variance weighted
MR Egger
1/SE$_{IV}$
7
6
5
4
3
−1.0
−0.5
0.0
0.5
β_{IV}

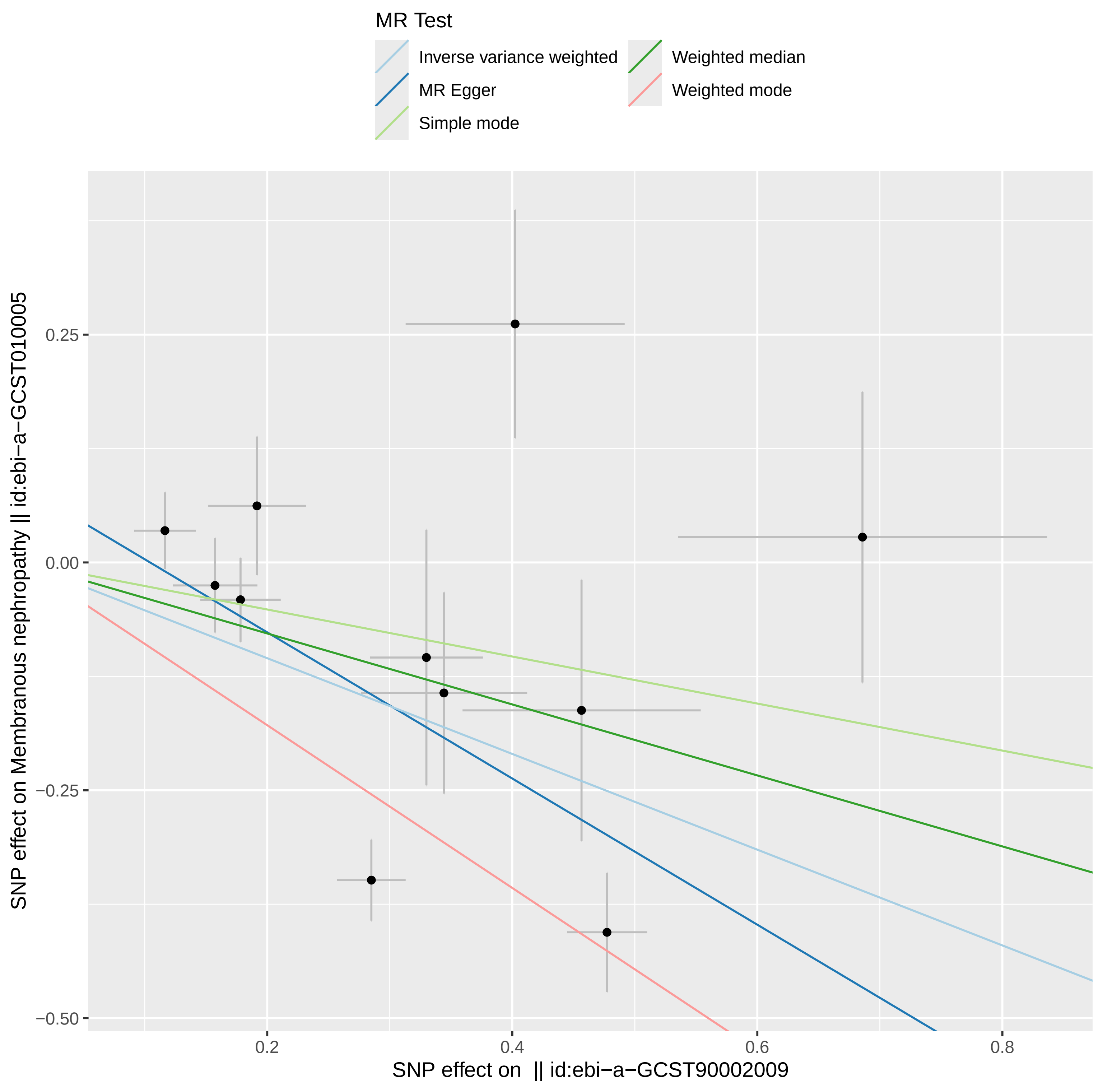

MR Test
Inverse variance weighted
MR Egger
Simple mode
Weighted median
Weighted mode
SNP effect on Membranous nephropathy || id:ebi−a−GCST010005
0.25
0.00
−0.25
−0.50
0.2
0.4
0.6
0.8
SNP effect on || id:ebi−a−GCST90002009

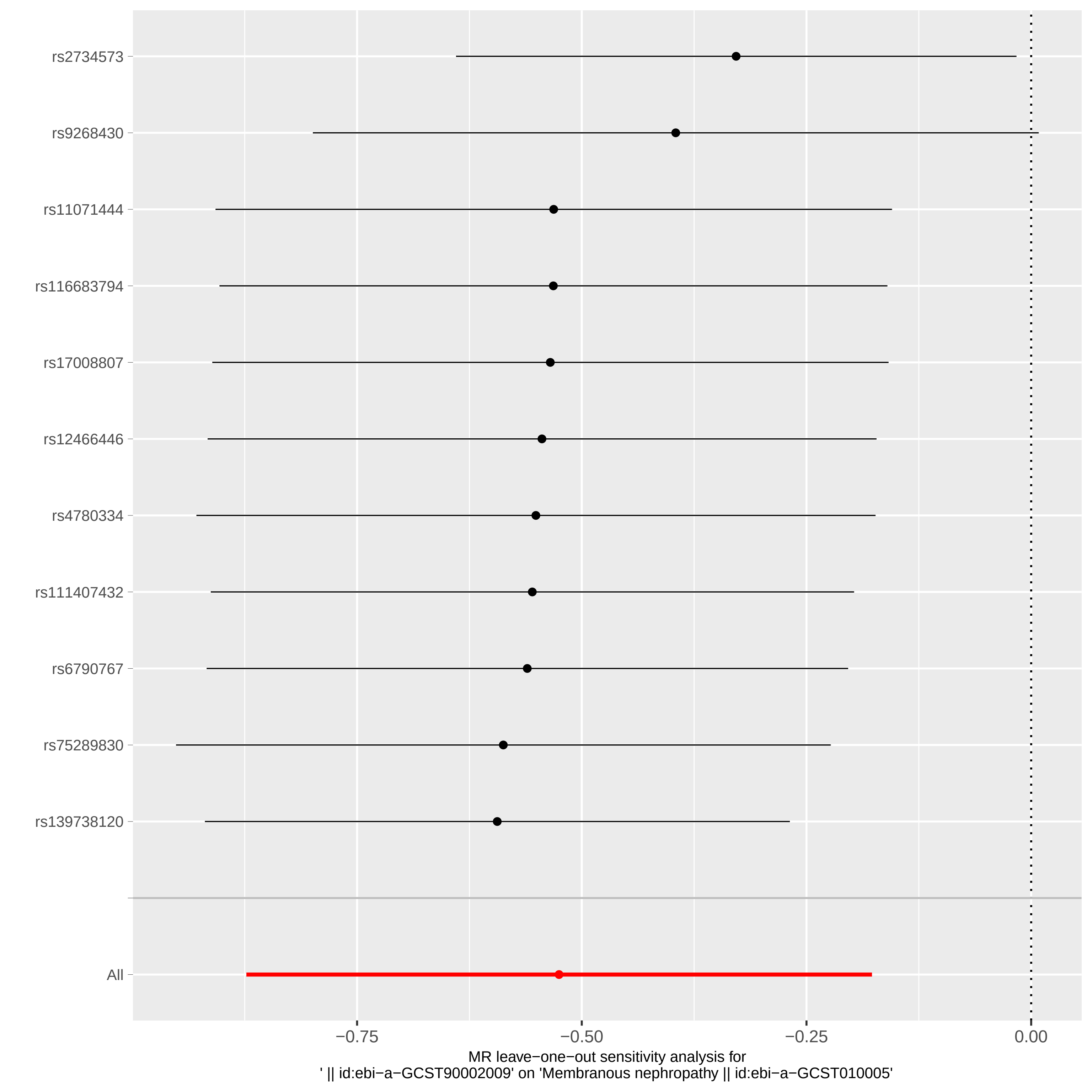
rs2734573
rs9268430
rs11071444
rs116683794
rs17008807
rs12466446
rs4780334
rs111407432
rs6790767
rs75289830
rs139738120
All
−0.75
−0.50
−0.25
0.00
MR leave−one−out sensitivity analysis for
' || id:ebi−a−GCST90002009' on 'Membranous nephropathy || id:ebi−a−GCST010005'

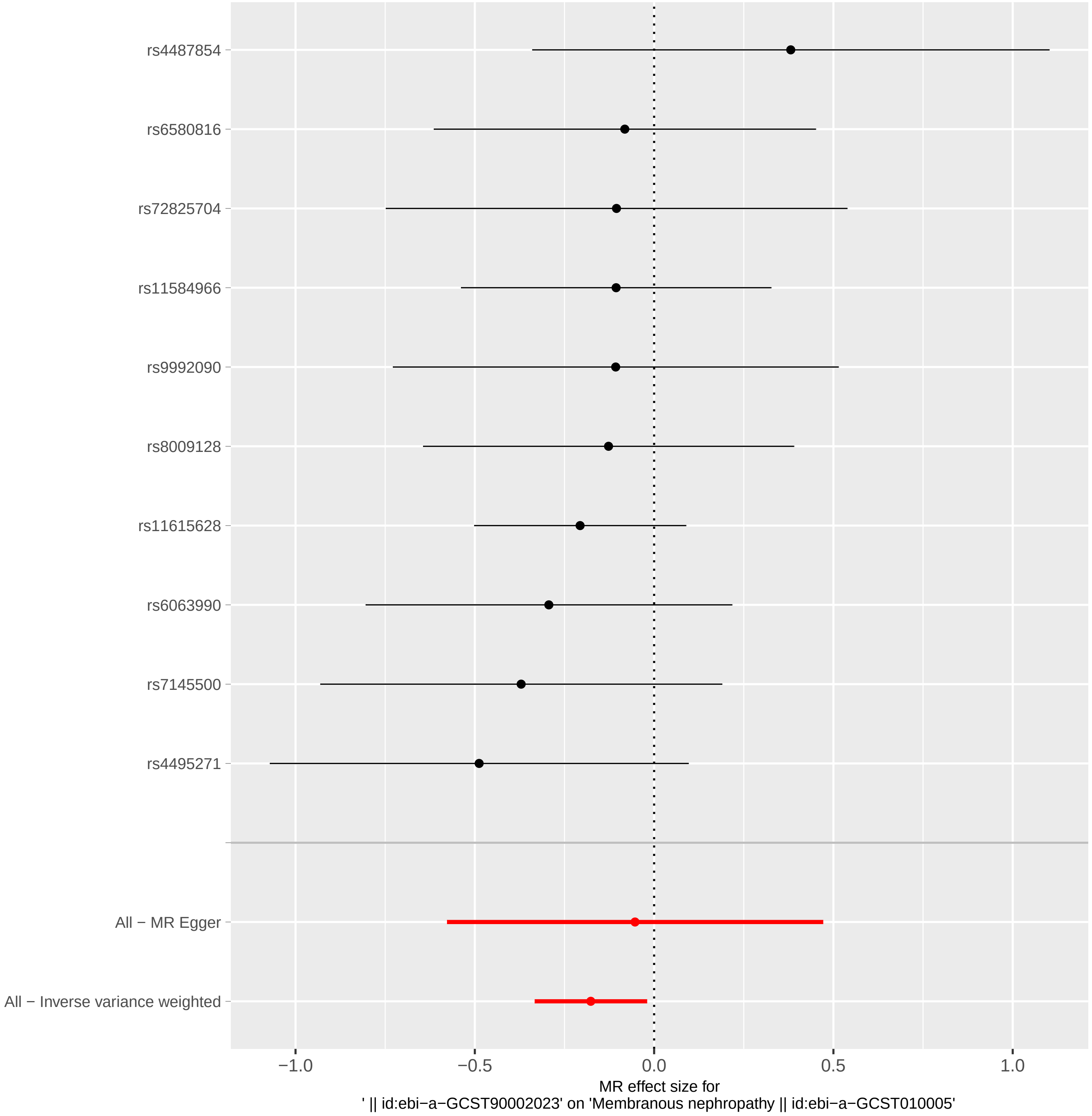

rs4487854
rs6580816
rs72825704
rs11584966
rs9992090
rs8009128
rs11615628
rs6063990
rs7145500
rs4495271
All − MR Egger
All − Inverse variance weighted
−1.0
−0.5
0.0
0.5
1.0
MR effect size for
' || id:ebi−a−GCST90002023' on 'Membranous nephropathy || id:ebi−a−GCST010005'

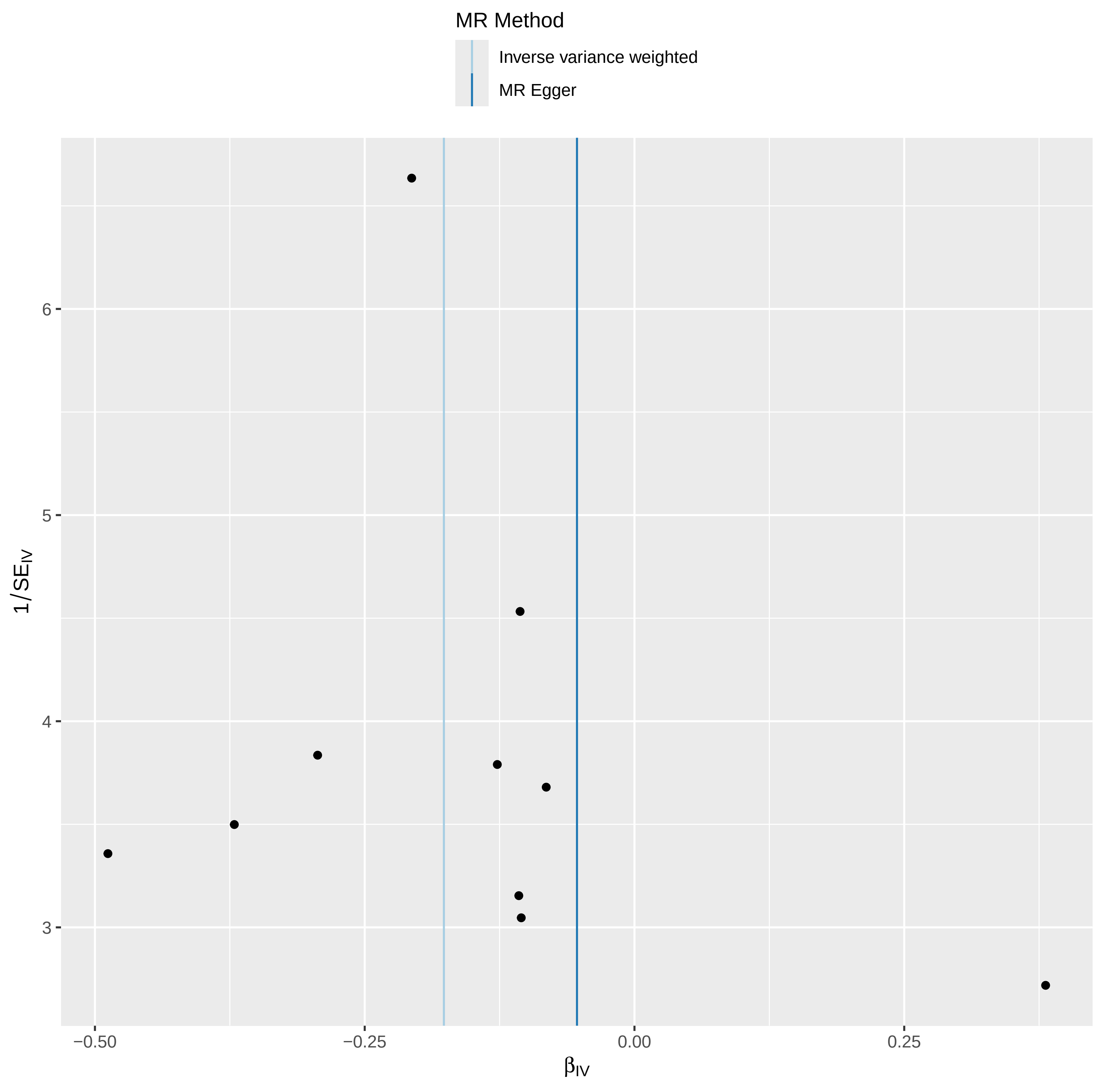
MR Method
Inverse variance weighted
MR Egger
6
5
4
3
1/SE$_{IV}$
−0.50
−0.25
0.00
0.25
β$_{IV}$

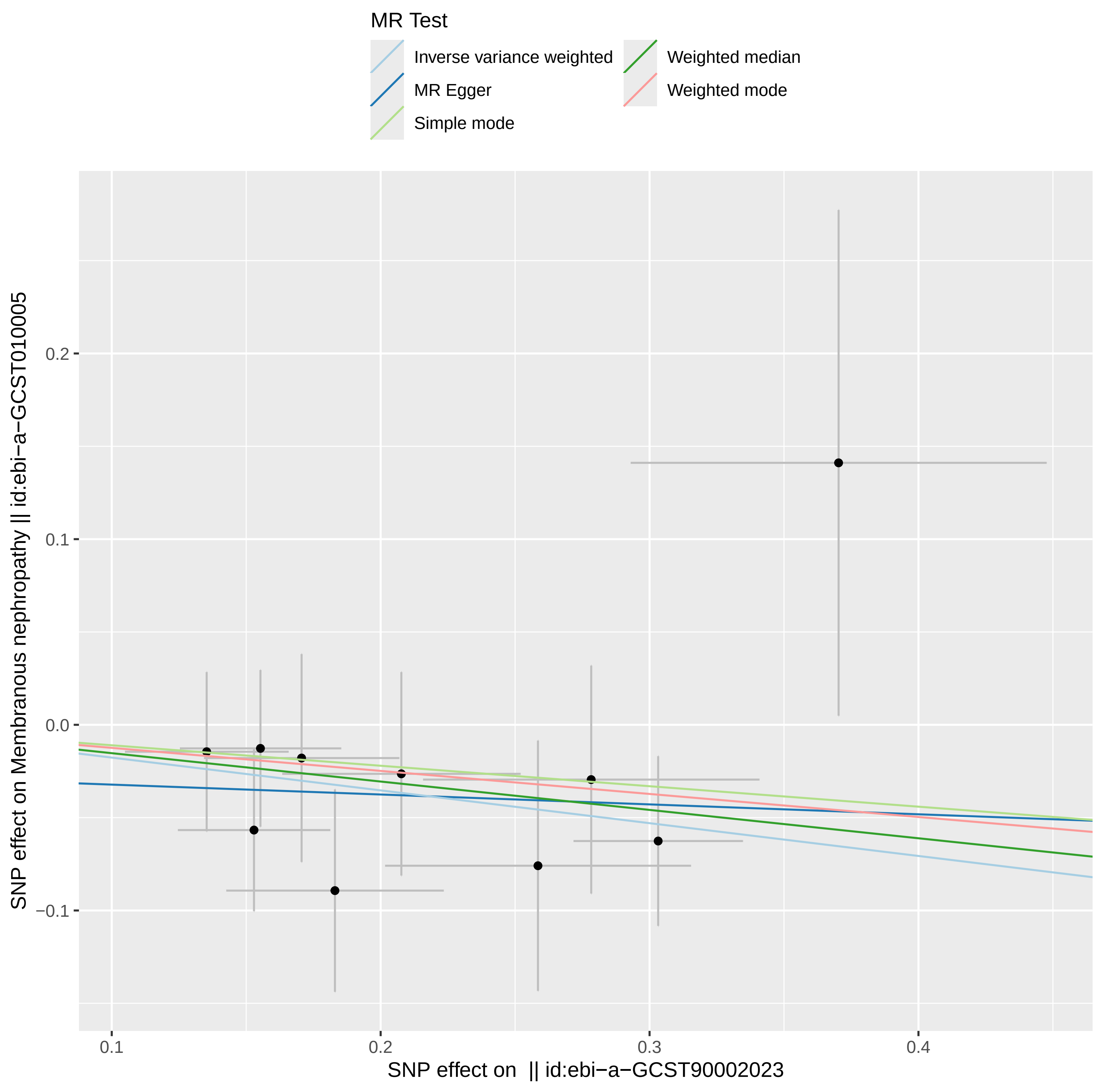

MR Test
Inverse variance weighted
MR Egger
Simple mode
Weighted median
Weighted mode
SNP effect on Membranous nephropathy || id:ebi−a−GCST010005
0.2
0.1
0.0
−0.1
0.1
0.2
0.3
0.4
SNP effect on || id:ebi−a−GCST90002023

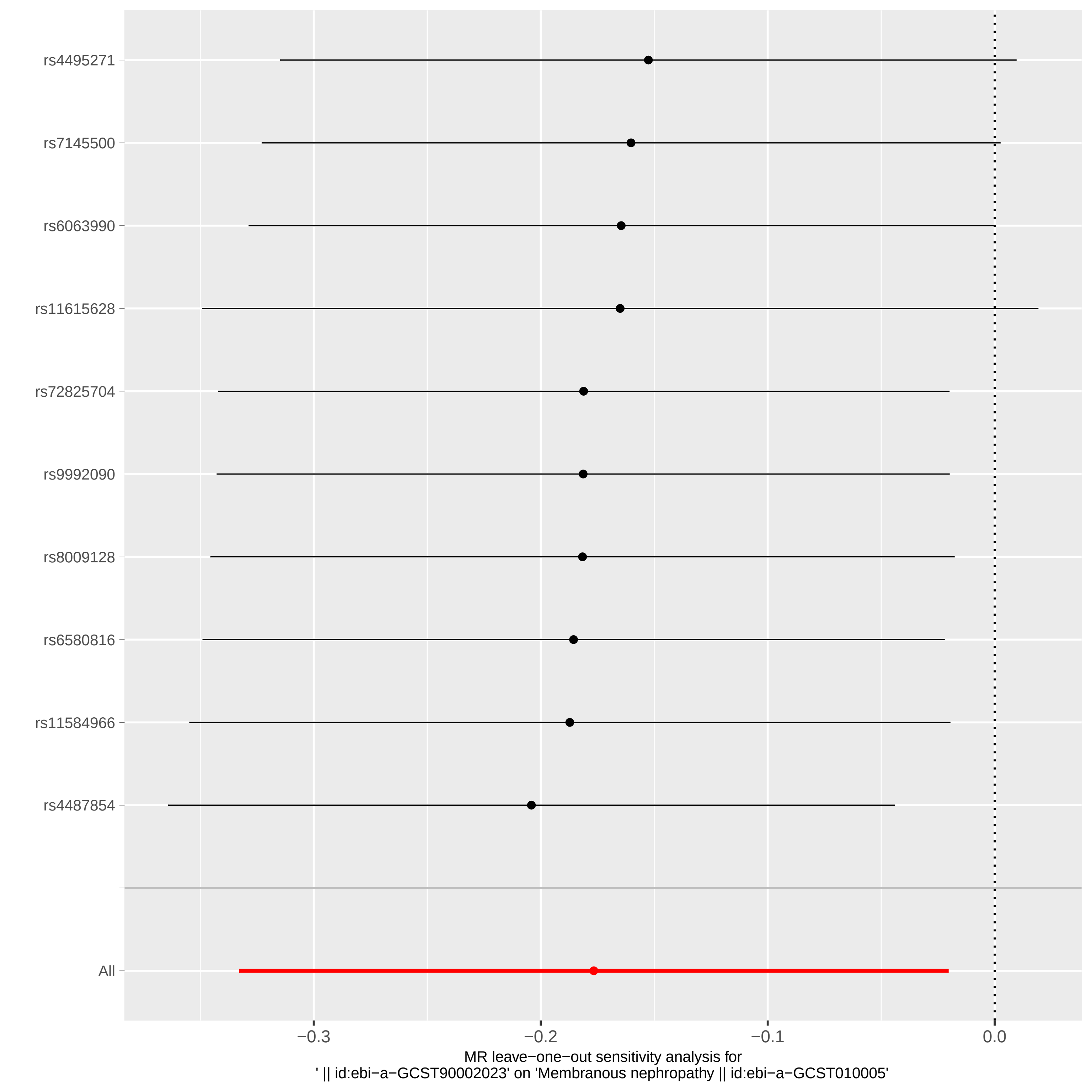

rs4495271
rs7145500
rs6063990
rs11615628
rs72825704
rs9992090
rs8009128
rs6580816
rs11584966
rs4487854
All
−0.3
−0.2
−0.1
0.0
MR leave−one−out sensitivity analysis for
' || id:ebi−a−GCST90002023' on 'Membranous nephropathy || id:ebi−a−GCST010005'

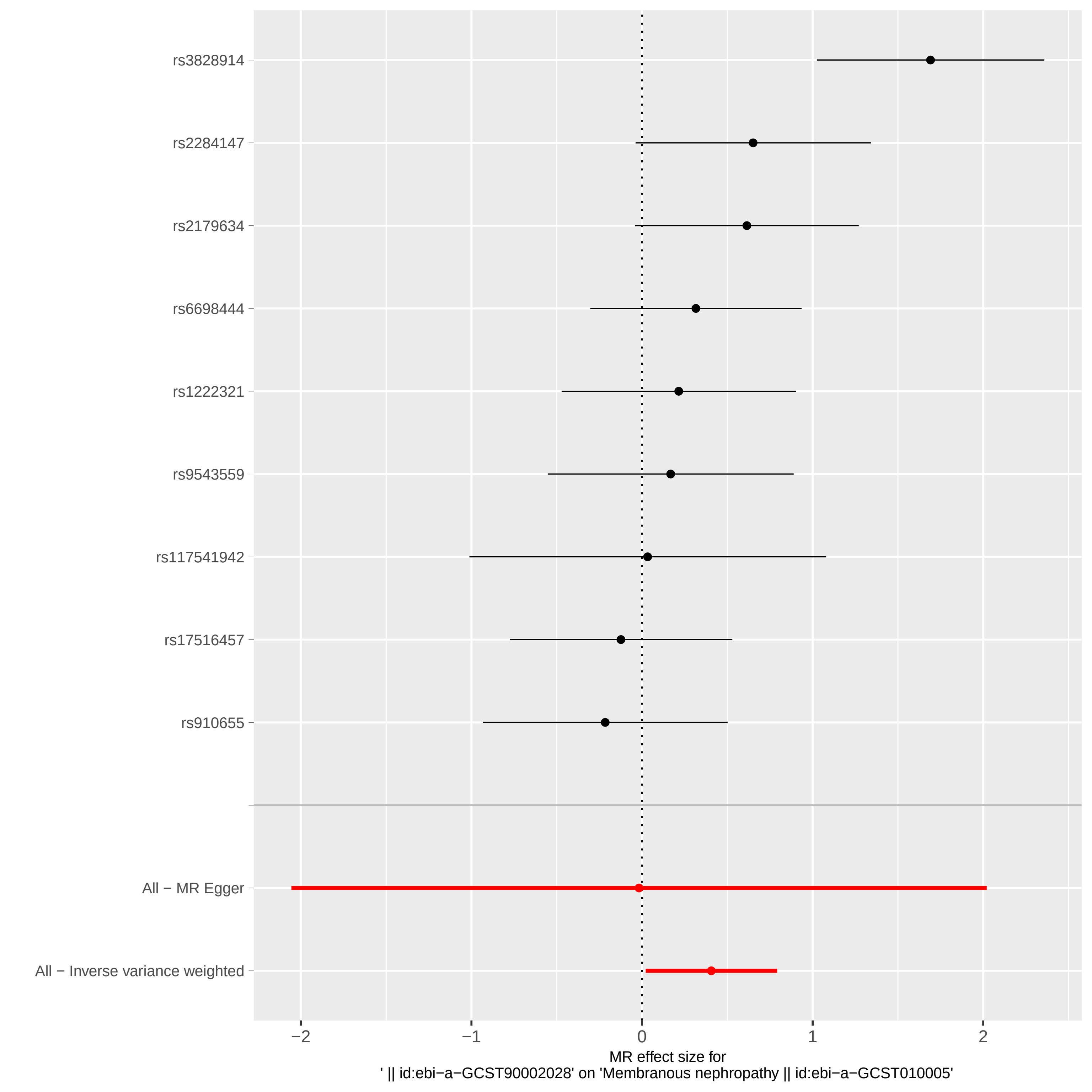

rs3828914
rs2284147
rs2179634
rs6698444
rs1222321
rs9543559
rs117541942
rs17516457
rs910655
All − MR Egger
All − Inverse variance weighted
−2
−1
0
1
2
MR effect size for
' || id:ebi−a−GCST90002028' on 'Membranous nephropathy || id:ebi−a−GCST010005'

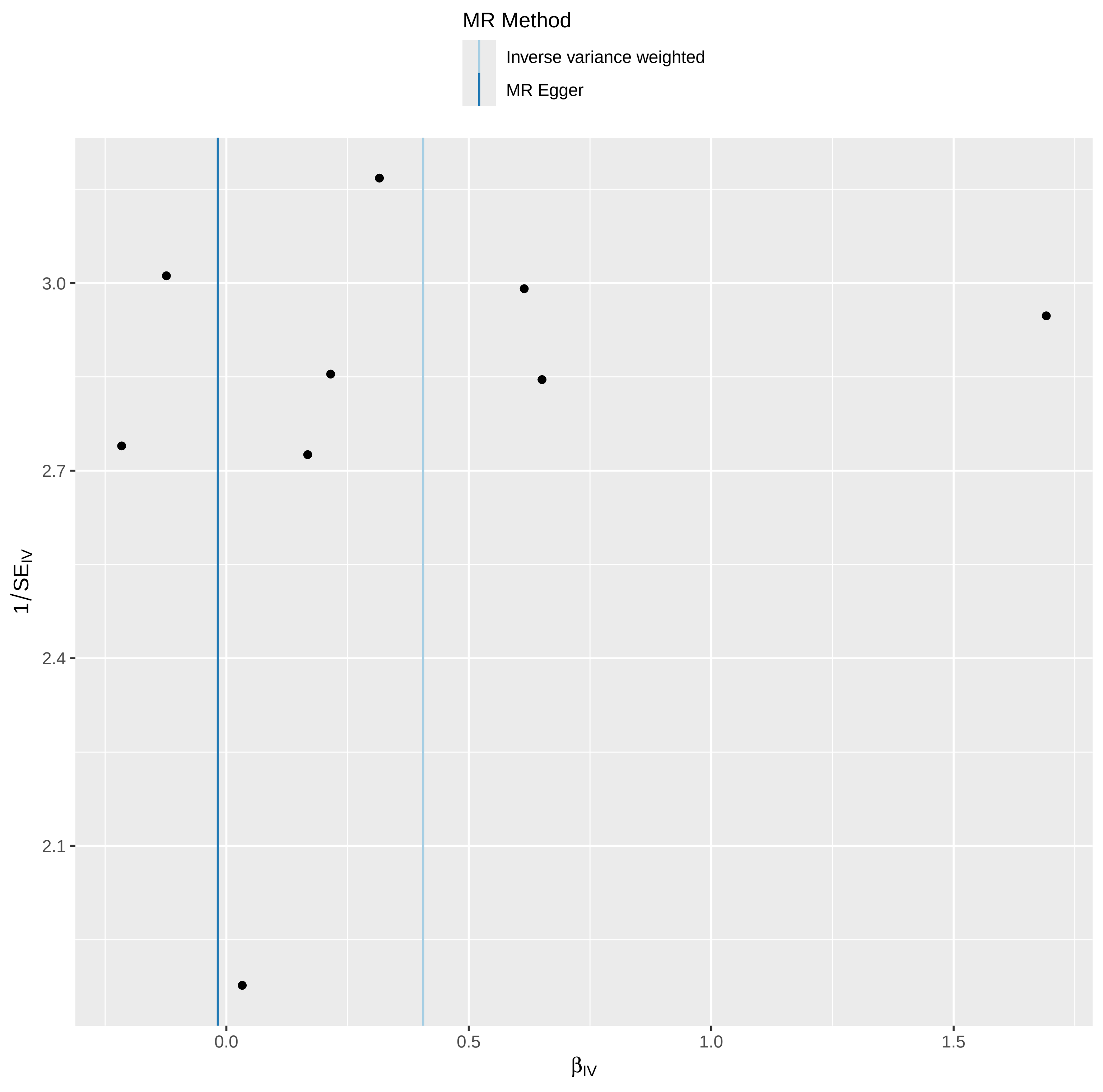

MR Method
Inverse variance weighted
MR Egger
3.0
2.7
2.4
2.1
1/SE$_{IV}$
0.0
0.5
1.0
1.5
β$_{IV}$

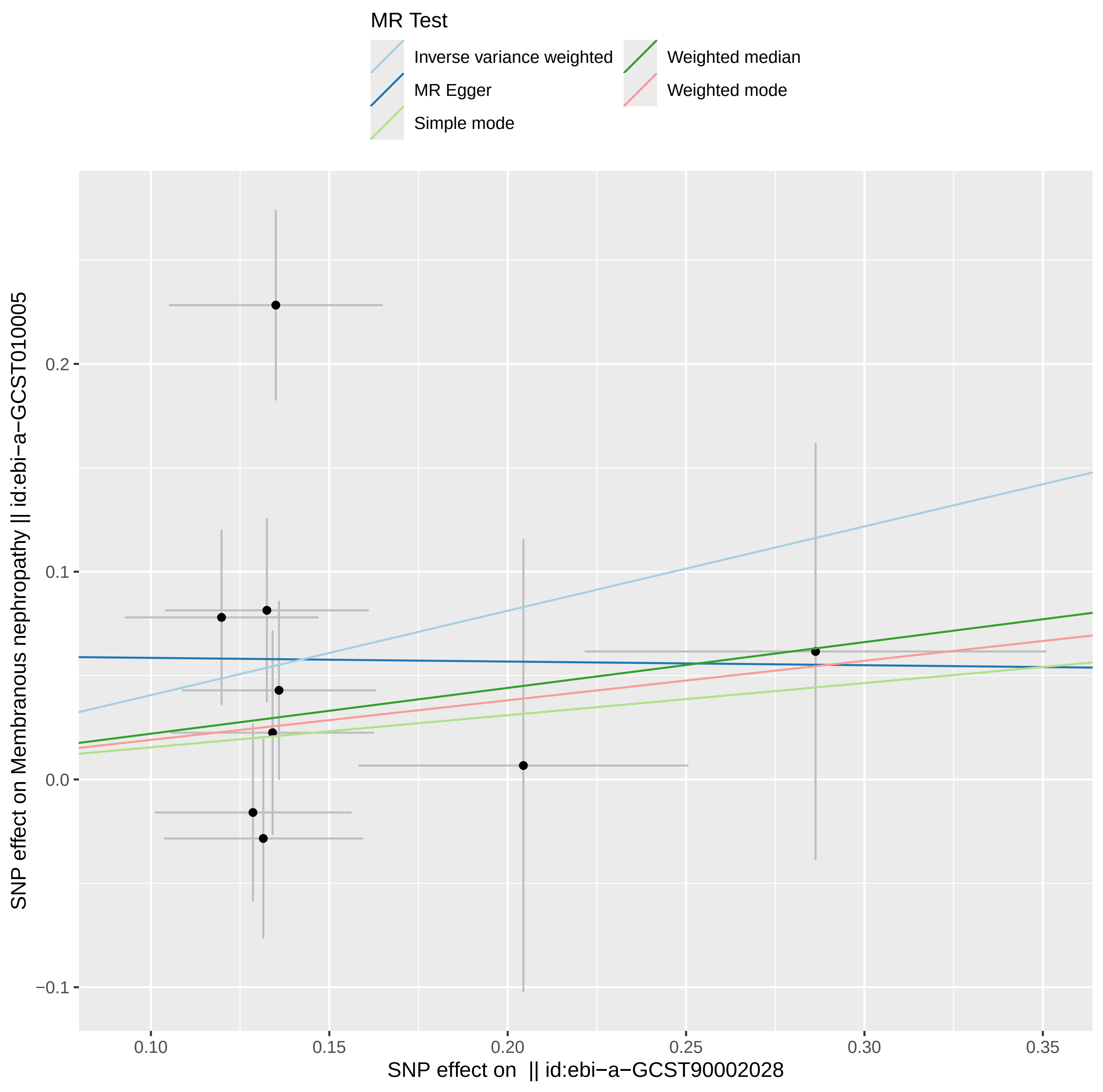
MR Test
Inverse variance weighted
MR Egger
Simple mode
Weighted median
Weighted mode
SNP effect on Membranous nephropathy || id:ebi−a−GCST010005
0.2
0.1
0.0
−0.1
0.10
0.15
0.20
0.25
0.30
0.35
SNP effect on || id:ebi−a−GCST90002028

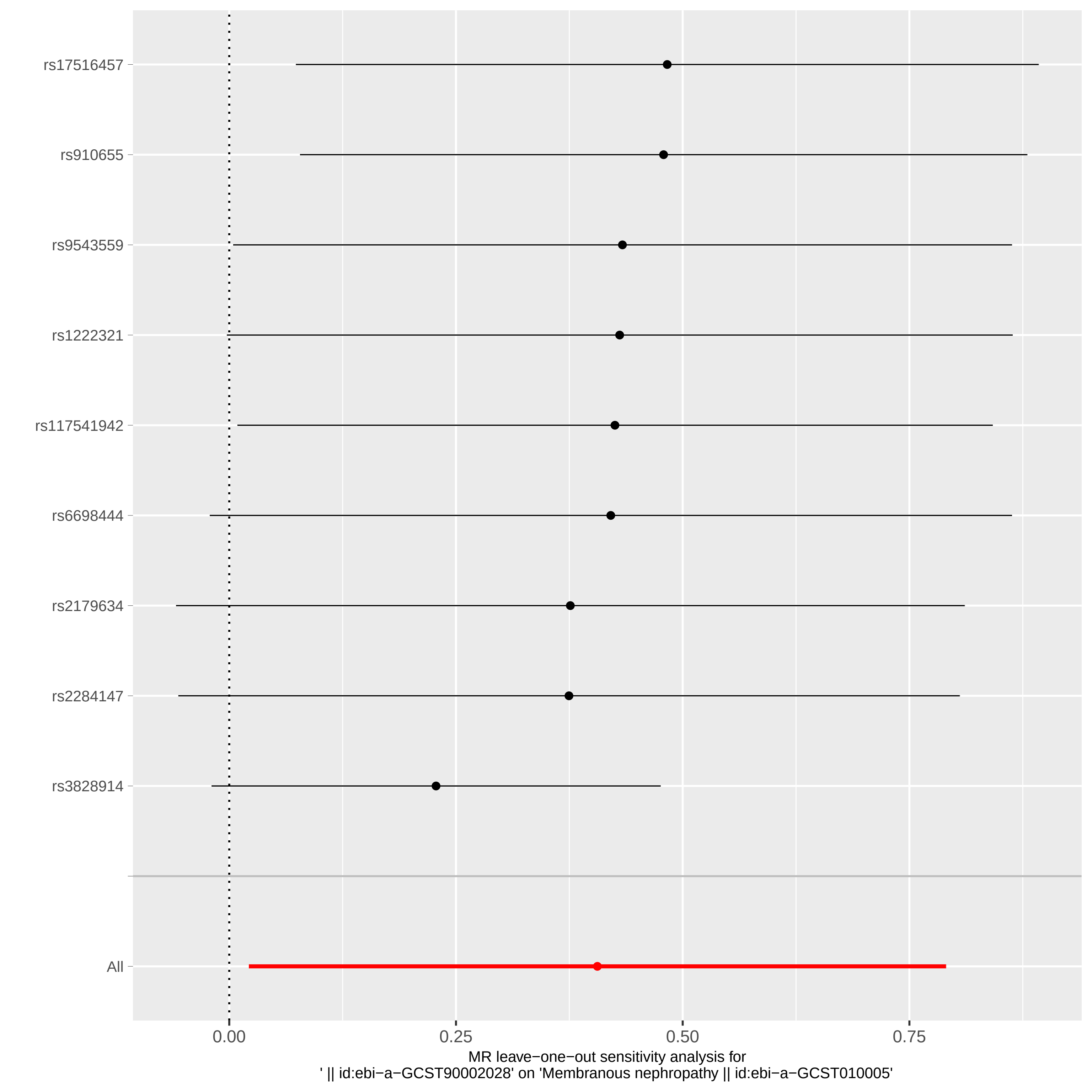
rs17516457
rs910655
rs9543559
rs1222321
rs117541942
rs6698444
rs2179634
rs2284147
rs3828914
All
0.00
0.25
0.50
0.75
MR leave−one−out sensitivity analysis for
' || id:ebi−a−GCST90002028' on 'Membranous nephropathy || id:ebi−a−GCST010005'

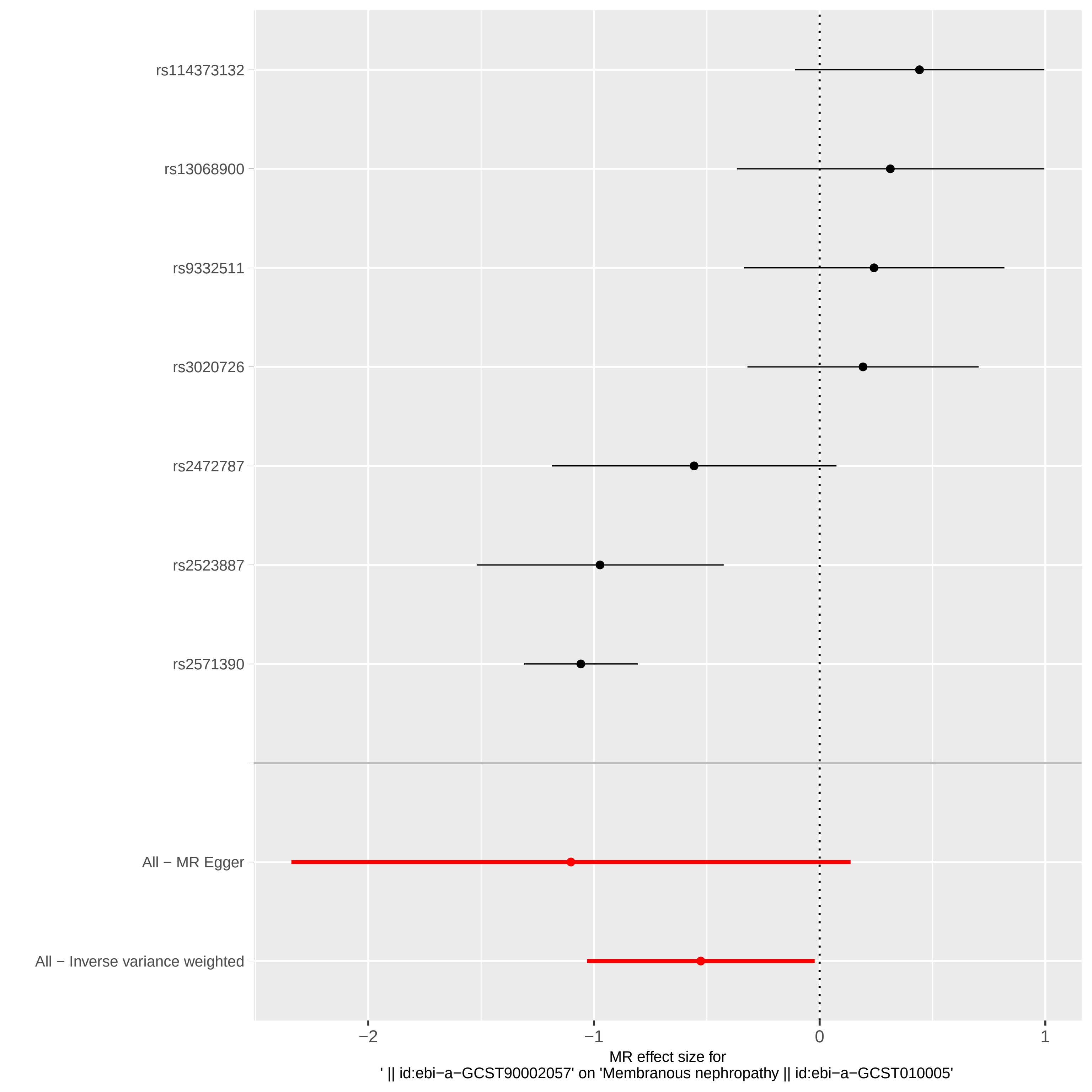
rs114373132
rs13068900
rs9332511
rs3020726
rs2472787
rs2523887
rs2571390
All − MR Egger
All − Inverse variance weighted
−2
−1
0
1
MR effect size for
' || id:ebi−a−GCST90002057' on 'Membranous nephropathy || id:ebi−a−GCST010005'

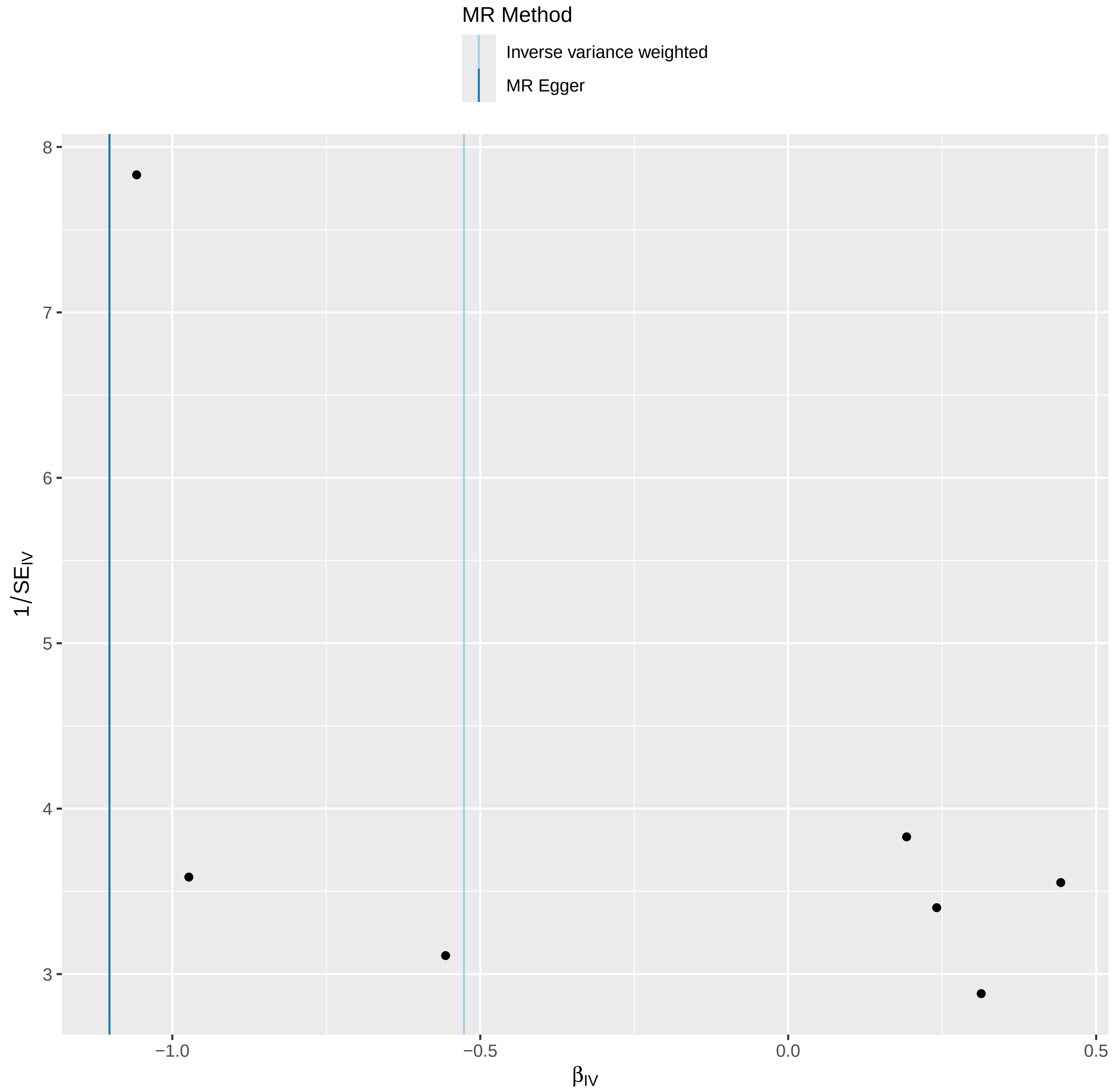

MR Method
Inverse variance weighted
MR Egger
1/SE$_{IV}$
8
7
6
5
4
3
−1.0
−0.5
0.0
0.5
β$_{IV}$

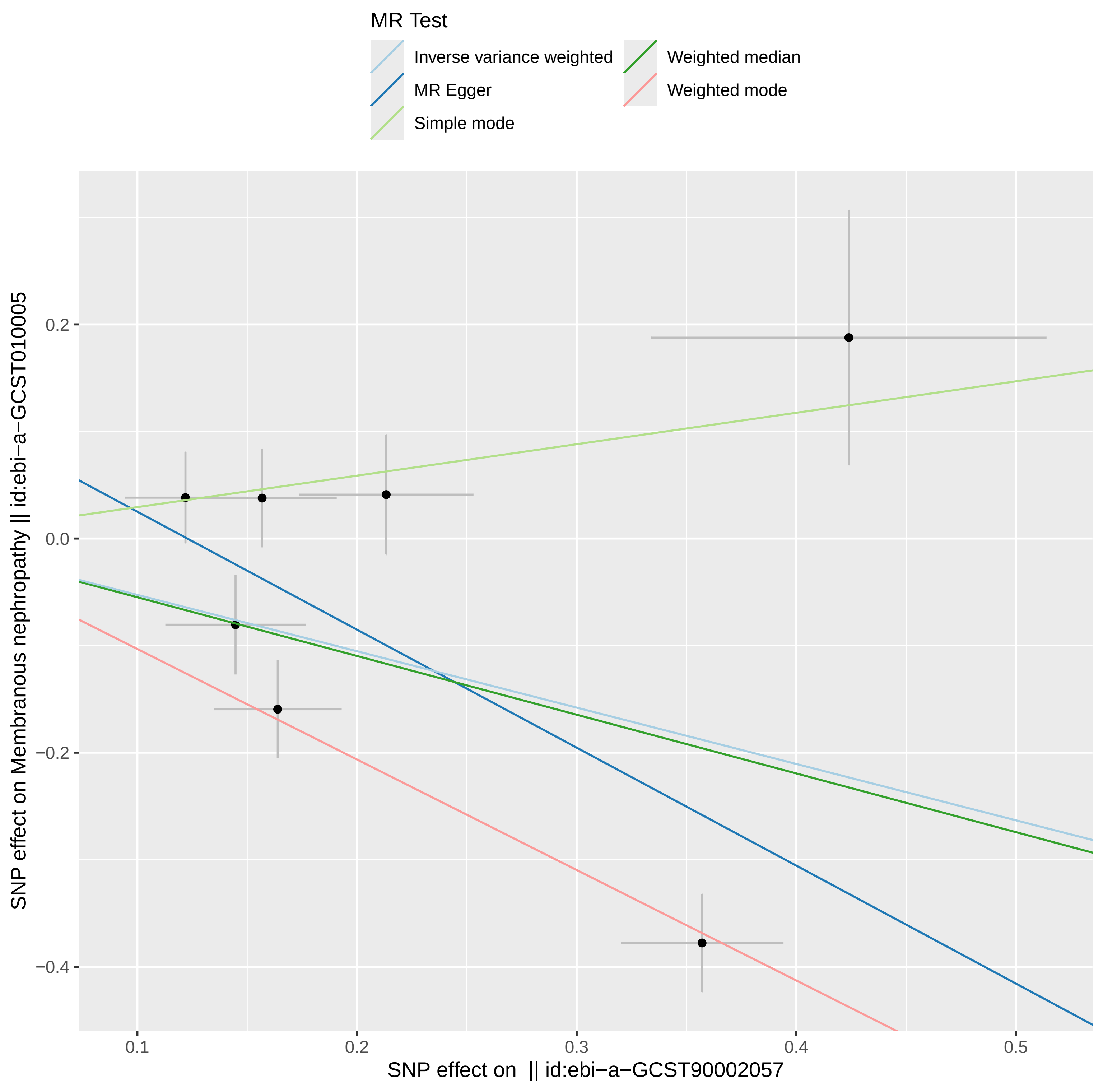

MR Test
Inverse variance weighted
MR Egger
Simple mode
Weighted median
Weighted mode
SNP effect on Membranous nephropathy || id:ebi−a−GCST010005
0.2
0.0
−0.2
−0.4
0.1
0.2
0.3
0.4
0.5
SNP effect on || id:ebi−a−GCST90002057

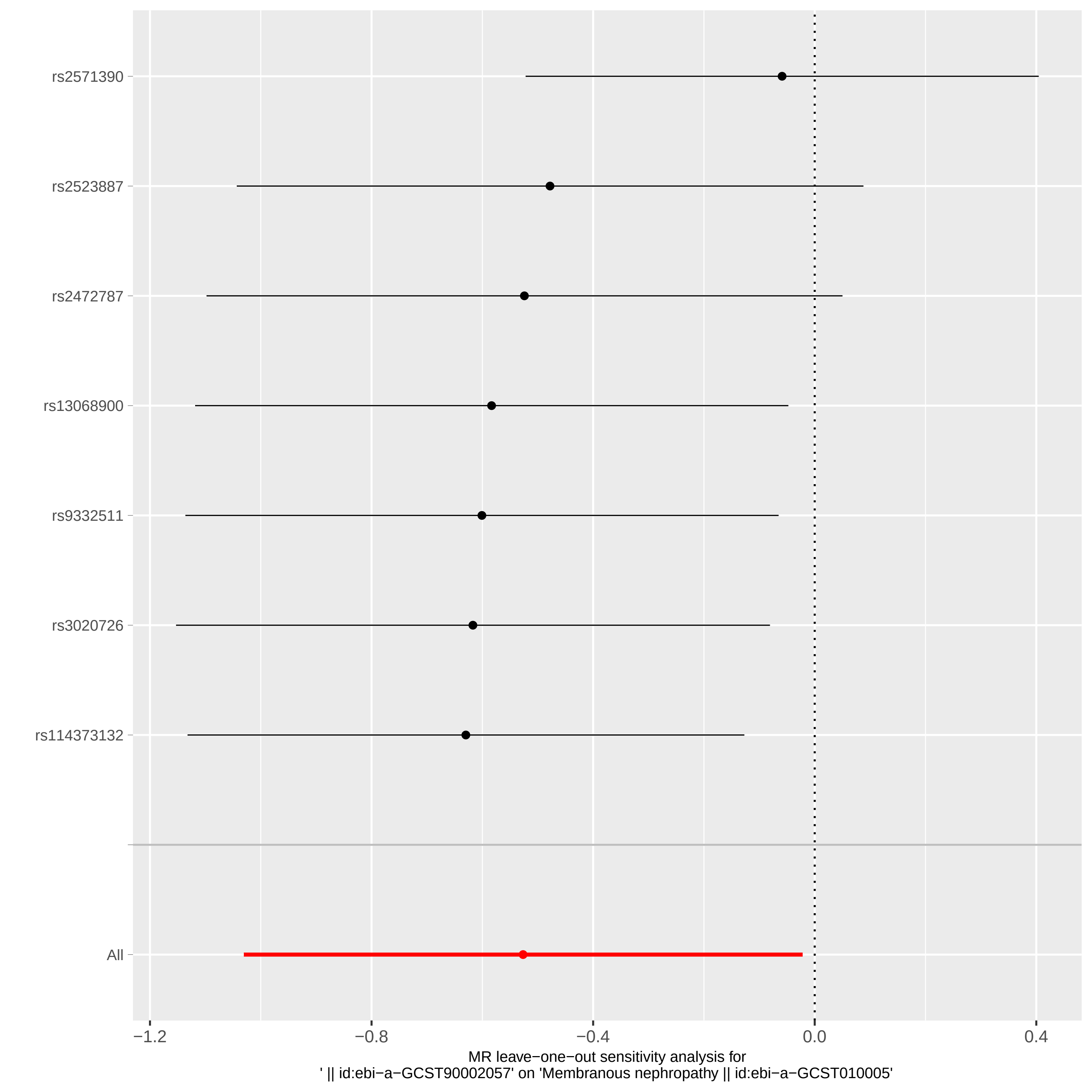

rs2571390
rs2523887
rs2472787
rs13068900
rs9332511
rs3020726
rs114373132
All
−1.2
−0.8
−0.4
0.0
0.4
MR leave−one−out sensitivity analysis for
' || id:ebi−a−GCST90002057' on 'Membranous nephropathy || id:ebi−a−GCST010005'

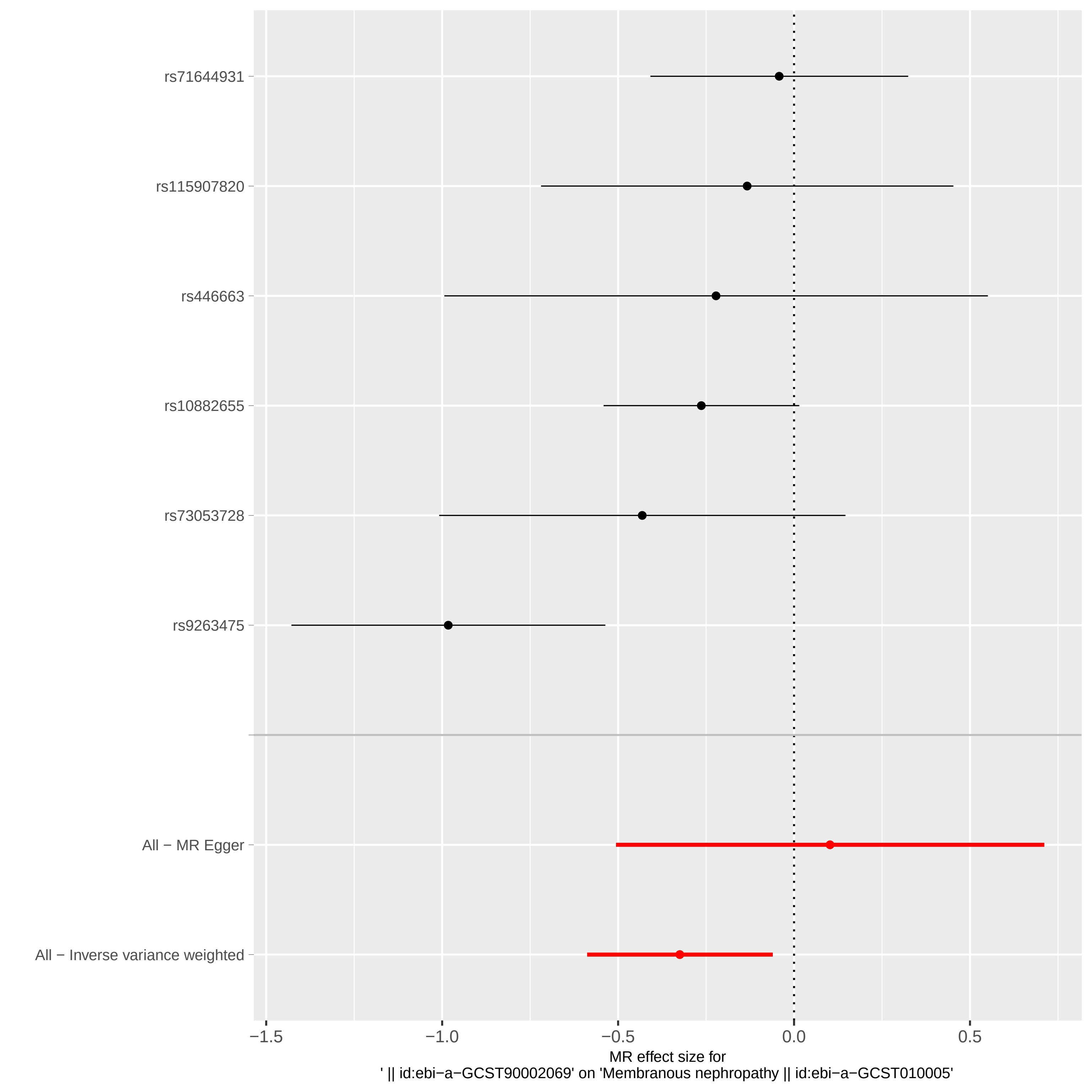
rs71644931
rs115907820
rs446663
rs10882655
rs73053728
rs9263475
All − MR Egger
All − Inverse variance weighted
−1.5
−1.0
−0.5
0.0
0.5
MR effect size for
' || id:ebi−a−GCST90002069' on 'Membranous nephropathy || id:ebi−a−GCST010005'

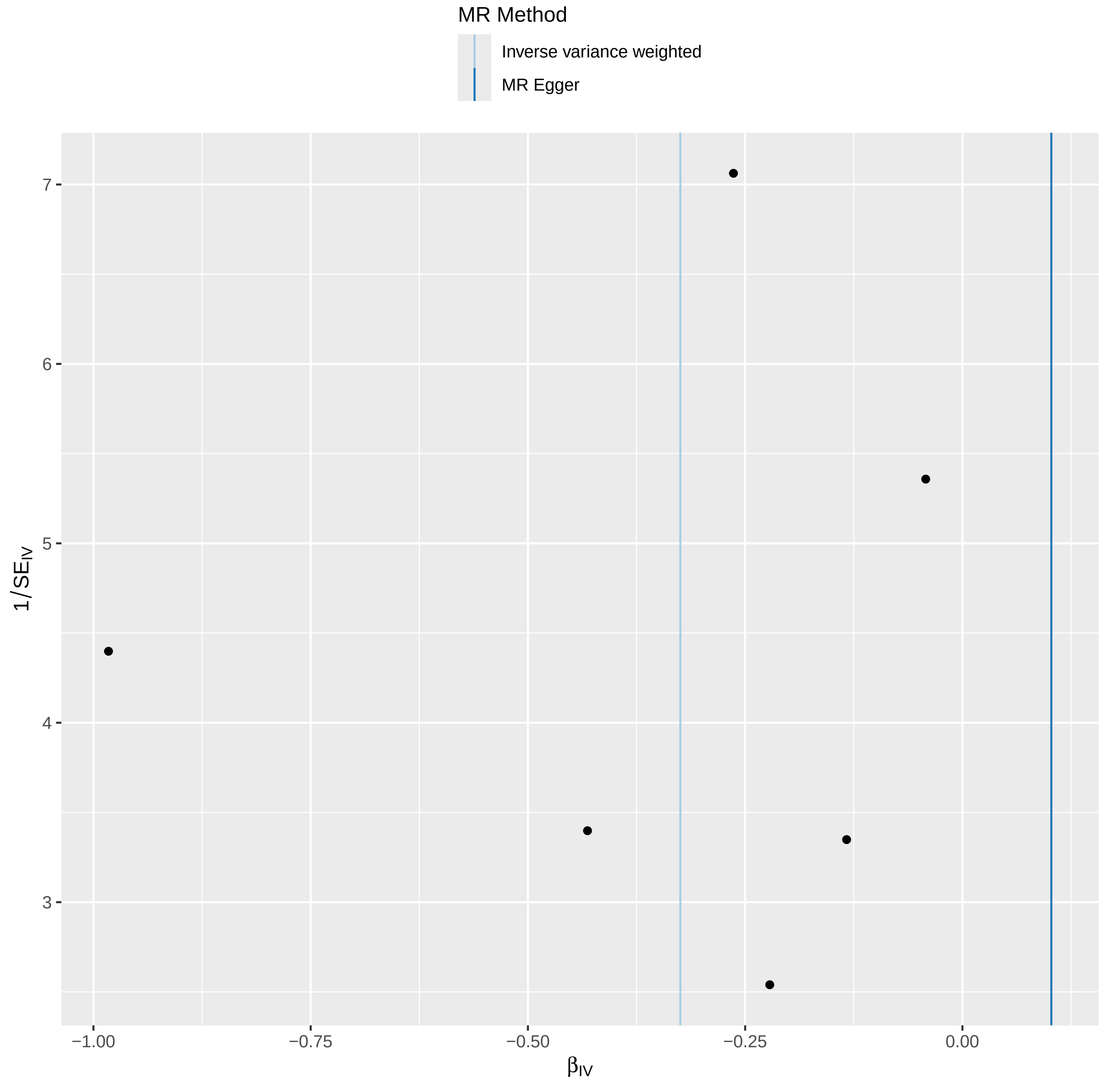

MR Method
Inverse variance weighted
MR Egger
7
6
5
4
3
1/SE$_{IV}$
−1.00
−0.75
−0.50
−0.25
0.00
β$_{IV}$

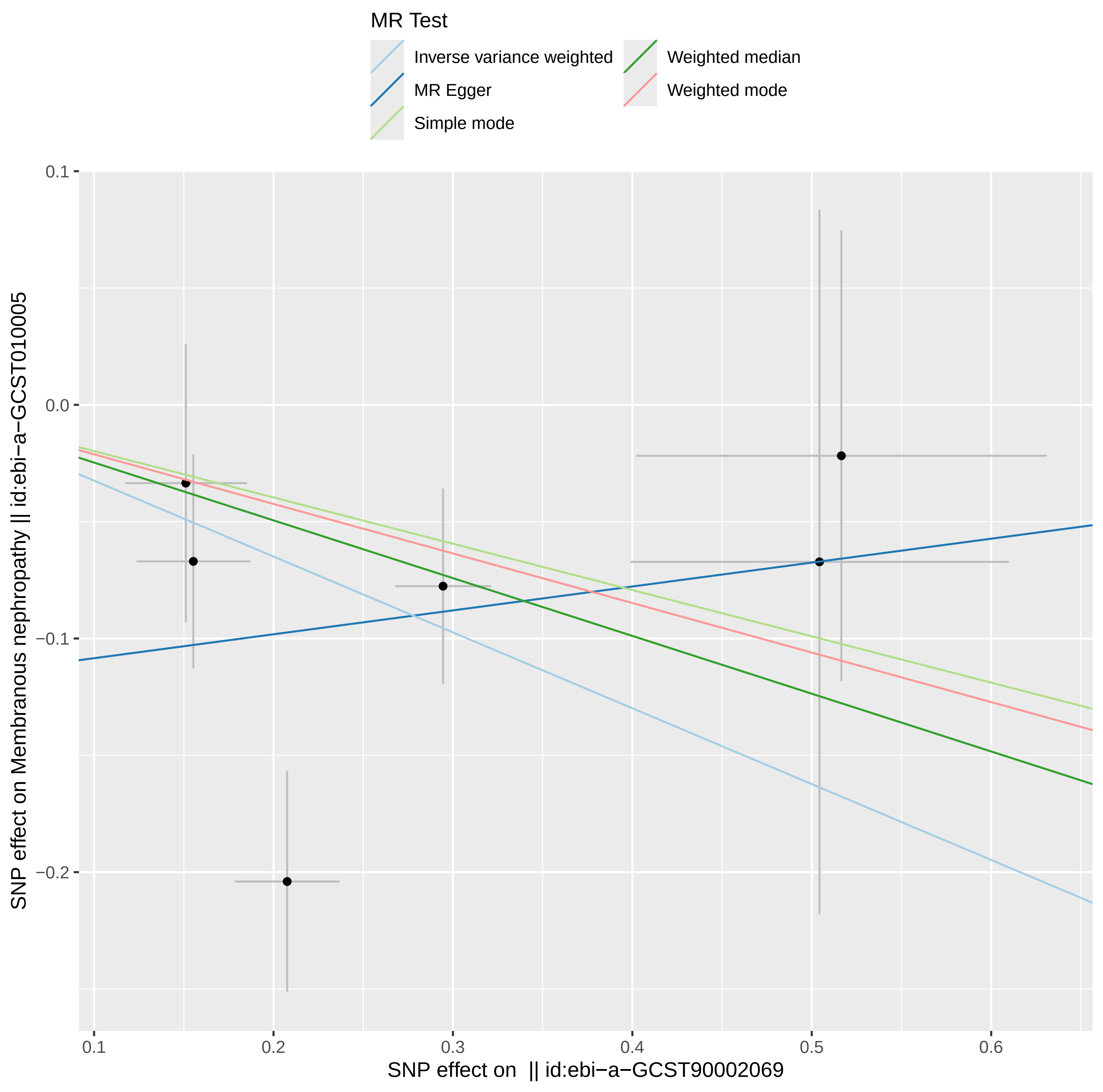

MR Test
Inverse variance weighted
MR Egger
Simple mode
Weighted median
Weighted mode
0.1
0.0
−0.1
−0.2
0.1
0.2
0.3
0.4
0.5
0.6
SNP effect on Membranous nephropathy || id:ebi−a−GCST010005
SNP effect on || id:ebi−a−GCST90002069

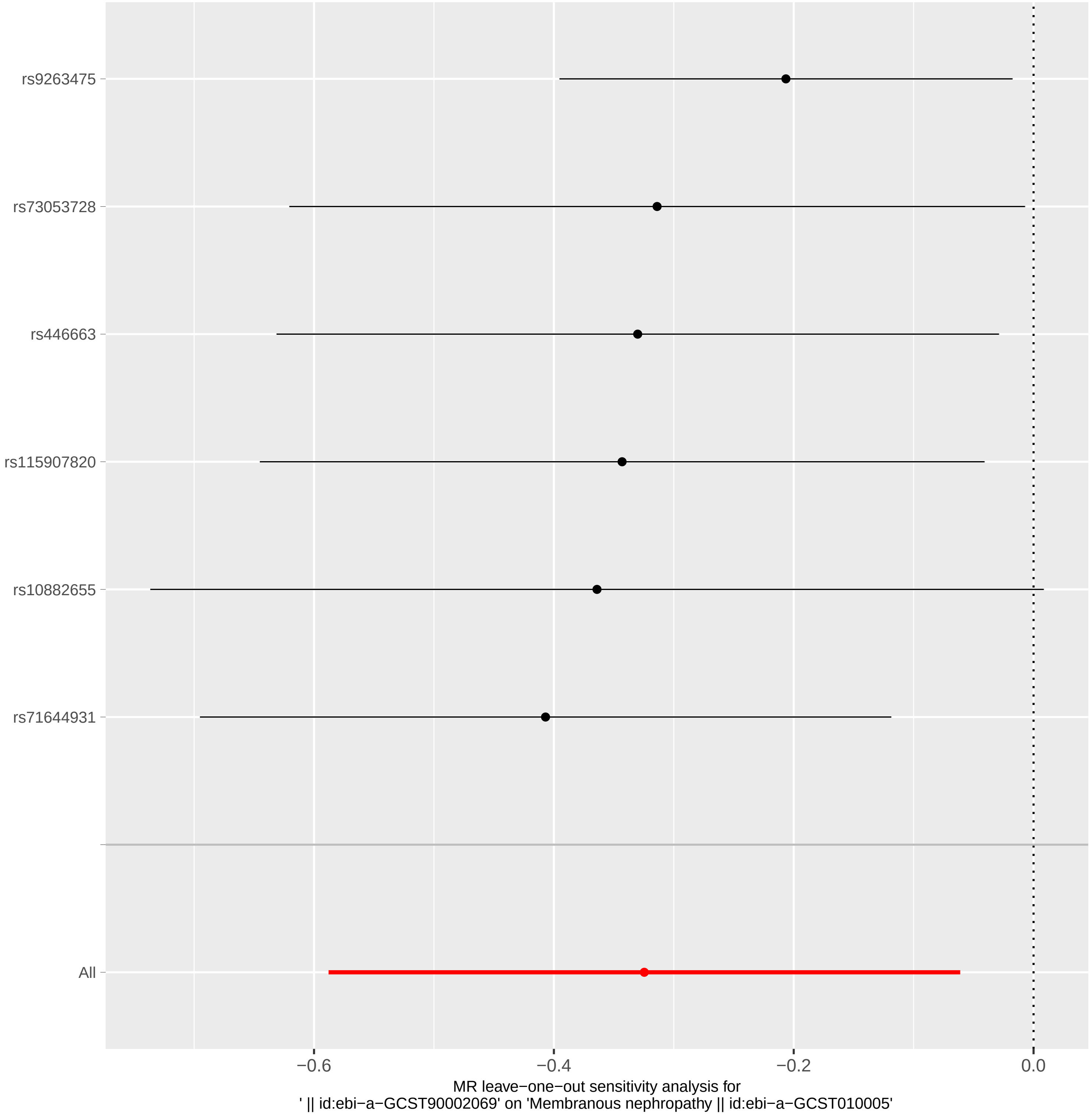

rs9263475
rs73053728
rs446663
rs115907820
rs10882655
rs71644931
All
−0.6
−0.4
−0.2
0.0
MR leave−one−out sensitivity analysis for
' || id:ebi−a−GCST90002069' on 'Membranous nephropathy || id:ebi−a−GCST010005'

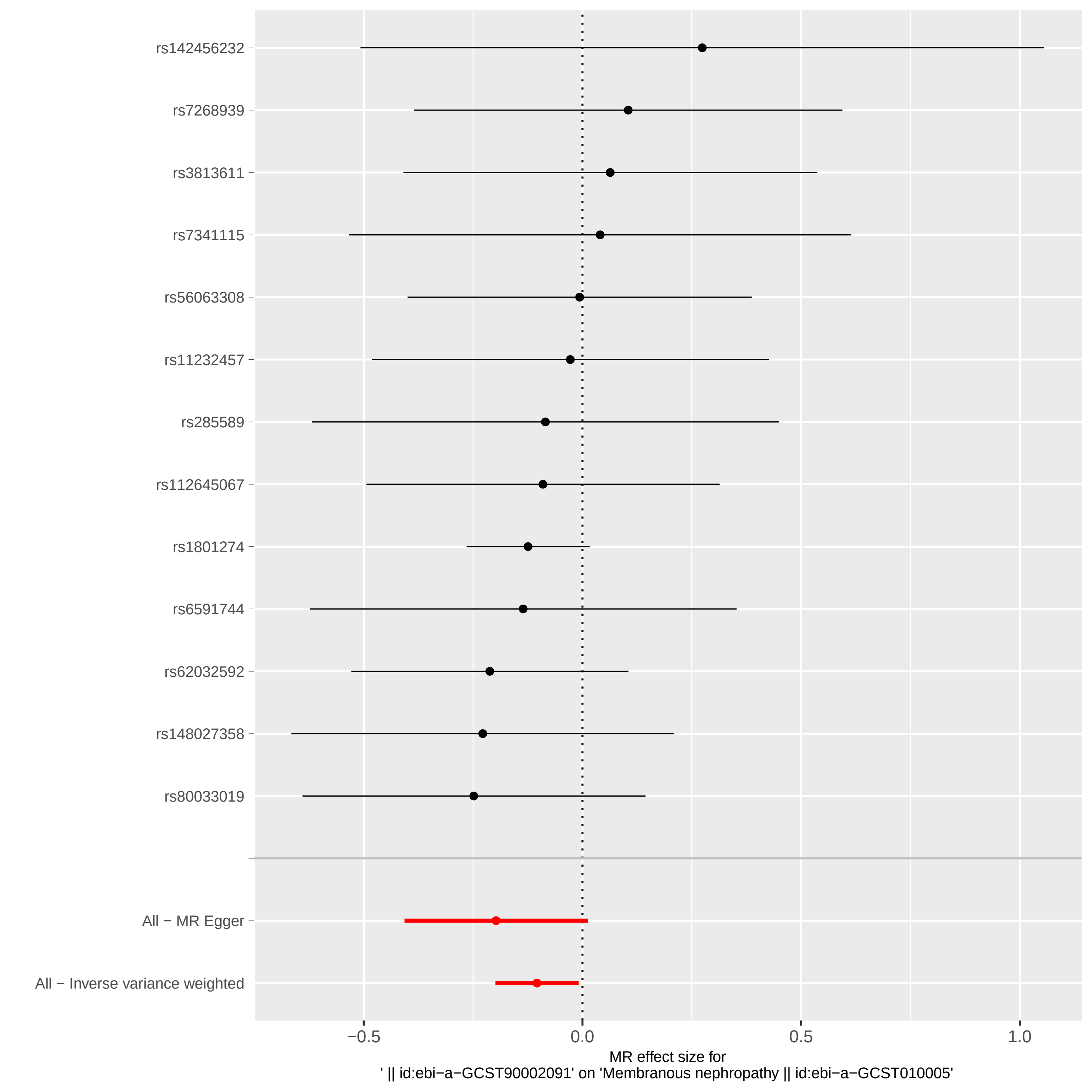
rs142456232
rs7268939
rs3813611
rs7341115
rs56063308
rs11232457
rs285589
rs112645067
rs1801274
rs6591744
rs62032592
rs148027358
rs80033019
All − MR Egger
All − Inverse variance weighted
−0.5
0.0
0.5
1.0
MR effect size for
' || id:ebi−a−GCST90002091' on 'Membranous nephropathy || id:ebi−a−GCST010005'

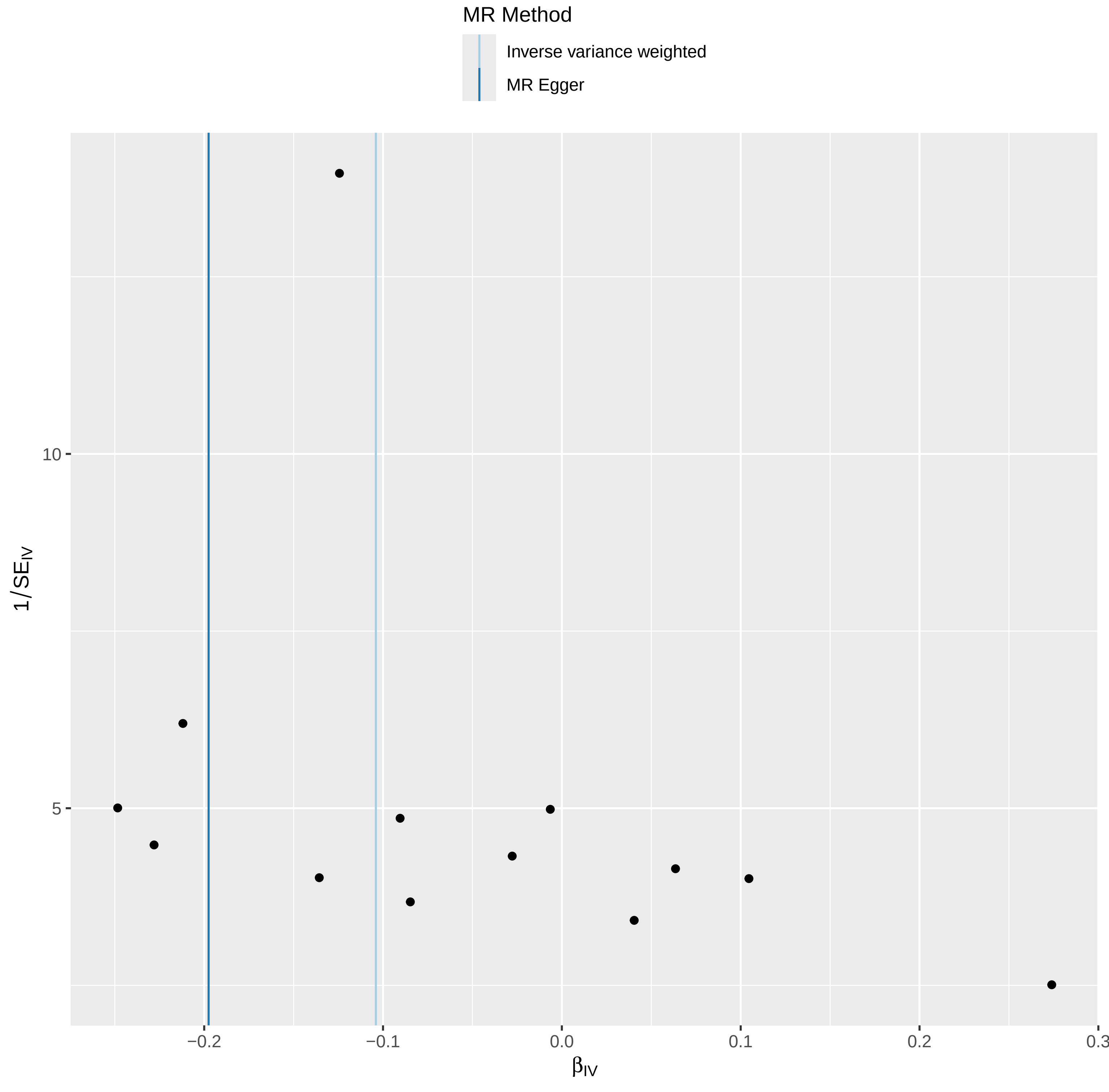

MR Method
Inverse variance weighted
MR Egger
10
5
1/SE_IV
−0.2
−0.1
0.0
0.1
0.2
0.3
β_IV

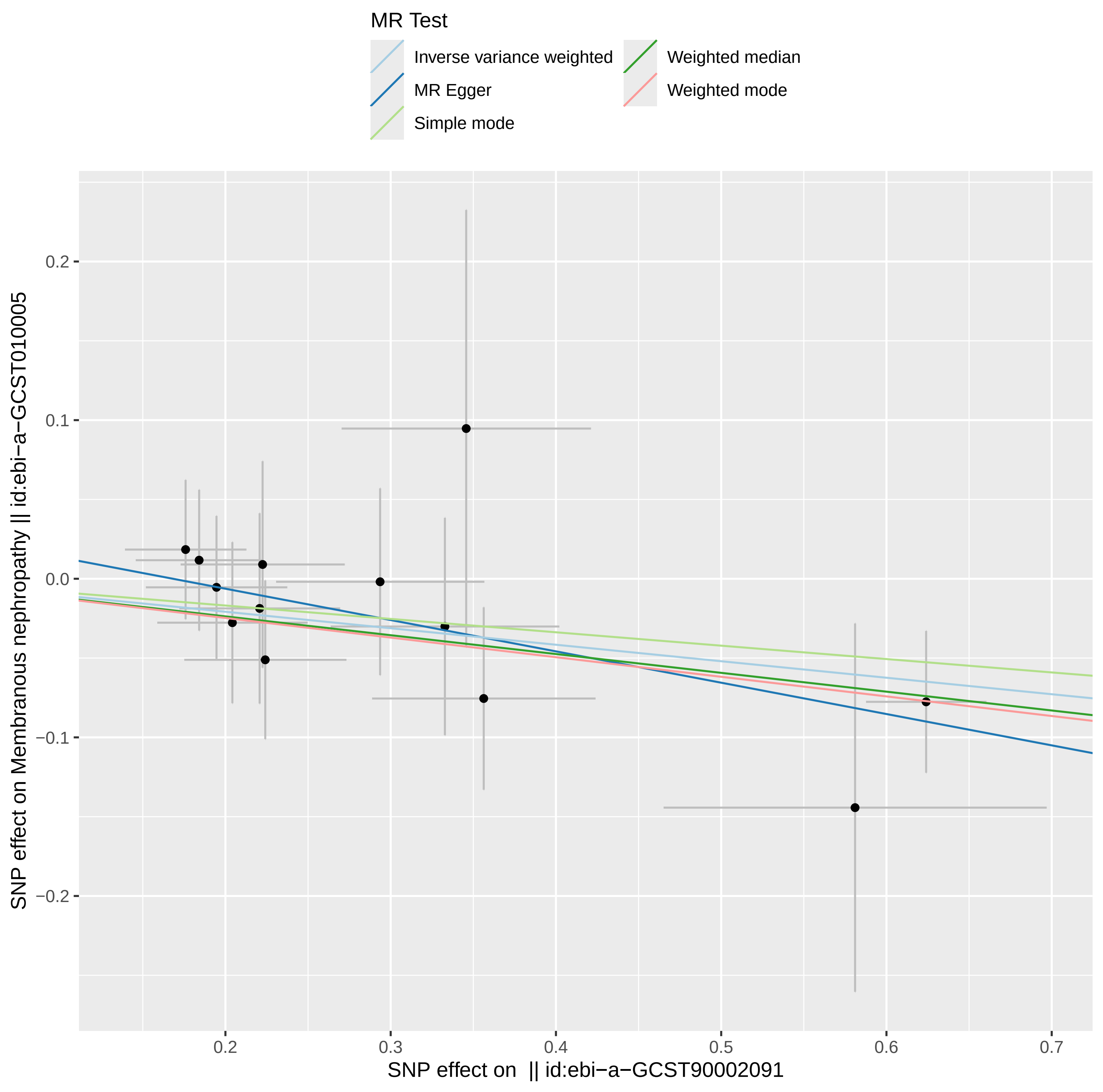

MR Test
Inverse variance weighted
MR Egger
Simple mode
Weighted median
Weighted mode
SNP effect on Membranous nephropathy || id:ebi−a−GCST010005
0.2
0.1
0.0
−0.1
−0.2
0.2
0.3
0.4
0.5
0.6
0.7
SNP effect on || id:ebi−a−GCST90002091

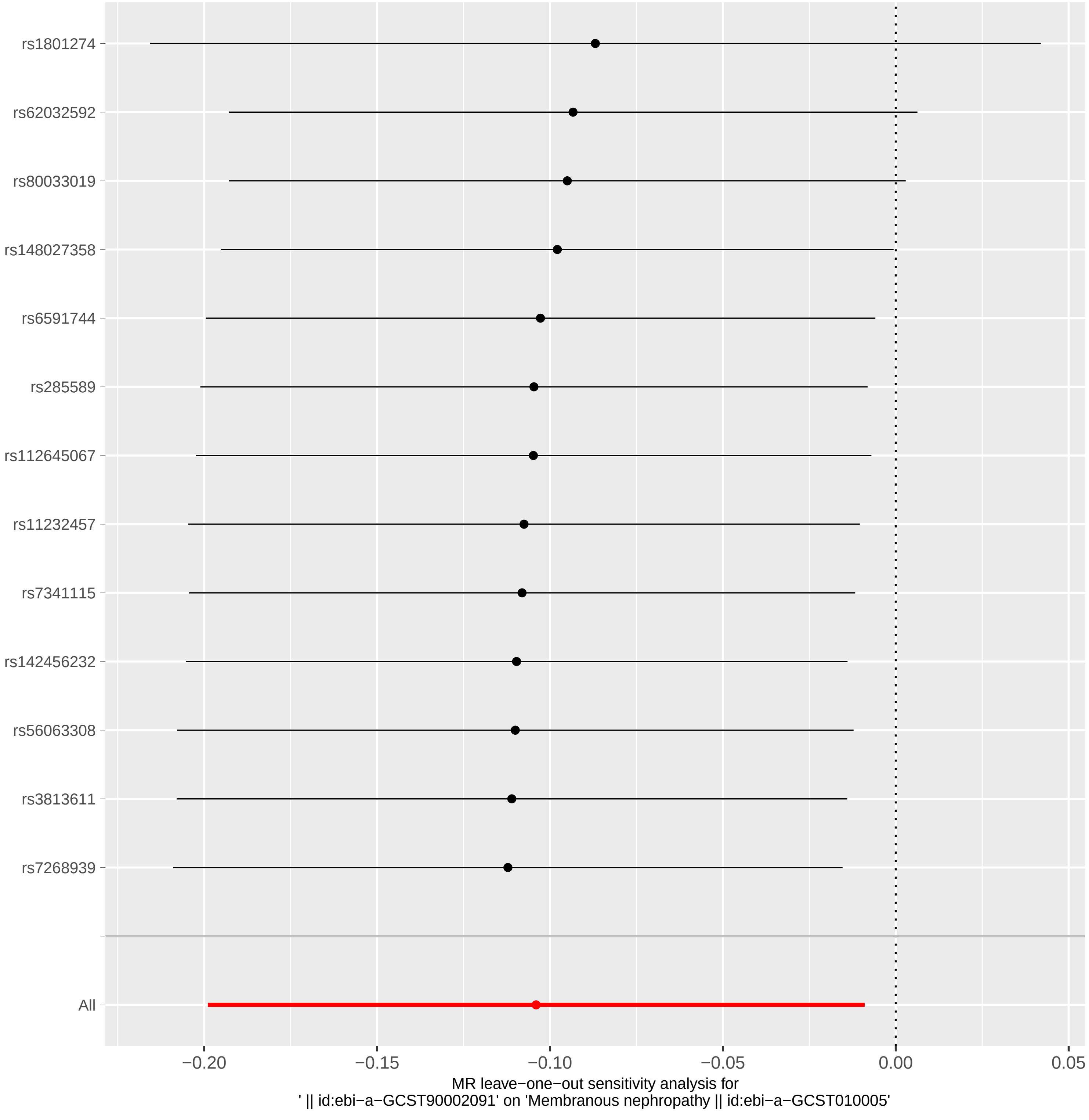
rs1801274
rs62032592
rs80033019
rs148027358
rs6591744
rs285589
rs112645067
rs11232457
rs7341115
rs142456232
rs56063308
rs3813611
rs7268939
All
−0.20
−0.15
−0.10
−0.05
0.00
0.05
MR leave−one−out sensitivity analysis for
' || id:ebi−a−GCST90002091' on 'Membranous nephropathy || id:ebi−a−GCST010005'

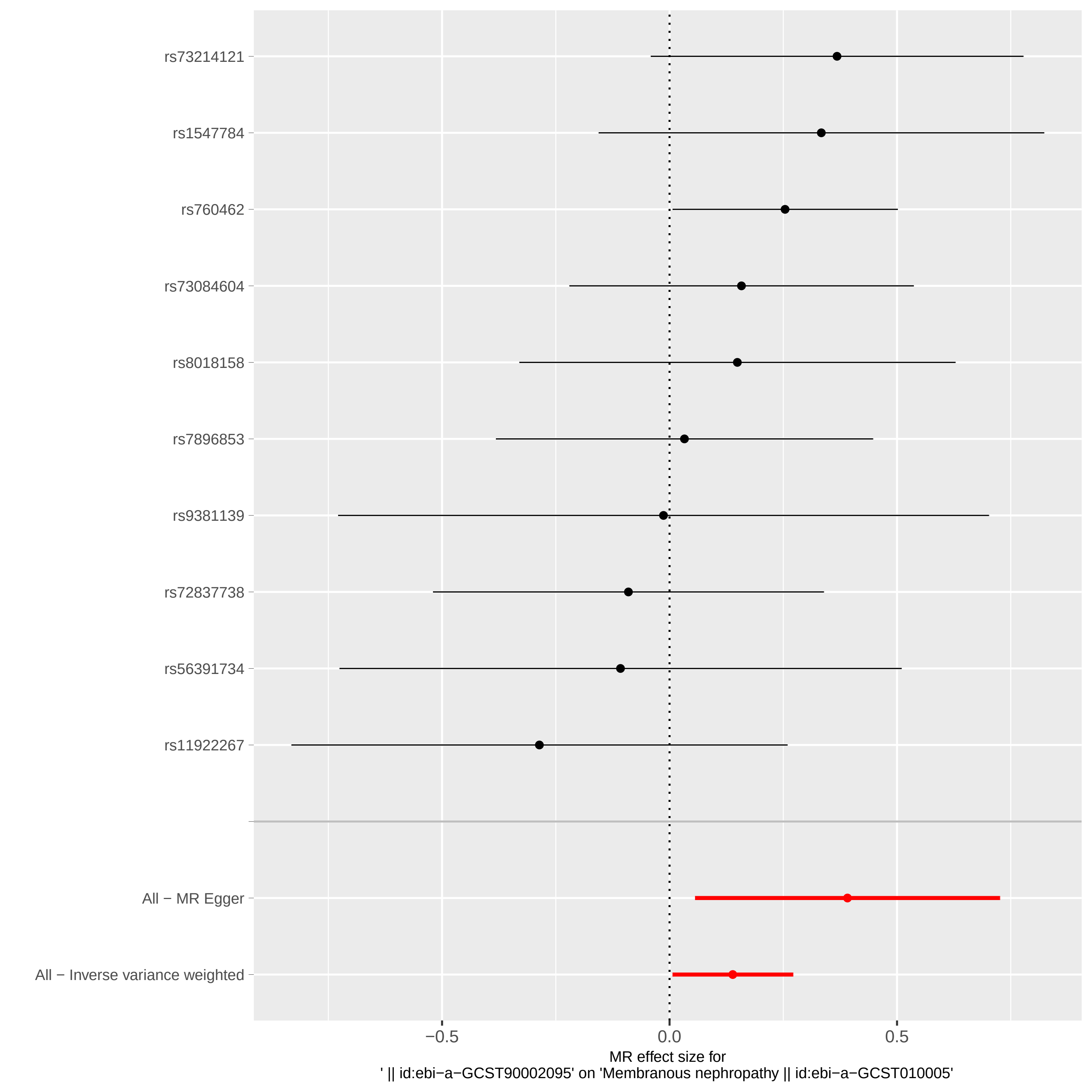

rs73214121
rs1547784
rs760462
rs73084604
rs8018158
rs7896853
rs9381139
rs72837738
rs56391734
rs11922267
All − MR Egger
All − Inverse variance weighted
−0.5
0.0
0.5
MR effect size for
' || id:ebi−a−GCST90002095' on 'Membranous nephropathy || id:ebi−a−GCST010005'

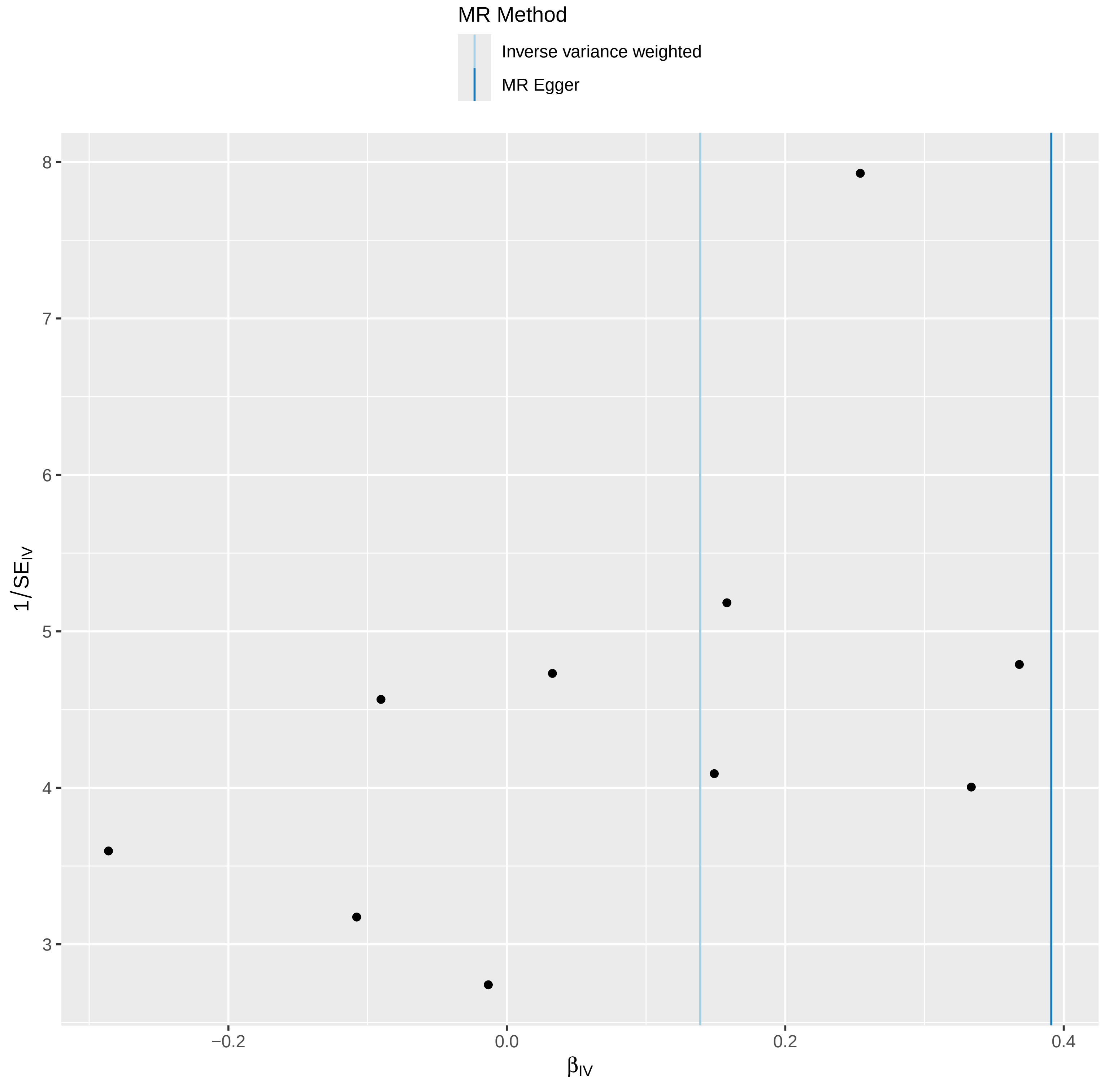
MR Method
Inverse variance weighted
MR Egger
8
7
6
5
4
3
1/SE_IV
−0.2
0.0
0.2
0.4
β_IV

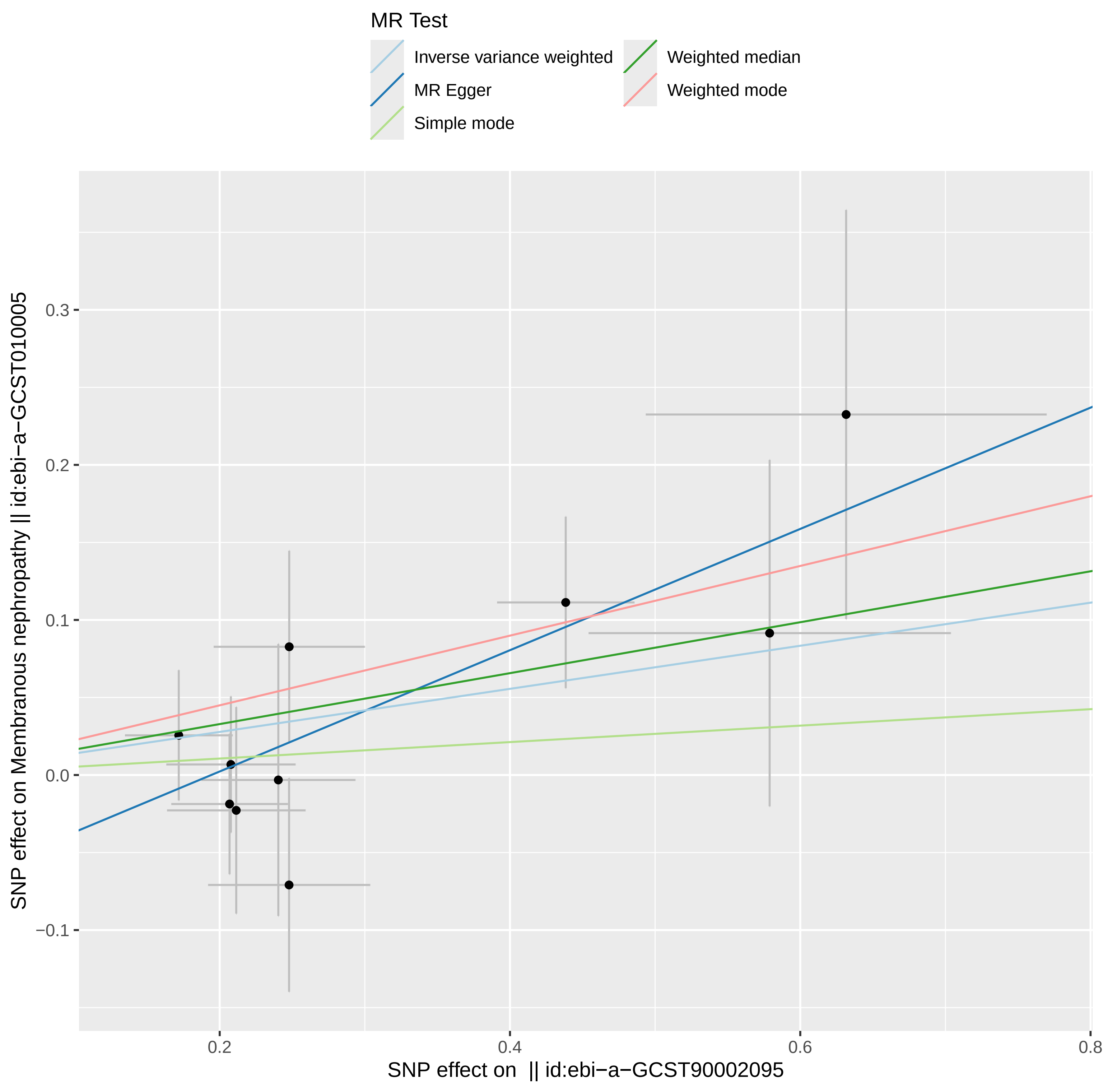
MR Test
Inverse variance weighted
MR Egger
Simple mode
Weighted median
Weighted mode
0.3
0.2
0.1
0.0
−0.1
0.2
0.4
0.6
0.8
SNP effect on Membranous nephropathy || id:ebi−a−GCST010005
SNP effect on || id:ebi−a−GCST90002095

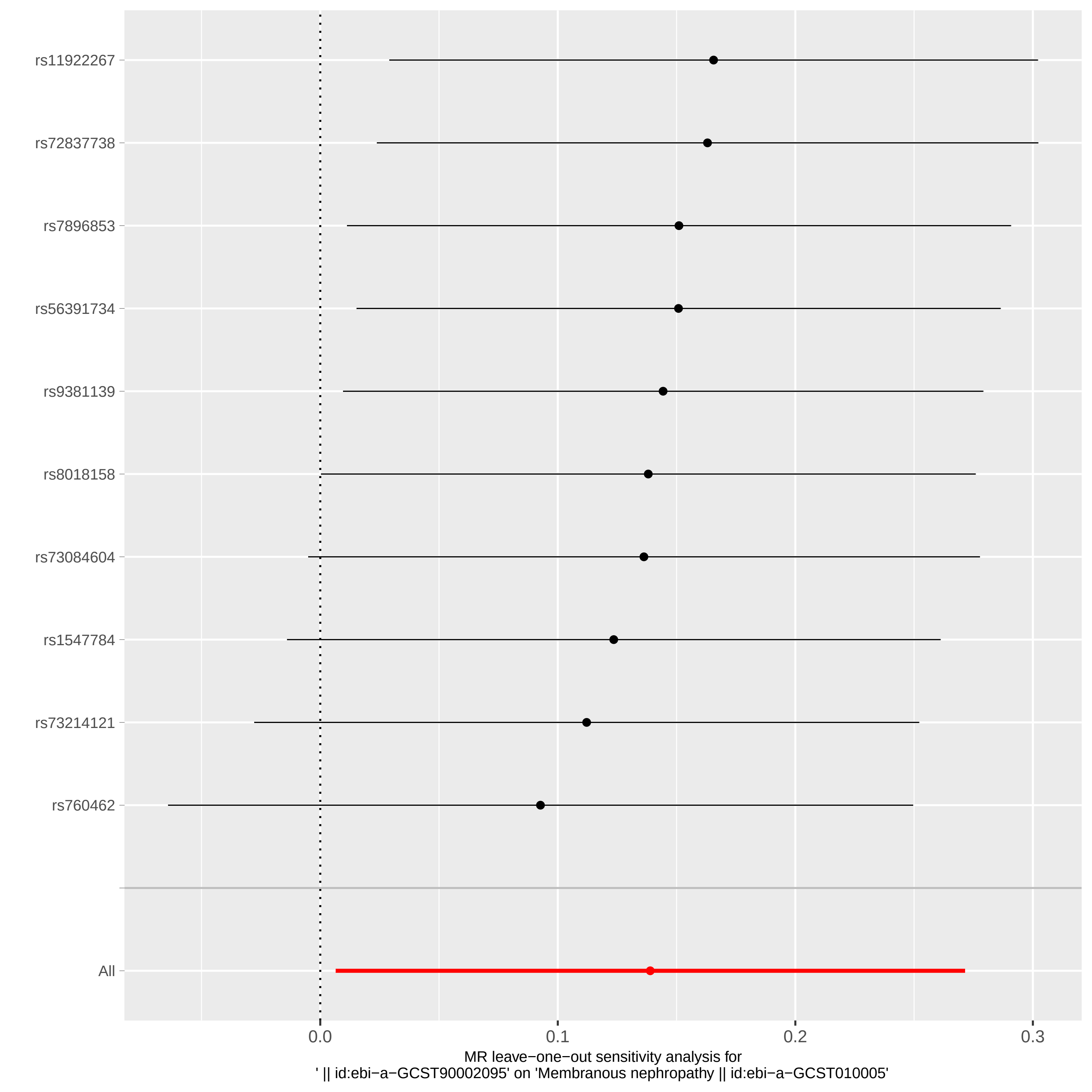

rs11922267
rs72837738
rs7896853
rs56391734
rs9381139
rs8018158
rs73084604
rs1547784
rs73214121
rs760462
All
0.0
0.1
0.2
0.3
MR leave−one−out sensitivity analysis for
' || id:ebi−a−GCST90002095' on 'Membranous nephropathy || id:ebi−a−GCST010005'

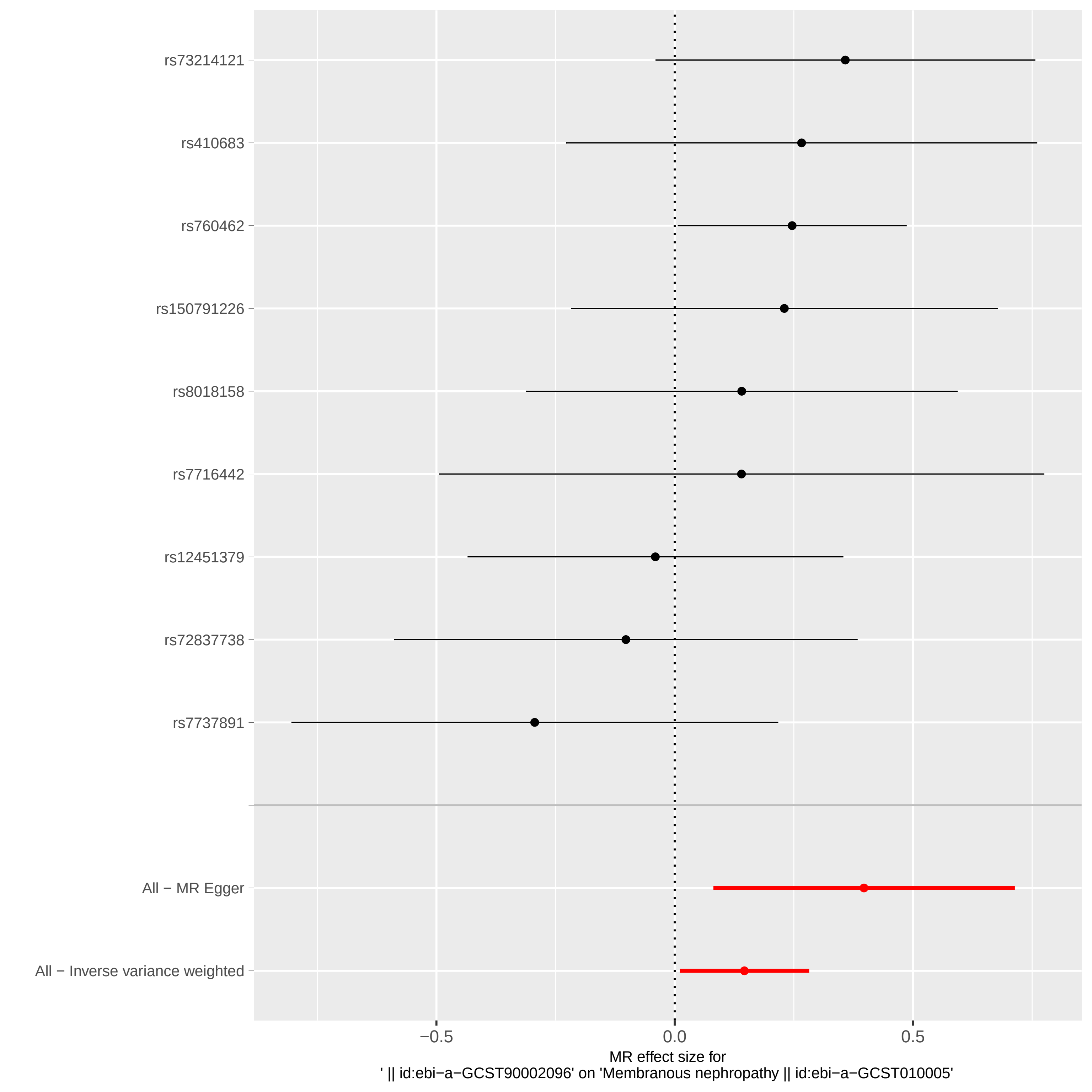
rs73214121
rs410683
rs760462
rs150791226
rs8018158
rs7716442
rs12451379
rs72837738
rs7737891
All − MR Egger
All − Inverse variance weighted
−0.5
0.0
0.5
MR effect size for
' || id:ebi−a−GCST90002096' on 'Membranous nephropathy || id:ebi−a−GCST010005'

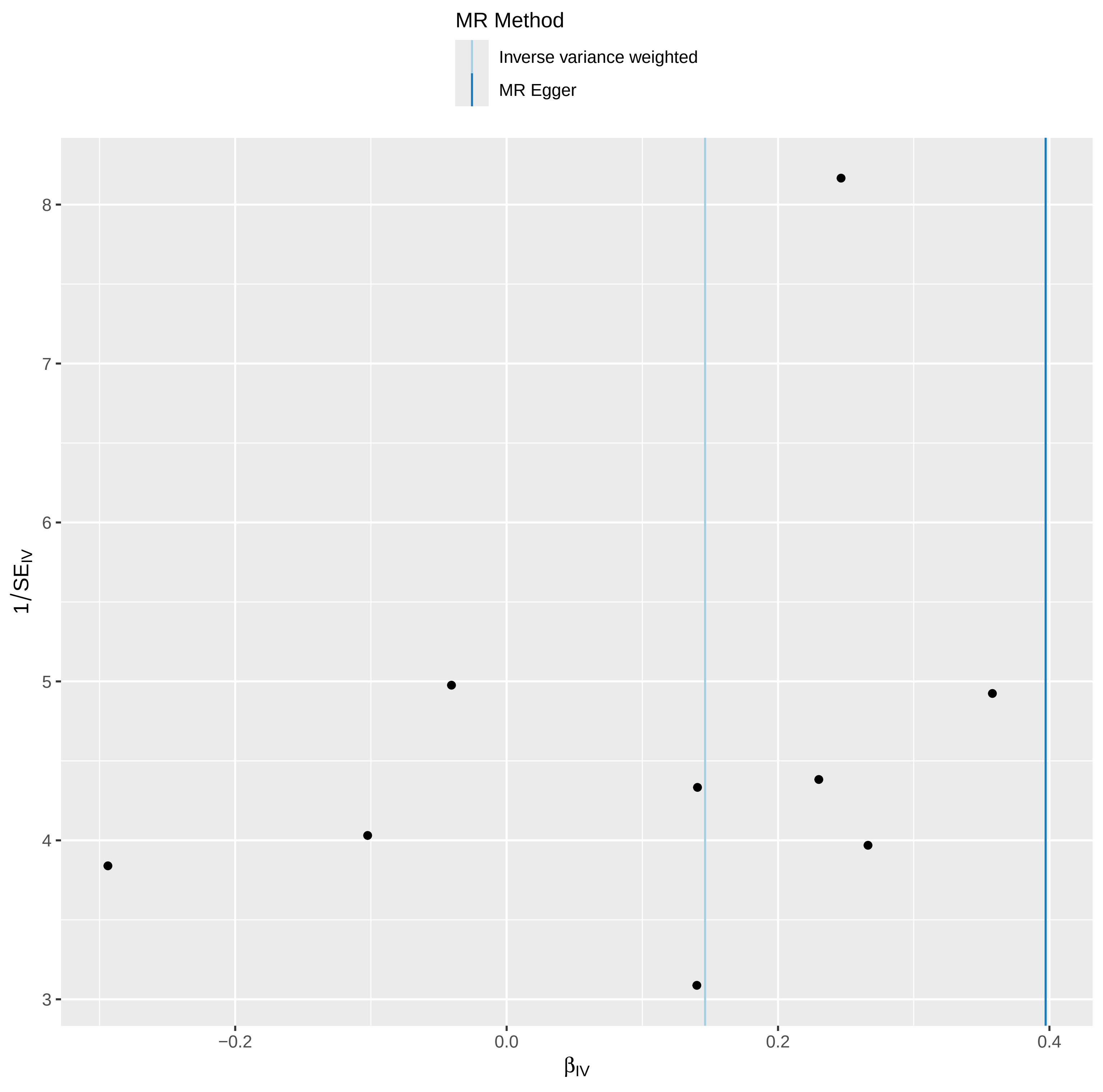
MR Method
Inverse variance weighted
MR Egger
8
7
6
5
4
3
1/SE_IV
−0.2
0.0
0.2
0.4
β_IV

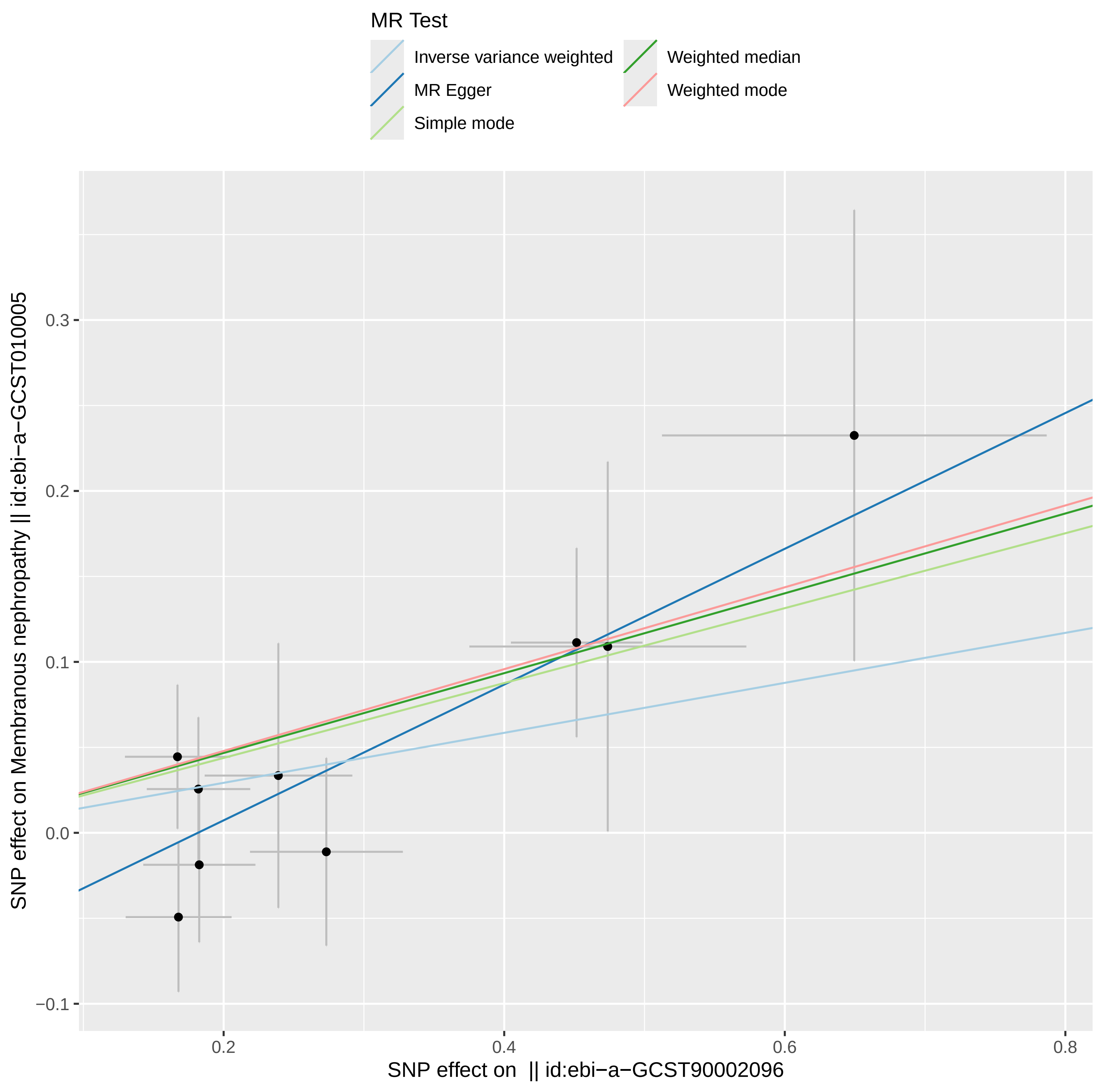
MR Test
Inverse variance weighted
MR Egger
Simple mode
Weighted median
Weighted mode
SNP effect on Membranous nephropathy || id:ebi−a−GCST010005
0.3
0.2
0.1
0.0
−0.1
0.2
0.4
0.6
0.8
SNP effect on || id:ebi−a−GCST90002096

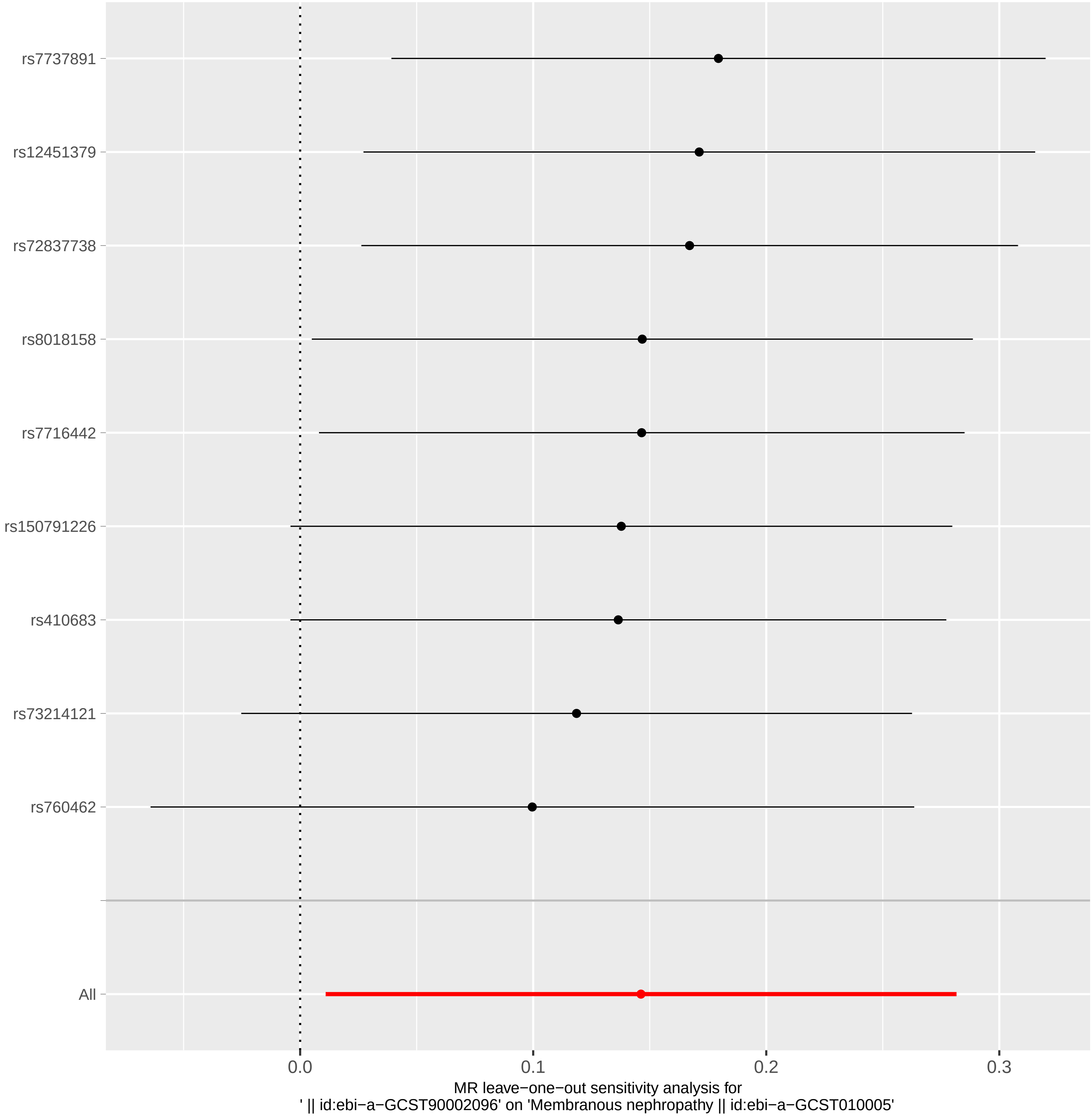
rs7737891
rs12451379
rs72837738
rs8018158
rs7716442
rs150791226
rs410683
rs73214121
rs760462
All
0.0
0.1
0.2
0.3
MR leave−one−out sensitivity analysis for
' || id:ebi−a−GCST90002096' on 'Membranous nephropathy || id:ebi−a−GCST010005'

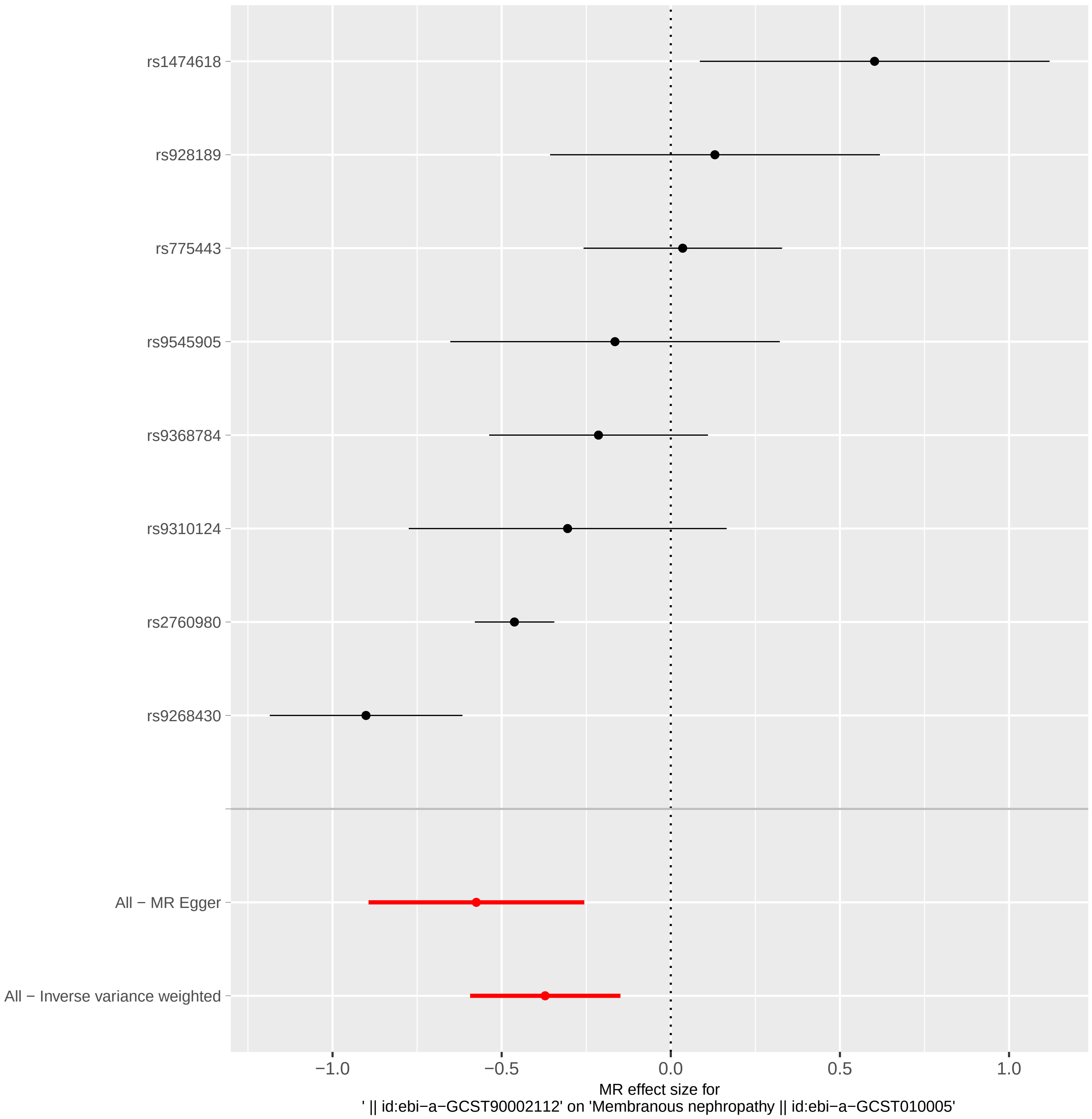

rs1474618
rs928189
rs775443
rs9545905
rs9368784
rs9310124
rs2760980
rs9268430
All − MR Egger
All − Inverse variance weighted
−1.0
−0.5
0.0
0.5
1.0
MR effect size for
' || id:ebi−a−GCST90002112' on 'Membranous nephropathy || id:ebi−a−GCST010005'

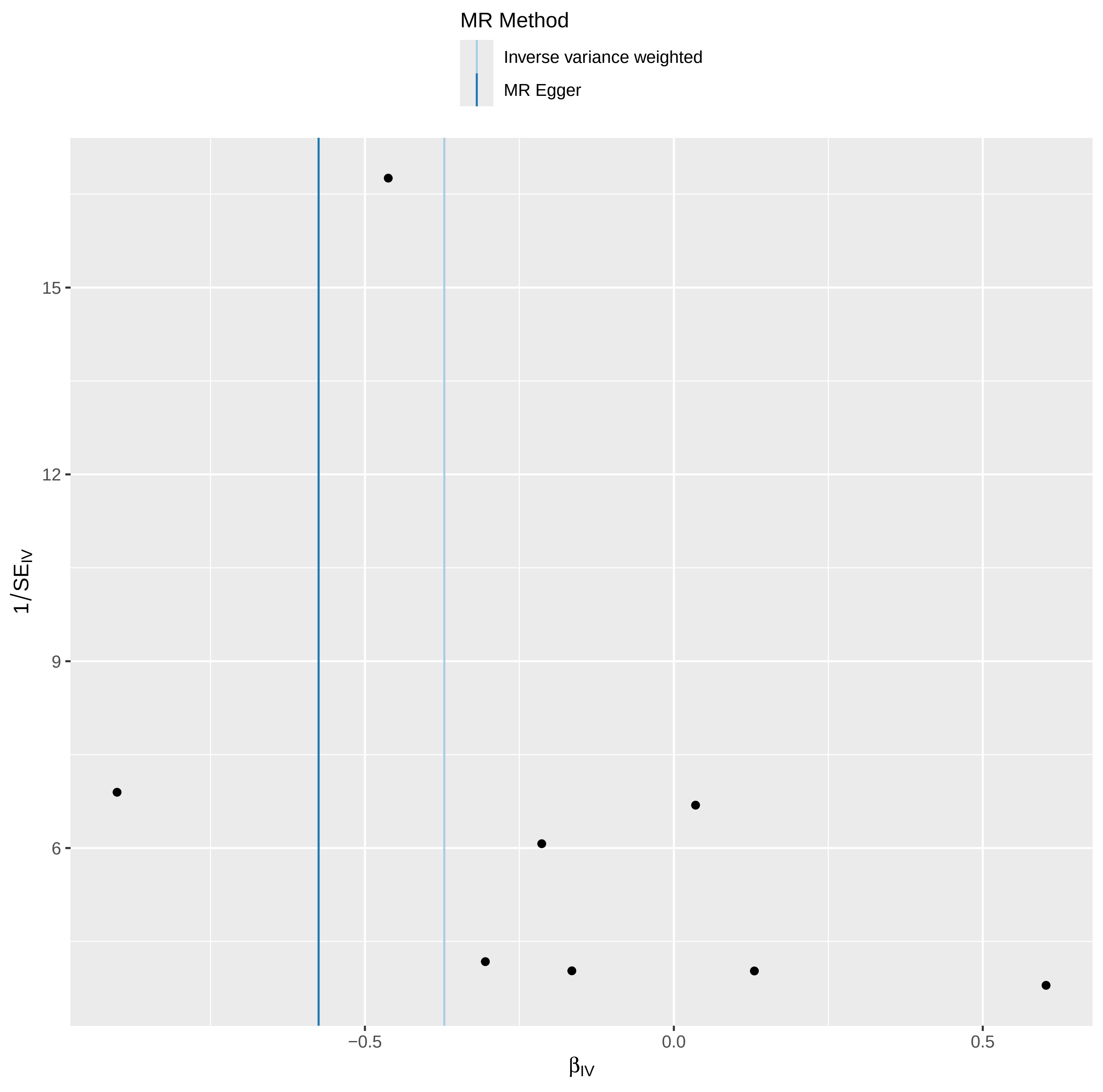

MR Method
Inverse variance weighted
MR Egger
15
12
9
6
1/SE_IV
−0.5
0.0
0.5
β_IV

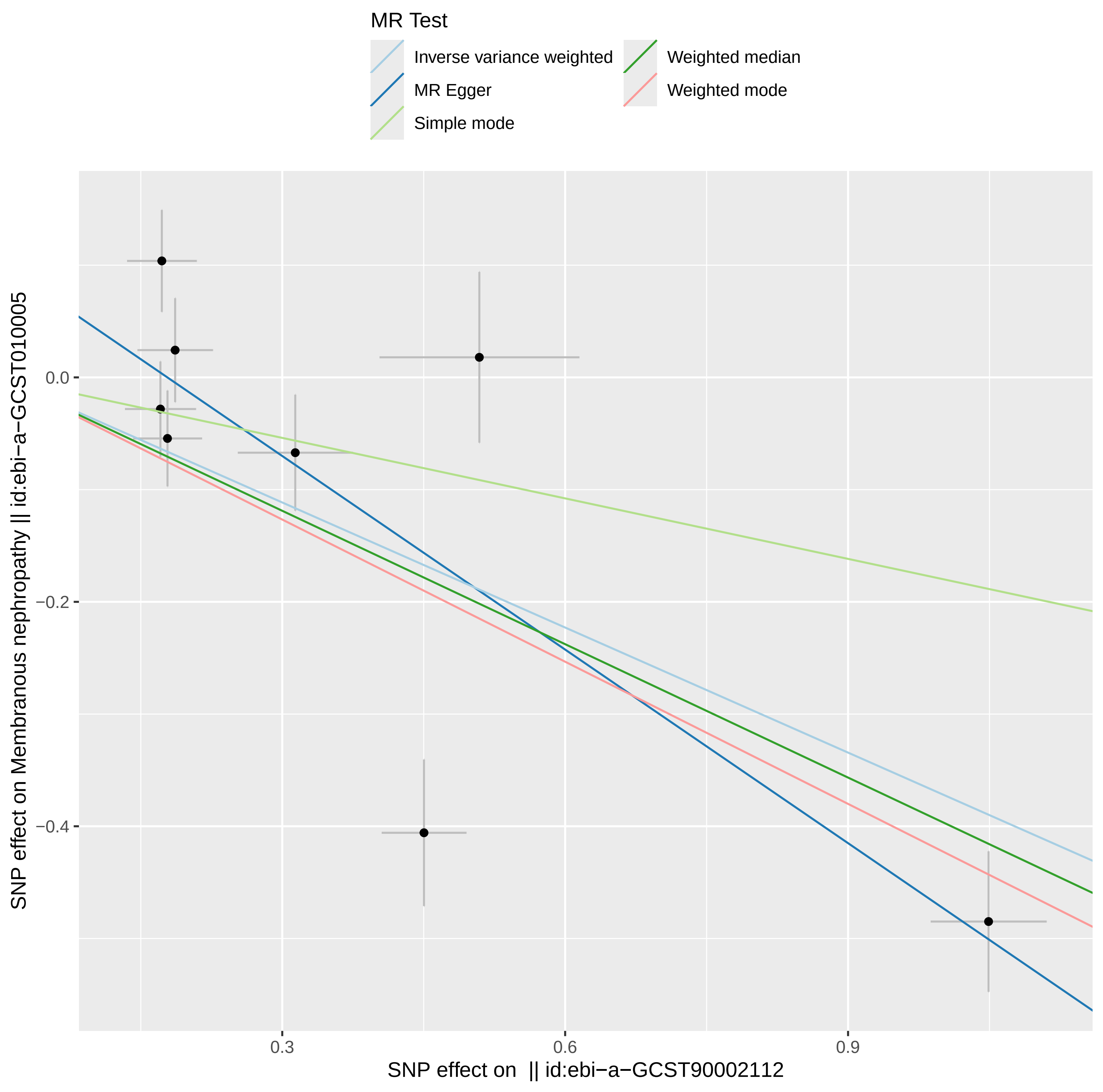

MR Test
Inverse variance weighted
MR Egger
Simple mode
Weighted median
Weighted mode
0.0
−0.2
−0.4
0.3
0.6
0.9
SNP effect on Membranous nephropathy || id:ebi−a−GCST010005
SNP effect on || id:ebi−a−GCST90002112

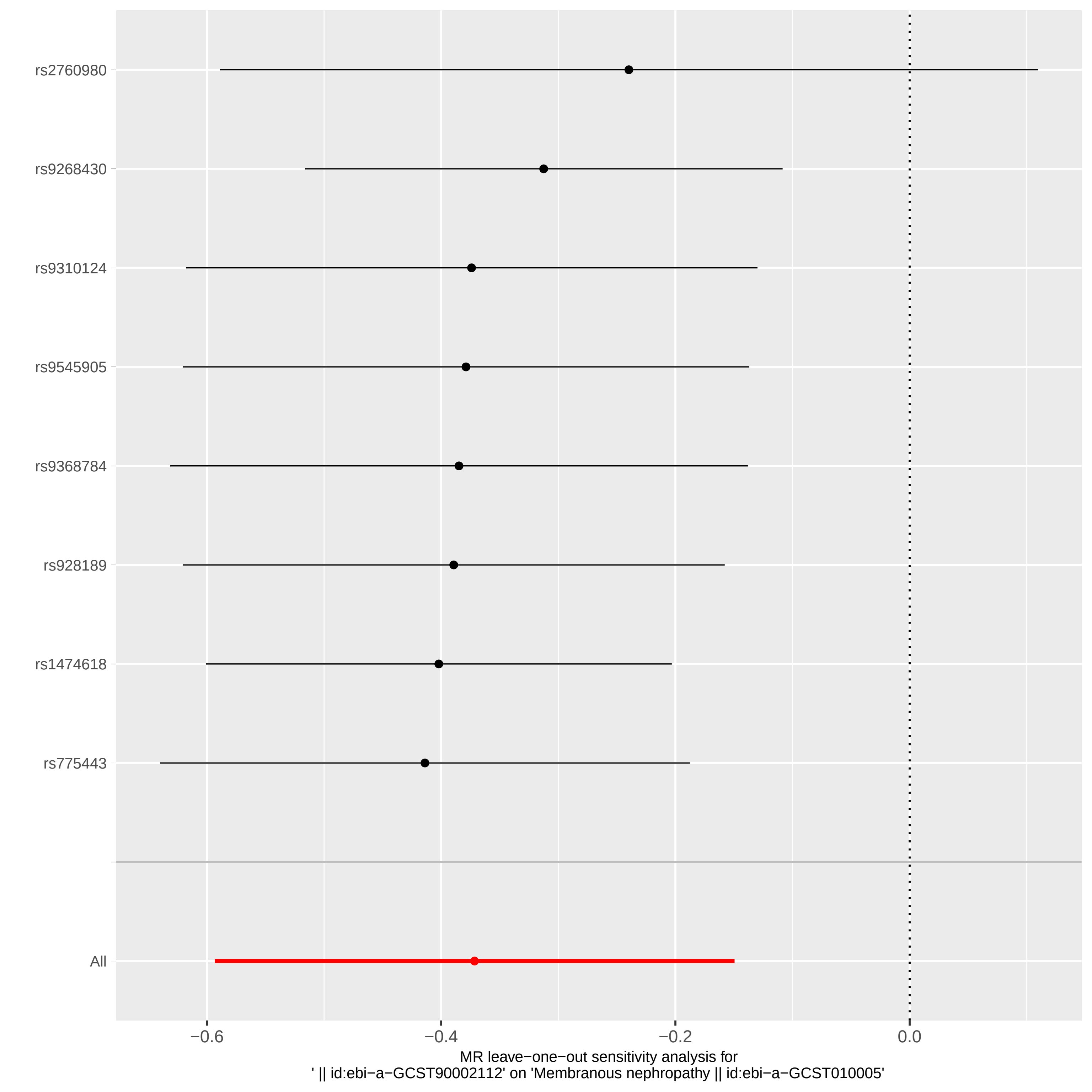

rs2760980
rs9268430
rs9310124
rs9545905
rs9368784
rs928189
rs1474618
rs775443
All
−0.6
−0.4
−0.2
0.0
MR leave−one−out sensitivity analysis for
' || id:ebi−a−GCST90002112' on 'Membranous nephropathy || id:ebi−a−GCST010005'

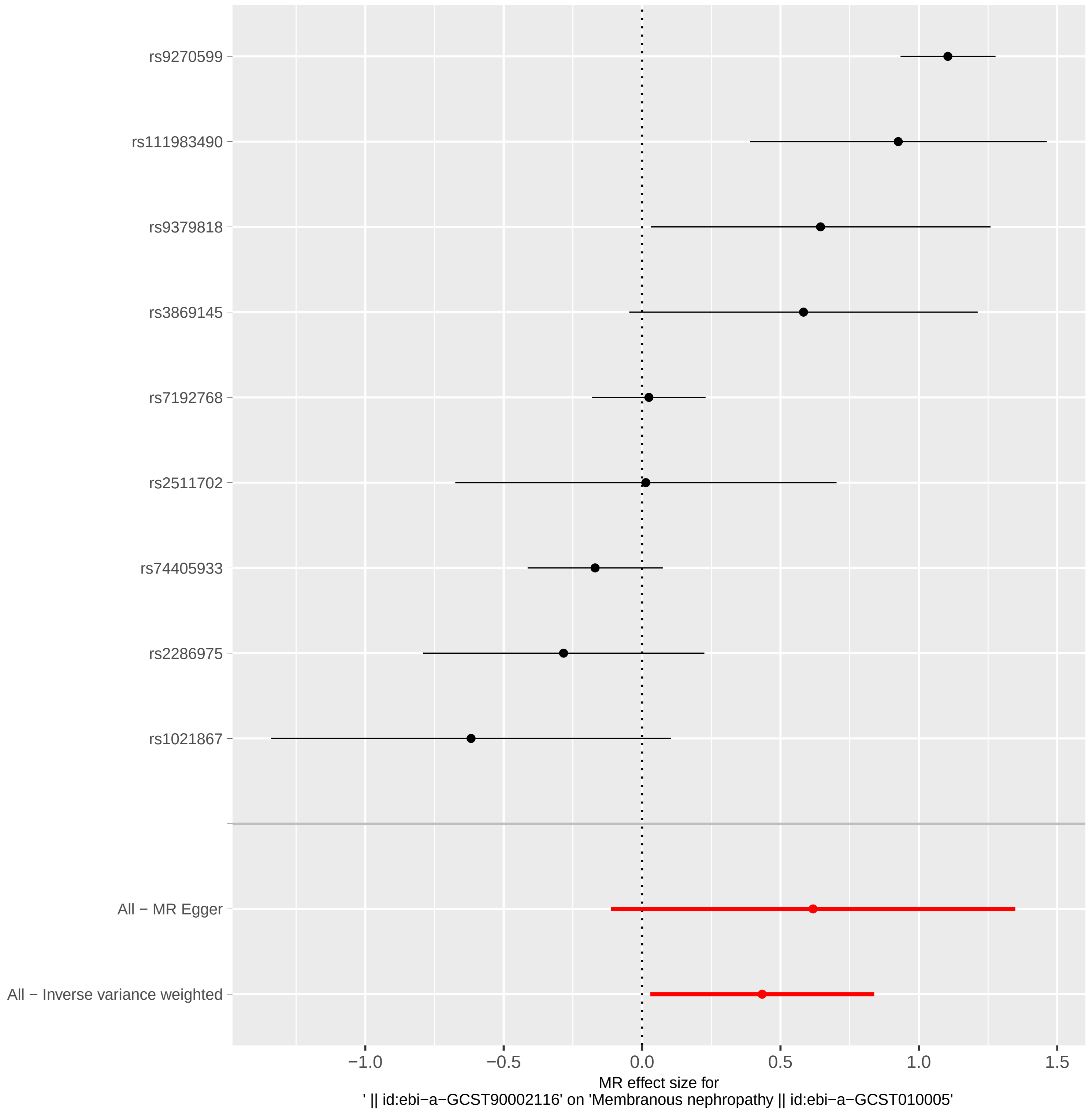
rs9270599
rs111983490
rs9379818
rs3869145
rs7192768
rs2511702
rs74405933
rs2286975
rs1021867
All − MR Egger
All − Inverse variance weighted
−1.0
−0.5
0.0
0.5
1.0
1.5
MR effect size for
' || id:ebi−a−GCST90002116' on 'Membranous nephropathy || id:ebi−a−GCST010005'

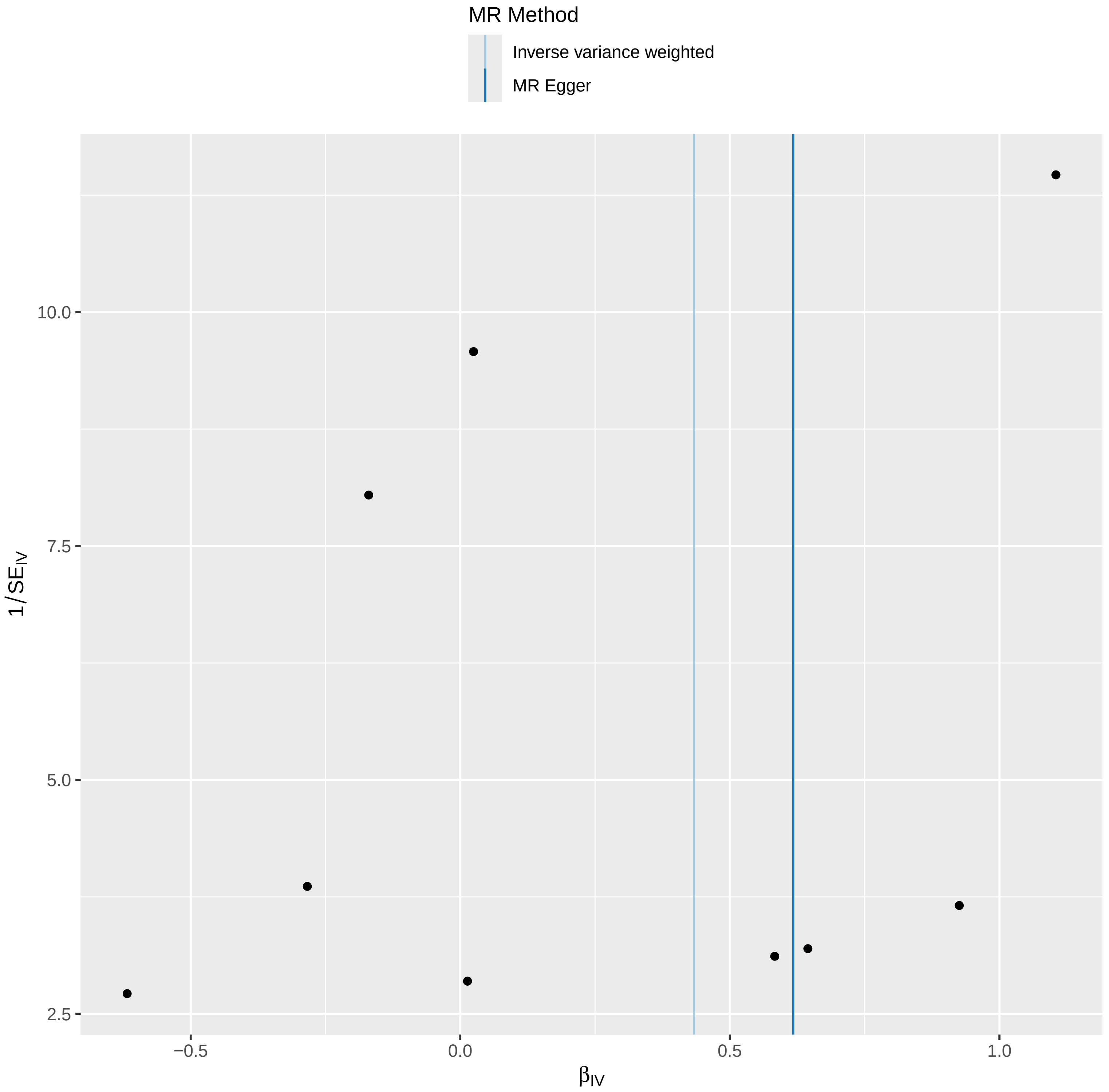

MR Method
Inverse variance weighted
MR Egger
10.0
7.5
5.0
2.5
1/SE_IV
−0.5
0.0
0.5
1.0
β_IV

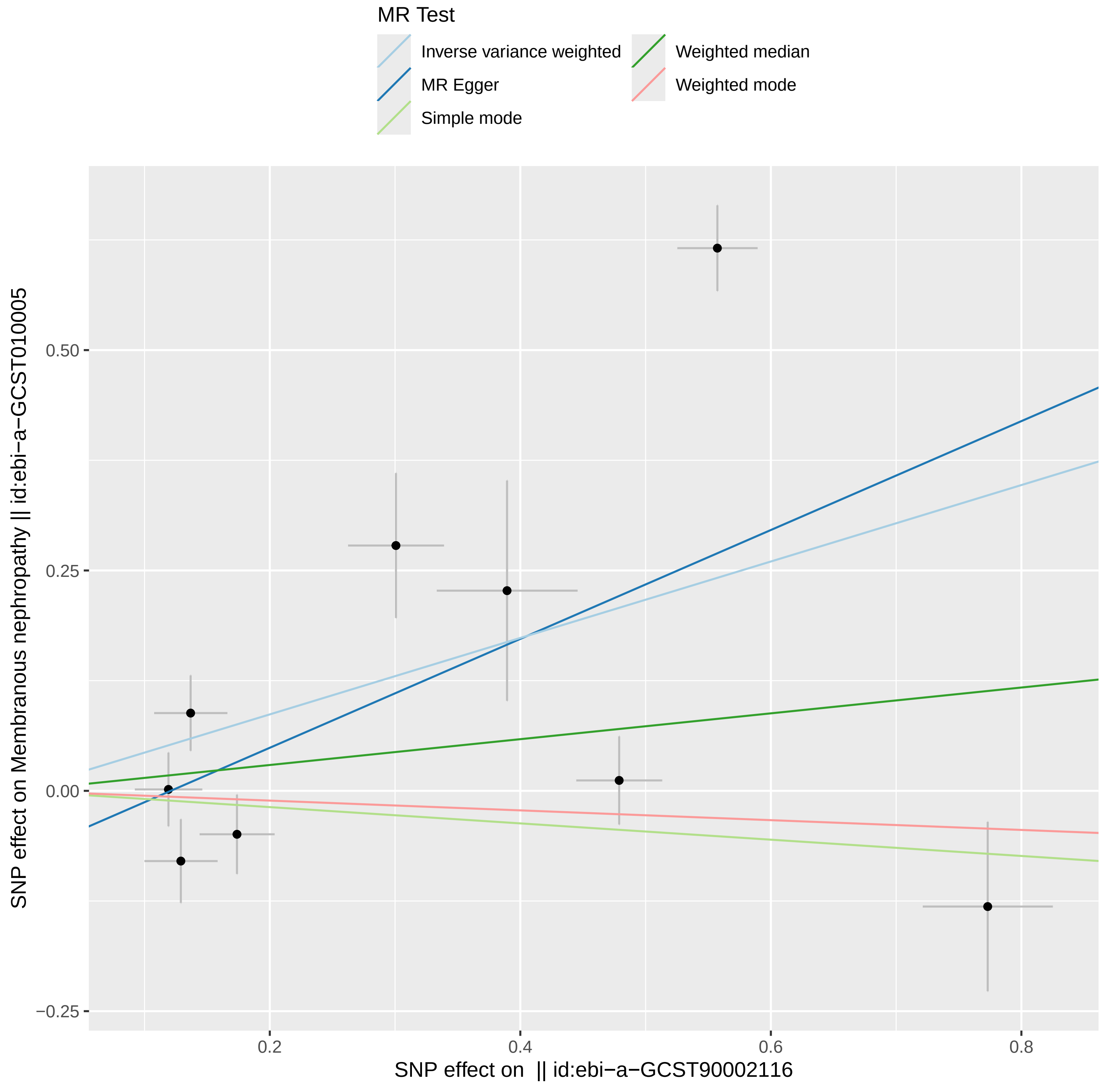
MR Test
Inverse variance weighted
MR Egger
Simple mode
Weighted median
Weighted mode
SNP effect on Membranous nephropathy || id:ebi−a−GCST010005
0.50
0.25
0.00
−0.25
0.2
0.4
0.6
0.8
SNP effect on || id:ebi−a−GCST90002116

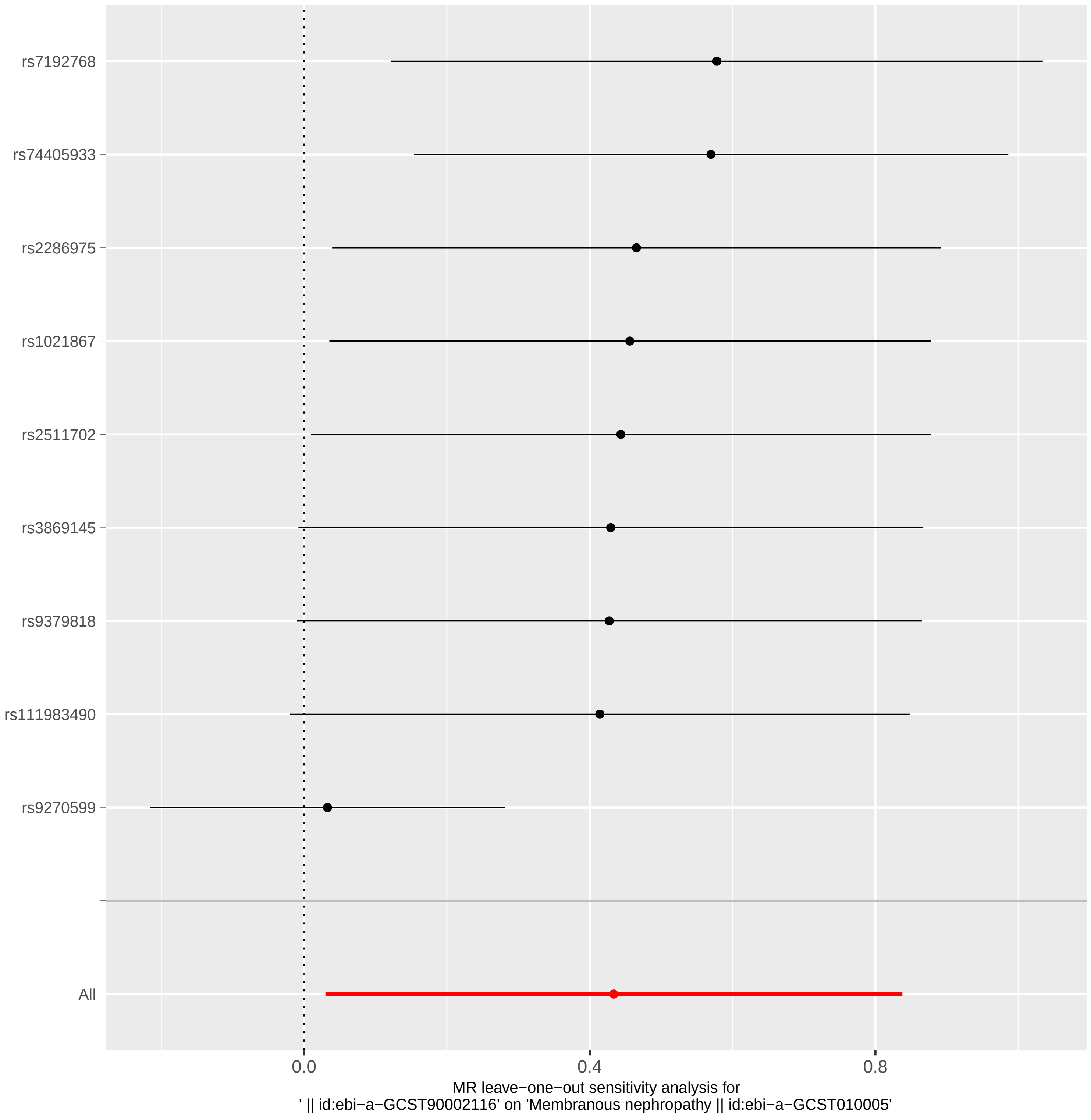
rs7192768
rs74405933
rs2286975
rs1021867
rs2511702
rs3869145
rs9379818
rs111983490
rs9270599
All
0.0
0.4
0.8
MR leave−one−out sensitivity analysis for
' || id:ebi−a−GCST90002116' on 'Membranous nephropathy || id:ebi−a−GCST010005'
