# Supplementary material for: Association between immune cell subtypes and membranous nephropathy: A bidirectional Mendelian randomization study
Source: Medicine (Baltimore). 2025 Jun 6;104(23):e42774. doi: 10.1097/MD.0000000000042774 (PMC12151028; doi:10.1097/MD.0000000000042774)
Supplement: Supplementary file 4 [file medi-104-e42774-s004.pdf]

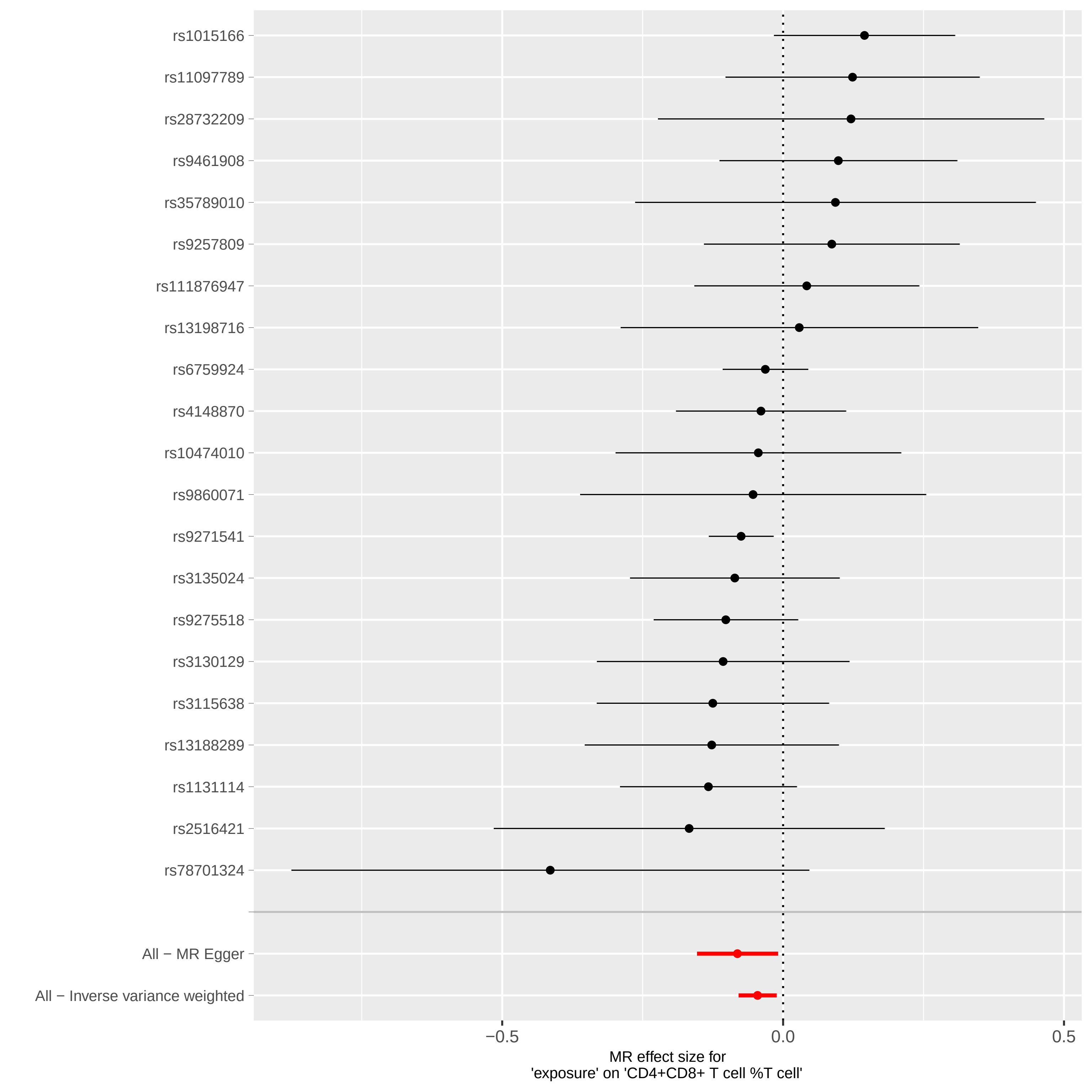

rs1015166
rs11097789
rs28732209
rs9461908
rs35789010
rs9257809
rs111876947
rs13198716
rs6759924
rs4148870
rs10474010
rs9860071
rs9271541
rs3135024
rs9275518
rs3130129
rs3115638
rs13188289
rs1131114
rs2516421
rs78701324
All − MR Egger
All − Inverse variance weighted
−0.5
0.0
0.5
MR effect size for
'exposure' on 'CD4+CD8+ T cell %T cell'

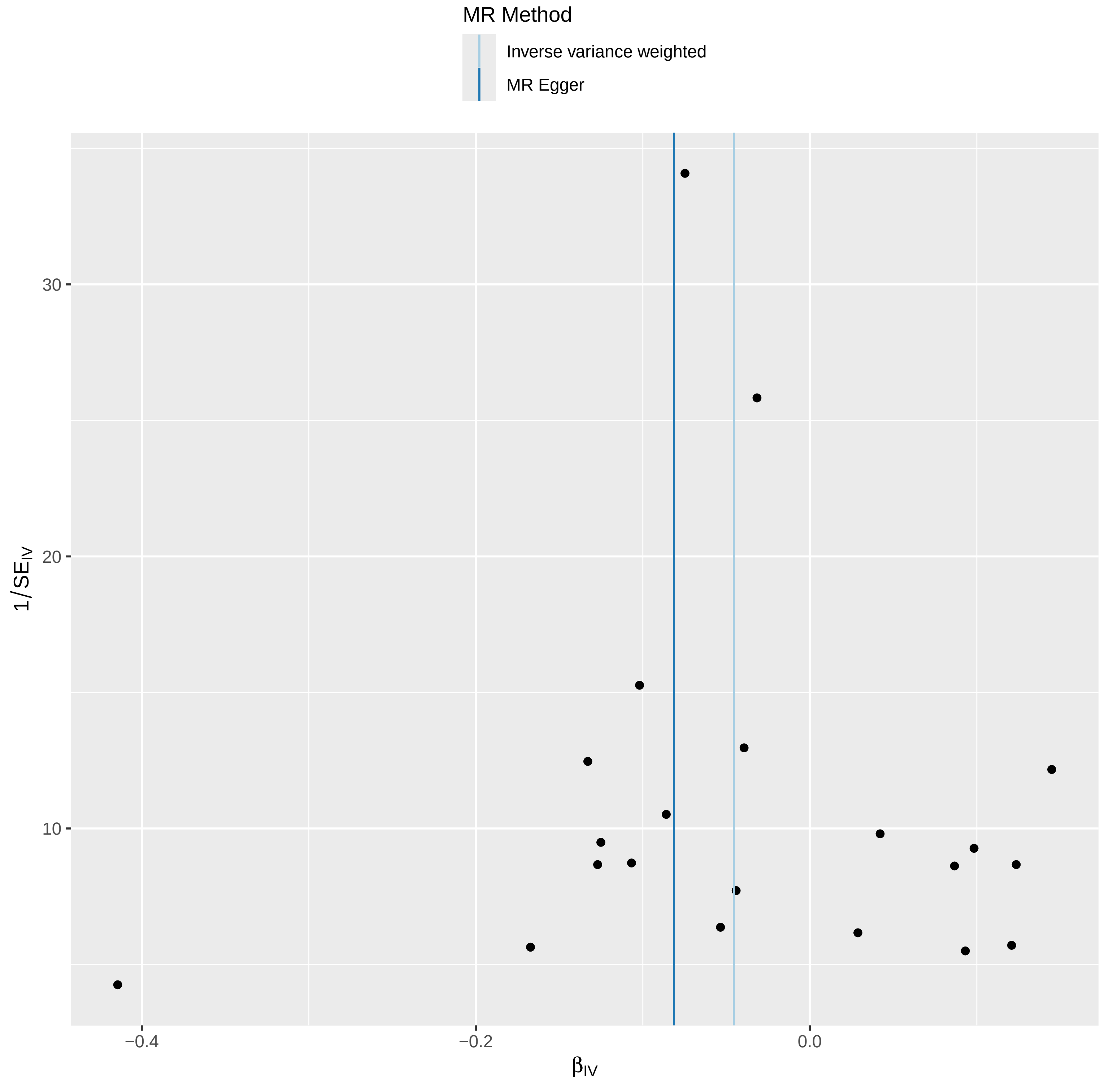

MR Method
Inverse variance weighted
MR Egger
30
20
10
1/SE_IV
−0.4
−0.2
0.0
β_IV

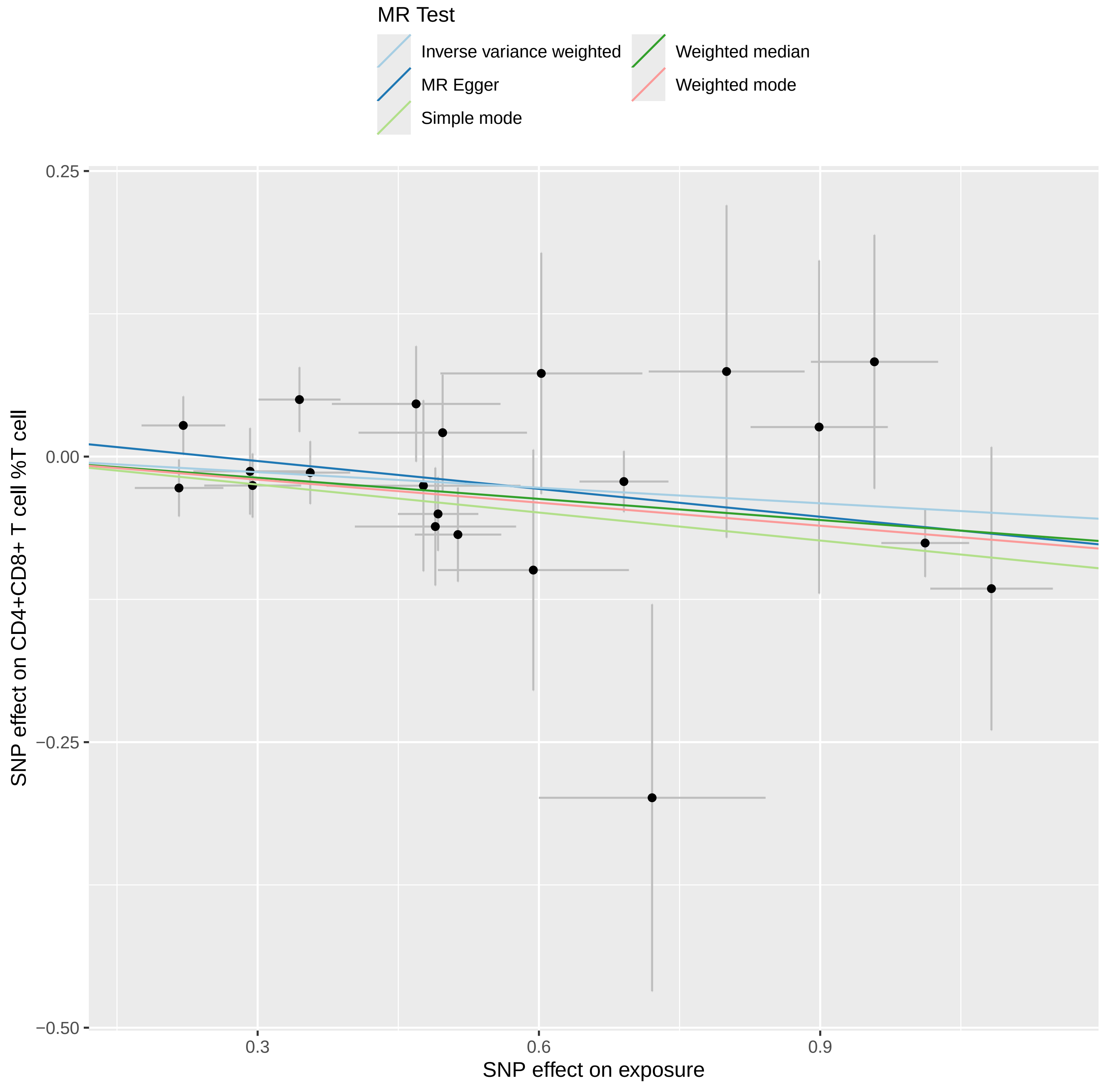

MR Test
Inverse variance weighted
MR Egger
Simple mode
Weighted median
Weighted mode
0.25
0.00
−0.25
−0.50
0.3
0.6
0.9
SNP effect on exposure
SNP effect on CD4+CD8+ T cell %T cell

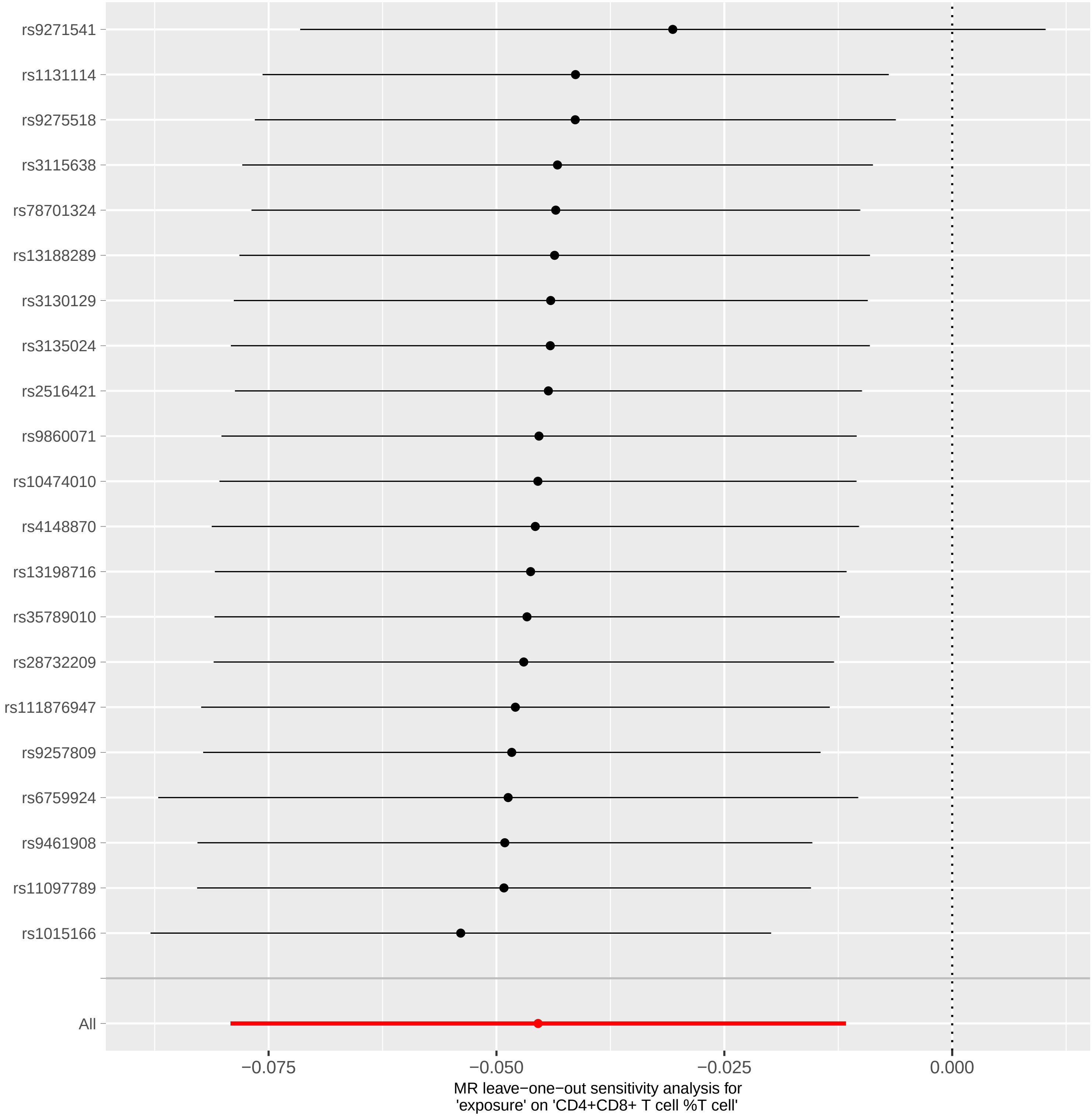

rs9271541
rs1131114
rs9275518
rs3115638
rs78701324
rs13188289
rs3130129
rs3135024
rs2516421
rs9860071
rs10474010
rs4148870
rs13198716
rs35789010
rs28732209
rs111876947
rs9257809
rs6759924
rs9461908
rs11097789
rs1015166
All
−0.075
−0.050
−0.025
0.000
MR leave−one−out sensitivity analysis for
'exposure' on 'CD4+CD8+ T cell %T cell'

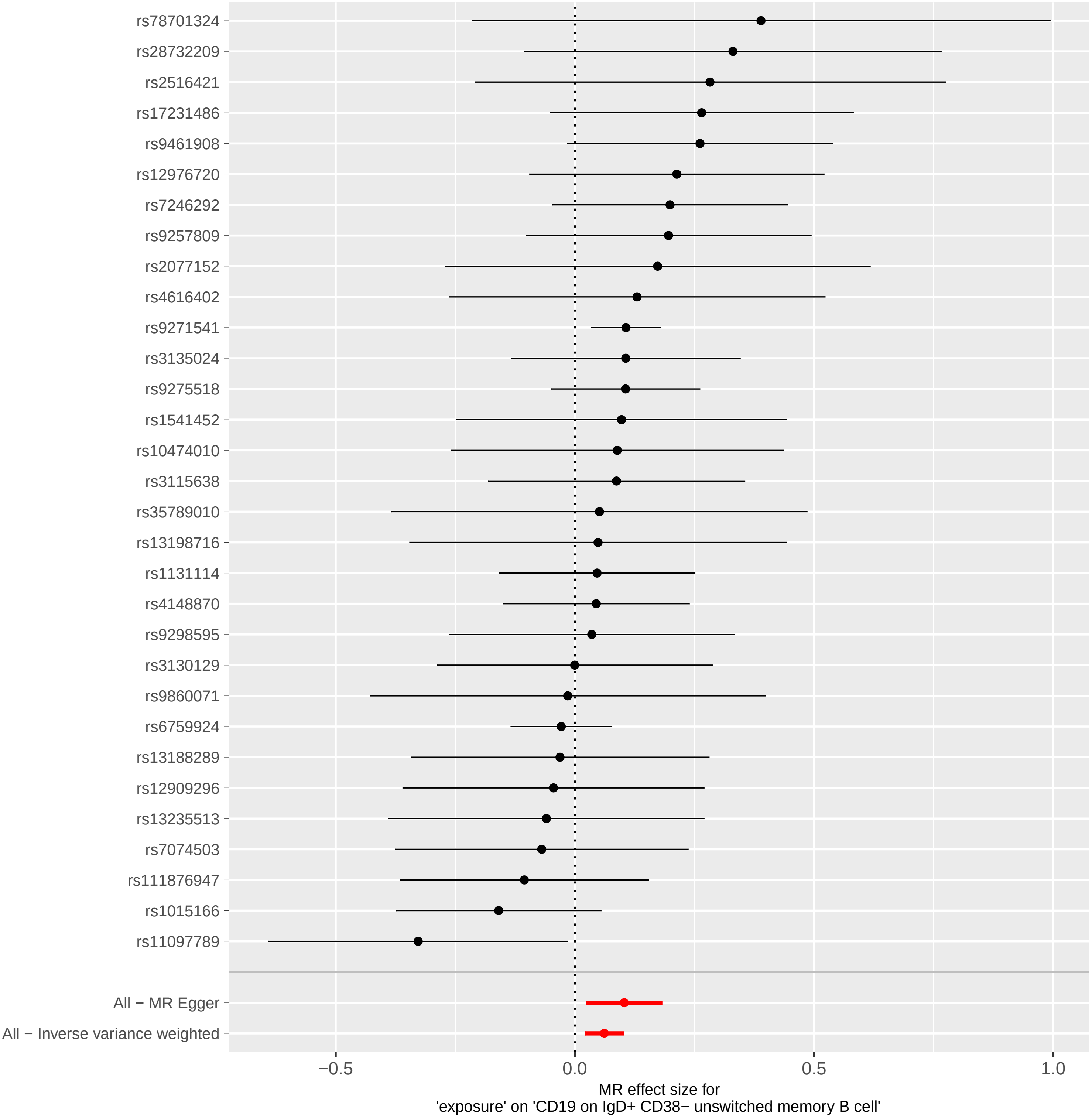

rs78701324
rs28732209
rs2516421
rs17231486
rs9461908
rs12976720
rs7246292
rs9257809
rs2077152
rs4616402
rs9271541
rs3135024
rs9275518
rs1541452
rs10474010
rs3115638
rs35789010
rs13198716
rs1131114
rs4148870
rs9298595
rs3130129
rs9860071
rs6759924
rs13188289
rs12909296
rs13235513
rs7074503
rs111876947
rs1015166
rs11097789
All − MR Egger
All − Inverse variance weighted
−0.5
0.0
0.5
1.0
MR effect size for
'exposure' on 'CD19 on IgD+ CD38− unswitched memory B cell'

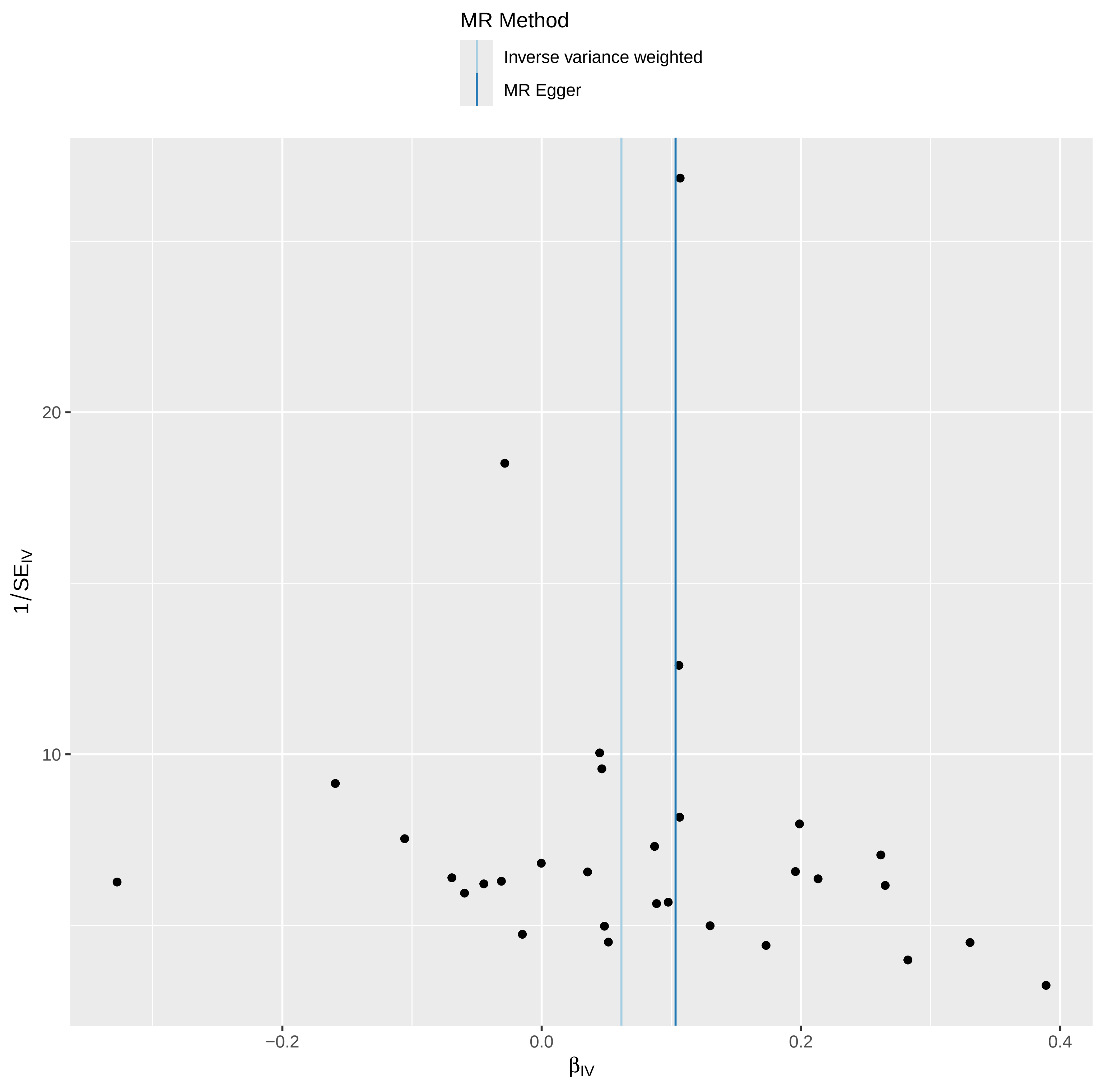

MR Method
Inverse variance weighted
MR Egger
20
10
1/SE_IV
−0.2
0.0
0.2
0.4
β_IV

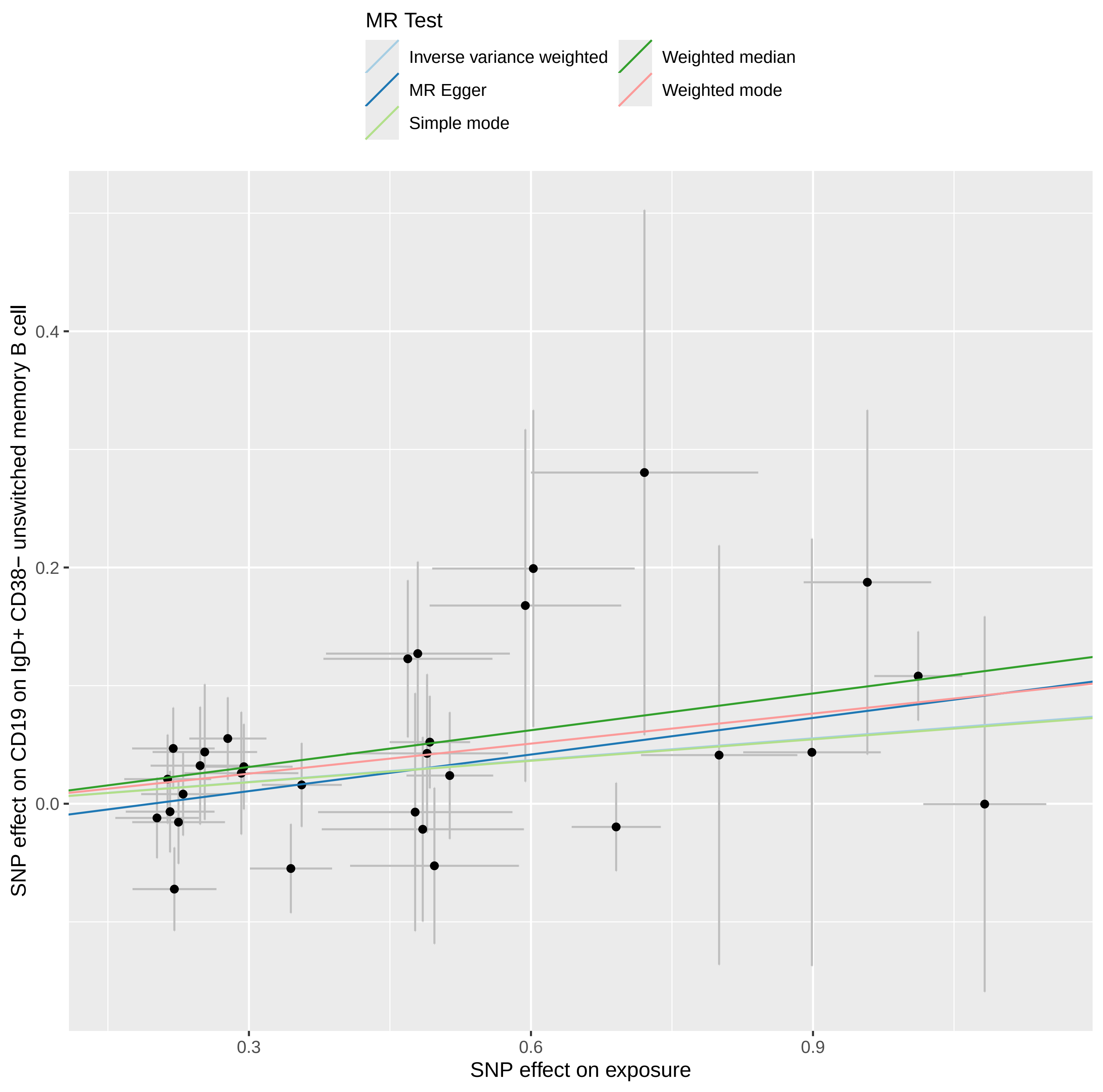
MR Test
Inverse variance weighted
MR Egger
Simple mode
Weighted median
Weighted mode
SNP effect on CD19 on IgD+ CD38− unswitched memory B cell
0.4
0.2
0.0
0.3
0.6
0.9
SNP effect on exposure

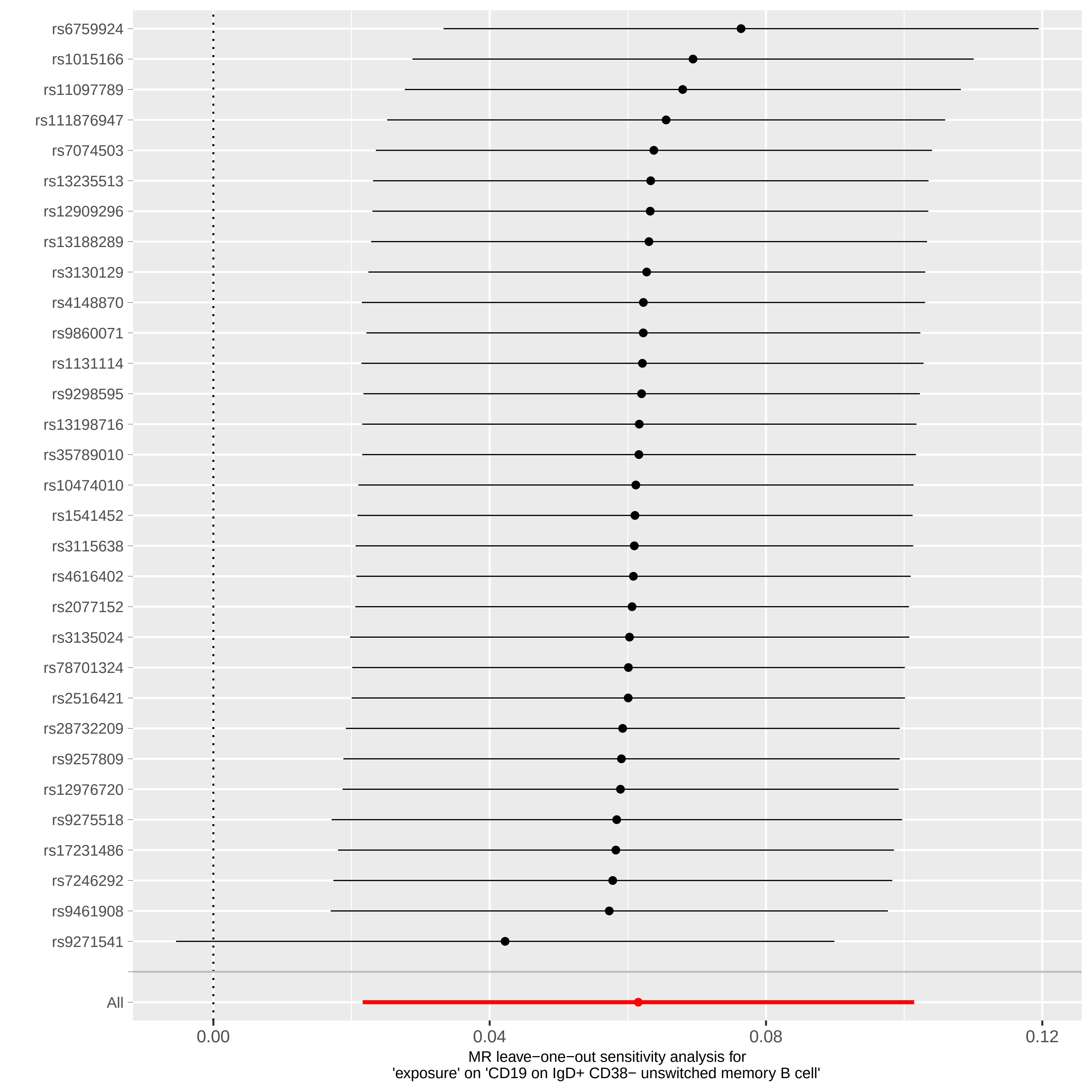
rs6759924
rs1015166
rs11097789
rs111876947
rs7074503
rs13235513
rs12909296
rs13188289
rs3130129
rs4148870
rs9860071
rs1131114
rs9298595
rs13198716
rs35789010
rs10474010
rs1541452
rs3115638
rs4616402
rs2077152
rs3135024
rs78701324
rs2516421
rs28732209
rs9257809
rs12976720
rs9275518
rs17231486
rs7246292
rs9461908
rs9271541
All
0.00
0.04
0.08
0.12
MR leave−one−out sensitivity analysis for
'exposure' on 'CD19 on IgD+ CD38− unswitched memory B cell'

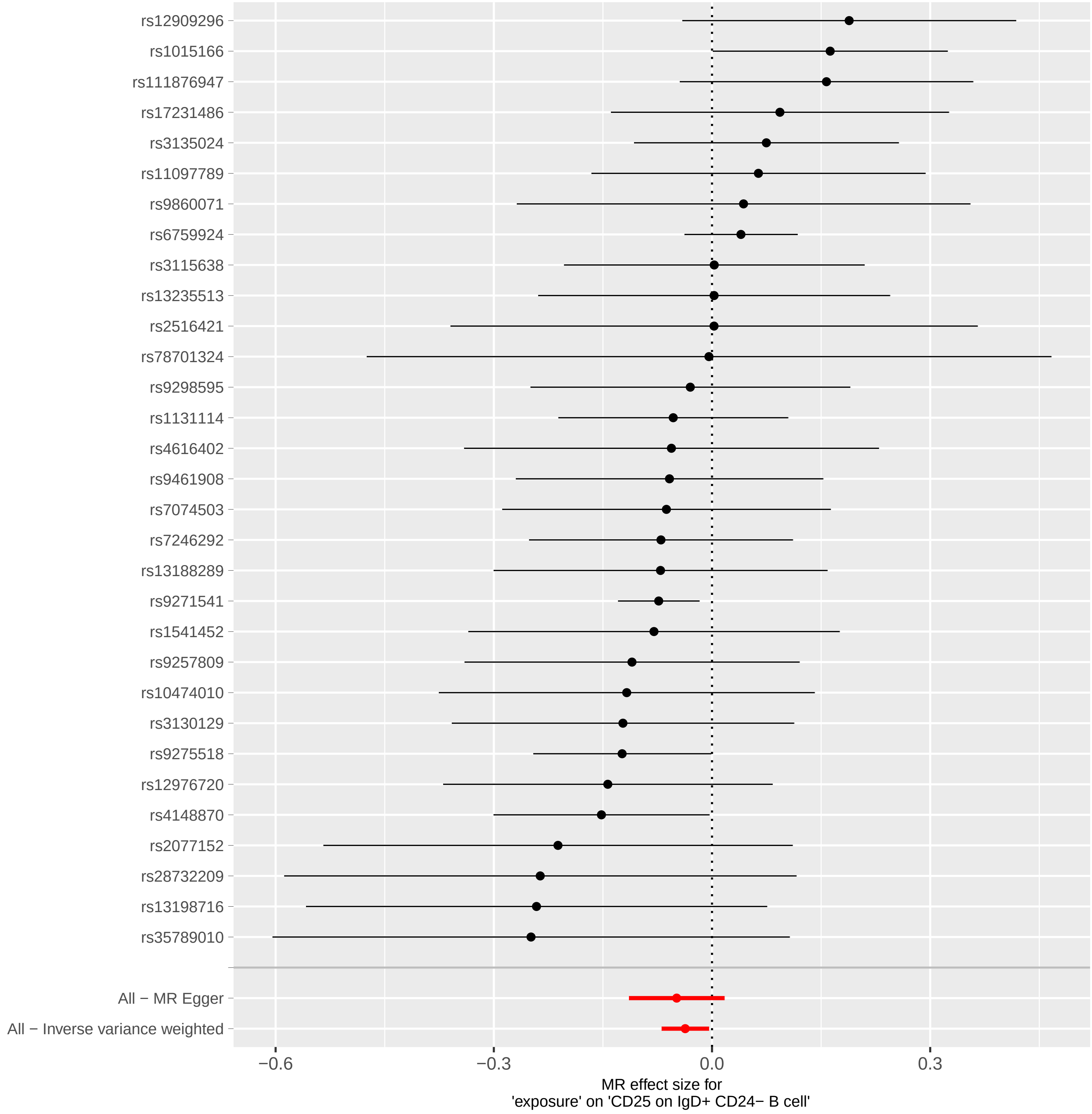
rs12909296
rs1015166
rs111876947
rs17231486
rs3135024
rs11097789
rs9860071
rs6759924
rs3115638
rs13235513
rs2516421
rs78701324
rs9298595
rs1131114
rs4616402
rs9461908
rs7074503
rs7246292
rs13188289
rs9271541
rs1541452
rs9257809
rs10474010
rs3130129
rs9275518
rs12976720
rs4148870
rs2077152
rs28732209
rs13198716
rs35789010
All − MR Egger
All − Inverse variance weighted
−0.6
−0.3
0.0
0.3
MR effect size for
'exposure' on 'CD25 on IgD+ CD24− B cell'

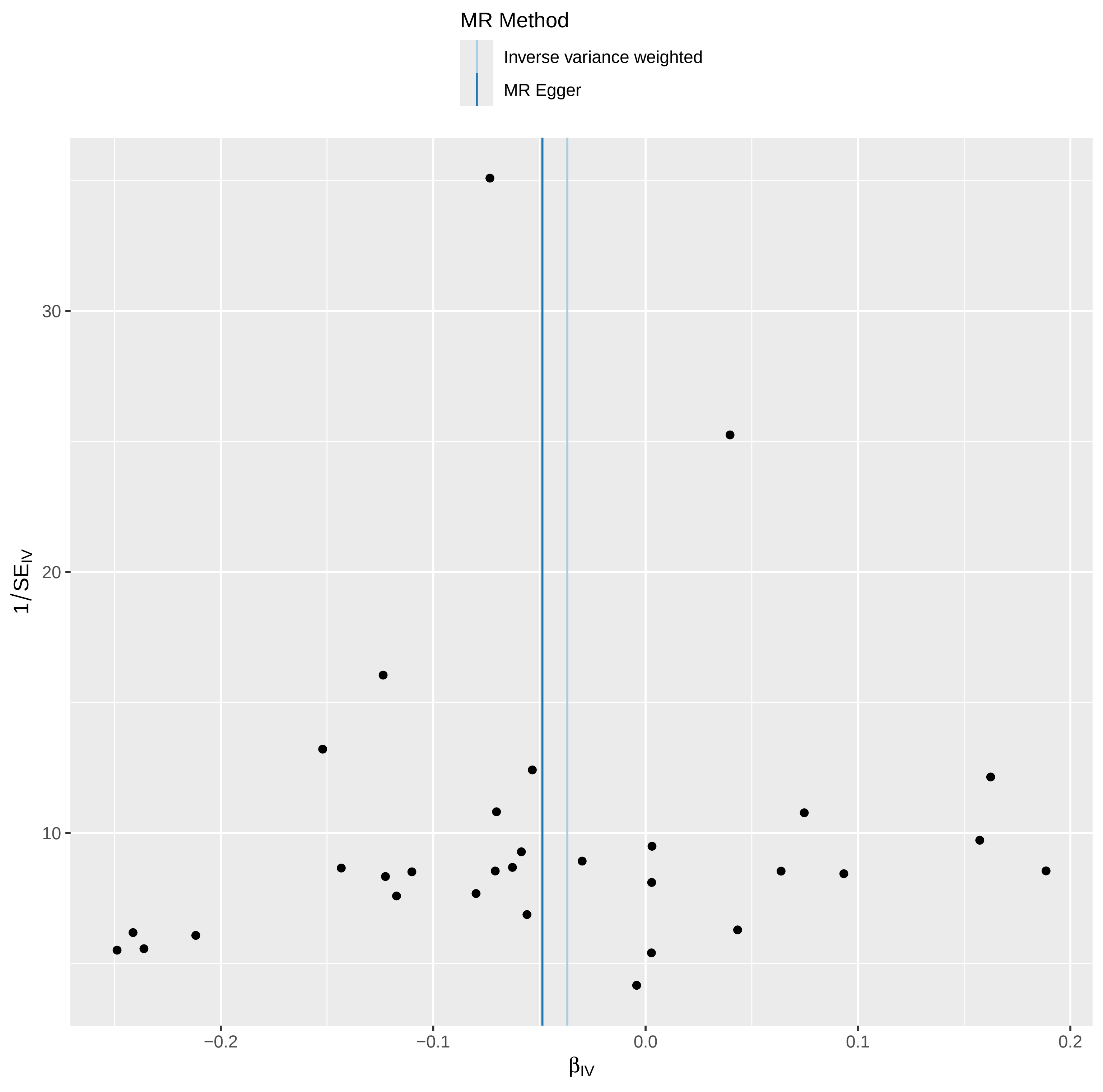
MR Method
Inverse variance weighted
MR Egger
30
20
10
1/SE$_{IV}$
−0.2
−0.1
0.0
0.1
0.2
β$_{IV}$

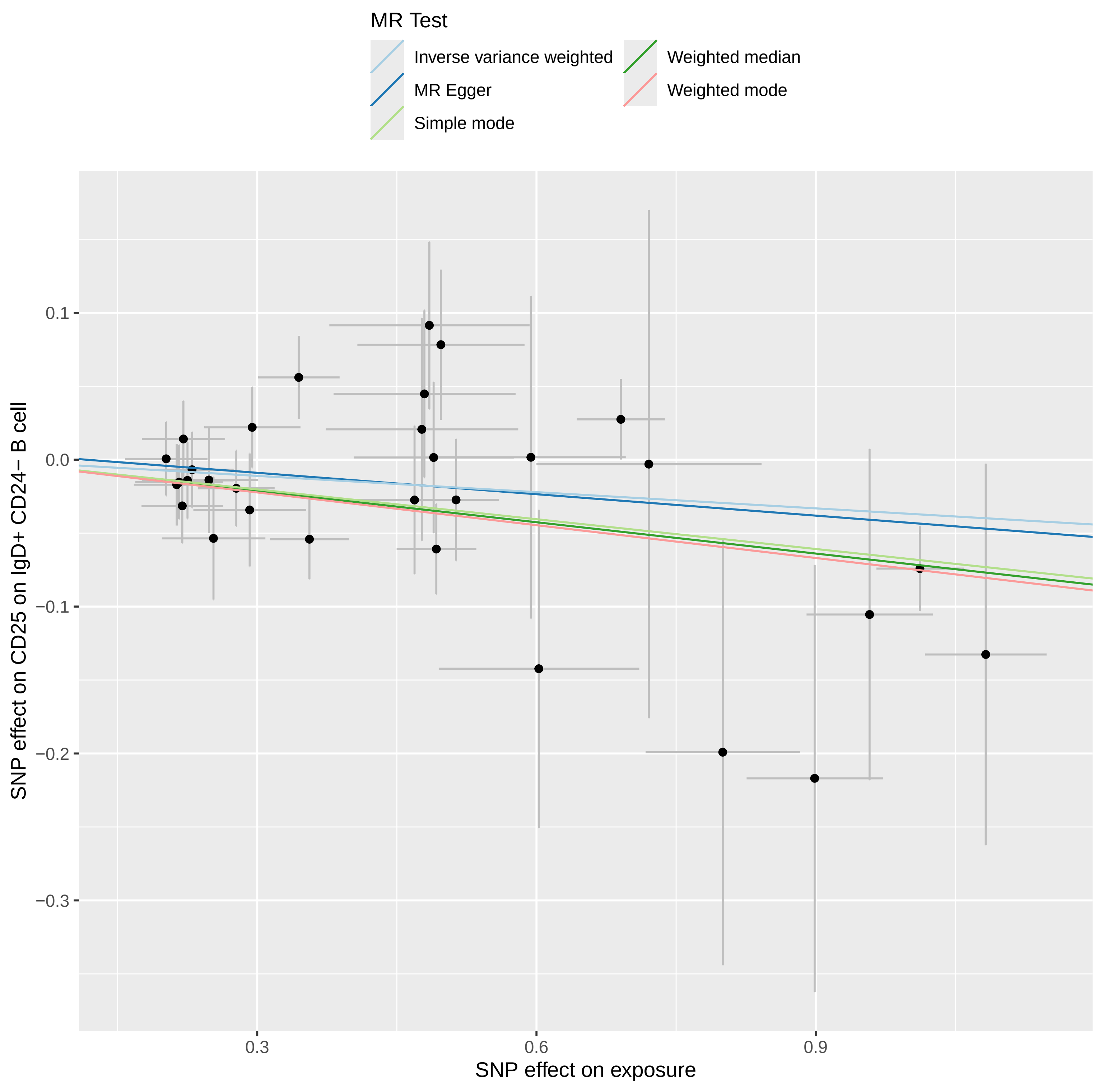
MR Test
Inverse variance weighted
MR Egger
Simple mode
Weighted median
Weighted mode
SNP effect on CD25 on IgD+ CD24− B cell
0.1
0.0
−0.1
−0.2
−0.3
0.3
0.6
0.9
SNP effect on exposure

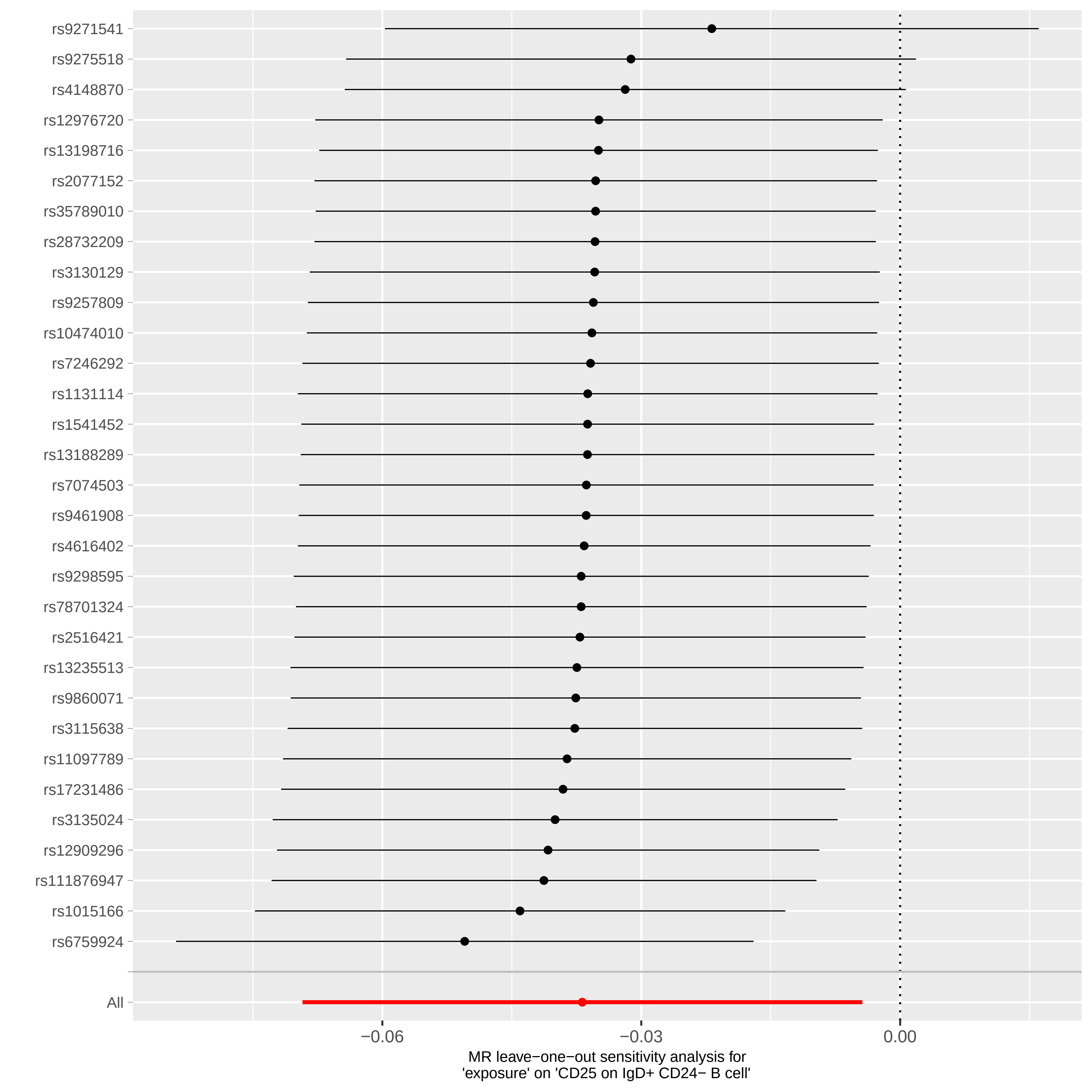
rs9271541
rs9275518
rs4148870
rs12976720
rs13198716
rs2077152
rs35789010
rs28732209
rs3130129
rs9257809
rs10474010
rs7246292
rs1131114
rs1541452
rs13188289
rs7074503
rs9461908
rs4616402
rs9298595
rs78701324
rs2516421
rs13235513
rs9860071
rs3115638
rs11097789
rs17231486
rs3135024
rs12909296
rs111876947
rs1015166
rs6759924
All
−0.06
−0.03
0.00
MR leave−one−out sensitivity analysis for
'exposure' on 'CD25 on IgD+ CD24− B cell'

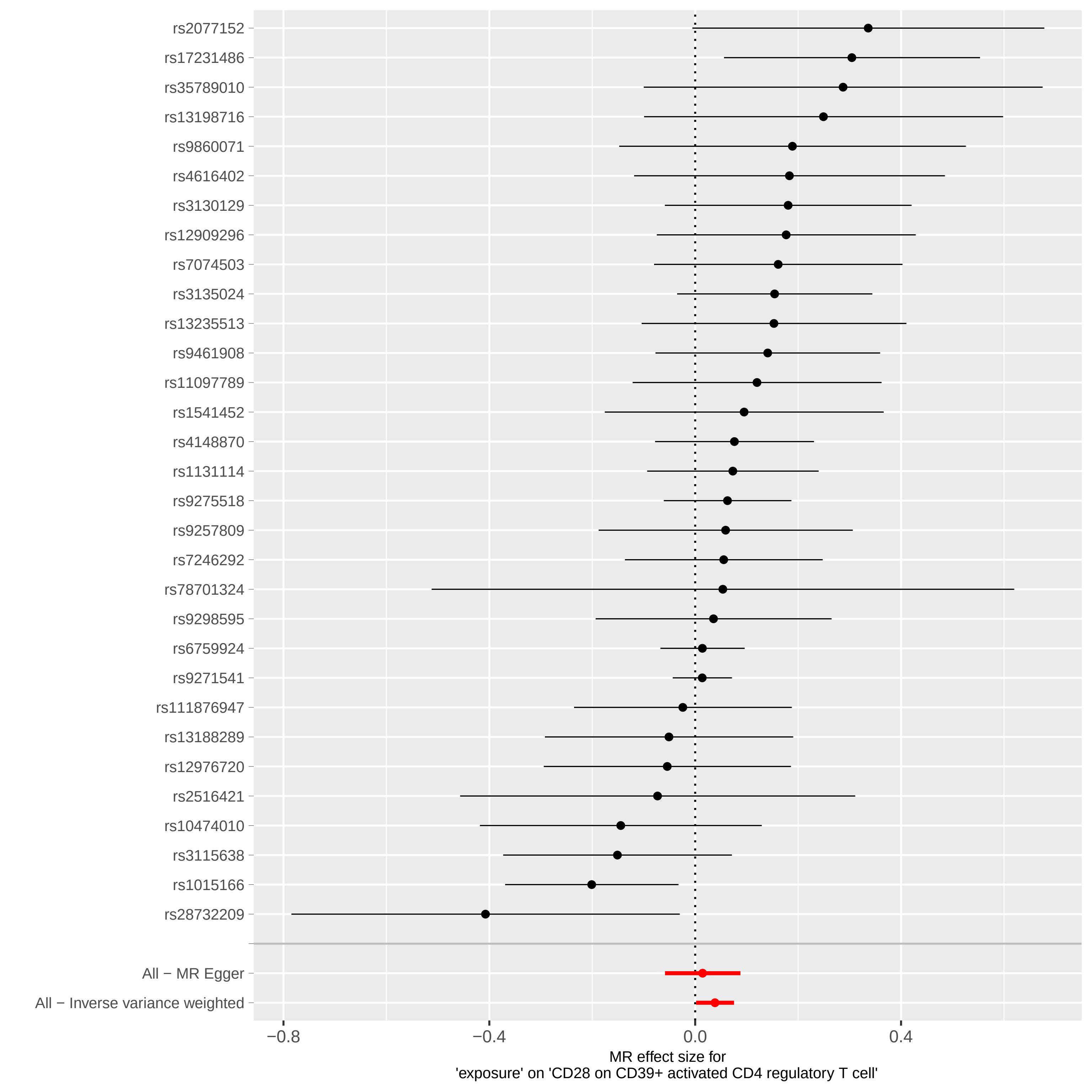

rs2077152
rs17231486
rs35789010
rs13198716
rs9860071
rs4616402
rs3130129
rs12909296
rs7074503
rs3135024
rs13235513
rs9461908
rs11097789
rs1541452
rs4148870
rs1131114
rs9275518
rs9257809
rs7246292
rs78701324
rs9298595
rs6759924
rs9271541
rs111876947
rs13188289
rs12976720
rs2516421
rs10474010
rs3115638
rs1015166
rs28732209
All − MR Egger
All − Inverse variance weighted
−0.8
−0.4
0.0
0.4
MR effect size for
'exposure' on 'CD28 on CD39+ activated CD4 regulatory T cell'

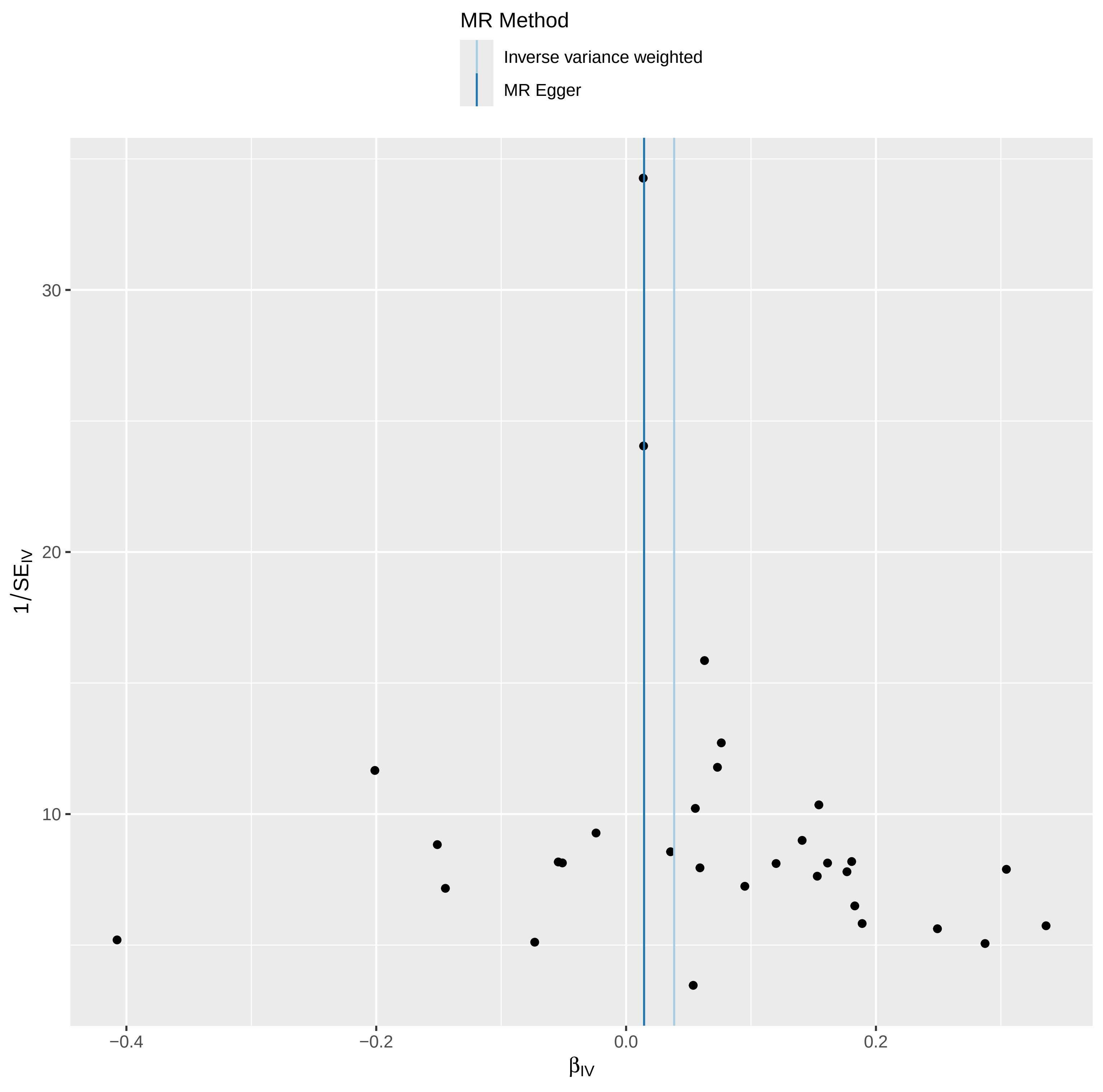
MR Method
Inverse variance weighted
MR Egger
30
20
10
$1/SE_{IV}$
−0.4
−0.2
0.0
0.2
β_{IV}

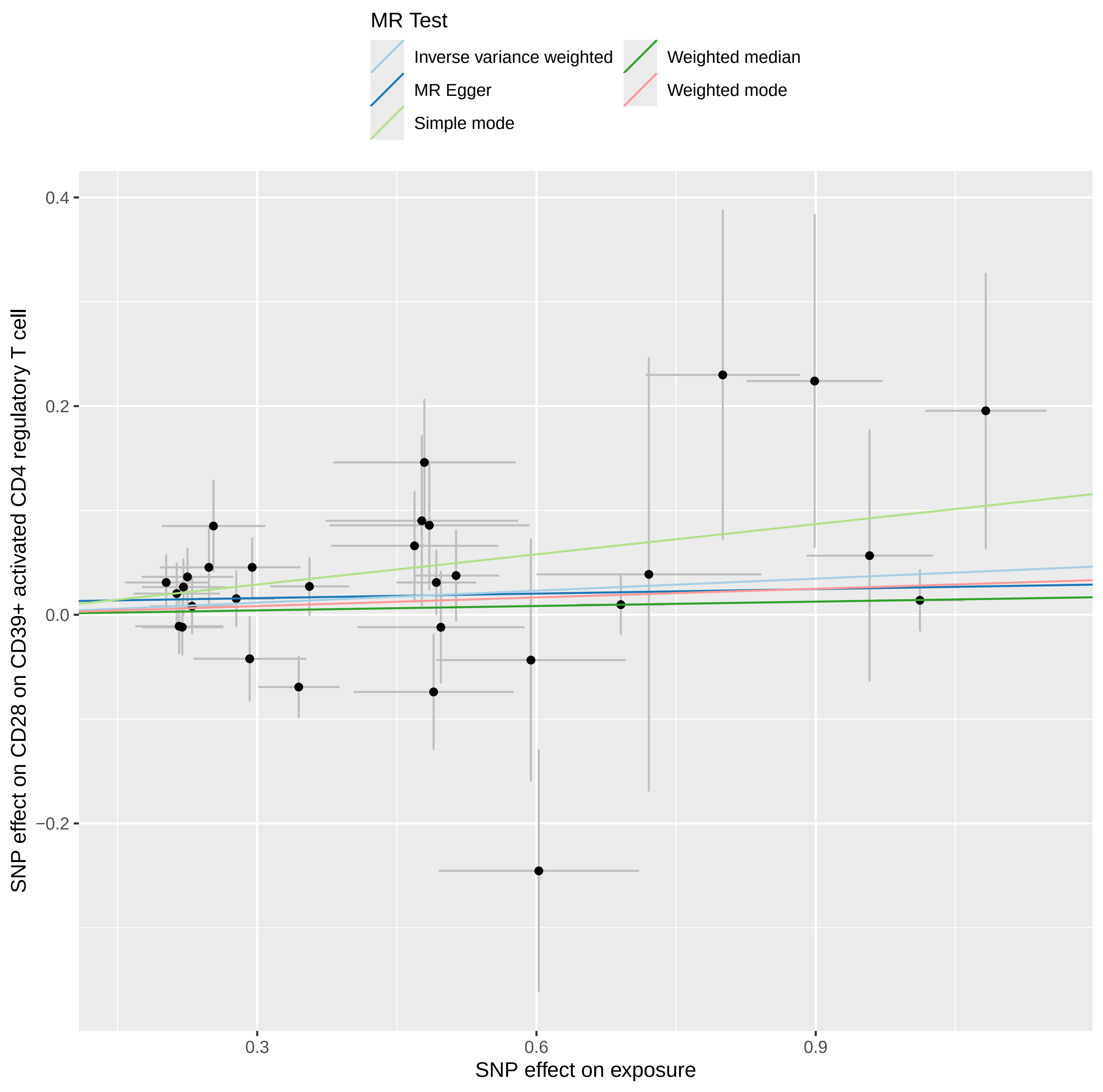
MR Test
Inverse variance weighted
MR Egger
Simple mode
Weighted median
Weighted mode
0.4
0.2
0.0
−0.2
0.3
0.6
0.9
SNP effect on CD28 on CD39+ activated CD4 regulatory T cell
SNP effect on exposure

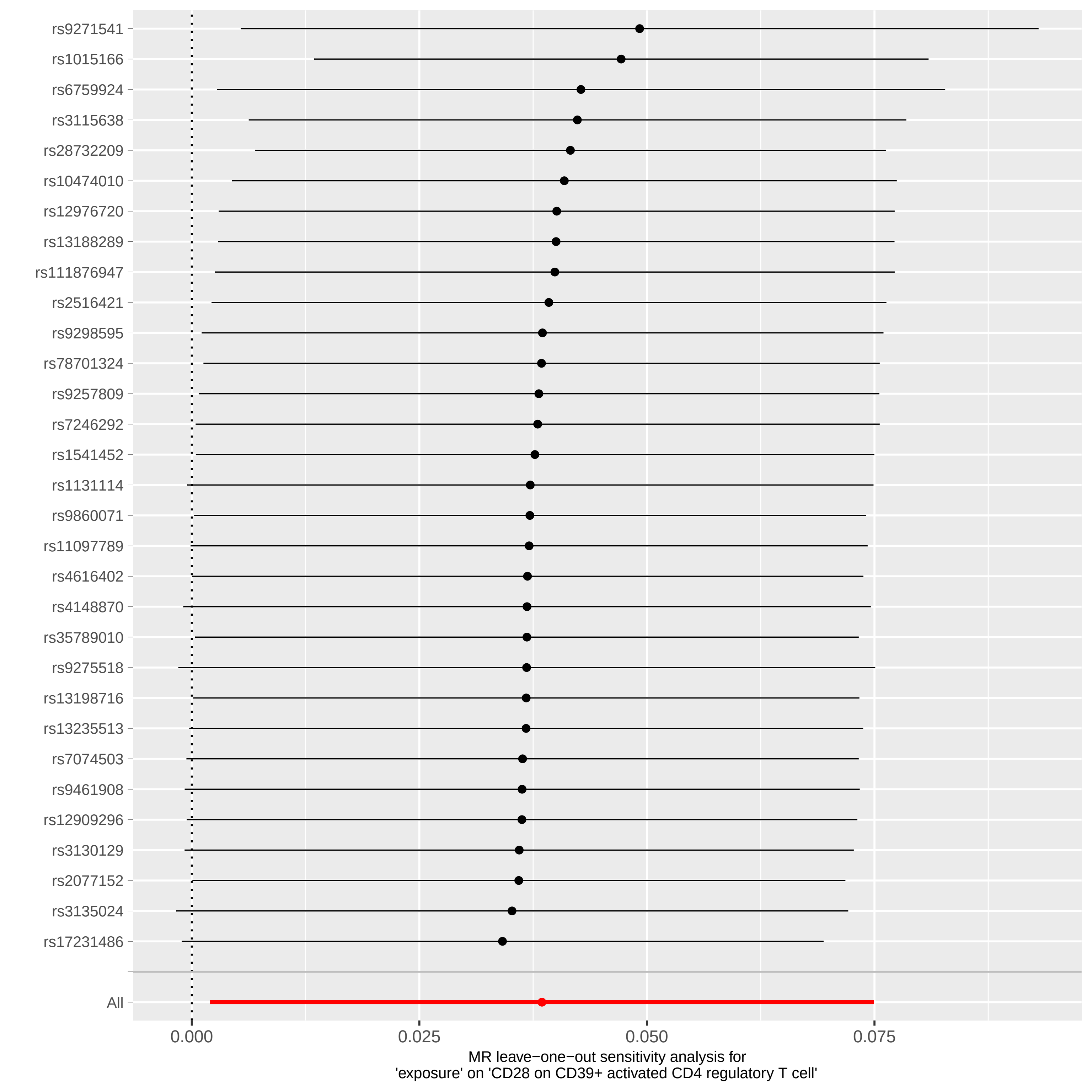
rs9271541
rs1015166
rs6759924
rs3115638
rs28732209
rs10474010
rs12976720
rs13188289
rs111876947
rs2516421
rs9298595
rs78701324
rs9257809
rs7246292
rs1541452
rs1131114
rs9860071
rs11097789
rs4616402
rs4148870
rs35789010
rs9275518
rs13198716
rs13235513
rs7074503
rs9461908
rs12909296
rs3130129
rs2077152
rs3135024
rs17231486
All
0.000
0.025
0.050
0.075
MR leave−one−out sensitivity analysis for
'exposure' on 'CD28 on CD39+ activated CD4 regulatory T cell'

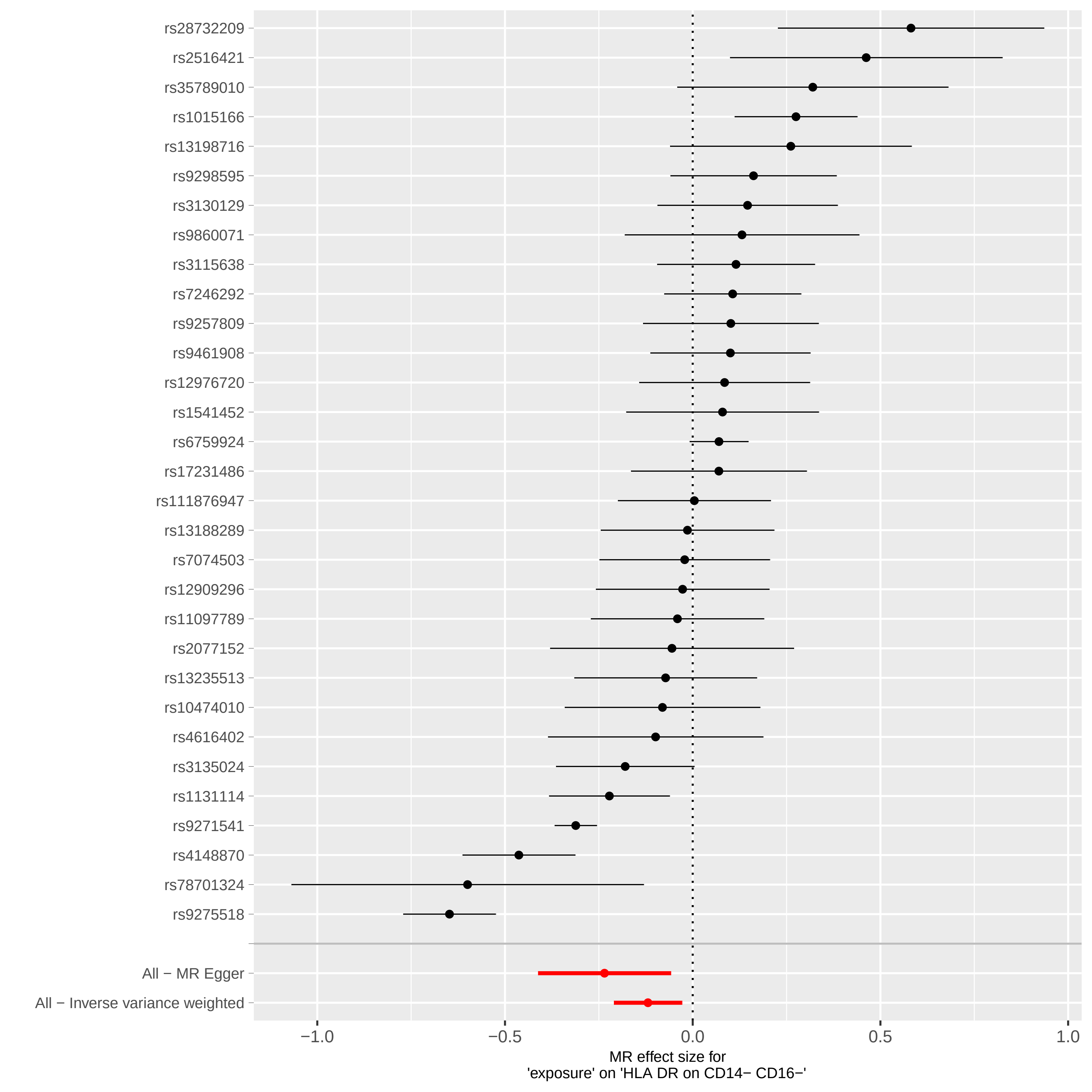

rs28732209
rs2516421
rs35789010
rs1015166
rs13198716
rs9298595
rs3130129
rs9860071
rs3115638
rs7246292
rs9257809
rs9461908
rs12976720
rs1541452
rs6759924
rs17231486
rs111876947
rs13188289
rs7074503
rs12909296
rs11097789
rs2077152
rs13235513
rs10474010
rs4616402
rs3135024
rs1131114
rs9271541
rs4148870
rs78701324
rs9275518
All − MR Egger
All − Inverse variance weighted
−1.0
−0.5
0.0
0.5
1.0
MR effect size for
'exposure' on 'HLA DR on CD14− CD16−'

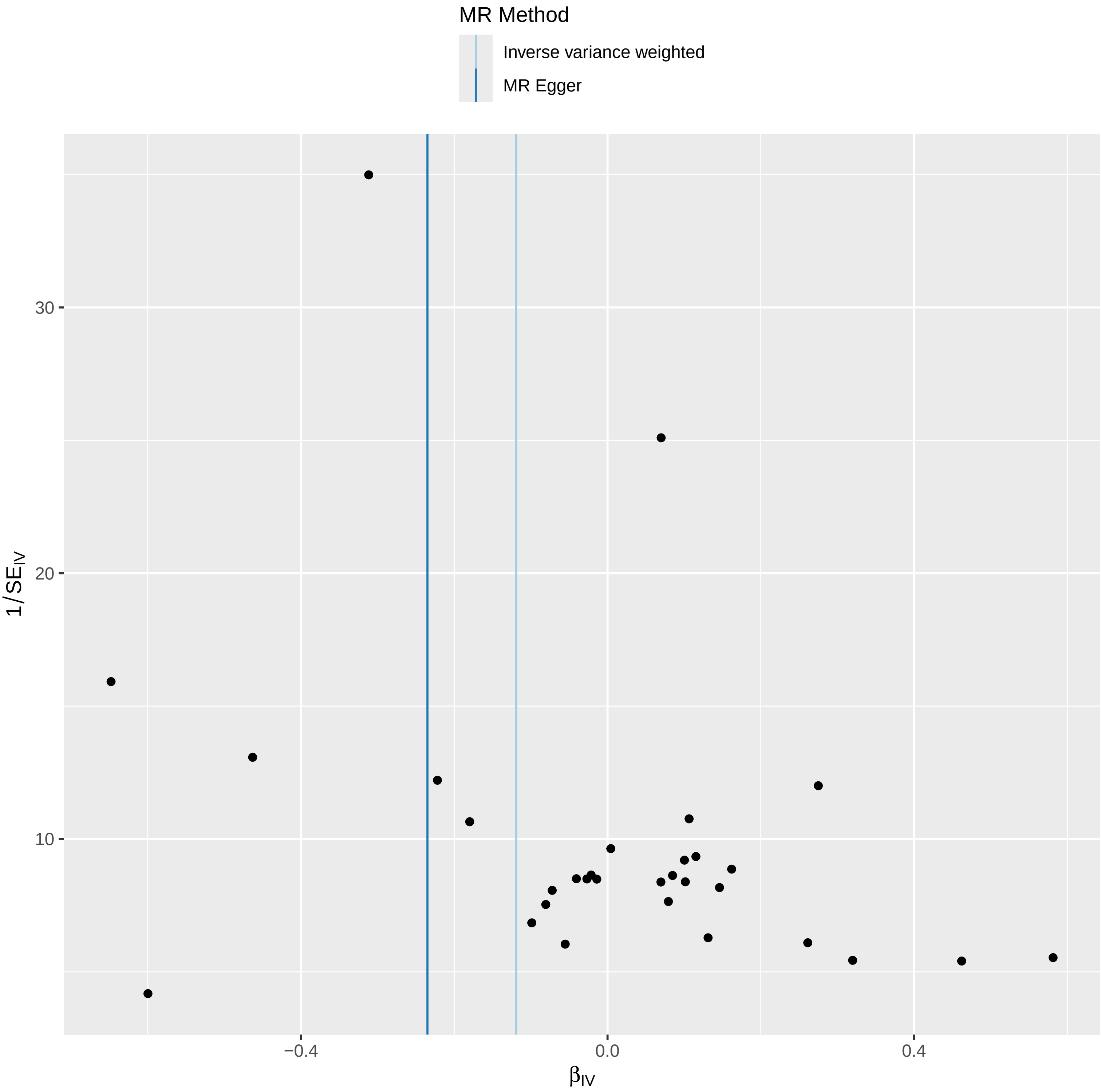

MR Method
Inverse variance weighted
MR Egger
30
20
10
1/SE$_{IV}$
−0.4
0.0
0.4
β_{IV}

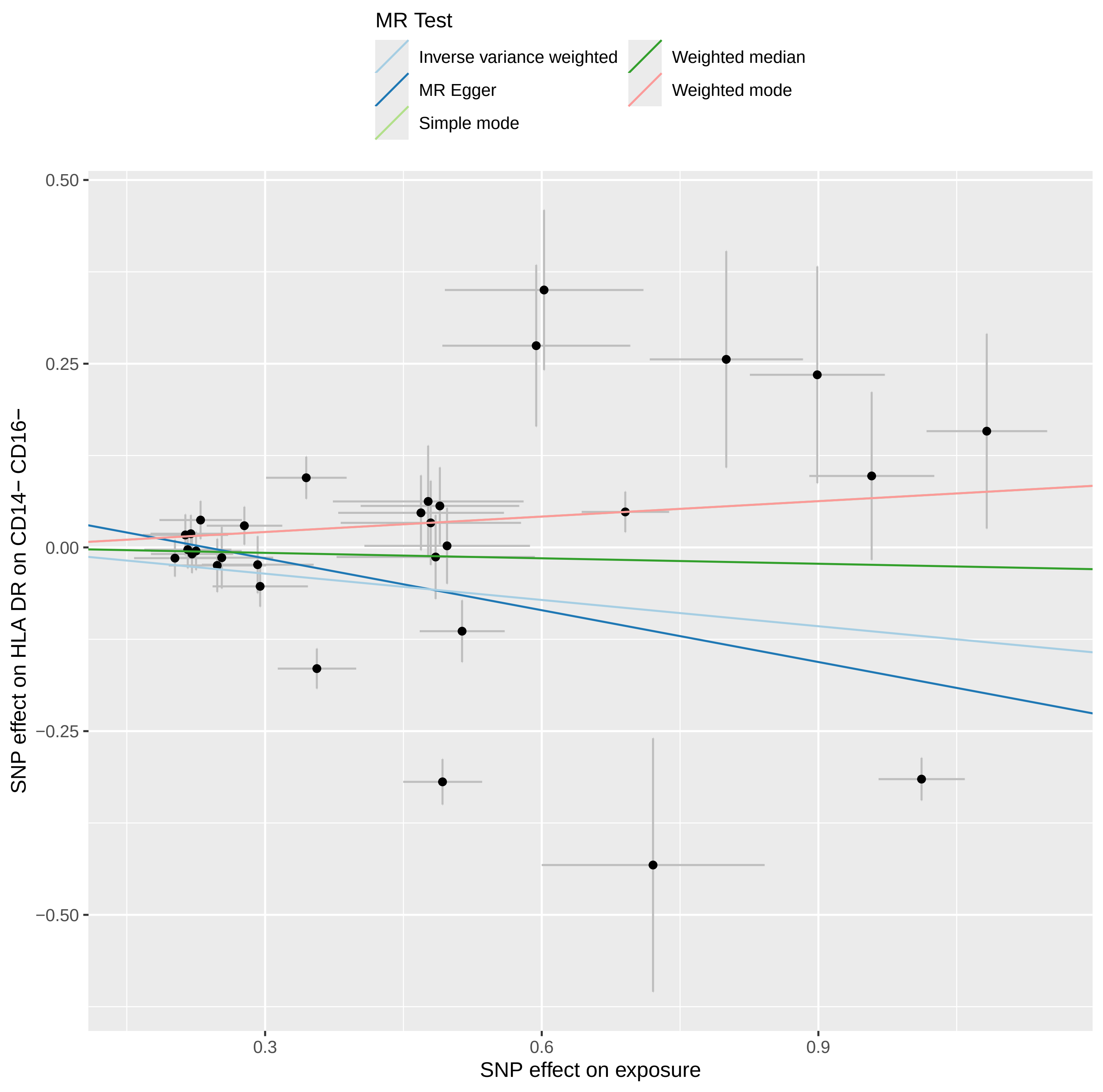
MR Test
Inverse variance weighted
MR Egger
Simple mode
Weighted median
Weighted mode
0.50
0.25
0.00
−0.25
−0.50
0.3
0.6
0.9
SNP effect on exposure
SNP effect on HLA DR on CD14− CD16−

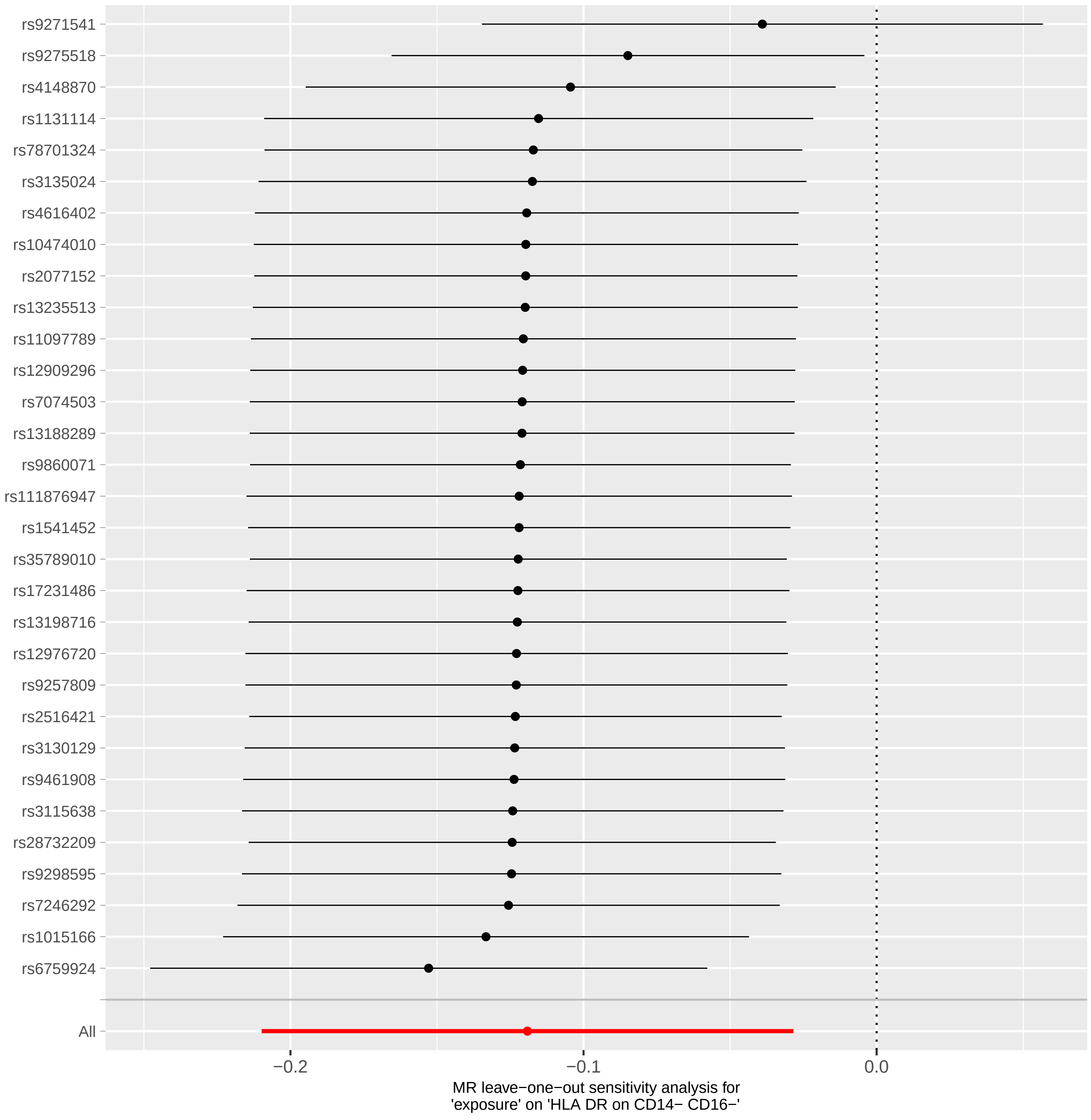
rs9271541
rs9275518
rs4148870
rs1131114
rs78701324
rs3135024
rs4616402
rs10474010
rs2077152
rs13235513
rs11097789
rs12909296
rs7074503
rs13188289
rs9860071
rs111876947
rs1541452
rs35789010
rs17231486
rs13198716
rs12976720
rs9257809
rs2516421
rs3130129
rs9461908
rs3115638
rs28732209
rs9298595
rs7246292
rs1015166
rs6759924
All
−0.2
−0.1
0.0
MR leave−one−out sensitivity analysis for
'exposure' on 'HLA DR on CD14− CD16−'

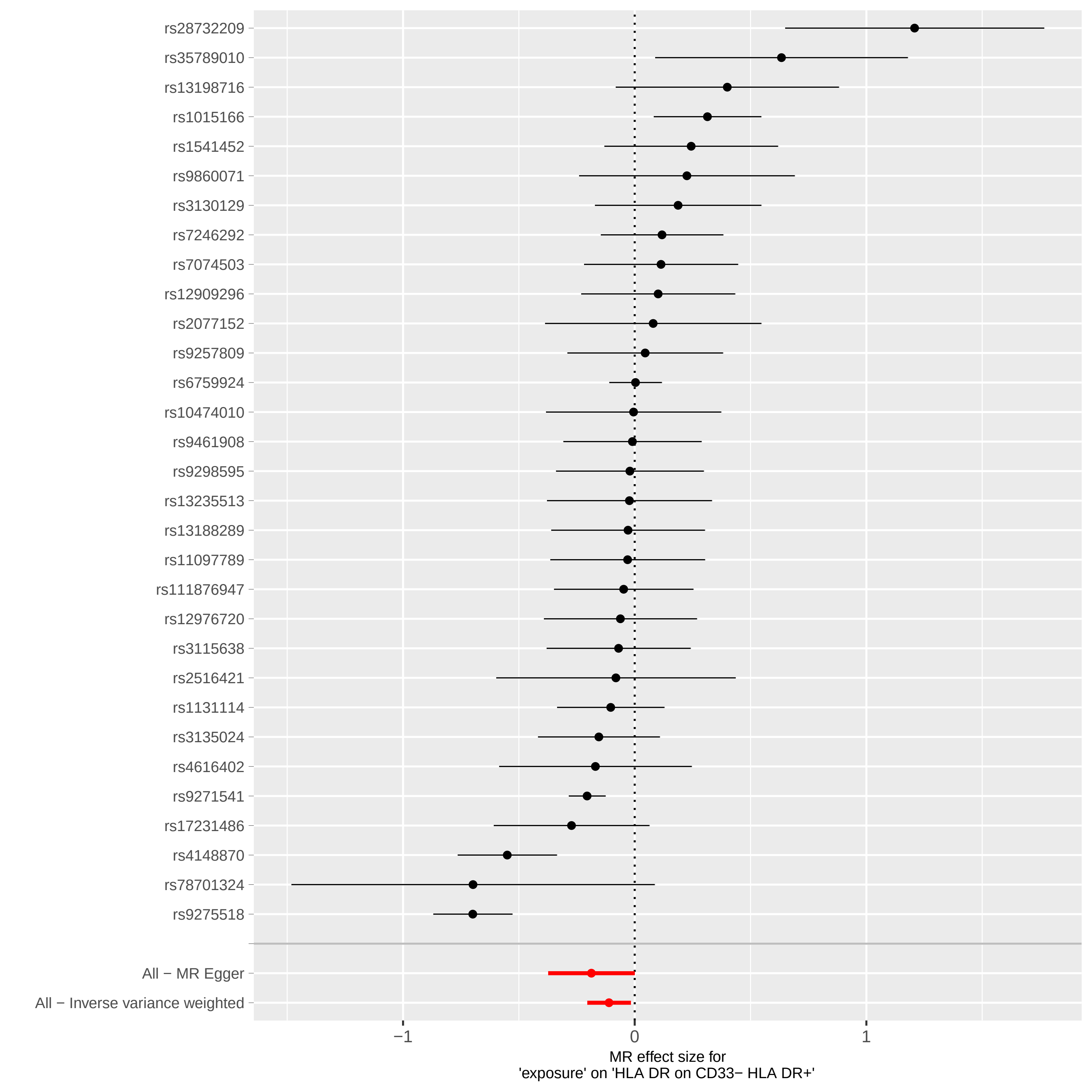
rs28732209
rs35789010
rs13198716
rs1015166
rs1541452
rs9860071
rs3130129
rs7246292
rs7074503
rs12909296
rs2077152
rs9257809
rs6759924
rs10474010
rs9461908
rs9298595
rs13235513
rs13188289
rs11097789
rs111876947
rs12976720
rs3115638
rs2516421
rs1131114
rs3135024
rs4616402
rs9271541
rs17231486
rs4148870
rs78701324
rs9275518
All − MR Egger
All − Inverse variance weighted
−1
0
1
MR effect size for
'exposure' on 'HLA DR on CD33− HLA DR+'

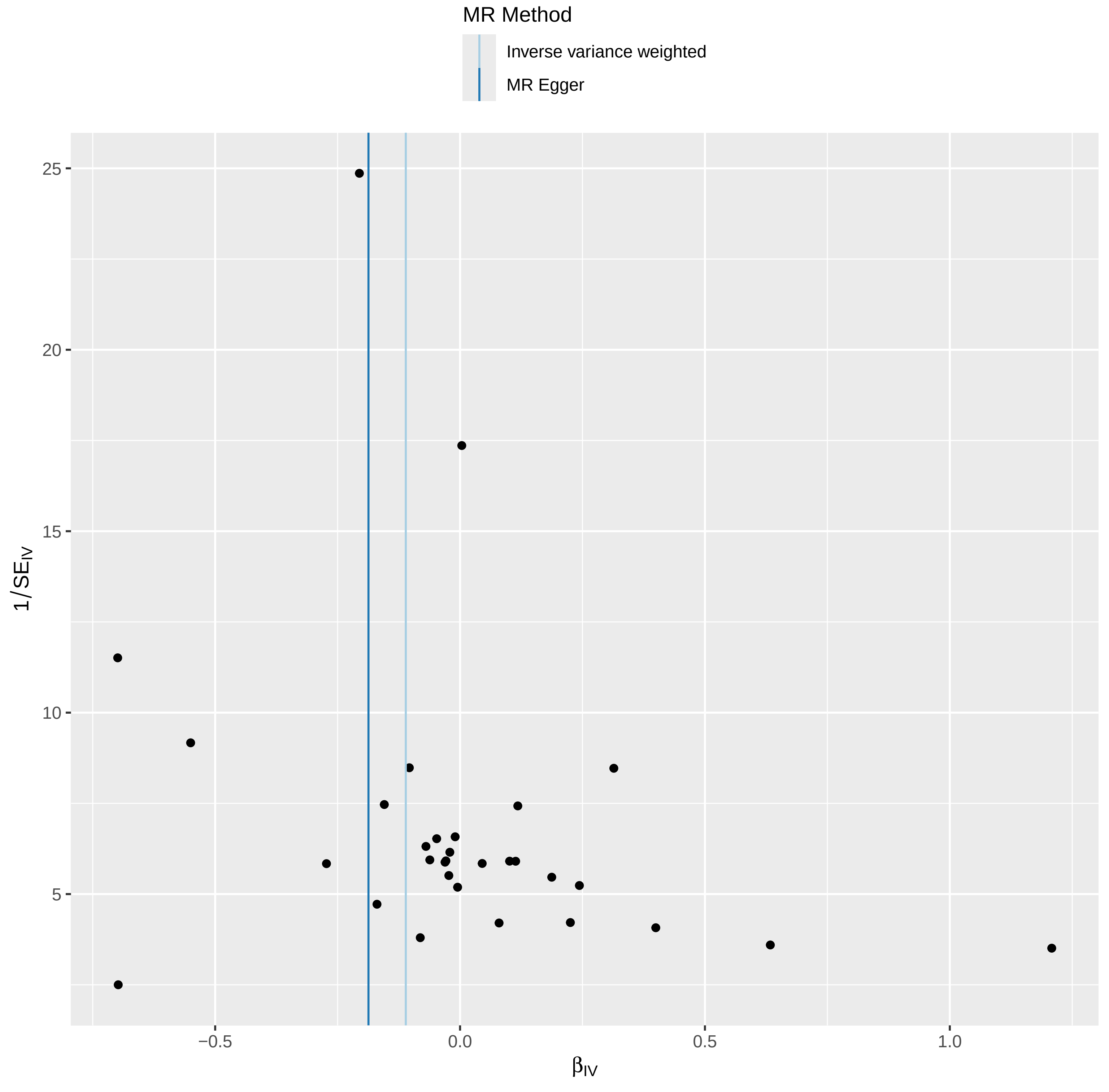
MR Method
Inverse variance weighted
MR Egger
25
20
15
10
5
1/SE_IV
−0.5
0.0
0.5
1.0
β_IV

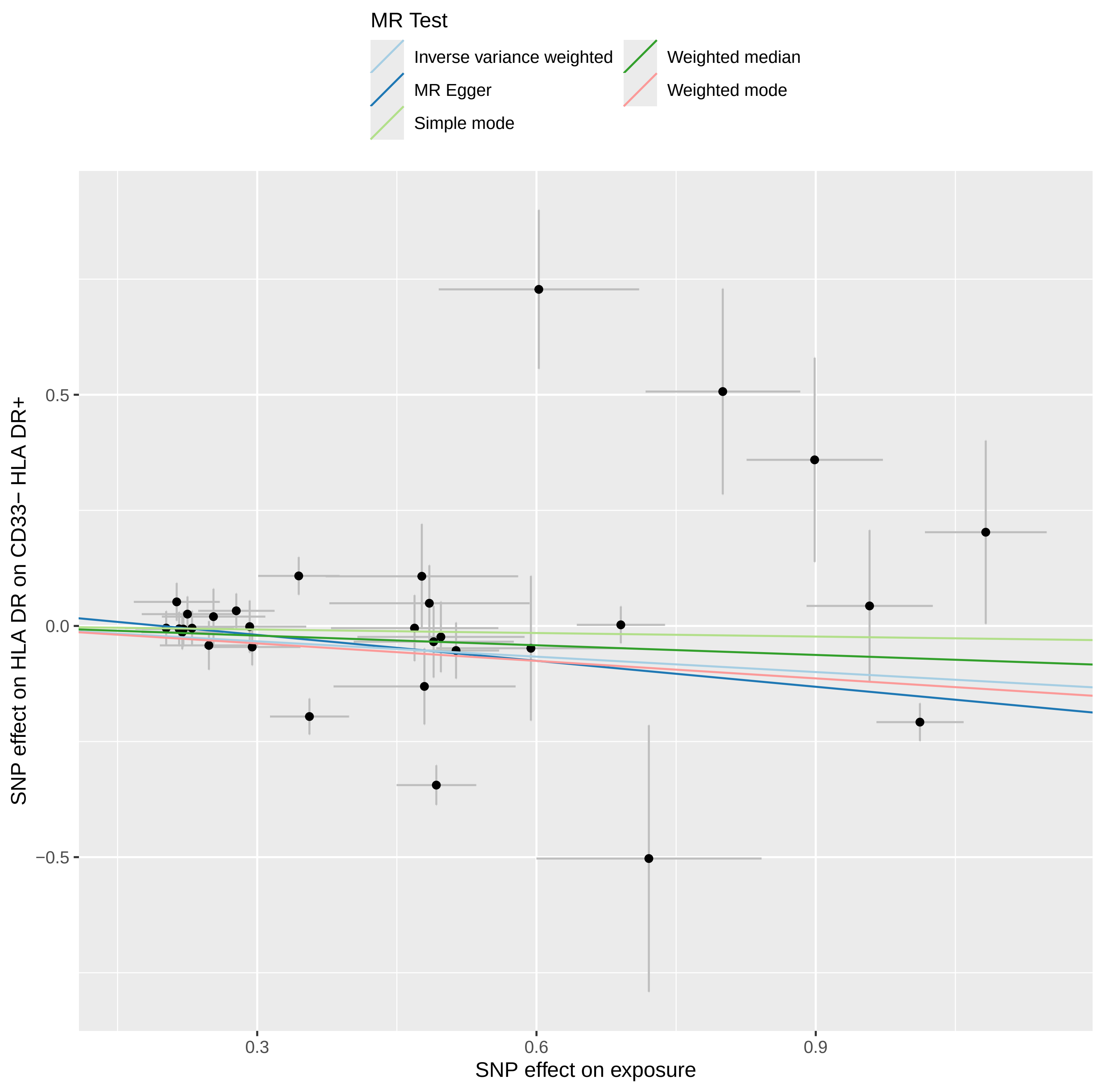
MR Test
Inverse variance weighted
MR Egger
Simple mode
Weighted median
Weighted mode
SNP effect on HLA DR on CD33− HLA DR+
0.5
0.0
−0.5
0.3
0.6
0.9
SNP effect on exposure

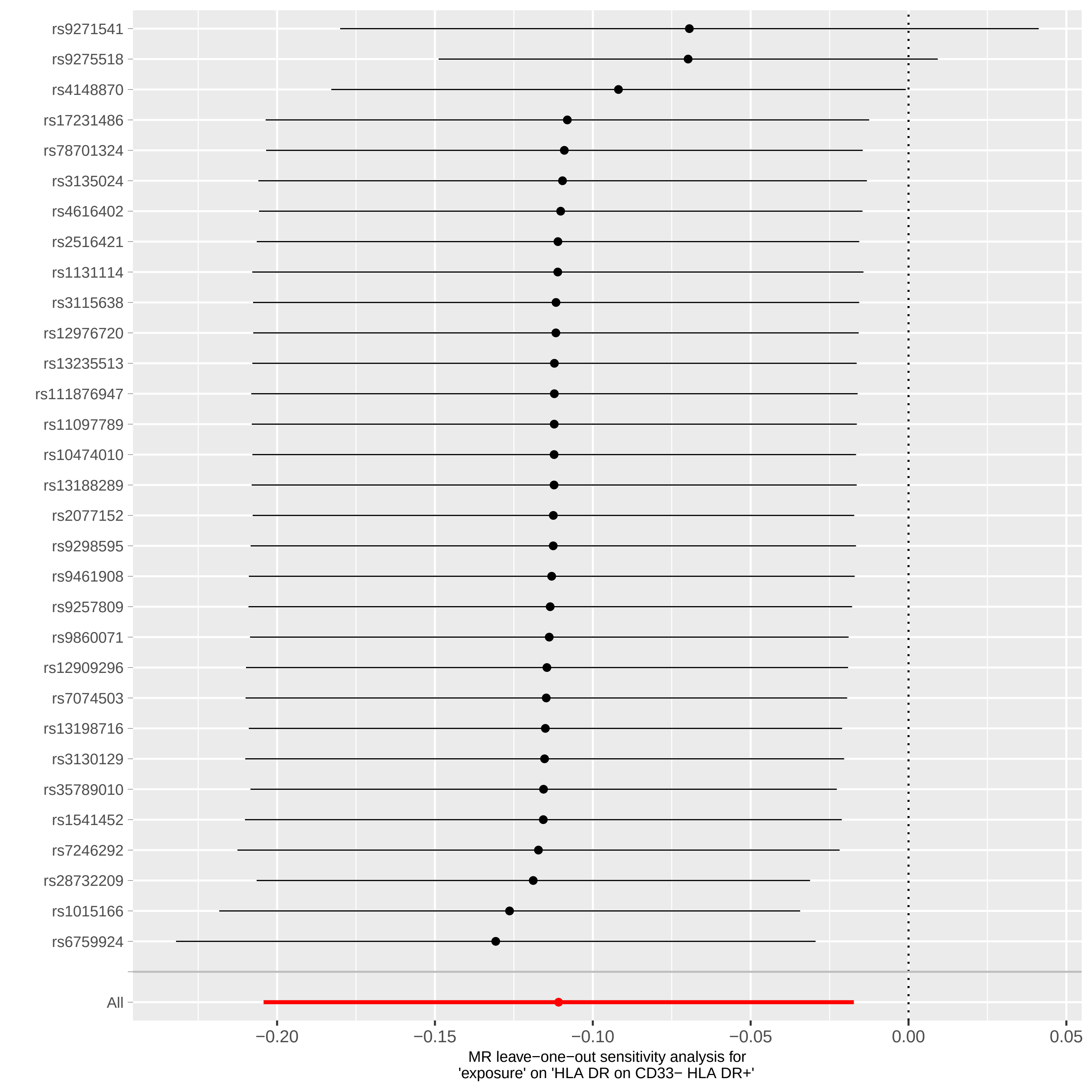
rs9271541
rs9275518
rs4148870
rs17231486
rs78701324
rs3135024
rs4616402
rs2516421
rs1131114
rs3115638
rs12976720
rs13235513
rs111876947
rs11097789
rs10474010
rs13188289
rs2077152
rs9298595
rs9461908
rs9257809
rs9860071
rs12909296
rs7074503
rs13198716
rs3130129
rs35789010
rs1541452
rs7246292
rs28732209
rs1015166
rs6759924
All
−0.20
−0.15
−0.10
−0.05
0.00
0.05
MR leave−one−out sensitivity analysis for
'exposure' on 'HLA DR on CD33− HLA DR+'
